# Comparative Genomics Suggests an Independent Origin of Cytoplasmic Incompatibility in *Cardinium hertigii*

**DOI:** 10.1371/journal.pgen.1003012

**Published:** 2012-10-25

**Authors:** Thomas Penz, Stephan Schmitz-Esser, Suzanne E. Kelly, Bodil N. Cass, Anneliese Müller, Tanja Woyke, Stephanie A. Malfatti, Martha S. Hunter, Matthias Horn

**Affiliations:** 1 Department of Microbial Ecology, University of Vienna, Vienna, Austria; 2 Institute for Milk Hygiene, University of Veterinary Medicine Vienna, Vienna, Austria; 3 Department of Entomology, The University of Arizona, Tucson, Arizona, United States of America; 4 Graduate Interdisciplinary Program in Entomology and Insect Science, The University of Arizona, Tucson, Arizona, United States of America; 5 U.S. Department of Energy Joint Genome Institute, Walnut Creek, California, United States of America; Yale University, United States of America

## Abstract

Terrestrial arthropods are commonly infected with maternally inherited bacterial symbionts that cause cytoplasmic incompatibility (CI). In CI, the outcome of crosses between symbiont-infected males and uninfected females is reproductive failure, increasing the relative fitness of infected females and leading to spread of the symbiont in the host population. CI symbionts have profound impacts on host genetic structure and ecology and may lead to speciation and the rapid evolution of sex determination systems. *Cardinium hertigii*, a member of the *Bacteroidetes* and symbiont of the parasitic wasp *Encarsia pergandiella*, is the only known bacterium other than the *Alphaproteobacteria Wolbachia* to cause CI. Here we report the genome sequence of *Cardinium hertigii c*Eper1. Comparison with the genomes of CI–inducing *Wolbachia pipientis* strains *w*Mel, *w*Ri, and *w*Pip provides a unique opportunity to pinpoint shared proteins mediating host cell interaction, including some candidate proteins for CI that have not previously been investigated. The genome of *Cardinium* lacks all major biosynthetic pathways but harbors a complete biotin biosynthesis pathway, suggesting a potential role for *Cardinium* in host nutrition. *Cardinium* lacks known protein secretion systems but encodes a putative phage-derived secretion system distantly related to the antifeeding prophage of the entomopathogen *Serratia entomophila*. Lastly, while *Cardinium* and *Wolbachia* genomes show only a functional overlap of proteins, they show no evidence of laterally transferred elements that would suggest common ancestry of CI in both lineages. Instead, comparative genomics suggests an independent evolution of CI in *Cardinium* and *Wolbachia* and provides a novel context for understanding the mechanistic basis of CI.

## Author Summary

Many arthropods are infected with bacterial symbionts that are maternally transmitted and have a great impact on their hosts' biology, ecology, and evolution. One of the most common phenotypes of facultative symbionts appears to be cytoplasmic incompatibility (CI), a type of reproductive failure in which bacteria in males modify sperm in a way that reduces the reproductive success of uninfected female mates. In spite of considerable interest, the genetic basis for CI is largely unknown. *Cardinium hertigii*, a symbiont of tiny parasitic wasps, is the only bacterial group other than the well-studied *Wolbachia* that is known to cause CI. Analysis of the *Cardinium* genome indicates that CI evolved independently in *Wolbachia* and *Cardinium*. However, a suite of shared proteins was likely involved in mediating host cell interactions, and CI shows functional overlap in both lineages. Our analysis suggests the presence of an unusual phage-derived, putative secretion system and reveals that *Cardinium* encodes biosynthetic pathways that suggest a potential role in host nutrition. Our findings provide a novel comparative context for understanding the mechanistic basis of CI and substantially increase our knowledge on reproductive manipulator symbionts that do not only severely affect population genetic structure of arthropods but may also serve as powerful tools in pest management.

## Introduction

Bacterial symbionts of terrestrial arthropods are common, influential associates, known to affect fundamental aspects of the host life history, ecology, and evolution. These maternally inherited bacteria may, for example, provide essential nutrients supplementing their host's diet, confer protection against natural enemies, increase stress resistance, or influence host plant suitability [1]–[4]. Others have evolved sophisticated means of manipulating the arthropod host's reproduction in ways that cause the symbiont to spread within the host population [5]–[6]. Infection with reproductive manipulators may drive rapid evolution of host sex determination [7], affect genetic population structure, including reproductive isolation and speciation [8], as well as influence the evolution of sexual traits [9]. Reproductive manipulator symbionts may also be powerful tools in pest management for suppression or transformation of pest or vector populations [10]–[11].

The most common symbiont-induced reproductive manipulation, cytoplasmic incompatibility (CI), is also perhaps the most enigmatic. CI occurs, in the simplest case, when a symbiont-infected male host mates with an uninfected female. Affected host embryos die in early development. The symbiont spreads because of the decreased fitness of uninfected relative to infected female hosts [5]. The CI manipulation has been studied most extensively in *Wolbachia pipientis*, a member of the *Alphaproteobacteria* established in as many as 40% of terrestrial arthropod species [12] and in filarial nematodes [13]. The verbal model that best describes CI has been termed “modification/rescue” [14], where a factor that is important for the normal development of the insect embryo is modified in sperm cells and can be rescued only if a related strain is present in the eggs. In the fertilized oocyte of an incompatible mating of *Drosophila* or the parasitic wasp *Nasonia vitripennis*, CI *Wolbachia* leads to asynchrony of the timing of maternal and paternal chromosome condensation and segregation during the first embryonic mitotic division, disrupting embryonic development [15]–[16]. However, the molecular basis of CI in this uncultivable microbe remains largely unknown [5].

Genome analysis and expression studies of genes of diverse CI *Wolbachia* strains have revealed a number of genes with a potential role in CI [17]–[22], but our inability to cultivate these bacteria in a host-free environment, the lack of methods to genetically manipulate *Wolbachia*, and the absence of an independently evolved CI lineage with which to make comparisons has limited the progress in this area. Here we describe the genome of the only CI-inducing symbiont known that is distantly related to *Wolbachia. Cardinium hertigii* is a member of the *Bacteroidetes*, and the strain *c*Eper1 infecting the parasitic wasp *Encarsia pergandiella* causes CI [23]. The tiny parasitic wasp host (∼18 µg, 1/1000 of the weight of *Drosophila* spp.) lays eggs in whiteflies, and larval wasps develop at the whiteflies' expense, emerging as adults from the whitefly remains. Related *Cardinium* strains have also been found in the arthropod groups Hymenoptera, Hemiptera, Diptera, Protura, Acari and Araneae, and an estimated 6–7% of all arthropods are infected with these bacteria [24]–[26]. The most recent analysis also places the nematode symbiont ‘*Candidatus* Paenicardinium endonii’ within the *Cardinium* clade, and *Cardinium* as sister group to the *Acanthamoeba* endosymbiont *Amoebophilus asiaticus* [26]–[28]. The *Cardinium*/*Amoebophilus* clade is only distantly related to other known insect symbiont lineages within the *Bacteroidetes*.

The genome sequence of *Cardinium hertigii c*Eper1 reveals a highly reduced genome, both in terms of genome size and metabolic pathways, and a 58 kb cryptic plasmid. *Cardinium* encodes a set of proteins with the potential to interfere with eukaryotic cell cycle regulation. These proteins, some of which also occur in CI-inducing *Wolbachia* strains, are good candidates for effectors mediating CI. Despite its metabolically restricted genome, *Cardinium* encodes a complete biotin biosynthesis pathway, which suggests a potential role of *Cardinium* in host nutrition. Lastly, several lines of evidence suggest that protists have served as hosts for the progenitor of *Cardinium* before its adaptation to insects.

## Results/Discussion

### A highly reduced genome with features of both facultative symbionts and obligate nutritional symbionts of arthropods

**Figure 1:**
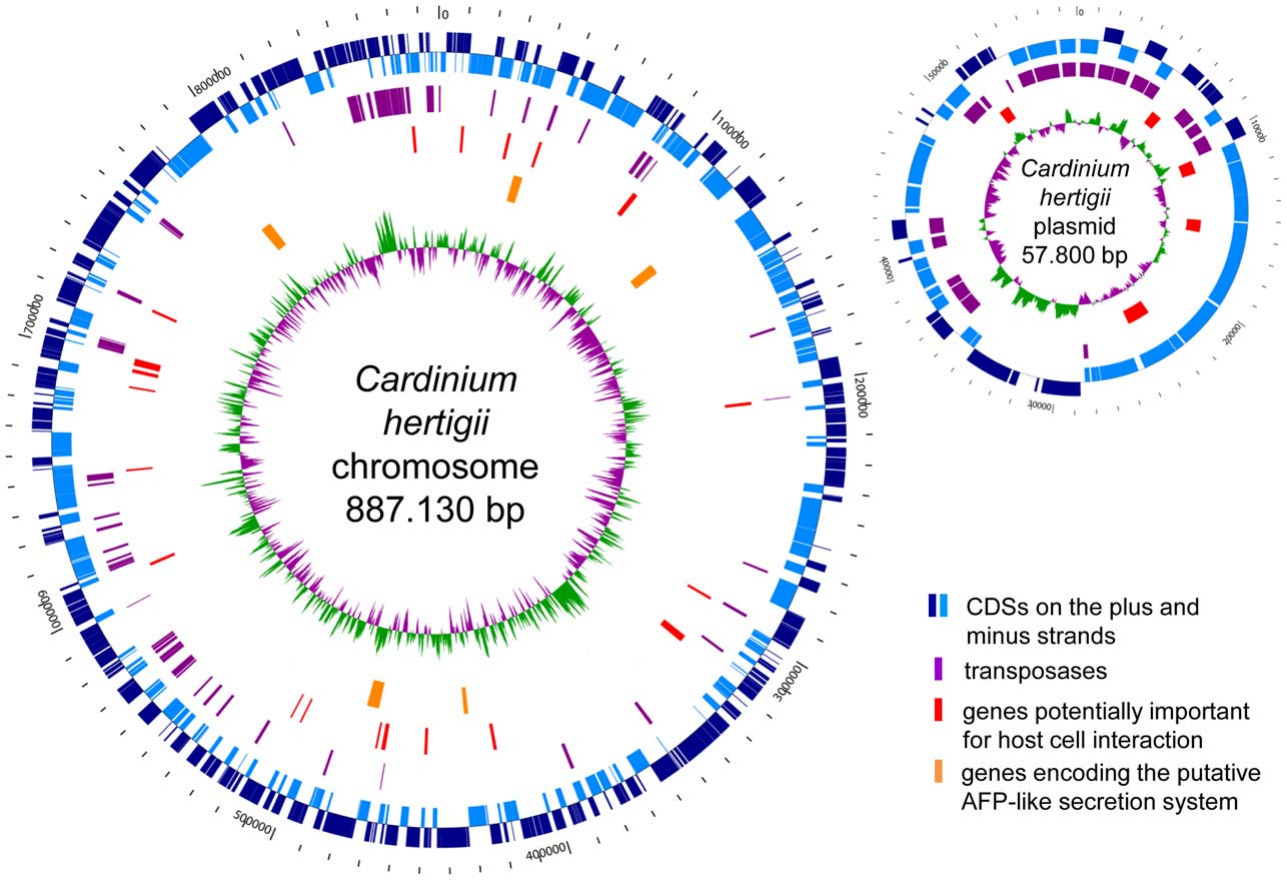
Circular maps of the *Cardinium hertigii c*Eper1 chromosome and plasmid pCher. The distribution of protein coding genes (CDSs), mobile genetic transposases, genes potentially important for host cell interaction including ankyrin repeat containing proteins, tetratricopetide repeat containing proteins and others, and the genes encoding the putative antifeeding prophage-derived secretion system is shown. The innermost green and violet circles represent the GC-skew (purple: below average, green: above average).

The genome of *Cardinium hertigii c*Eper1 consists of a single 887 kb chromosome and a 58 kb plasmid (pCher), with 841 protein coding genes (CDS) (Figure 1, Table 1). It is thus not only smaller than the genomes of free-living bacteria but also reduced compared to the genomes of the CI-inducing *Wolbachia* strains *w*Mel, *w*Ri, and *w*Pip (1.27–1.48 Mb; [20]–[22]). The size of the *Cardinium* genome is actually closer in size to the described genomes of obligate (mutualist) symbionts of diverse insect hosts, which are typically highly reduced and range from 140 kb to 790 kb (Table S1) [29]–[30]. Other genomic features of *Cardinium* such as a low G+C content (36.6%) and a single (unlinked) set of rRNA genes are also common characteristics of intracellular bacterial symbionts. *Cardinium* differs from obligate symbionts in its abundance of transposable genetic elements (n = 104; 12.4% of all CDSs; Table S2), a feature more typical of facultative symbionts, which generally show a broader host range than obligate symbionts and are not required for host reproduction [29], [31]. In addition, while some obligate insect symbionts harbor small plasmids [32], *Cardinium* possesses a large cryptic plasmid. pCher contains 65 CDSs, most of which code for transposases and proteins with unknown function (Figure 1, Table 1). Plasmids of similar size have been reported from several rickettsial symbionts infecting arthropods [33]–[35].

**Table 1:** General features of the genome of *Cardinium hertigii c*Eper1 and its closest sequenced relative *Amoebophilus asiaticus* 5a2.

	*Cardinium hertigii c*Eper1		*Amoebophilus asiaticus* 5a2
	chromosome	plasmid pCher	chromosome
size (bp)	887,130*	57,800	1,884,364
GC content (%)	36.6	31.5	35.0
CDS	841	65	1557
average CDS length (bp)	911	733	990
coding density (%)	85.5	82.1	81.8
rRNA gene set	1	-	1
tRNA genes	37	-	35
reference	this study	this study	[37]

The genome sequence of *Cardinium* contains a single gap that could not be closed due to repetitive elements (*).

**Figure 2:**
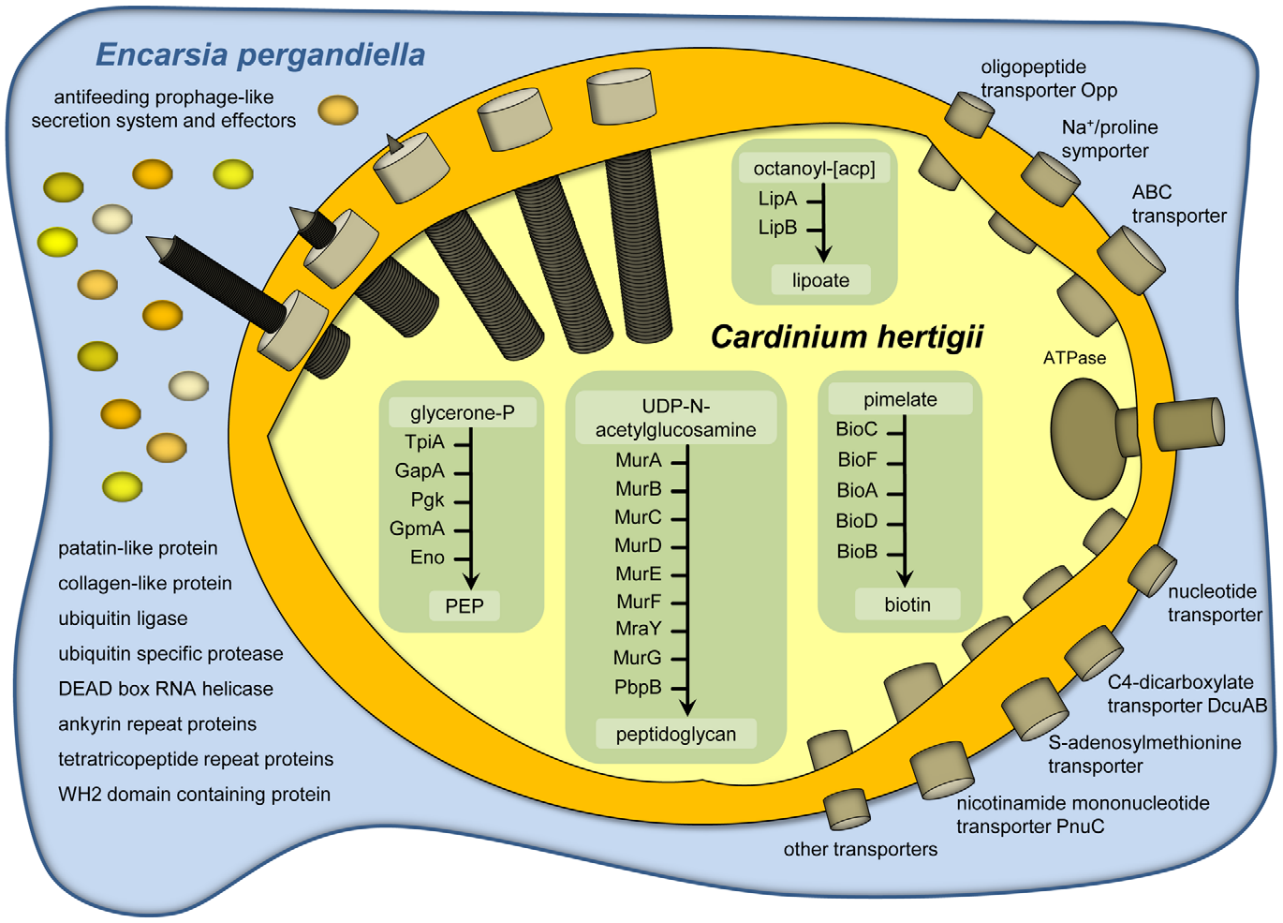
Metabolism, transport capabilities, and host cell interaction of *Cardinium hertigii c*Eper1. All predicted complete metabolic pathways and major transport proteins encoded on the genome are indicated. *Cardinium* lacks most biosynthetic pathways and imports nearly all essential metabolites from its host cell by employing a variety of transport proteins. Host cell interaction is mediated by secretion of effector proteins although no evidence for known protein secretion systems was found in the genome. A putative antifeeding prophage-derived secretion system could be used for translocation of proteins directly into the insect host cell by a contraction mechanism similar to type VI secretion systems [103].

The representation of functional categories in the *Cardinium* genome based on the assignment of CDSs to NCBI clusters of orthologous genes (COGs, [36]) is similar to that of other endosymbionts with small genomes (Figure S1). For example, the gene set required for DNA repair and recombination is similarly reduced as in other facultative symbionts. While several proteins involved in recombination are not encoded (RecBCD, RecF, RecN, RecR), *Cardinium* has retained RecA, which is missing in most obligate symbionts [32]. The presence of this and other important components suggests that homologous recombination is still possible in *Cardinium*. The biosynthetic capabilities of *Cardinium* are very limited, similar to other intracellular insect symbionts and *Cardinium*'s closest sequenced relative, *Amoebophilus* [37]. *Cardinium* is not able to synthesize most cofactors or any amino acids or nucleotides *de novo*. The tricarboxylic acid cycle is missing completely; an F-type ATPase is present but other components of a respiratory chain are lacking. Only the pay-off phase of glycolysis for the generation of ATP and NADH is present (Table S3, Figure 2). To compensate for its reduced metabolic capabilities *Cardinium* encodes 60 transport proteins (Table S4), facilitating the uptake of oligopeptides and amino acids via an oligopeptide transport system Opp A-F (CAHE_0240-0242, 0244 and 0245), ATP and other nucleotides via nucleotide transport proteins (CAHE_0018, 0158, 0160 and 0789), dicarboxylates via a C4-dicarboxylate transporter DcuAB (CAHE_0645 and 0647), and *S*-adenosylmethionine via an *S*-adenosylmethionine transporter (CAHE_0109), among others. Clearly, *Cardinium* is highly dependent on its intracellular environment and gains most key metabolites and energy in the form of ATP from its eukaryotic host cell.

### Potential role of retained biosynthetic pathways in host nutrition

Virtually the only complete biosynthetic pathways in the *Cardinium* genome are those for lipoate and biotin (Figure 2, Table S3). Lipoate is a highly conserved sulfur-containing cofactor involved in oxidative reactions, and also associated with pathogenesis and virulence of microbial pathogens [38]. Biotin is important for carboxylation reactions and cannot be synthesized by many multicelluar eukaryotes, including insects. This B-vitamin is thus an indispensable nutritional factor for insect growth and metamorphosis [39]. Vertebrate blood is deficient in B-vitamins and a complete biotin pathway is also present in the genome of a number of symbionts of blood-feeding hosts including the tsetse fly endosymbiont *Wigglesworthia* and the tick-associated *Ehrlichia*, *Anaplasma*, and *Rickettsia* species [35], [40]–[41]. It was also experimentally shown that the *Wolbachia* strain of the bedbug *Cimex lectularius* supplies various B-vitamins, including biotin, to compensate for the lack of these compounds in their insect host's blood diet [42]. The presence of the biotin pathway in *Cardinium c*Eper1 despite of the lack or truncation of almost all other metabolic pathways is puzzling given the hosts' predaceous larval lifestyle, and that antibiotic curing of *Cardinium* does not lead to obvious fitness deficits in its host [23]. This does not rule out a possible benefit of supplemental B-vitamin provision that could partially compensate for what appear to be moderately severe fecundity costs (∼15%) to *Cardinium* infection [43]. It appears reasonably common for facultative, reproductive manipulator symbionts to simultaneously confer host fitness benefits [44]–[45]. On the other hand biotin is also essential for bacteria, and in the absence of alternative sources this pathway might be equally beneficial for *Cardinium* and its host.

### A putative phage-derived protein secretion system

**Figure 3:**
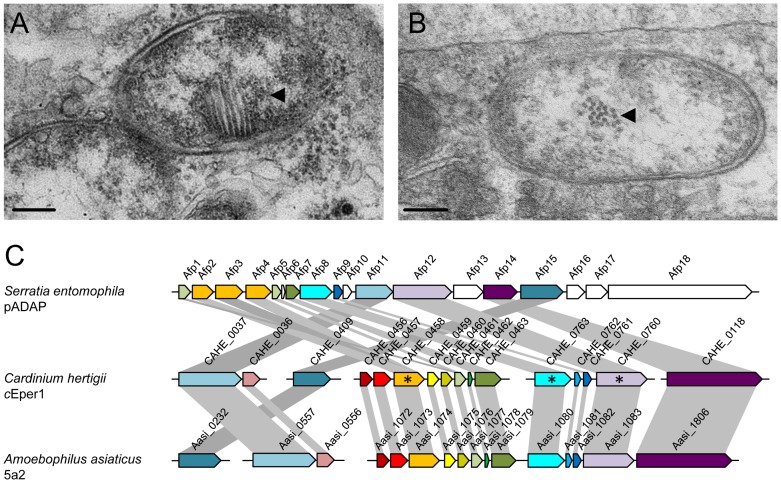
The putative phage derived protein secretion system of *Cardinium hertigii c*Eper1. Electron micrographs showing *Cardinium* in *Encarsia pergandiella* ovaries within a nurse cell (A) and a follicle cell (B), respectively. Arrows point to the antifeeding prophage (AFP) like fibril structures in longitudinal view (A) and cross section (B) representing the putative secretion system for translocation of effector proteins into the host cells; bars, 200 nm. (C) A schematic representation of the genomic organization of the AFP-like gene cluster of *Cardinium* compared to those of *Serratia entomophila* and *Amoebophilus asiaticus*. Locus tags and gene names are indicated. Homologous proteins are shown in the same color and connected with grey bars. Genes labeled with an asterisk are conserved among five different *Cardinium* strains tested by PCR (Tables S5, S6, S7).

While many obligate symbionts of insects lack dedicated protein secretion systems, several facultative symbionts, including *Wolbachia* and *Rickettsia* species, *Hamiltonella defensa* and *Sodalis glossinidius* encode protein secretion systems well known from pathogenic microbes [46]–[48]. In *Wolbachia*, a type four (IV) secretion system is likely involved in mediating CI or other effects on their insect hosts [48]–[49]. No known protein secretion system is present in the genome of *Cardinium*, but we identified 16 genes arranged in five different genome regions that show highest similarity to antifeeding prophage (AFP)-like genes recently identified in *Amoebophilus* (amino acid sequence identity between 24% and 76%; E-value< = 1e^−10^; Figure 3C; Tables S5, S6) [50]. These AFP-like genes are somewhat similar to the putative defective prophage of the entomopathogen *Serratia entomophila*, which delivers toxins into the hemocytes of its insect host [51]. AFP-like genes are encoded also in other *Bacteroidetes* [52], with the phage tail sheath protein SCFP from the algicidal bacterium *Saprospira* sp. being one of the few characterized components. This protein forms characteristic cytoplasmic fibril structures in *Saprospira* [53]. Interestingly, transmission electron microscopy shows similar subcellular structures in *Cardinium* (Figure 3A, 3B) [54]–[56], suggesting the presence of an intact protein secretion system encoded by the AFP-like genes. The *Cardinium* AFP gene cluster lacks putative toxins that are the substrates of the *Serratia* and *Photorhabdus* AFPs. Instead, the AFP-like genes of *Cardinium* may encode a more general secretion system for proteins that are important for manipulation of the insect host cell, taking over the function of the type IV secretion system found in other reproductive manipulators such as *Wolbachia*. We were able to detect by PCR the three most highly conserved AFP-like genes (CAHE_0458, 0763, 0760) in four other *Cardinium* strains from three different *Encarsia* host species (Figure 3C, Table S7), suggesting that AFP-like genes are conserved among *Cardinium* strains displaying different phenotypes and likely serve an important function. Our hypothesis of a phage-derived protein secretion system in *Cardinium* parallels the finding that the type six (VI) secretion system shares a common origin with phage tail-associated protein complexes [57]–[58].

### Candidate proteins for CI, host cell interaction, and host cell modulation

Typically, bacterial proteins for host cell interaction contain domains that are known to function in the context of a eukaryotic cell [59], including tetratricopeptide repeats (TPR), ankyrin repeats (ANK), leucine-rich repeats, and F- and U-box domains. Several *Cardinium* proteins contain characteristic TPR and ANK eukaryotic protein-protein interaction motifs (Table S8). In eukaryotic cells TPRs are often associated with multiprotein complexes and play important roles in the functioning of chaperones, transcription and protein transport complexes [60]. Proteins containing TPRs are also involved in the regulation of the eukaryotic cell cycle as components of the anaphase promoting complex (APC), a multi-subunit E3 ubiquitin ligase [61]. Proteins containing TPRs are also present in *Amoebophilus* and in CI-inducing *Wolbachia* strains, as well as in the mutualistic nematode-associated *Wolbachia* strain *w*Bm [62].

ANK proteins play important roles in a variety of cellular processes in eukaryotes such as cell cycle regulation, cytoskeleton regulation, developmental and transcriptional regulation [63]. For example, the ANK protein PLUTONIUM has an important role in the regulation of DNA replication in early *Drosophila* development [64]. ANK proteins are also known from pathogenic intracellular bacteria such as *Legionella pneumophila*, *Anaplasma phagocytophilum*, and *Coxiella burnetii*, which use type IV secretion systems to translocate these bacterial effectors into their eukaryotic host cells [65]–[66]. Notably, among bacteria, *Amoebophilus* and CI-inducing *Wolbachia* strains encode the largest number of ANK proteins (54 ANK proteins in *Amoebophilus*, 60 in *w*Pip, 35 in *w*Ri, and 23 in *w*Mel), and, while ANK proteins are virtually absent in other sequenced *Bacteroidetes* genomes and the mutualist *Wolbachia* strain *w*Bm (five ANK proteins; [62]) *Cardinium* encodes 19 ANK proteins (14 encoded on the chromosome, five on the plasmid pCher). This overrepresentation of ANK proteins in CI-inducing but only distantly related *Cardinium* and *Wolbachia* strains suggests that this class of proteins comprises important mediators of host cell interaction possibly involved in CI. Indeed, it has been frequently suggested earlier that ANK proteins could play a role in *Wolbachia* CI [22], [67], although the evidence has been equivocal [19], [68].

*Cardinium* encodes a DEAD box RNA helicase (CAHE_0677). Eukaryotic homologs of this protein promote chromosome segregation in concert with the RNA interference pathway [69]. The DEAD box RNA helicase in *Cardinium* is conserved among five different *Cardinium* strains (Table S7), and shows the greatest similarity to *Amoebophilus* and to intracellular *Alphaproteobacteria*, including *Wolbachia*. In addition, the gene encoding this protein is located in a predicted operon with a gene (CAHE_0676) coding for a cold shock DNA-binding protein that is also conserved in CI-inducing *Wolbachia* strains.

Ubiquitination is a key regulatory process specific to eukaryotes and absent in bacteria. It is thus interesting that *Cardinium* encodes a protein with a putative RING domain ubiquitin ligase activity (CAHE_p0026; Figure S2) and an ubiquitin specific protease (USP, CAHE_0028; Figure S3). USPs are effector proteins that in bacteria are known in only a few pathogens and symbionts [70]–[71]. The *Cardinium* USP is conserved among five different strains (Table S7) and belongs to the CA clan of cysteine proteases; the three key domains, the catalytic cysteine box and two histidine boxes, are highly conserved among known and functionally characterized eukaryotic USPs [72]. This high degree of sequence conservation suggests that the *Cardinium* USP functions in the context of a eukaryotic cell and is able to manipulate the host's ubiquitin system. Ubiquitin proteases are involved in stabilizing/destabilizing proteins, signaling, DNA repair, histone structure, and cell-cycle progression [70], [73]. Among other proteins, eukaryotic USPs interact with cyclin-dependent kinases (CDKs) and with CDK inhibitor proteins (CKI). CDKs are associated with DNA replication initiation in the S-phase, nuclear envelope breakdown, chromosome condensation, assembly of mitotic spindle and changes in microtubule behavior in the M-phase [74]. In CI induced by *Wolbachia*, delayed nuclear envelope breakdown and histone H3 phosphorylation of mitotic male pronuclei relative to female pronuclei indicates a delayed activity of Cdk1 in the male pronuclei of insect embryos. As a consequence, male pronuclear chromosomes do not segregate properly during mitotic anaphase [5]. Interference of bacterial effectors with CDKs is thus one way in which reproductive incompatibility could be accomplished. If *Cardinium* used a similar mechanism for induction of CI as *Wolbachia*, this could be directly achieved via secretion of the *Cardinium* encoded USP and the counteracting ubiquitin ligase. In *Wolbachia* strains, which appear to lack USPs, this could be performed through other effectors targeting host USPs, for example ANK proteins [48], [67].

Although orthologs of some of these proteins were also detected in Cardinium strains that cause other phenotypes (Table S7), they are still likely to be good candidates for CI involvement. In addition, *Cardinium* encodes a number of other more general host interaction proteins. One such protein contains a WH2 motif and a proline-rich domain at the N-terminus (CAHE_0010). These two features are commonly found in actin binding proteins, such as the Sca2 protein in *Rickettsia* of the spotted fever group, used for bacterial motility within the eukaryotic host cell [75]. Similar proteins are also present in *Wolbachia*. Other known virulence factors present in *Cardinium* include a patatin-like phospolipase (CAHE_0286) that is most similar to patatin-like proteins encoded in WO prophages in *Wolbachia* [76], and a collagen-like protein containing collagen triple helix repeats (CAHE_0706). Collagen is mainly found in multicellular eukaryotes, but is also present in pathogenic bacteria and viruses [77] and has been associated with adhesion and invasion of eukaryotic cells [78].

### Evolution from an ancestor in amoebae

*Cardinium* shares a number of genome characteristics with its closest sequenced relative, the amoeba symbiont *Amoebophilus*. Sixty-seven percent of all CDSs (n = 561) show similarity with *Amoebophilus* proteins (at least 25% sequence identity, at least 80% similarity in size). Further, their metabolic pathways are similarly truncated, encode similar transporters for the import of host-derived metabolites, and contain a notably large fraction of transposases or remnants of IS elements compared to other bacteria. The similarity of these genome features between *Cardinium* and *Amoebophilus* is striking considering the low degree of 16S rRNA sequence similarity (91%) between these symbionts, indicative of a large evolutionary distance. Consistent with its smaller size (47% relative to *Amoebophilus*) the *Cardinium* genome represents a subset of the *Amoebophilus* genome, with fewer CDSs (841 versus 1557), a greater degree of truncation of metabolic pathways (Figure S1), and the fewer functional transposase genes; 71% (74 out of 104) of the transposase genes are truncated or contain a frame shift compared to 43% in *Amoebophilus* [79]. Transposable elements are key mediators of genome plasticity; they are able to disrupt genes and to induce rearrangements such as inversions, duplications and deletions. They also play important roles in the shaping of symbiont genomes and in genome size reduction [29], [80]–[81]. The irregular genomic GC skew of *Cardinium* (Figure 1) is indicative of past activity of transposable elements. Distortion of the compositional strand bias is well known from other bacteria containing large numbers of transposases, including *Wolbachia* [20]–[22], [82]–[83]. The presence of a large proportion of transposase genes in the genomes of *Cardinium* and *Amoebophilus* is also consistent with the low degree of synteny in these relatives, indicating extensive reshuffling during the evolution of these bacteria from their last common ancestor (Figure S4).

The reduction in the capabilities of the *Cardinium* genome relative to *Amoebophilus* is also illustrated by cell wall biosynthesis. Both *Cardinium* and *Amoebophilus* are able to generate peptidoglycan, but they lack lipopolysaccharide (LPS) and show truncated phospholipid biosynthesis pathways. While *Amoebophilus* still encodes the complete MreBCD complex, RodA, and IspA considered necessary for a rod-shaped morphology [29], *Cardinium* lacks all of these genes with the exception of *mreB* (CAHE_0369) and indeed has a more coccoid appearance compared to *Amoebophilus*, a pattern also observed in other insect endosymbionts [29]. In general, the *Cardinium* genome represents a subset of the larger genome of the amoeba symbiont *Amoebophilus*. The large amount of inactivated transposase genes in the *Cardinium* genome suggests that it is undergoing further degradation and reduction.

In the *Cardinium* genome, we identified 68 genes (8% of all CDSs) that were possibly involved in past horizontal gene transfer (HGT) events (Table S9). A prominent example are the genes encoding the biotin synthesis pathway. Phylogenetic analysis suggests that *Cardinium* has originally lost all genes involved in biotin synthesis, and acquired the complete gene cluster by horizontal gene transfer, putatively from a donor related to rickettsiae (Figure S5). HGT among intracellular bacteria may occur among bacteria infecting the same hosts [84]–[86], and thus document ecological niches inhabited during the organism's evolutionary history. We used phylogenetic analysis to determine the putative HGT partners (donors or recipients) and infer additional possible hosts of the bacterial lineage leading to *Cardinium*. As expected, *Cardinium* contains a number of HGT-affected genes shared with partners generally found in arthropod hosts (38% of all HGT affected genes, Figures S6, S7; Table S9). In addition, there are many genes shared with a diverse assemblage of bacteria, and a few eukaryotic genes. Notably, 14% of the HGT-affected genes of *Cardinium* are shared with bacteria known to be associated with amoebae, e.g. *Simkania negevensis* and *Legionella drancourtii*, and 24% are shared with bacteria that have been reported to infect both amoebae and arthropods. The most likely explanation for the presence of genes from amoeba-associated bacteria is that prior to the adaptation to its arthropod host, *Cardinium* (or its ancestor) lived as a symbiont of amoebae or other protists, in which HGT with other amoeba-associated bacteria was facilitated. This notion is consistent with our observation that the *Cardinium* genome represents a subset of the genome of the sister lineage to *Cardinium*, the amoeba symbiont *Amoebophilus*. It is thus likely that the common ancestor of *Cardinium* and *Amoebophilus* lived as a symbiont of an amoeba or a protist. These unicellular eukaryotes are known to have contributed to the development of key features for survival in eukaryotic host cells by other intracellular bacteria [84], [87]–[88].

### Independent origin of CI

At some point during its evolutionary history, *Cardinium* made the transition from amoebae to insect hosts and became a reproductive manipulator able to induce CI to facilitate its spread in host populations. Although *Cardinium* and *Wolbachia* share this phenotype, it is unknown whether the molecular mechanisms leading to CI are identical. If they were, and if the ability to cause CI originated in either one of the two groups and subsequently was acquired by the other through HGT during coinfection of the same host [89]–[91], one would expect to observe a set of genes in *Cardinium* and CI-inducing *Wolbachia* that likely mediate this phenotype and share a common evolutionary origin. Among the orthologous genes shared by *Cardinium* and *Wolbachia* there is not a single obvious case of a gene encoding a candidate effector involved in CI. Apart from the patatin-like phospolipase, which is considered a more general virulence factor, we identified only one orthologous gene (CAHE_0604) that was exclusive to *Cardinium* and some rickettsiae including the CI-inducing *Wolbachia* strains. This gene encodes a predicted integral membrane protein without any known functional domains and is thus unlikely to mediate CI. This suggests that there is no common evolutionary origin of CI in *Cardinium* and *Wolbachia*, and that the molecular mechanism of CI is either different in these two groups, or convergent.

It is striking, however, that comparison of the genomes of CI-inducing *Wolbachia* strains with the CI lineage of *Cardinium* revealed in both genomes a large number of proteins that contain eukaryotic domains and likely mediate host cell interaction and CI. These include a DEAD box RNA helicase, and many ANK and TPR proteins that are highly unusual in bacterial genomes and good candidates for CI effectors manipulating the eukaryotic cell cycle. Most of these proteins are highly divergent and show no sequence similarity beyond the presence of eukaryotic domains. This indicates an independent origin of genes involved in CI, most likely through independent HGT events and acquisition of host genes. This notion is further supported by the presence of ubiquitin modifying proteins in *Cardinium*, which might be involved in CI, and the absence of these in CI-inducing *Wolbachia* strains. Taken together, CI seems to be based on the exploitation of eukaryotic domains for host cell manipulation, and there is strong evidence for an independent emergence of the molecular mechanisms underlying CI in these two groups. In general, the *Cardinium* genome points to the utility of a comparative context for analysis of reproductive manipulation in symbiotic bacteria that are refractory to direct genetic manipulation, a fertile area for research in the coming years.

## Materials and Methods

### Nomenclature of *Cardinium* strains

No strain nomenclature has previously been adopted for *Cardinium hertigii*. In an effort to create a convenient and consistent system, strains have been named in this study following the strain nomenclature of *Wolbachia pipientis* [5]. Thus the genome reference strain is “*c*Eper1”, where “*c*” refers to *Cardinium*, “Eper” refers to the host *Encarsia pergandiella*, and “1” simply denotes the first named strain from this host.

### Rearing of *Encarsia pergandiella* wasps harboring *Cardinium*

*Cardinium hertigii c*Eper1 is a symbiont of the minute parasitoid wasp *Encarsia pergandiella* (∼18 µg) that attacks whiteflies [23]. Wasps were originally collected from the whitefly *Bemisia tabaci* near Weslaco, Texas in October 2006, and kept in culture on *B. tabaci* on cowpea. Males of *E. pergandiella* develop as hyperparasites and were reared on another whitefly primary parasitoid, *Eretmocerus eremicus*. Prior to purification of *Cardinium* cells, wasps were reared on *B. tabaci* that were not infected with *Rickettsia spp*.

### Purification of *Cardinium* cells and DNA isolation

For *Cardinium* purification, wasps were reared on dozens of whitefly-infested plants. Approximately 8,000 adult wasps were collected from emergence jars. The *Cardinium* purification protocol was modified from [92]. Wasps were surface-sterilized with 2.6% sodium hypochlorite and 0.5% SDS for 1 min, washed with sterile water, and homogenized by hand in buffer A (250 mM EDTA, 35 mM Tris-HCl, 250 mM sucrose, 25 mM KCl, 10 mM MgCl_2_) using a Dounce tissue grinder (Wheaton). The homogenate was transferred to a 1.5 ml centrifuge tube with an additional 1 ml of buffer A. Cellular debris was pelleted for 5 min at 80 g, 4°C. The supernatant was centrifuged for 5 min at 4000 g, 4°C. The resulting pellet was carefully resuspended in 1 ml of buffer A, then vortexed for 3 sec. Following a 5 min centrifugation at 300 g, the supernatant was loaded onto a 13 mm diameter filter cassette holder (Swinnnex filter holder, Millipore) containing a 0.8 to 8 µm pore size glass fiber prefilter (Millipore) and a strong protein binding 5 µm pore-size mixed cellulose ester membrane (Millipore). The supernatant was slowly pushed through the filter with a syringe. The filter cassette holder was washed with buffer A (without EDTA) until 1.5 ml of filtrate was obtained. The filtrate was centrifuged for 5 min at 5000 g, 4°C. Following resuspension of the pellet in buffer A (without EDTA), 10 units of DNase 1 (Roche) were added to the cell suspension and incubated for 30 min at 4°C to remove insect host DNA. The reaction was stopped with 100 µl 0.5 M EDTA. The tube was spun down for 5 min at 4100 g, 4°C, the pellet washed with 1 ml buffer A, then spun down again. The cell pellet was resuspended in 250 µl of TE buffer.

The purified *Cardinium* cells were mixed with 675 µl of DNA extraction buffer (100 mM Tris/HCl, 100 mM EDTA, 100 mM sodium phosphate, 1.5 M NaCl, 1% cetyltrimethylammonium bromide (CTAB) (w/v), 200 µg/ml proteinase K, pH 8.0; [93]), 10 µl of 20 mg/ml proteinase K (Roche) was added and the tube was incubated for 30 min at 37°C. Then 75 µl of 20% SDS was added, the tube was shaken and incubated at 65°C for 1 h, with gentle inversions every 15 to 20 min. Following the incubation, 1 ml of chloroform/isoamyalcohol (24∶1 v/v) was mixed in. The aqueous phase was recovered following centrifugation. Nucleic acids were precipitated by adding 0.6 volumes of isopropanol, holding at room temperature for 1 h, then centrifuging at 16,000 g for 20 min, 4°C. The pellet was washed with cold 70% ethanol and spun down for 5 min at max speed, 4°C. Ethanol was removed and the pellet allowed to air dry. The DNA pellet was resuspended in TE buffer with 7 units RNase/ml (RNaseA, Qiagen), and incubated for 20 min at 37°C.

### Whole-genome amplification

The extracted *Cardinium* DNA was quantified using PicoGreen (Invitrogen), totaling approximately 2 ng, which was insufficient for library generation and sequencing, thus requiring amplification. To minimize bias, multiple displacement amplification (MDA) was performed on eight replicate reactions as follows. Approximately 0.1 ng of template DNA was denatured using alkaline solution and amplified using the Repli-g UltraFast Mini Kit (Qiagen) according to the manufacturer's instructions. MDA was performed overnight and the eight resulting MDA products were pooled prior to library generation and sequencing.

### Sequencing, assembly, and gap closure

A combination of Illumina and 454 shotgun sequencing was performed on the pooled symbiont MDA DNA product. Two differing 454 standard libraries (one un-normalized, one normalized) were generated and sequenced totaling 300,490,911 bp. In addition, we generated and sequenced two 454 paired end libraries totaling 106,933,881 bp. An Illumina GAii shotgun library was constructed and sequenced (run mode 2×76 bp) generating 1,371,155,520 bp. All general aspects of library construction and sequencing can be found at http://www.jgi.doe.gov/. The Illumina GAii sequencing data was assembled with Velvet (http://genome.cshlp.org/content/18/5/821.short) with a hash length of 61 and with the following parameters -exp_cov 130 -cov_cutoff 1 -min_contig_lgth 100. The consensus sequences were shredded into 1.5 Kbp overlapped fake reads and assembled together with the 454 data. The velvet contig fake reads (17,983 reads, 9.2 Mbp) and the 454 pyrosequencing reads (400.3 Mbp) were assembled using the Newbler assembler version 2.4 (Roche) using the parameters –ace -g –mi 98 -ml 80 –rip. The Newbler assembly consisted of 20,306 contigs in 1,154 scaffolds. Illumina reads were additionally used to correct potential base errors and increase consensus quality using the software SeqMan NGen from DNASTAR. One scaffold consisting of 78 contigs was identified as the *Cardinium* chromosome based on BLAST searches against the ribosomal rRNA database Silva (release_102); another scaffold (6 contigs) was identified representing the *Cardinium* plasmid based on BLAST searches against the non-redundant sequence dataset (nr) at GenBank/EMBL/DBBJ. The gaps in both scaffolds were closed by manual refinement of the assembly and by PCR and Sanger sequencing in house and by LGC Genomics (Berlin, Germany).

### Genome annotation and analysis

The genome was analyzed and automatically annotated using the Microbial Genome Analysis and Annotation Platform MaGe [94]. The automatic annotation was further refined by blastp against Swiss-Prot and UniProt using an E-value of 1e^−5^, a minimum amino acid identity of 30%, and minimum alignment overlaps of 40% as threshold values, and by manual annotation of selected genes. The circular view of the genome (Figure 1) was generated using the software GenVision (DNASTAR); the GC skew was calculated using the program CGView [95] with a sliding window size of 887 bp. Transposable genetic elements were identified using blastp. Data for NCBI clusters of orthologous genes (COGs, [36]) analysis were taken from the MaGe [94]. Biochemical pathway reconstruction was performed using KEGG [96] integrated in MaGe [94]. Classification of transport proteins into Transport Classification Database (TCDB) families was done using BLAST (http://www.tcdb.org/index.php) [97]. The antifeeding prophage (AFP)-like cluster was first identified by using blastp with proteins encoded on the AFP-like gene cluster of *Amoebophilus* and then by using either blastp or psi-blast with proteins of the AFP from *Serratia*. Putative host cell interaction proteins were further analyzed using blastp; protein domains were predicted using PFAM [98] and SMART [99]. Multiple amino acid sequence alignments were done using MAFFT [100]. Putative horizontal gene transfer candidate proteins were predicted by blastp of all *Cardinium* proteins against the non-redundant protein GenBank/EMBL/DDBJ sequence database (nr). *Cardinium* proteins with ten best blast hits to proteins from organisms outside the bacterial phylum *Bacteroidetes* were considered to potentially beinvolved in a past horizontal gene transfer (HGT) events. To further investigate this, the top 50 blast hits were used for amino acid sequence alignments with MUSCLE [101], and phylogenetic trees were reconstructed using the software MEGA5 [102]. Trees were calculated using the neighbor joining algorithm (2000 bootstrap resamplings) and the maximum likelihood algorithm (100 bootstrap resamplings). The nearest neighbor of putatively HGT affected genes of *Cardinium* was identified by the lowest number of internal nodes in the calculated trees. If there were several neighbors with the same number of nodes, the minimum sum of branch lengths was used as criterion. The sequences described in this paper have been deposited at GenBank/EMBL/DDBJ under accession numbers HE983995 (chromosome) and HE983996 (plasmid pCher). All contigs from the original *Encarsia* metagenome from which the *Cardinium* genome was reconstructed are also available at GenBank/EMBL/DDBJ.

### PCR screening for putative host cell interaction genes in different *Cardinium* strains

Approximately 100 wasps from five *Encarsia* spp. cultures harboring different *Cardinium hertigii* strains, including the reference strain *c*Eper1 were each collected in a 1.5 ml reaction tube, resuspended in 180 µl buffer ATL (QIAGEN DNeasy blood and tissue kit) and homogenized with a pellet pestle suitable for 1.5 ml microcentrifuge tubes. DNA from homogenized wasps was isolated with QIAGEN DNeasy blood and tissue kit as recommended in the manufacturer's protocol with the exception of the usage of 400 µg proteinase K (Roche) resuspended in 20 µl ddH_2_O instead of the proteinase K recommended by the manufacturer. A standard PCR cycling program with 35 cycles with primers specific for different *Cardinium* genes was used for the amplification (for primer sequences and annealing temperatures see Table S10). PCR included New England Biolabs Taq DNA Polymerase at a concentration of 0.8 units/20 µl reaction with ThermoPol Buffer. dNTPs were used at a final concentration of 1 mM. Primers were used at a concentration of 0.4 µM; BSA was added at 0.6 µg/µl.

### Transmission electron microscopy of *Cardinium* cells

Transmission electron microscopy of *Cardinium* cells was performed as described elsewhere [55]. Ovaries of adult *E. pergandiella* wasps were fixed in 4% glutaraldehyde in 0.05 M cacodylate buffer overnight at 4°C. After postfixation in 2% OsO_4_ for 2 h, the samples were washed, *en bloc*-stained in 2% uranyl acetate, and dehydrated through an ethanol series (50, 70, 95, and 100%). The samples were then placed in propylene oxide and embedded in Epon. Serial sections were cut with an RMC MT7000 ultra microtome. The grids were stained with saturated uranyl acetate and lead citrate and viewed under a Philips Electronic Instruments CM12 transmission electron microscope.

## Supporting Information

Figure S1 Representation of clusters of orthologous gene (COG) categories in selected genomes of obligate and facultative bacterial symbionts.
(PDF)

Figure S2 Conservation of the RING-like domain encoded in CAHE_p0026. Comparison of the domain found in CAHE_p0026 with RING-like domains showing E3 ubiquitin ligase activity according to [44]. The domains RING-HC and RING-H2 represent the two major subcategories of RING finger domains (depending on whether a Cys or His occupies the fifth coordination site); Mdm2, murine double minute 2 protein; RBQ-1, retinoblastoma binding protein 6 (RBBP6); RBX1, RING-box protein 1; Cnot4, CCR4-NOT transcription complex subunit 4. Only conserved amino acid residues indicative for the RING finger domain are shown. Cys, cysteine; His, histidine; X, any amino acid; subscript number corresponds to number of amino acid.
(PDF)

Figure S3 Multiple sequence alignment of selected ubiquitin-specific proteases (USPs) with CAHE_0028, the USP of *Cardinium hertigii*. An amino acid sequence alignment of the catalytic core domains of selected USPs is shown. The alignment was done with MAFFT [45], shading of conserved amino acid residues was performed with Boxshade available at the Swiss EMBnet server (http://www.ch.embnet.org/software/BOX_form.html). Data for important amino acid residues are taken from [46]–[47]. Amino acid residues forming the catalytic triad are highlighted in red. Amino acid residues that have been shown to be involved in van der Waals contact with ubiquitin are highlighted in green. Amino acid residues that are involved in direct inter-molecular hydrogen bond interactions using their side chains and main chains are highlighted in blue. Amino acid residues are only highlighted if they were present in all aligned sequences. Regions of high sequence conservation within characterized USPs are underlined: Cys-box (215–229), QDE-box (292–305), His-box (446–468, 477–486, 512–520); numbering according to UBP7_HUMAN residues. The consensus is displayed at the bottom of each alignment block, asterisks indicate identical positions, dots indicate similar positions. Abbreviations and accession numbers: UBP2_HUMAN (human, O75604), UBP7_HUMAN (human, Q93009), UBP14_HUMAN (human, P54578), UBP4_YEAST (*S. cerevisiae*, CAA86791), UBP8_YEAST (*S. cerevisiae*, P50102), UBP15_YEAST (*S. cerevisiae*, P50101), Aasi_0770 (*A. asiaticus*, YP_001957879), Aasi_1805 (*A. asiaticus*, YP_003573189), USP_Cardinium (*C. hertigii*, CAHE_0028).
(PDF)

Figure S4 Synteny between *Cardinium hertigii* and *Amoebophilus asiaticus*. Syntons comprising at least three genes are indicated by green lines if the orientation is conserved or by red lines in case of inversions. In total, 284 *Cardinium* CDSs are arranged in 106 syntons (larger than three genes) with *Amoebophilus*.
(PDF)

Figure S5 Phylogenetic analysis of the biotin biosynthesis cluster of *Cardinium hertigii*. Tree calculations were performed using the maximum likelihood algorithm (1000 bootstrap resamplings) with a concatenated dataset of six biotin synthesis proteins (BioB, BioF, BioH, BioC, BioD and BioH; Table S11) of bacteria from eight different phyla. Genes and their genomic organization are indicated as colored boxes. Breaks in the black bars denote noncontiguous genes. Boxes above and below the black bars indicate genes encoded on the plus and minus strand, respectively. Bootstrap values are indicated at the respective node. Note that the *Cardinium* genes are synthenic with those of the putative rickettsial donors.
(PDF)

Figure S6 HGT-affected genes in *Cardinium hertigii* and its putative donors/recipients. Only HGT candidates with a bootstrap value higher than 75% and a consistent grouping in both neighbor joining and maximum likelihood trees (shown in Figure S6) were included from the list of HGT candidate genes (Table S9).
(PDF)

Figure S7 Phylogenetic relationships of candidate HGT genes of *Cardinium hertigii*. Phylogenetic trees are based on amino acid sequences and were calculated with MEGA using the neighbor-joining algorithm (NJ) with 2000× bootstrapping and maximum-likelihood algorithm (ML) with 100× bootstrapping. Bootstrap values are indicated at the respective nodes. GenBank accession numbers are indicated.
(PDF)

Table S1 Genome sizes of selected endosymbionts. Obligate (primary) symbionts are shaded in grey; obligate symbionts are indicated with a section sign; members of the *Bacteroidetes* are indicated by an asterisk; plasmids were not taken into account.
(DOCX)

Table S2 Transposases in the genome of *Cardinium hertigii*.
(DOCX)

Table S3 *Cardinium hertigii* proteins involved in biotin biosynthesis, glycolysis, peptidoglycan biosynthesis, and lipoate biosynthesis.
(DOCX)

Table S4 Transport proteins in the genome of *Cardinium hertigii*.
(DOCX)

Table S5 Comparison of the *Amoebophilus asiaticus* AFP-like gene cluster (as query) with the *Cardinium hertigii* AFP-like gene cluster and the *Serratia entomophila* AFP on the pADAP plasmid by blast. Blast results obtained using psi-blast are labeled with an asterisk; I, amino acid identity to best blast hit; E, E-value; n.d., not determined.
(DOCX)

Table S6 Comparison of the *Serratia entomophila* AFP gene cluster (as query) with the AFP-like gene cluster of *Cardinium hertigii* and *Amoebophilus asiaticus* by blastp. I, amino acid identity to best blast hit; E, E-value; n.d., not determined.
(DOCX)

Table S7 Phenotypes of different *Cardinium hertigii* strains, their *Encarsia* wasp hosts, and presence of selected genes detected by PCR. CI (cytoplasmic incompatibility inducing), PI (parthenogensis inducing).
(DOCX)

Table S8 Proteins of *Cardinium hertigii* likely involved in host cell interaction. I, amino acid identity to best blast hit; E, E-value; n.a., not applicable; n.p., not present.
(DOCX)

Table S9 *Cardinium hertigii* genes putatively involved in past horizontal gene transfer events. Nearest neighbors in phylogenetic trees are indicated (neighbor-joining trees, 2000 bootstrap replications; maximum-likelihood trees, 100 bootstrap replications; Figure S6). Genes encoding transposases, repeat proteins and Na^+^/proline symporters, and genes shared with *Amoebophilus* are not listed. AM, amoeba associated bacteria; AA, *Rickettsia* that are able to multiply in amoebae and arthropods; ART, arthropod associated bacteria; E, eukaryotes; X, other bacteria; n.a., not applicable. Nodes with a bootstrap higher than 75 and the same group are indicated with an asterisk.
(DOCX)

Table S10 Primers used for the detection of putative host cell interaction genes in different *Cardinium hertigii* strains (Table S7).
(DOCX)

Table S11 NCBI accession numbers of proteins from the biotin biosynthesis pathway used for a concatenated data set for the calculation of a phylogenetic tree with the maximum likelihood algorithm.
(DOCX)
