# Supplementary figures and images for: Comparative Genomics Suggests an Independent Origin of Cytoplasmic Incompatibility in Cardinium hertigii
Source: PLoS Genet. 2012 Oct 25;8(10):e1003012. doi: 10.1371/journal.pgen.1003012 (PMC3486910; doi:10.1371/journal.pgen.1003012)

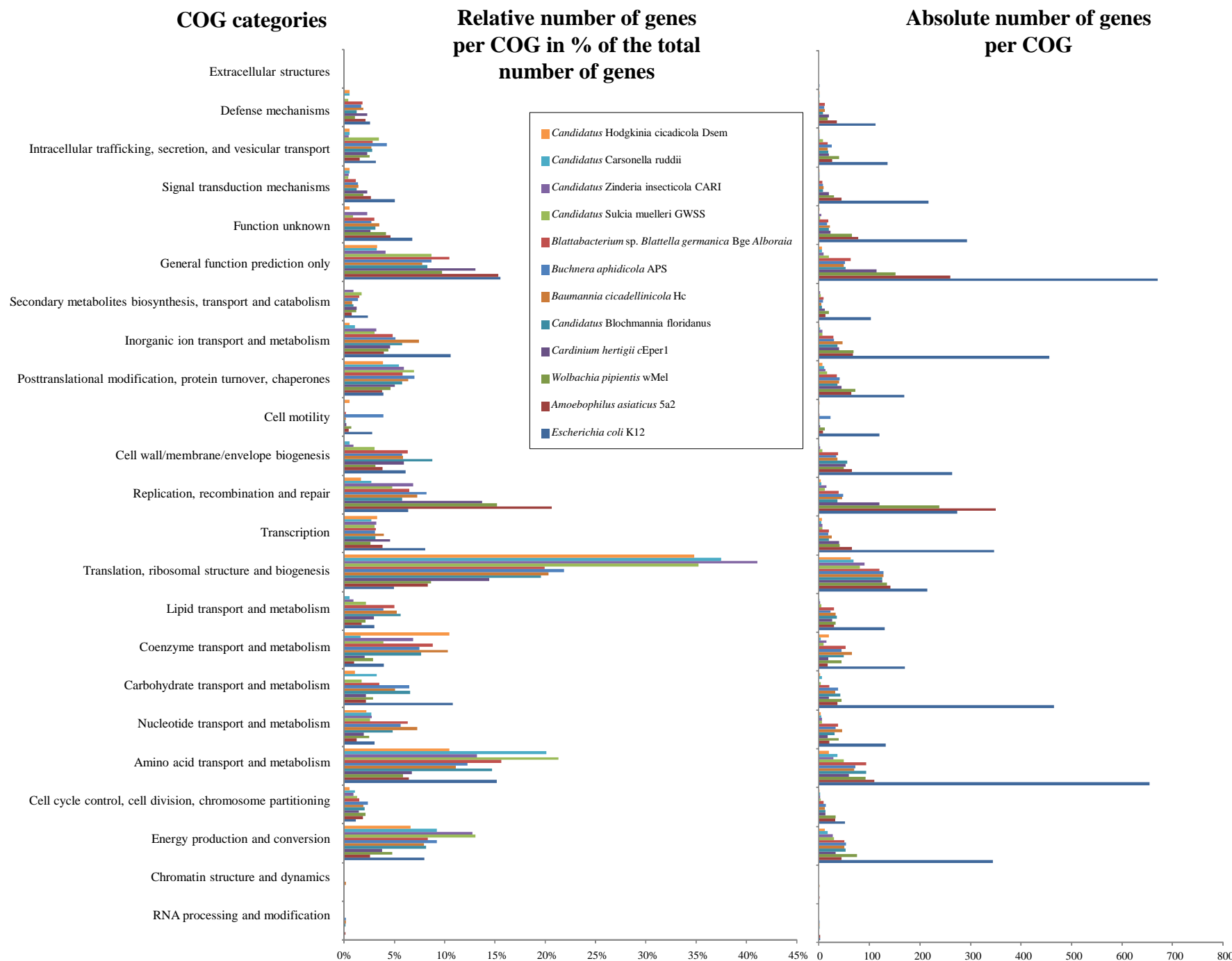

Supplement: Figure S1 — Representation of clusters of orthologous gene (COG) categories in selected genomes of obligate and facultative bacterial symbionts. (PDF) [file pgen.1003012.s001.pdf]

*Cardinium hertigii* cEper1 genome (887 kbp)

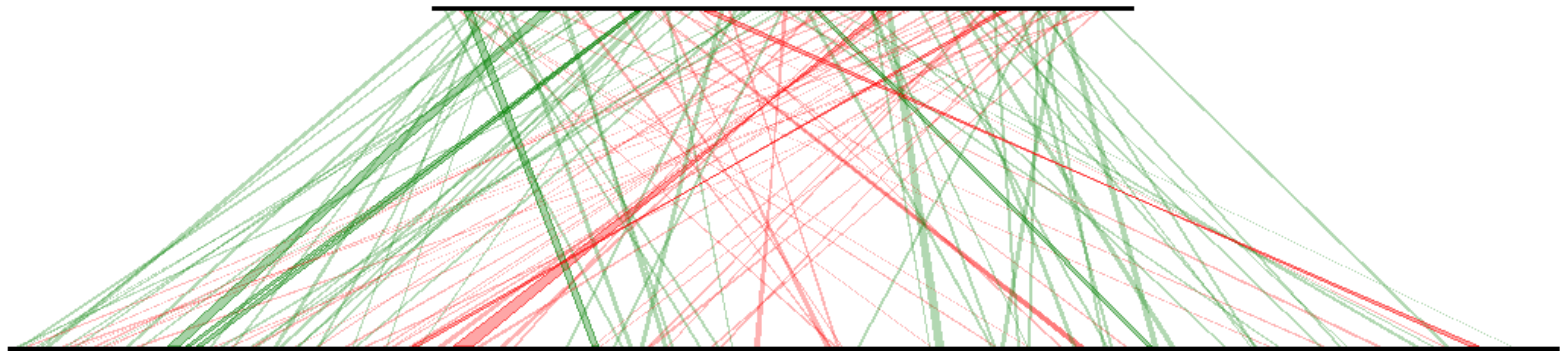

*Amoebophilus asiaticus* 5a2 genome (1,884 kbp)

Supplement: Figure S4 — Synteny between Cardinium hertigii and Amoebophilus asiaticus. Syntons comprising at least three genes are indicated by green lines if the orientation is conserved or by red lines in case of inversions. In total, 284 Cardinium CDSs are arranged in 106 syntons (larger than three genes) with Amoebophilus. (PDF) [file pgen.1003012.s004.pdf]

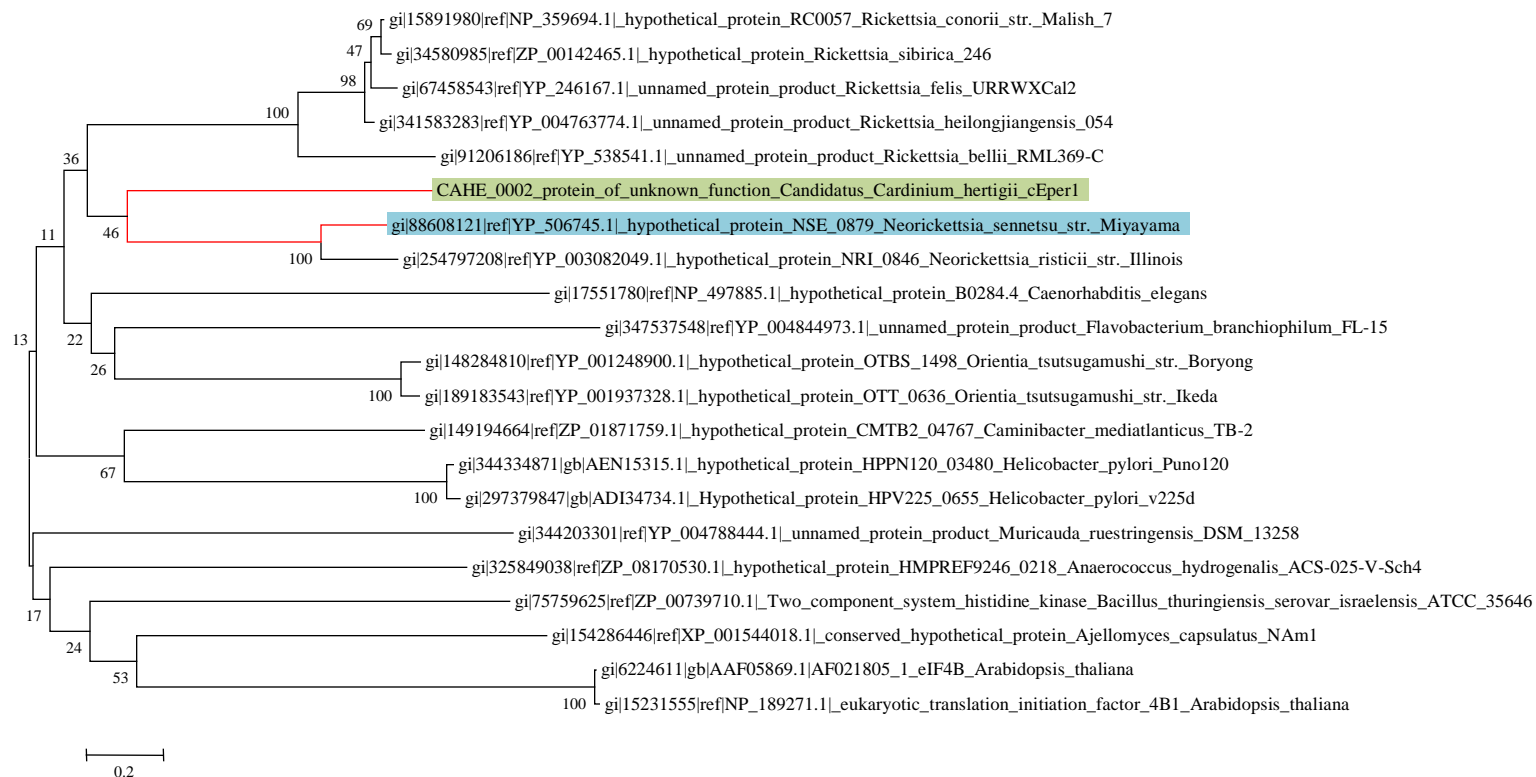

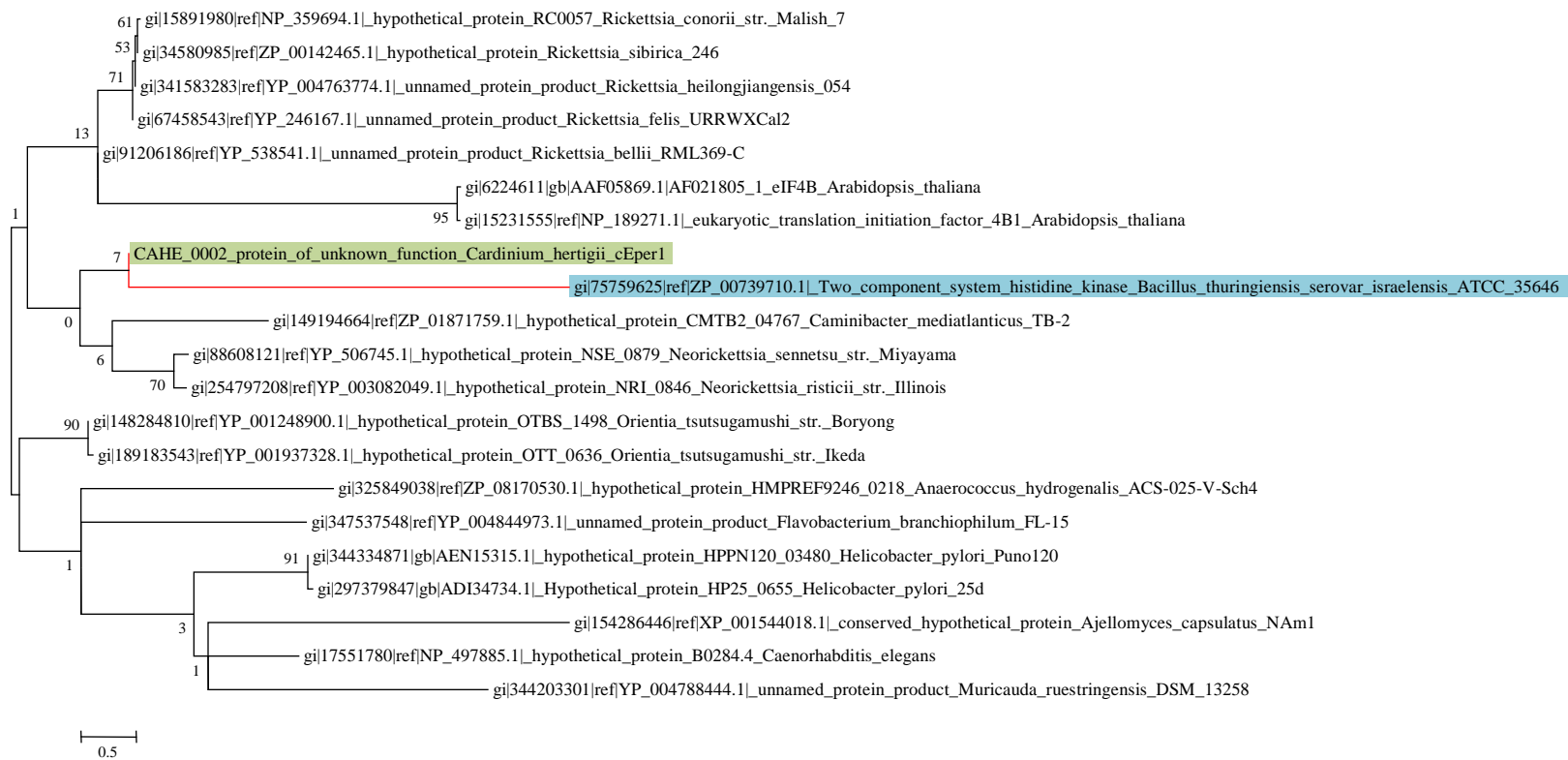

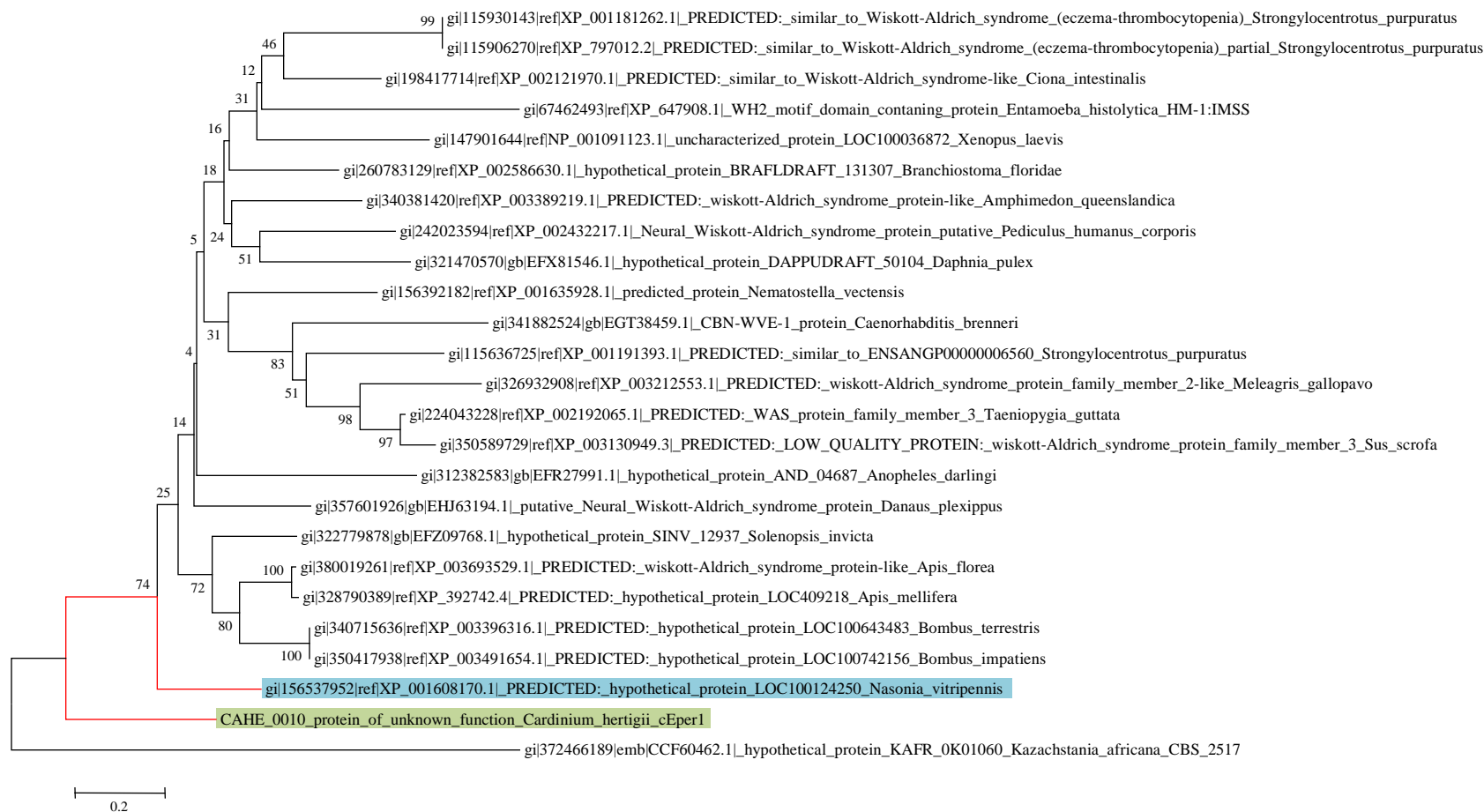

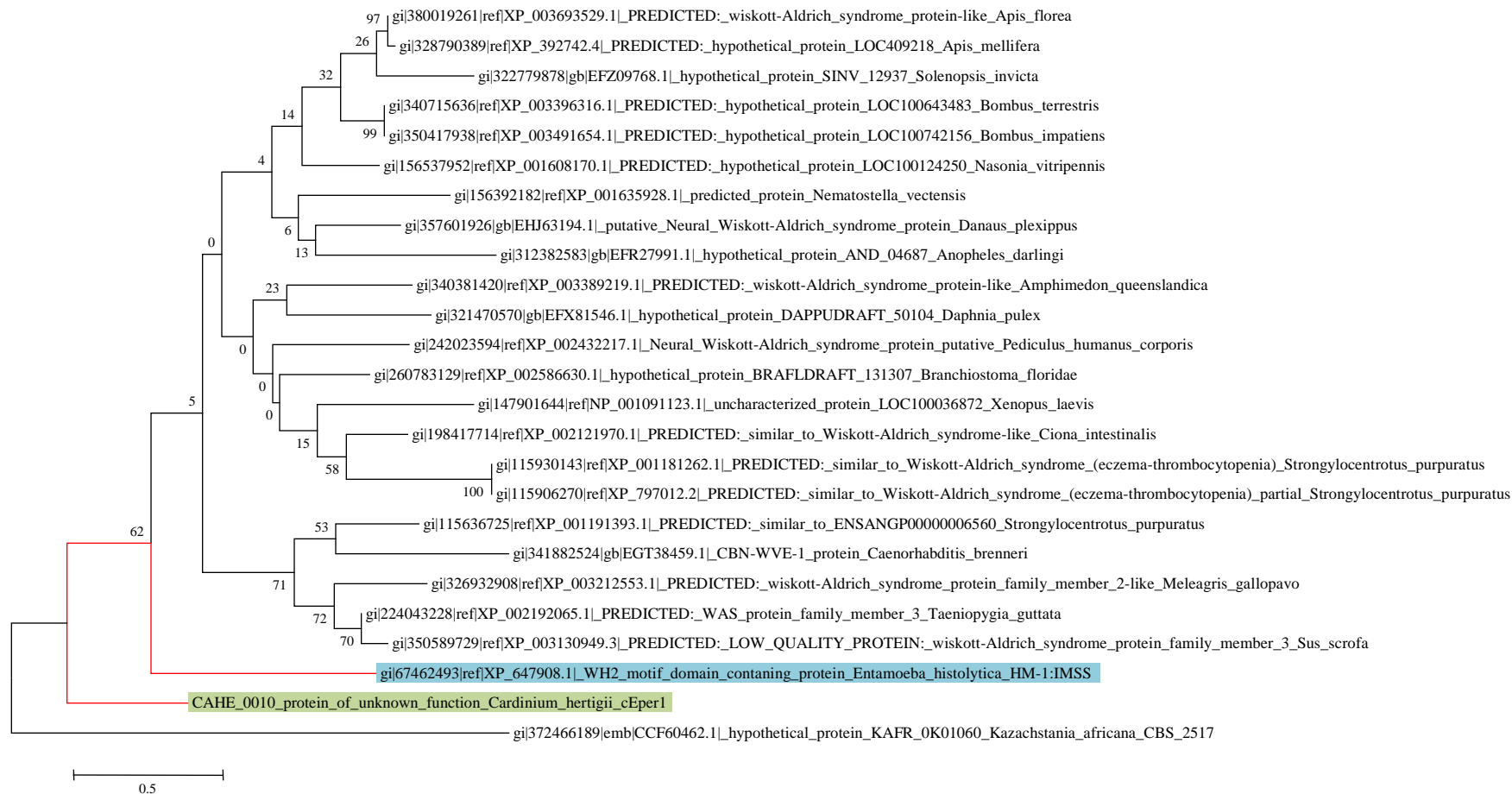

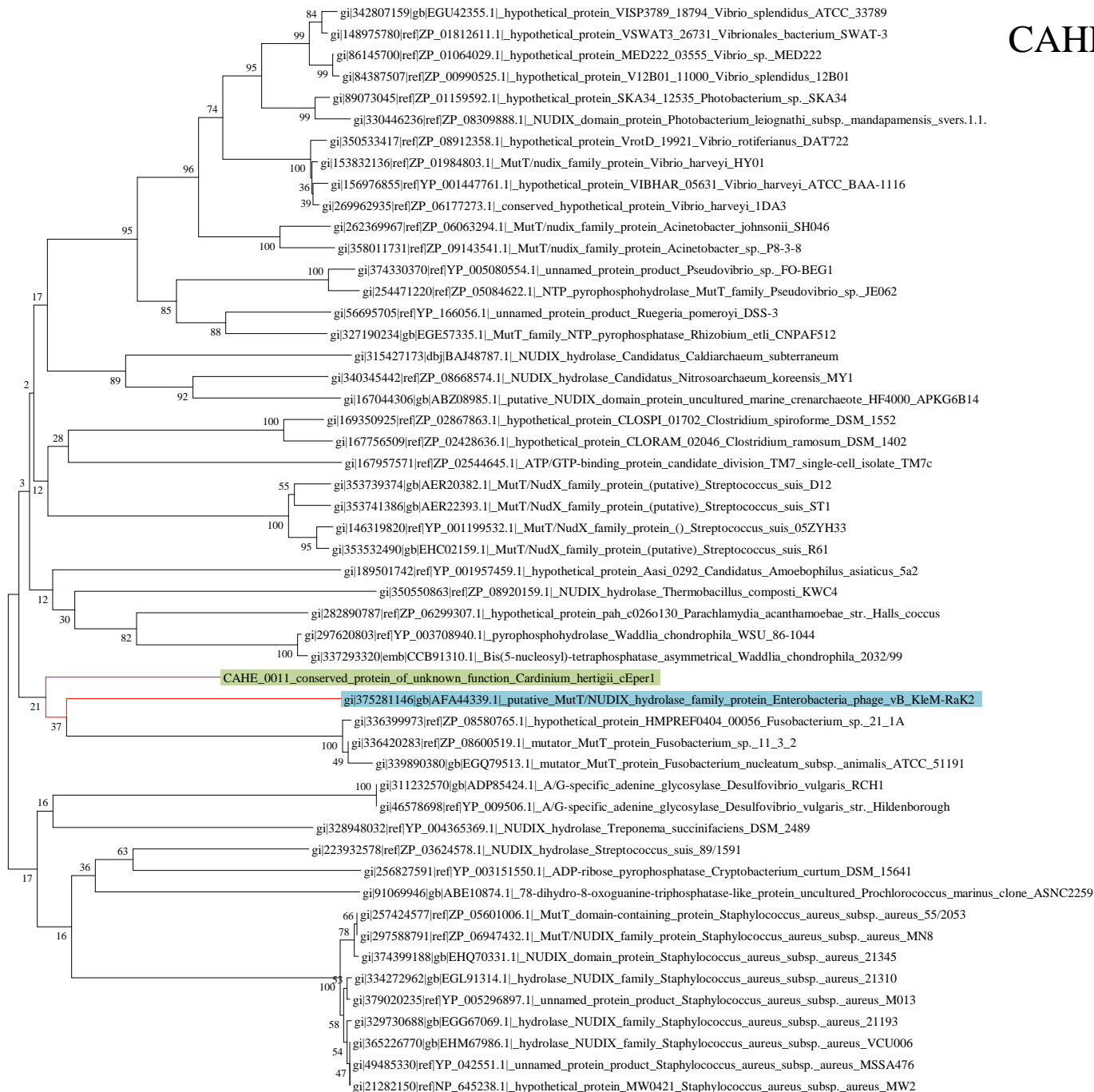

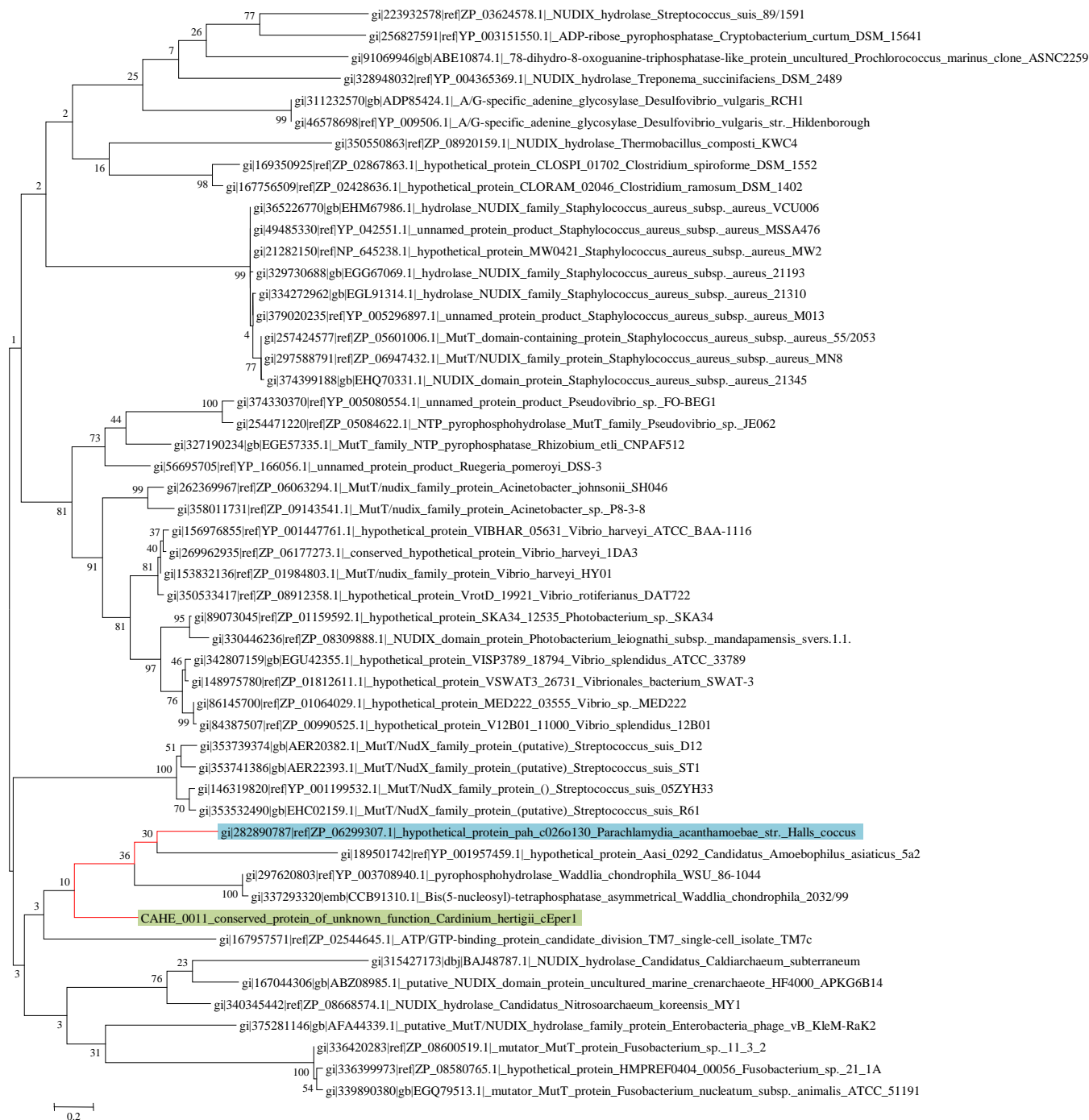

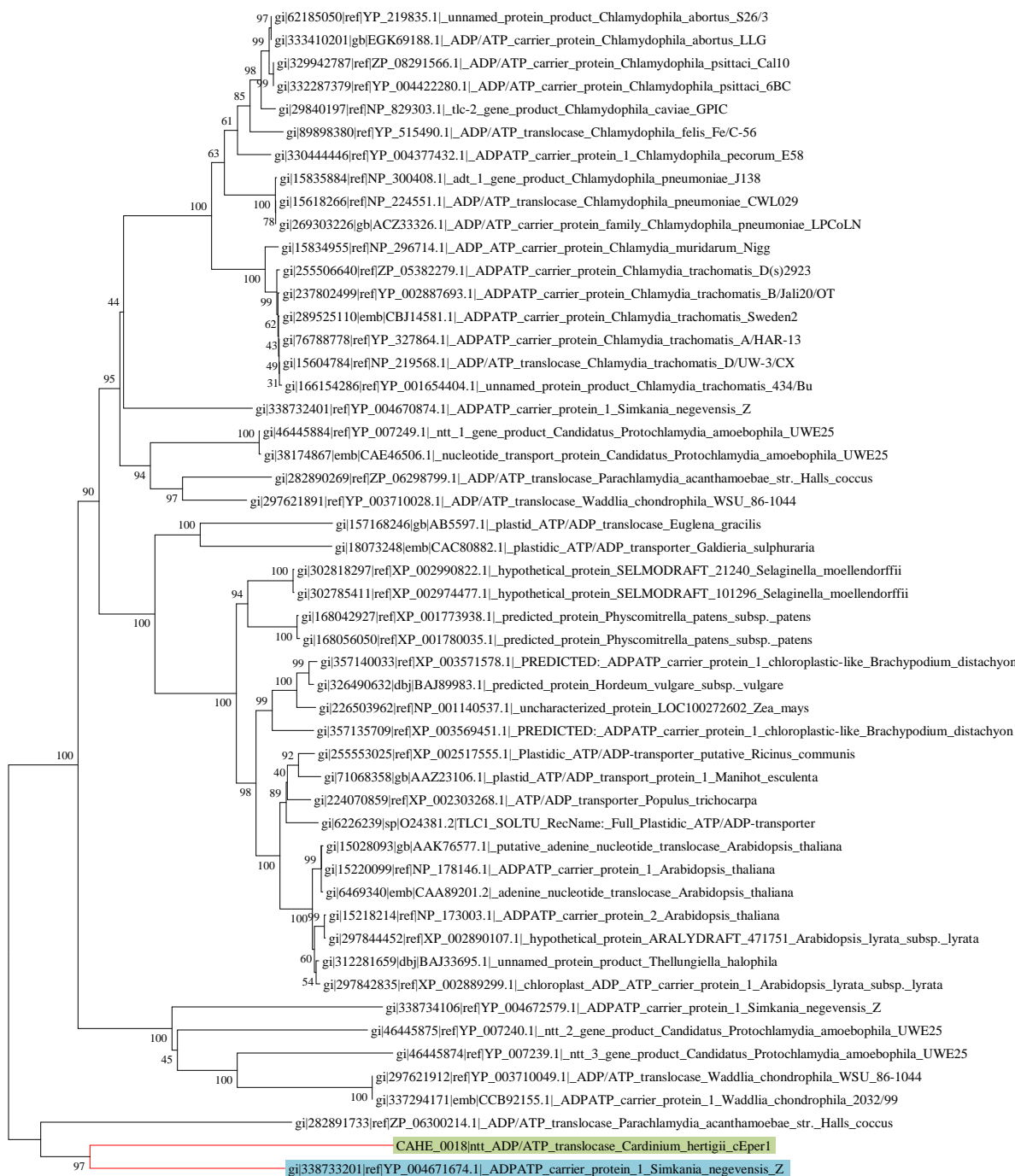

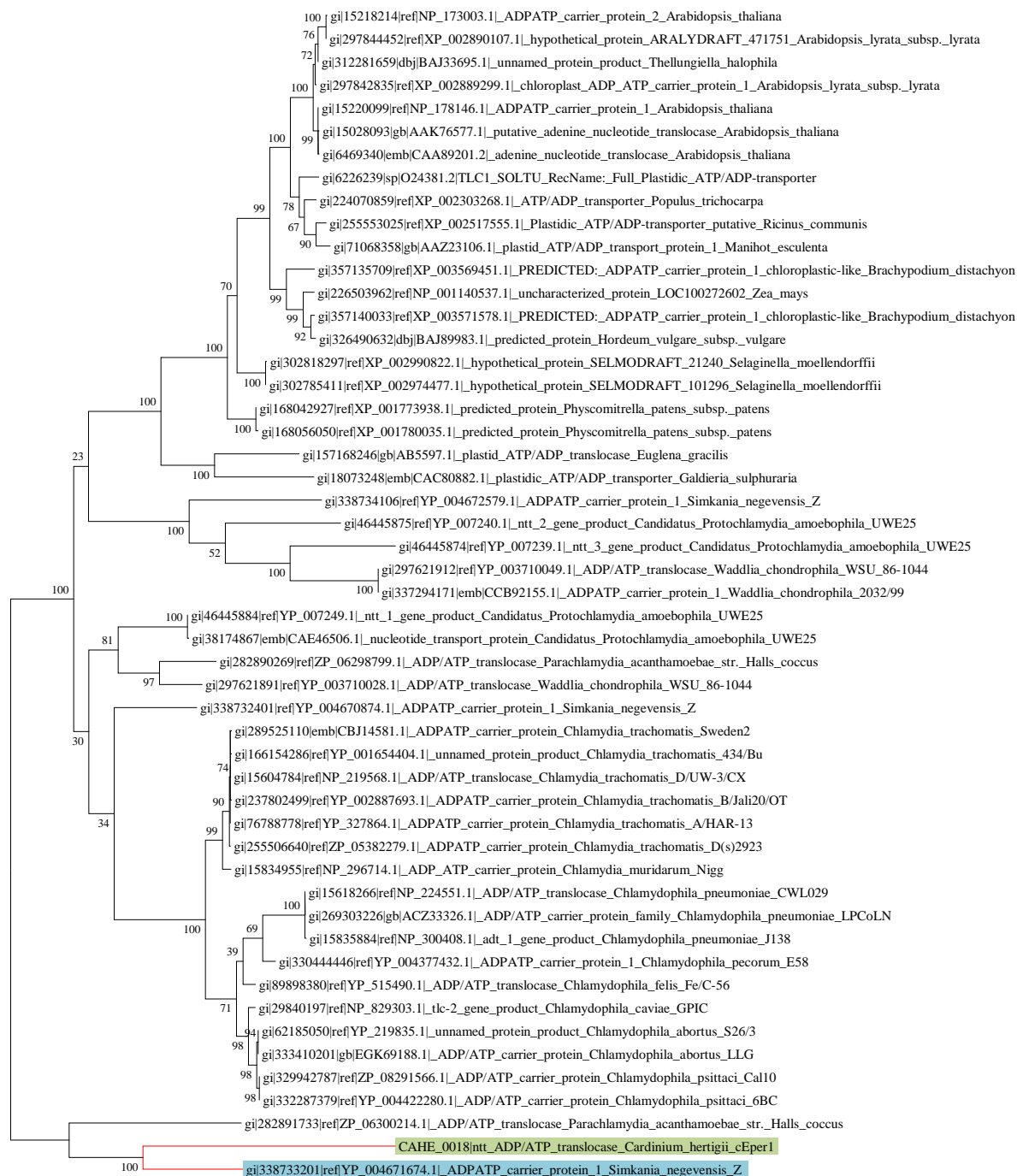

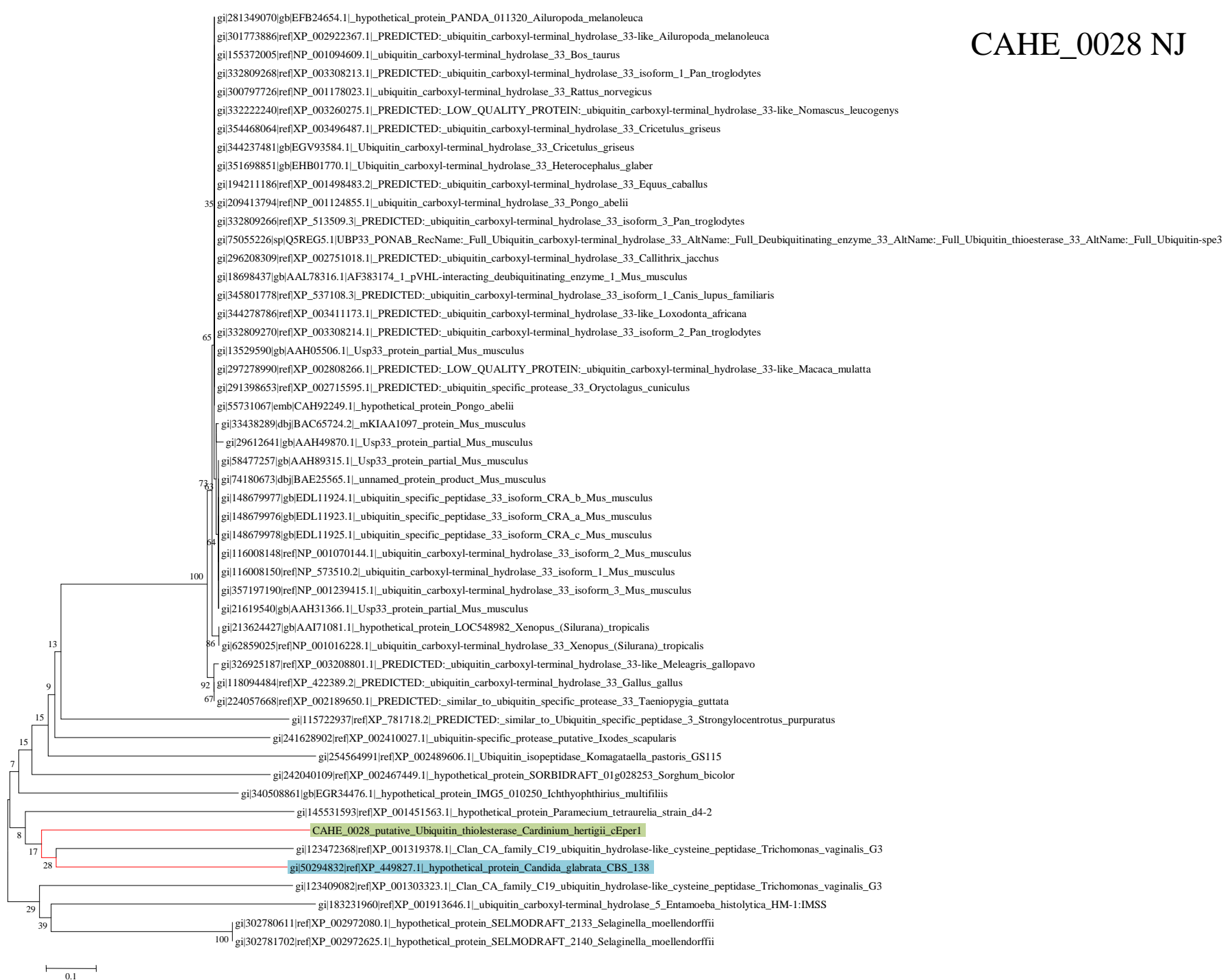

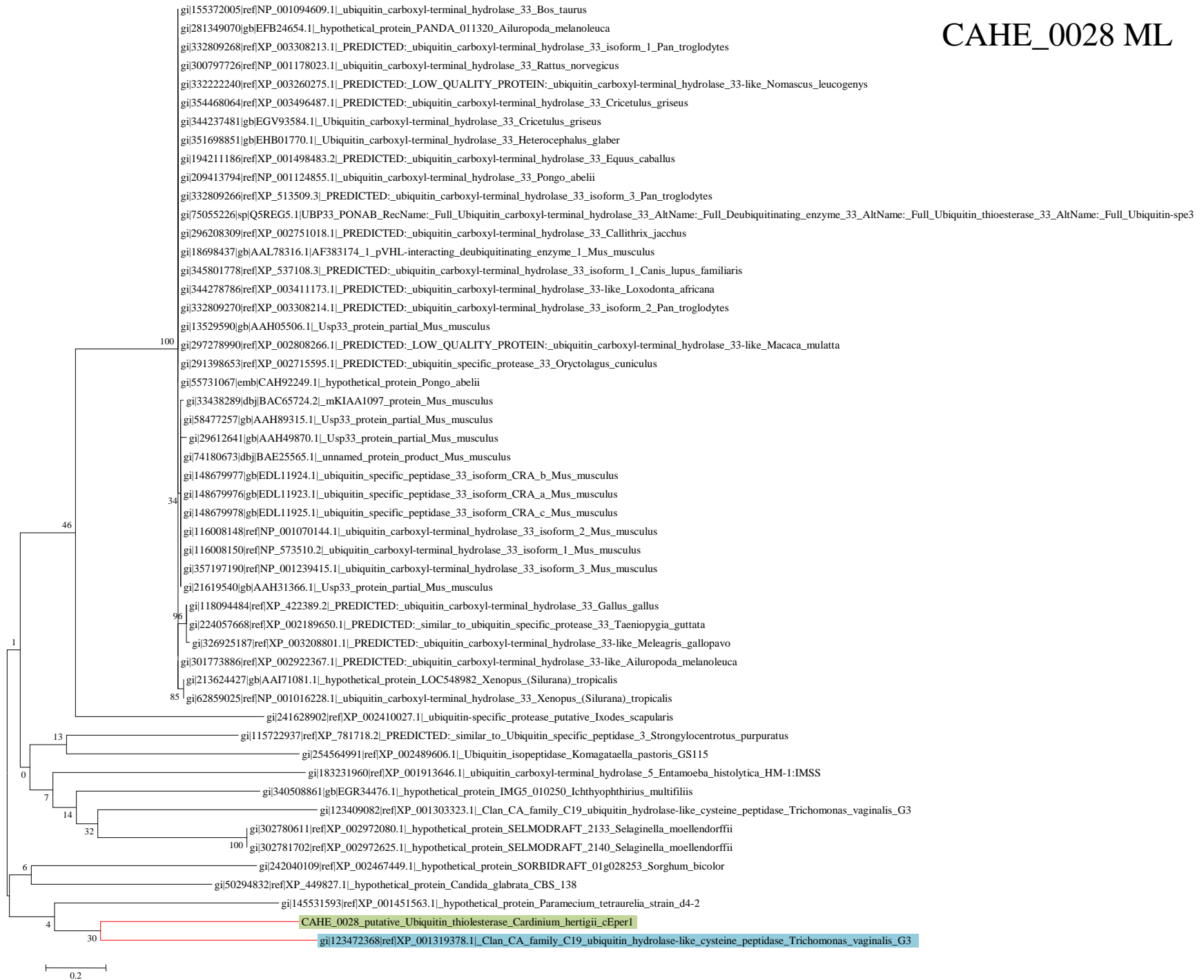

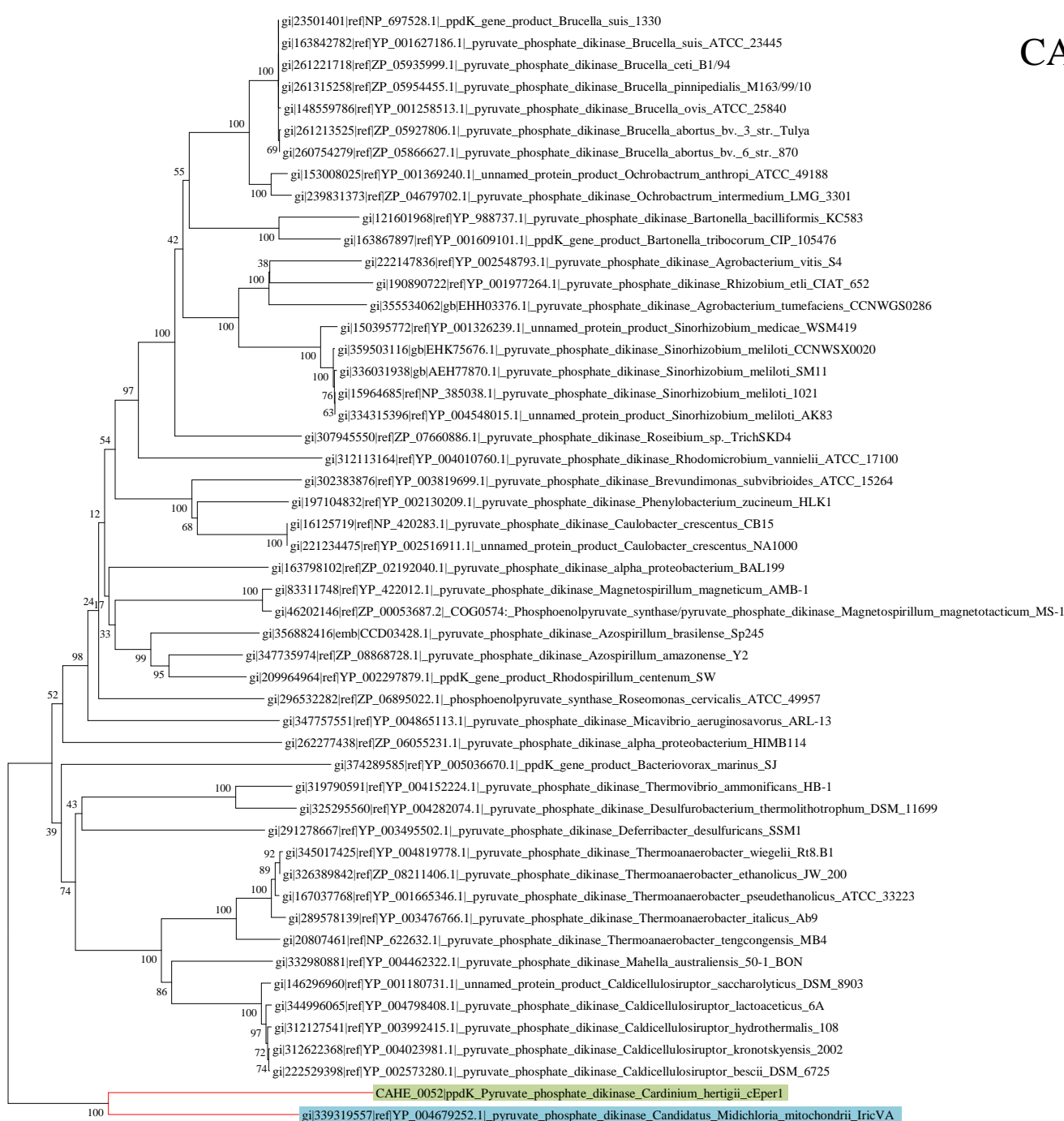

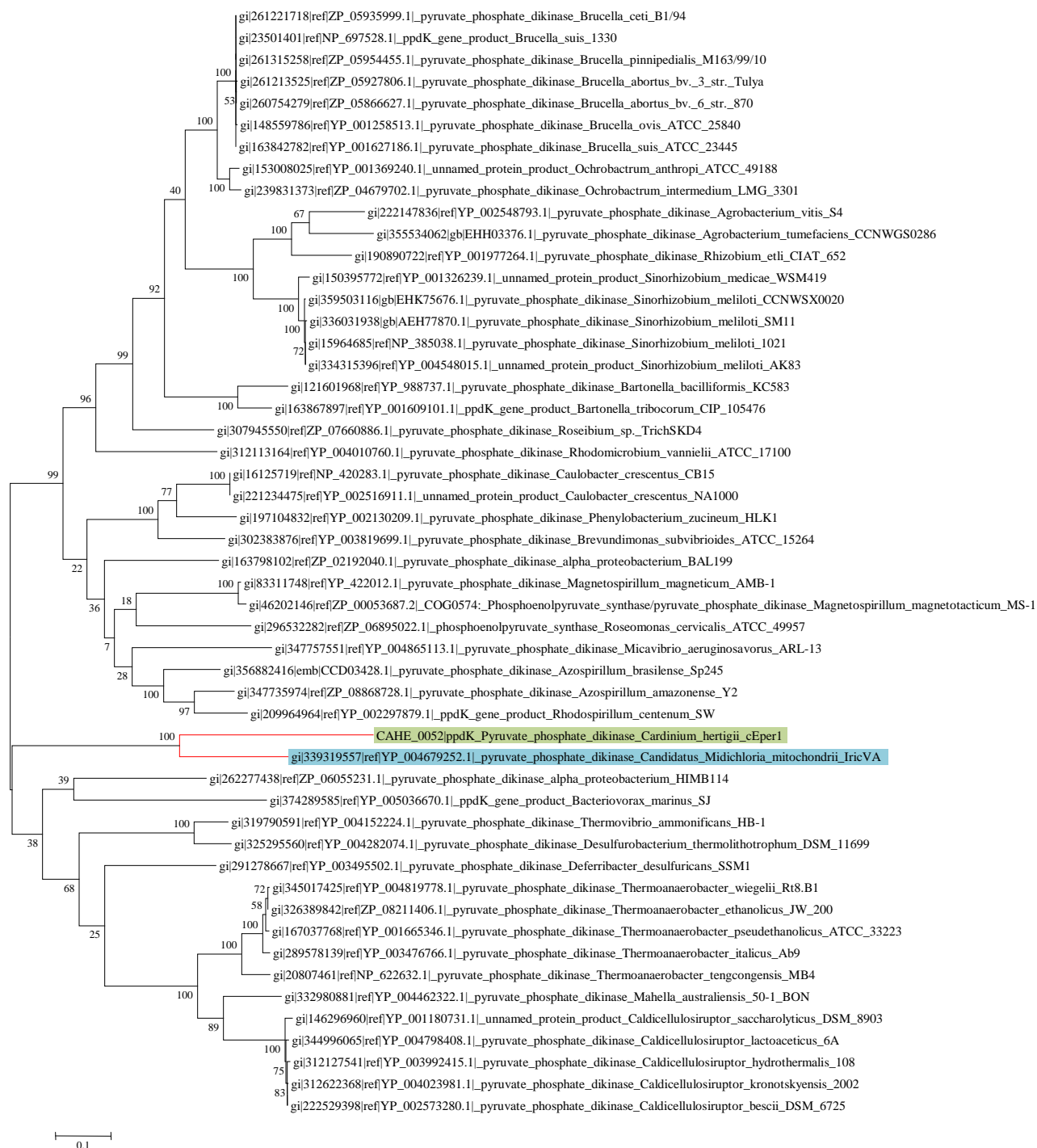

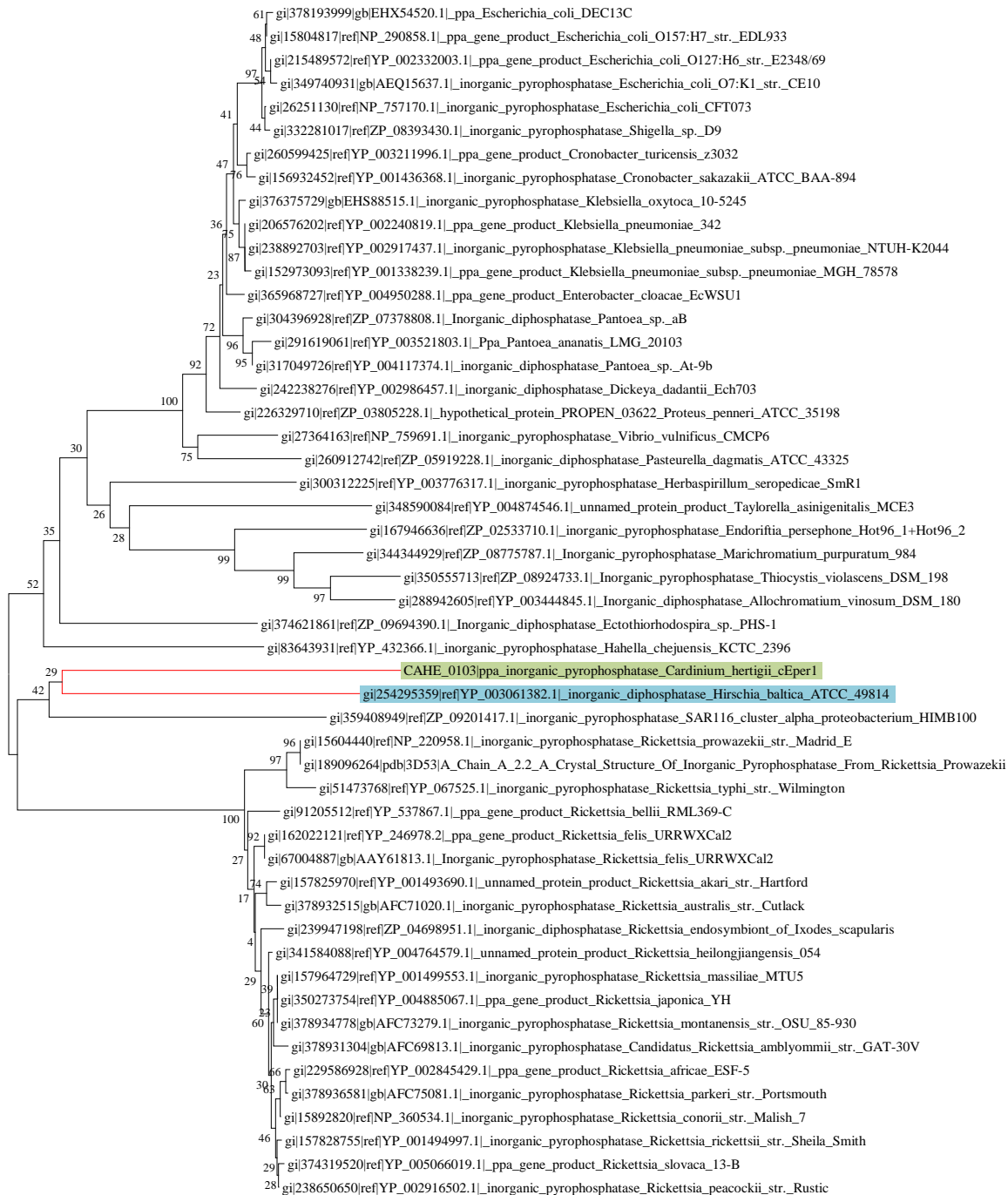

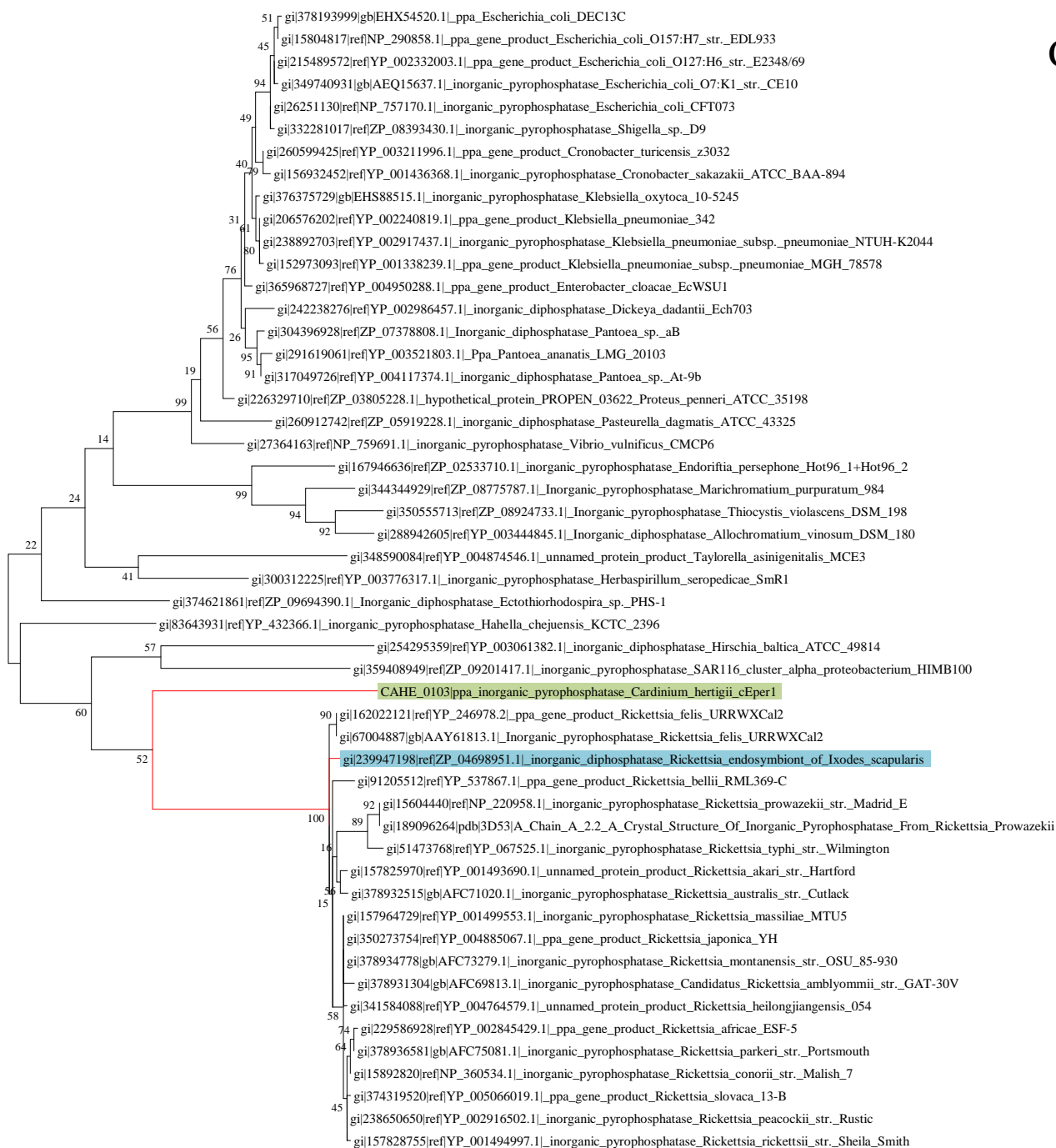

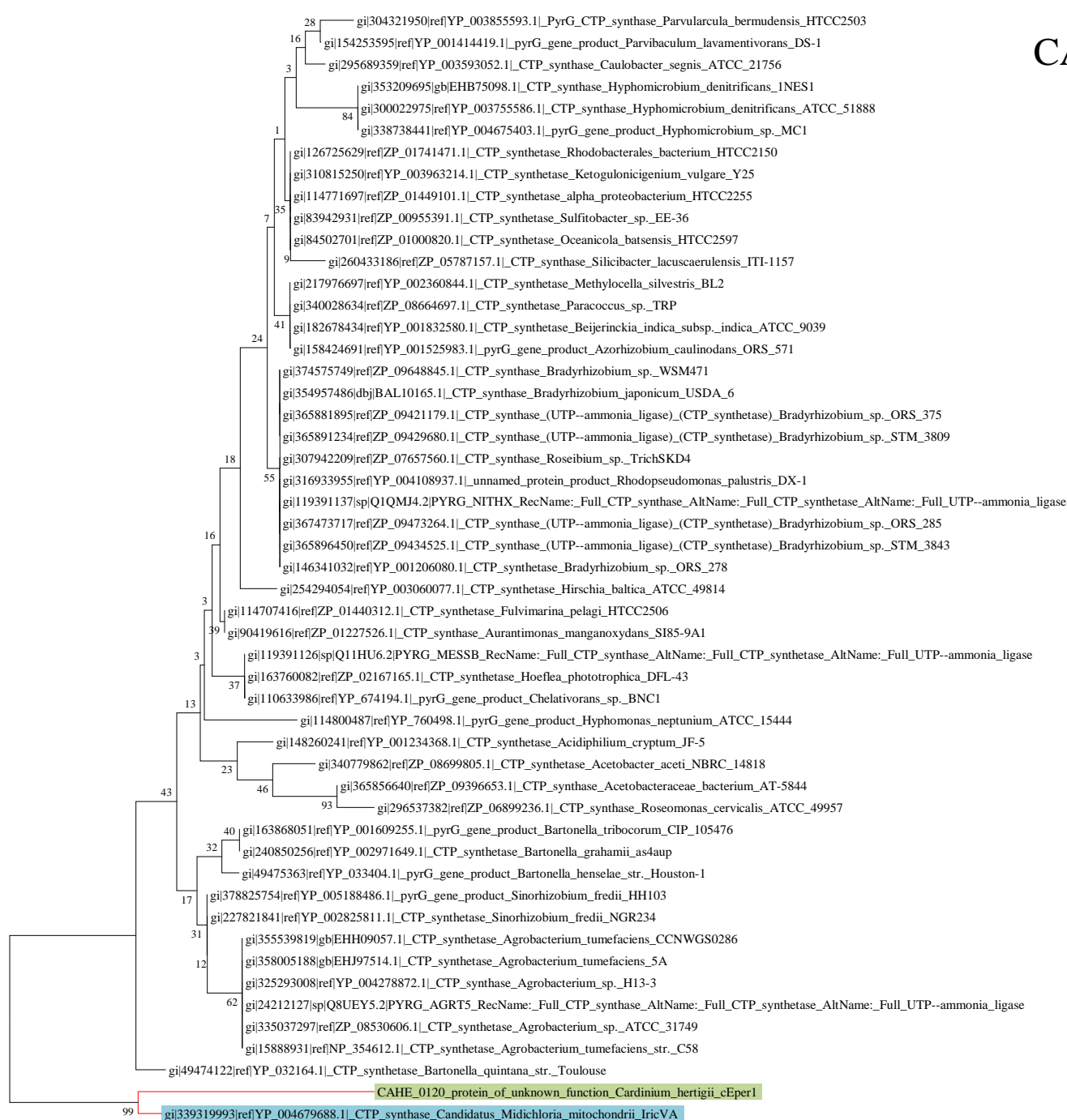

# CAHE\_0120 ML

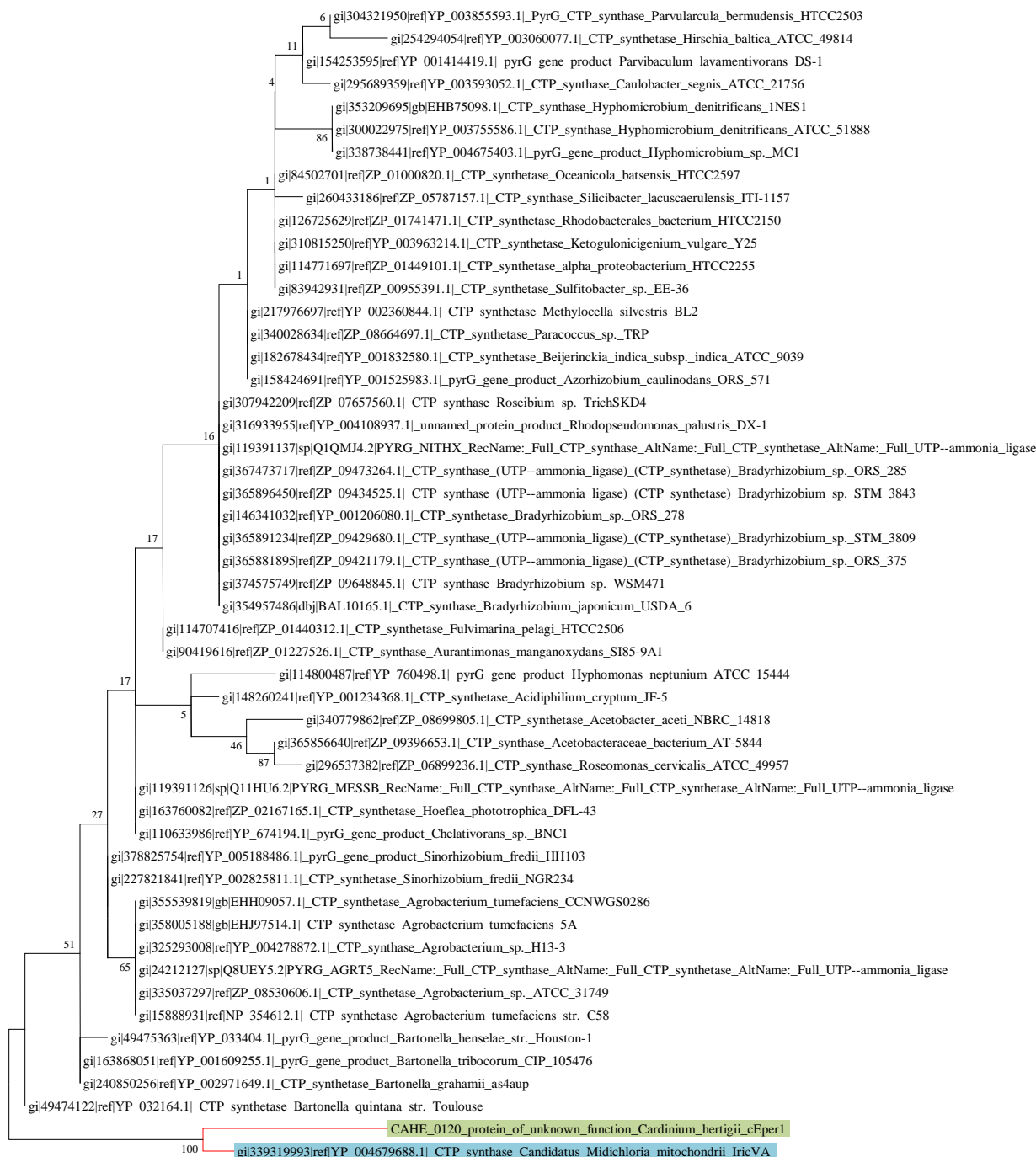

0.05

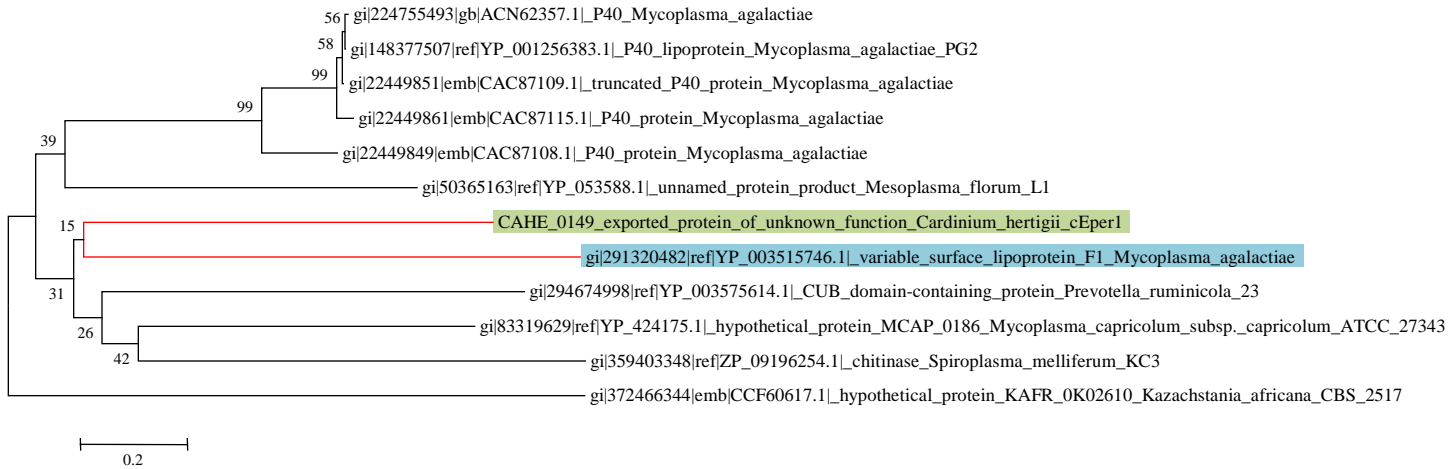

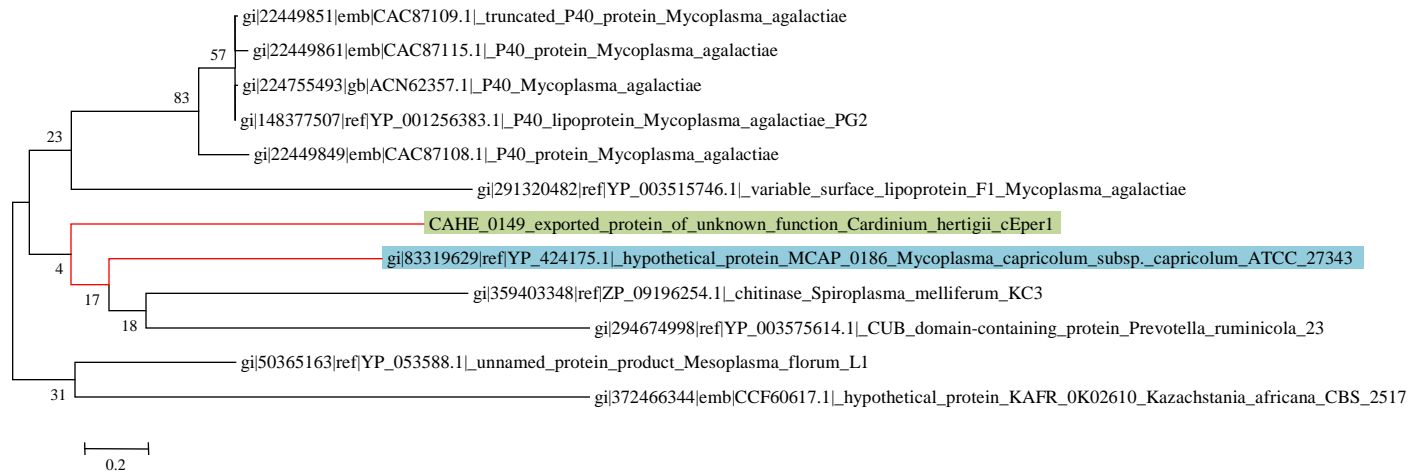

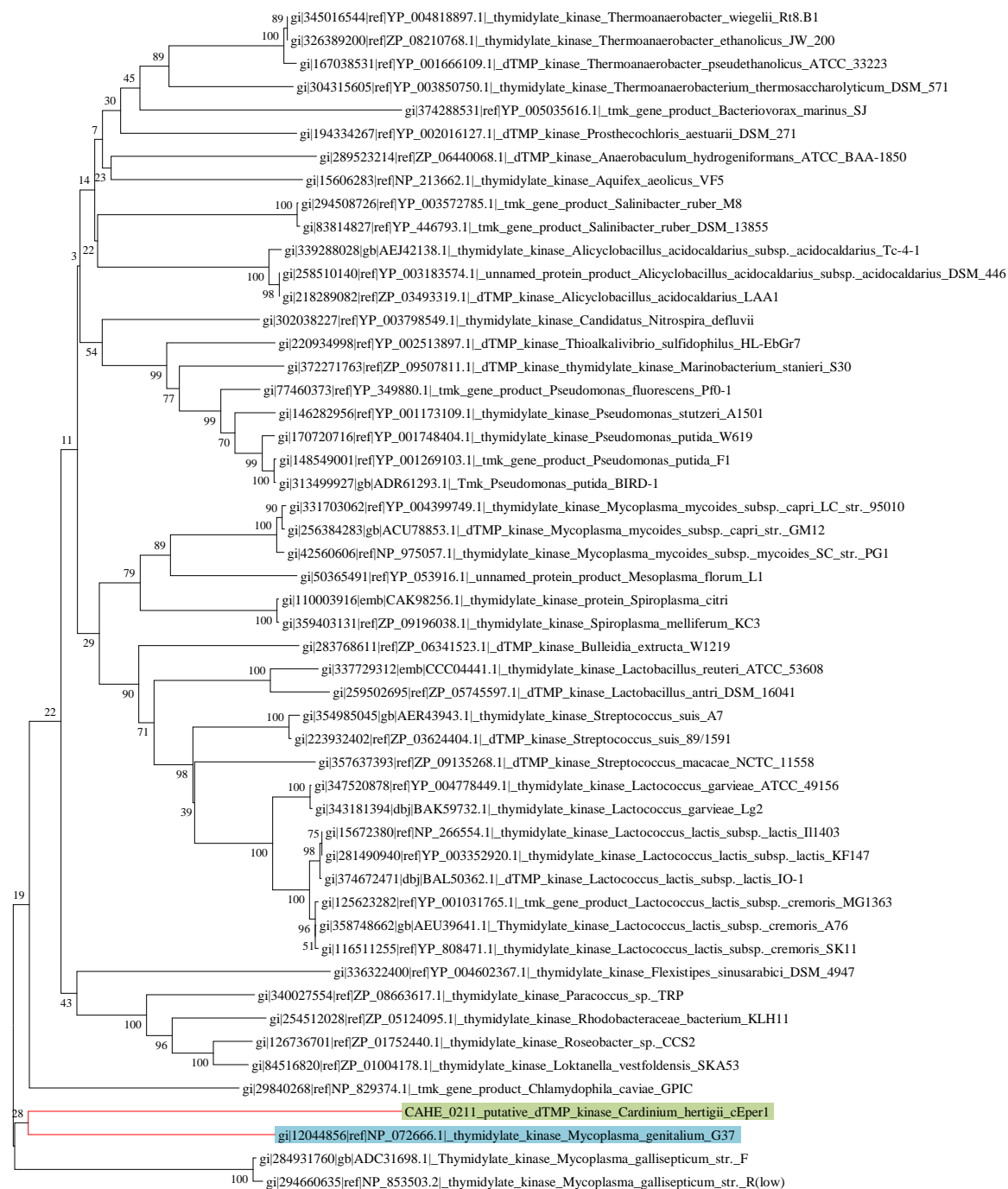

0.1

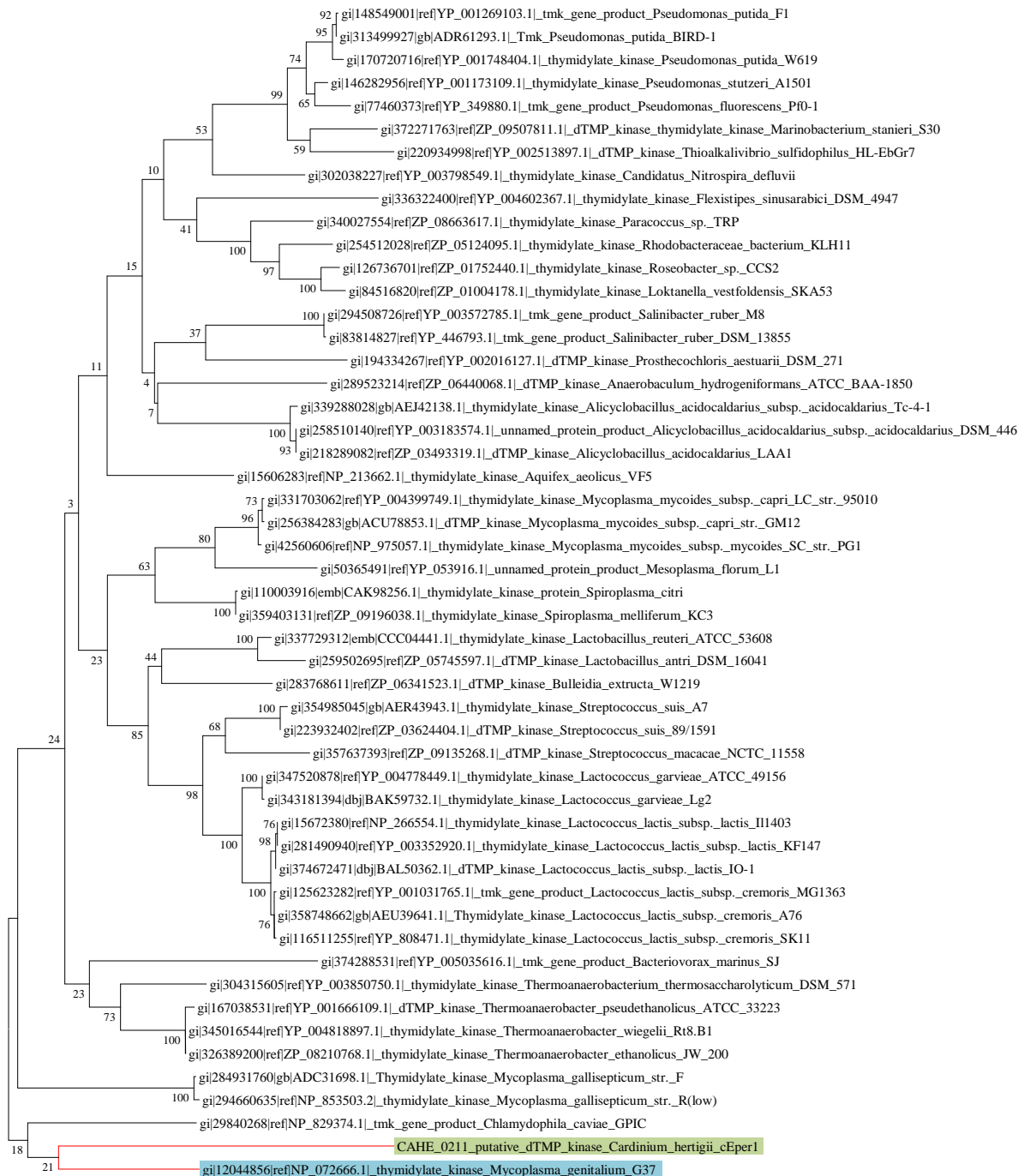

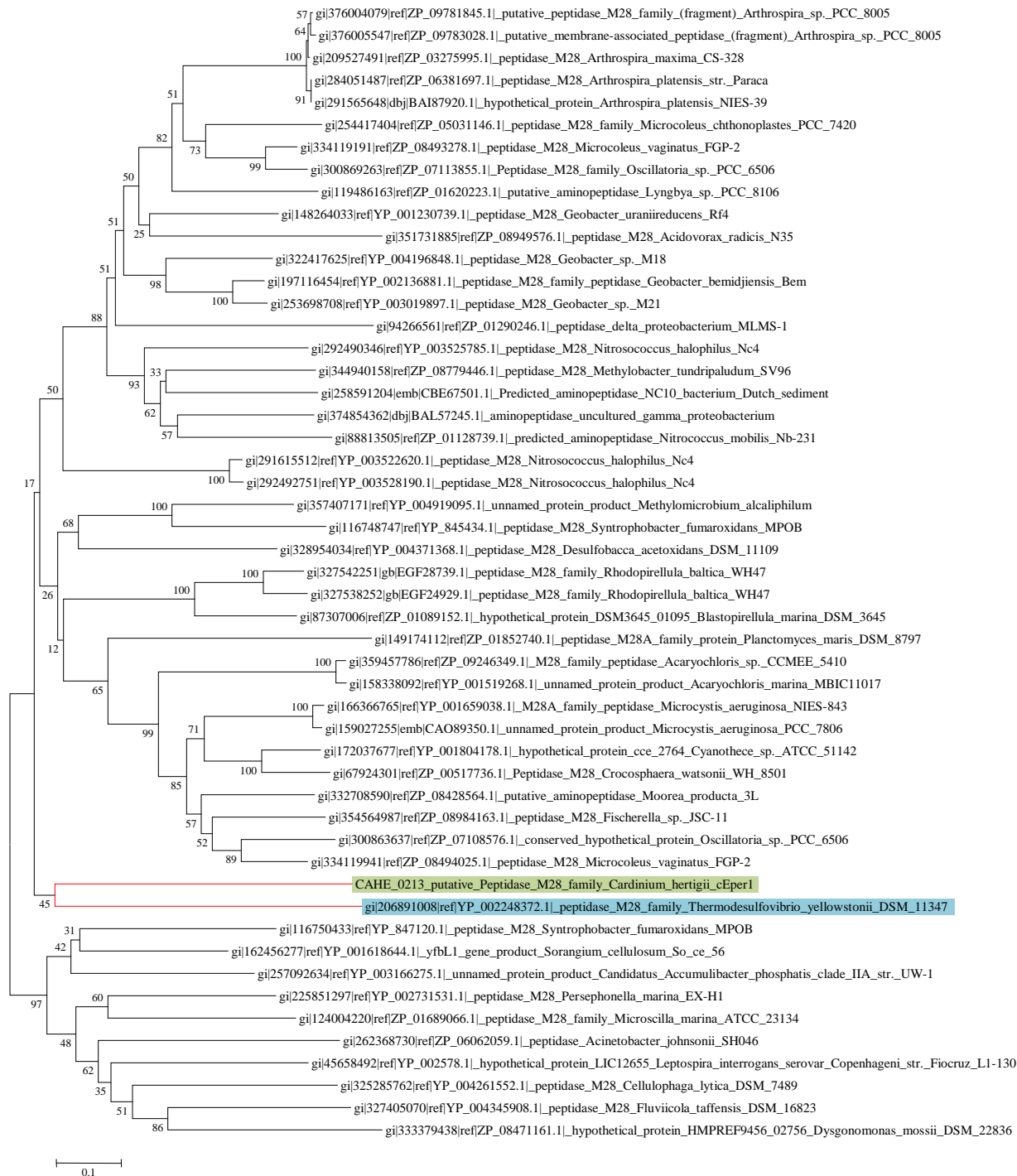

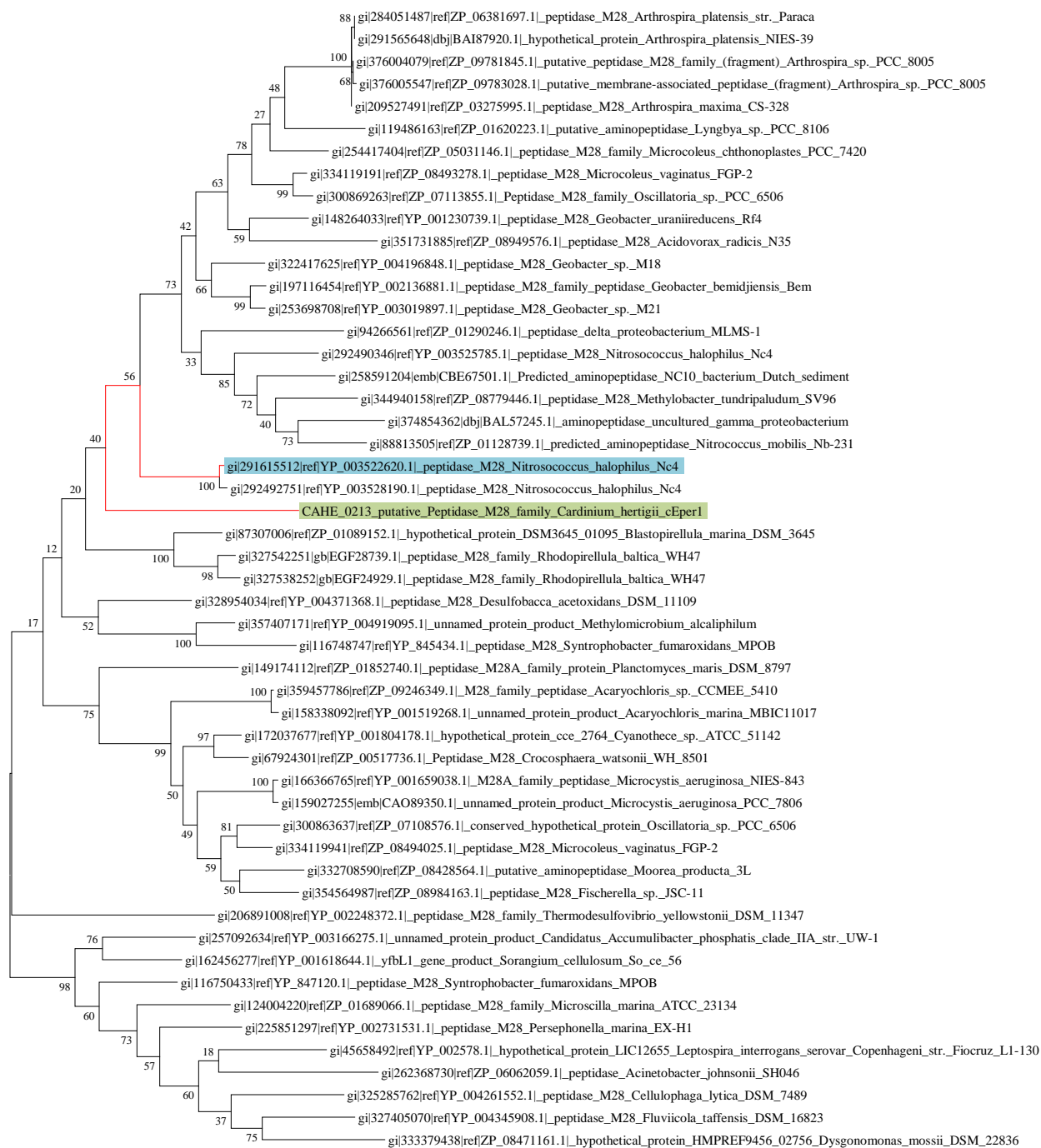

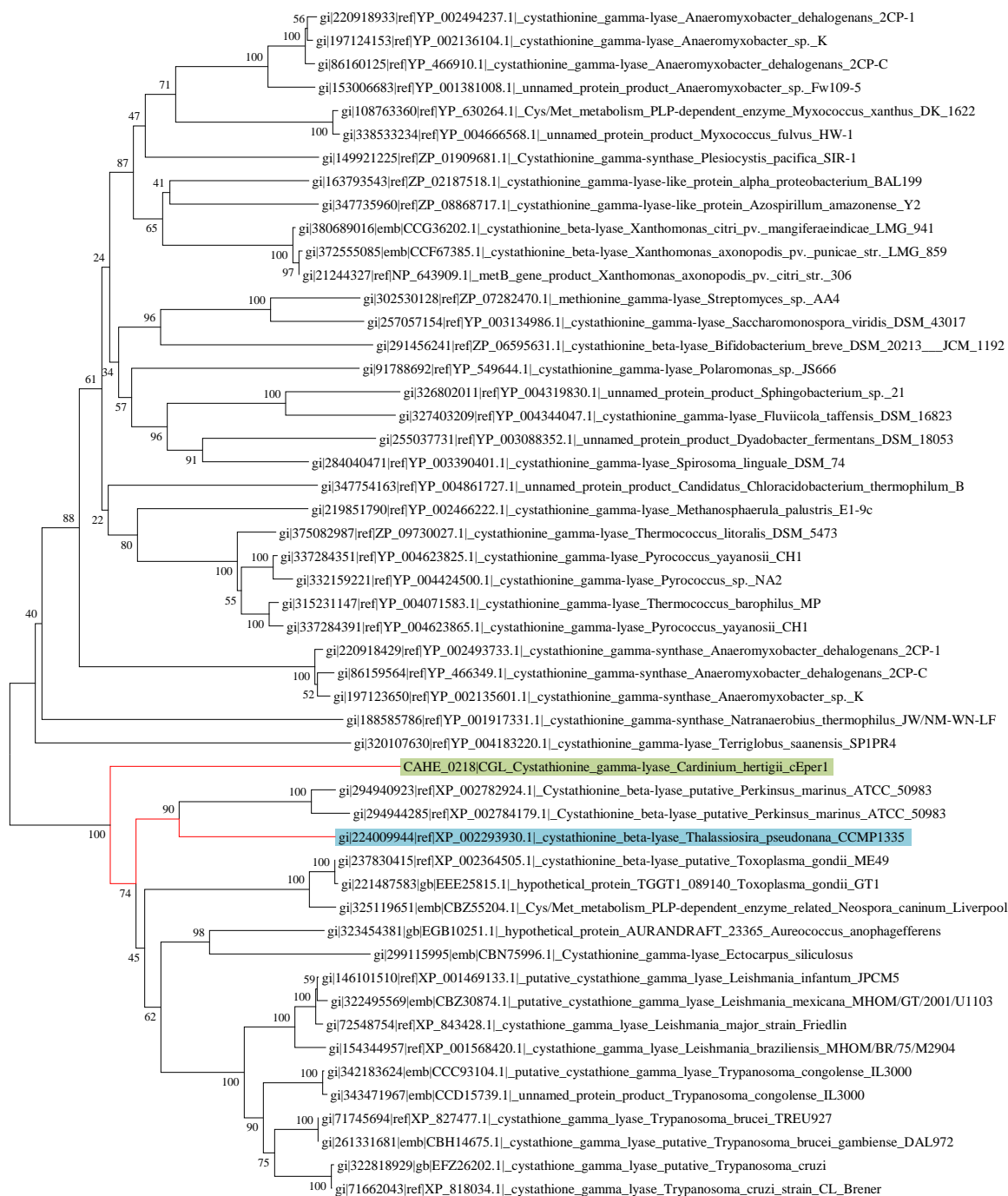

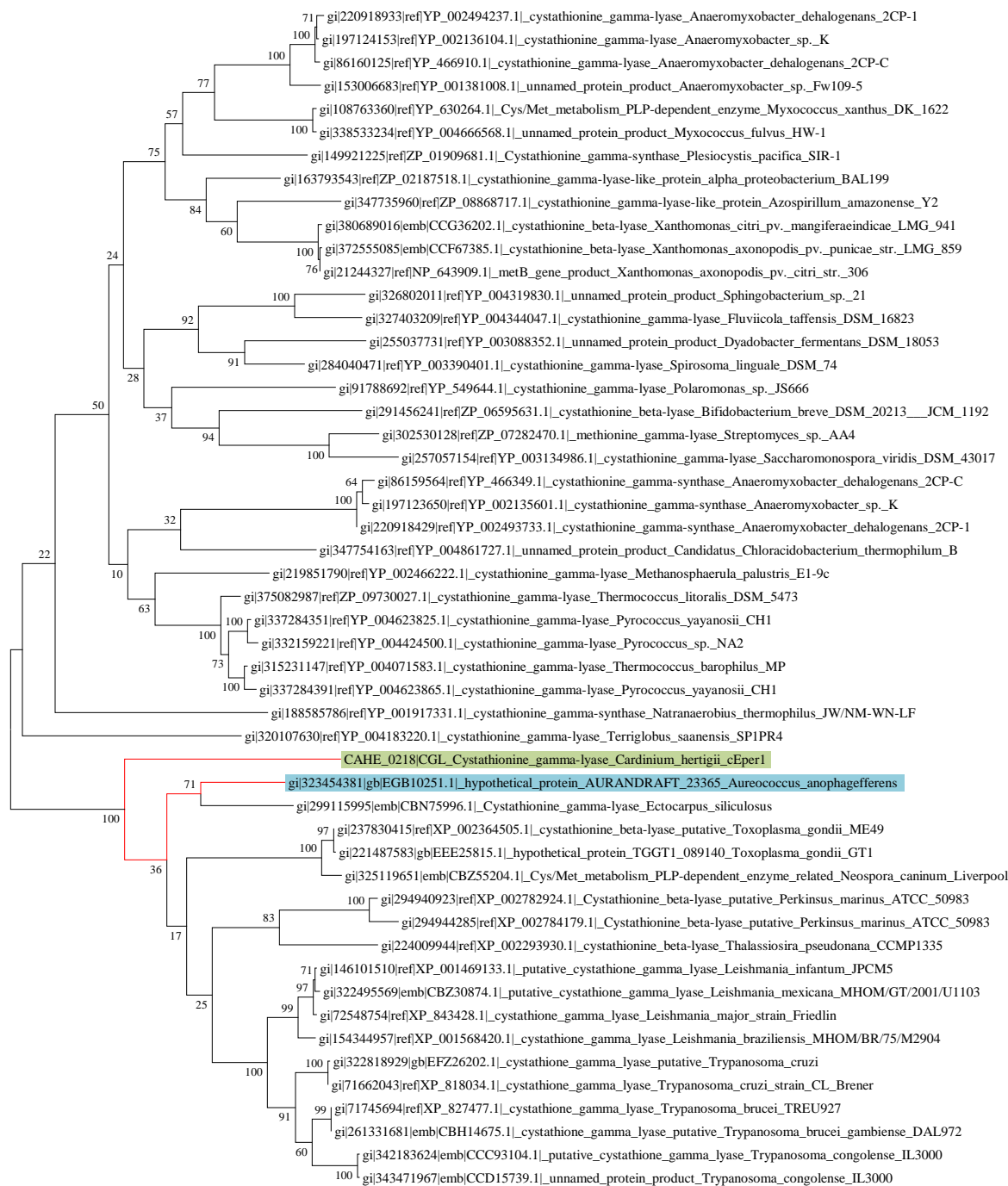

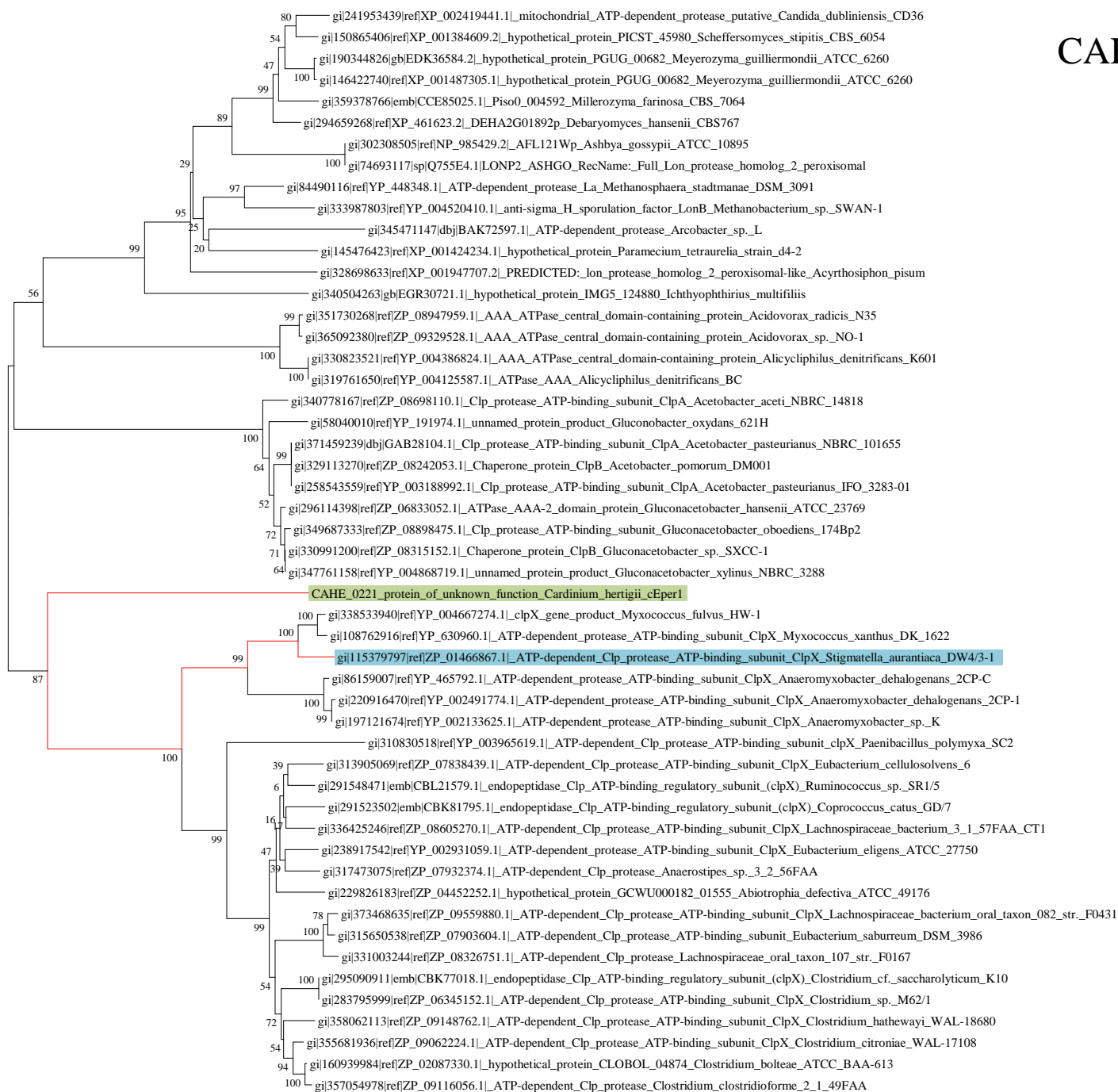

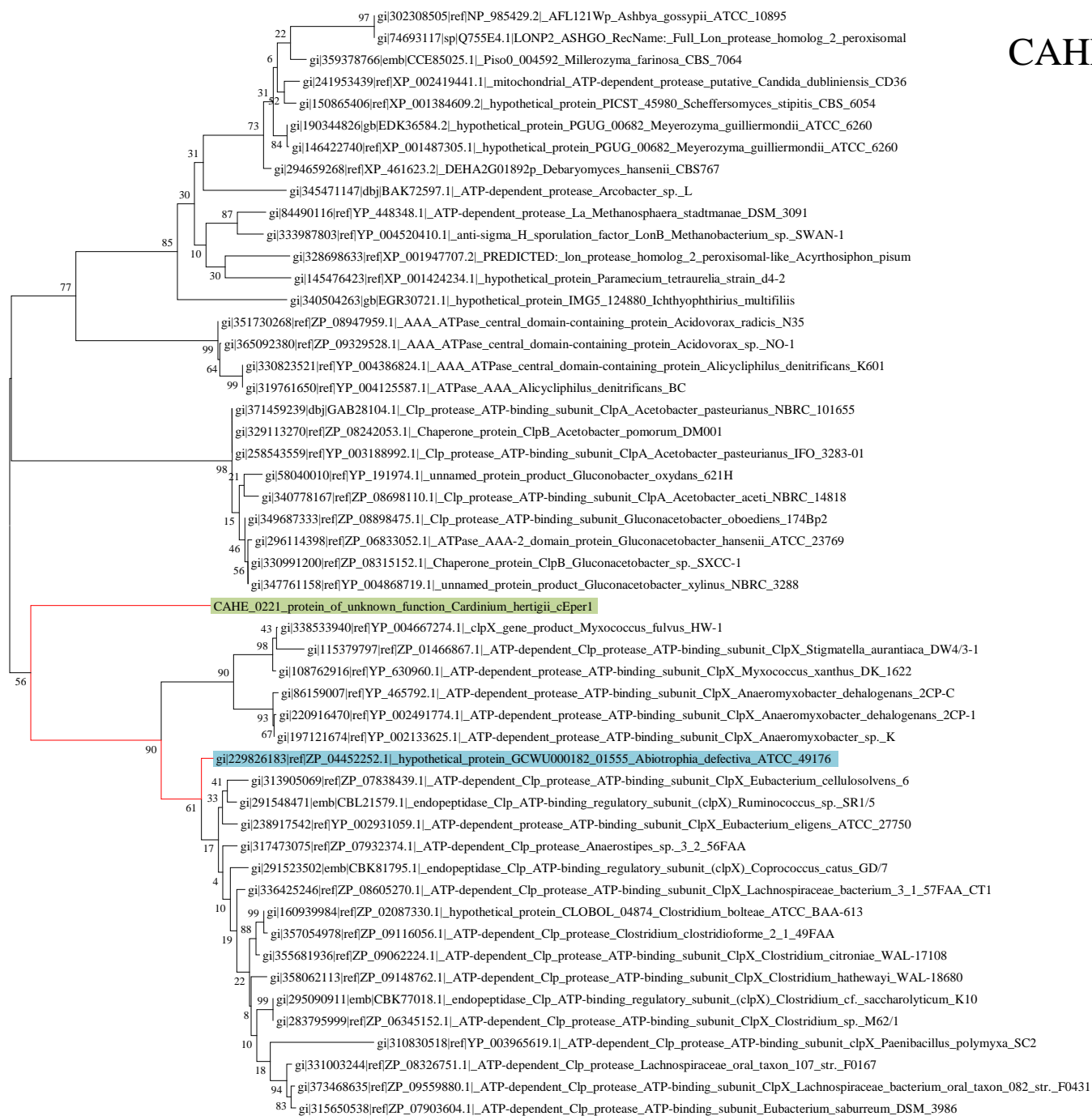

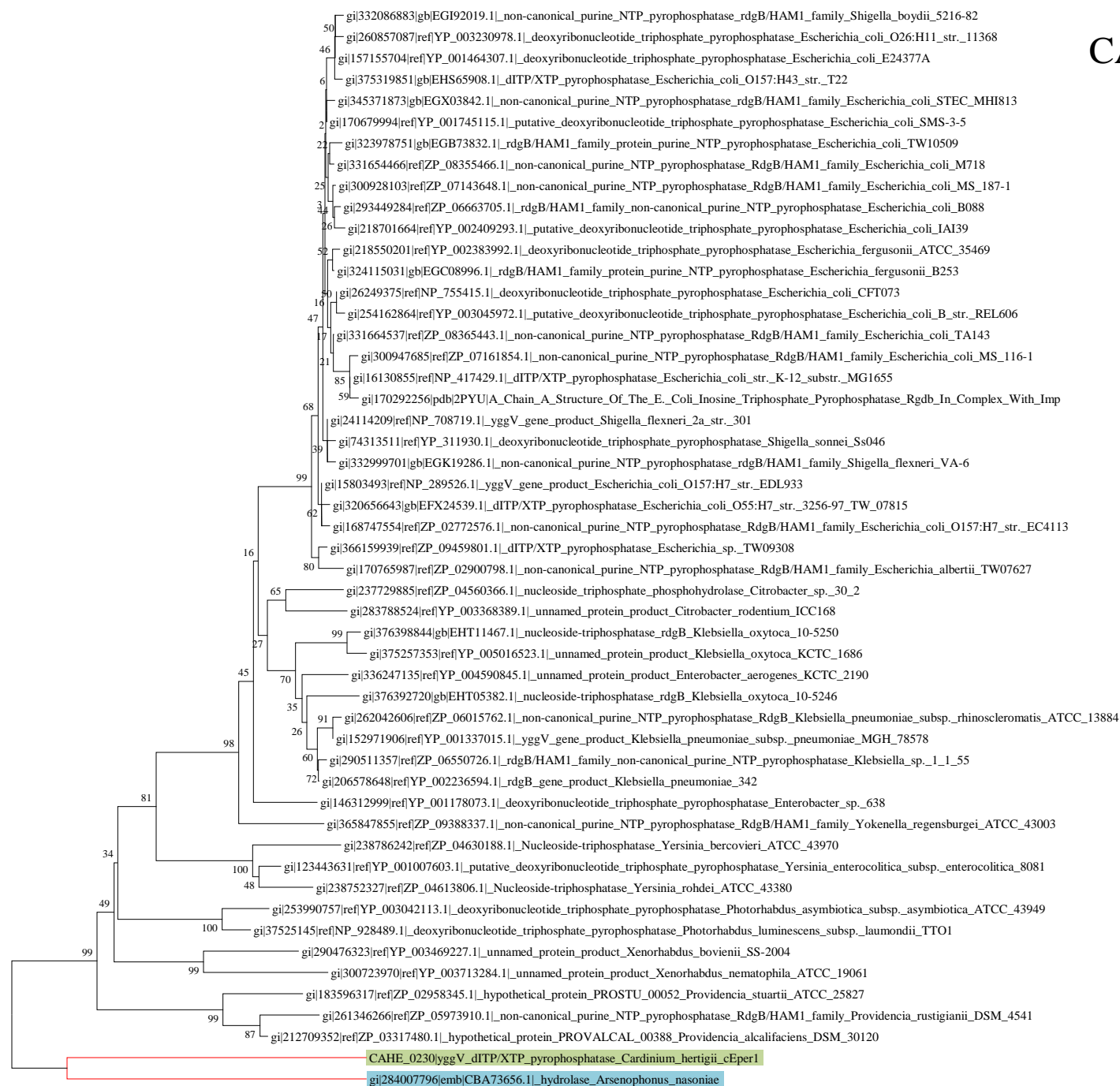

0.05

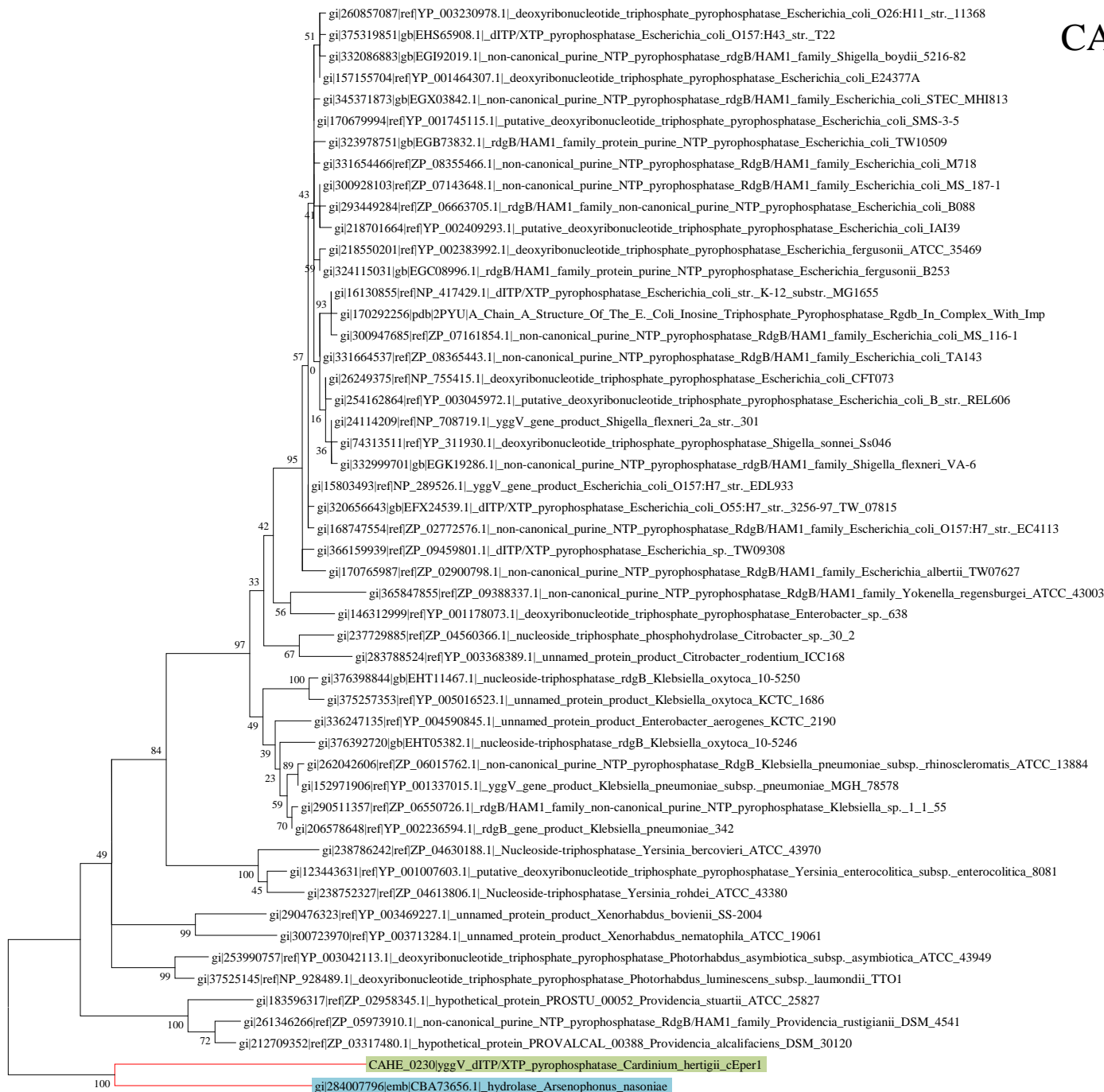

0.05

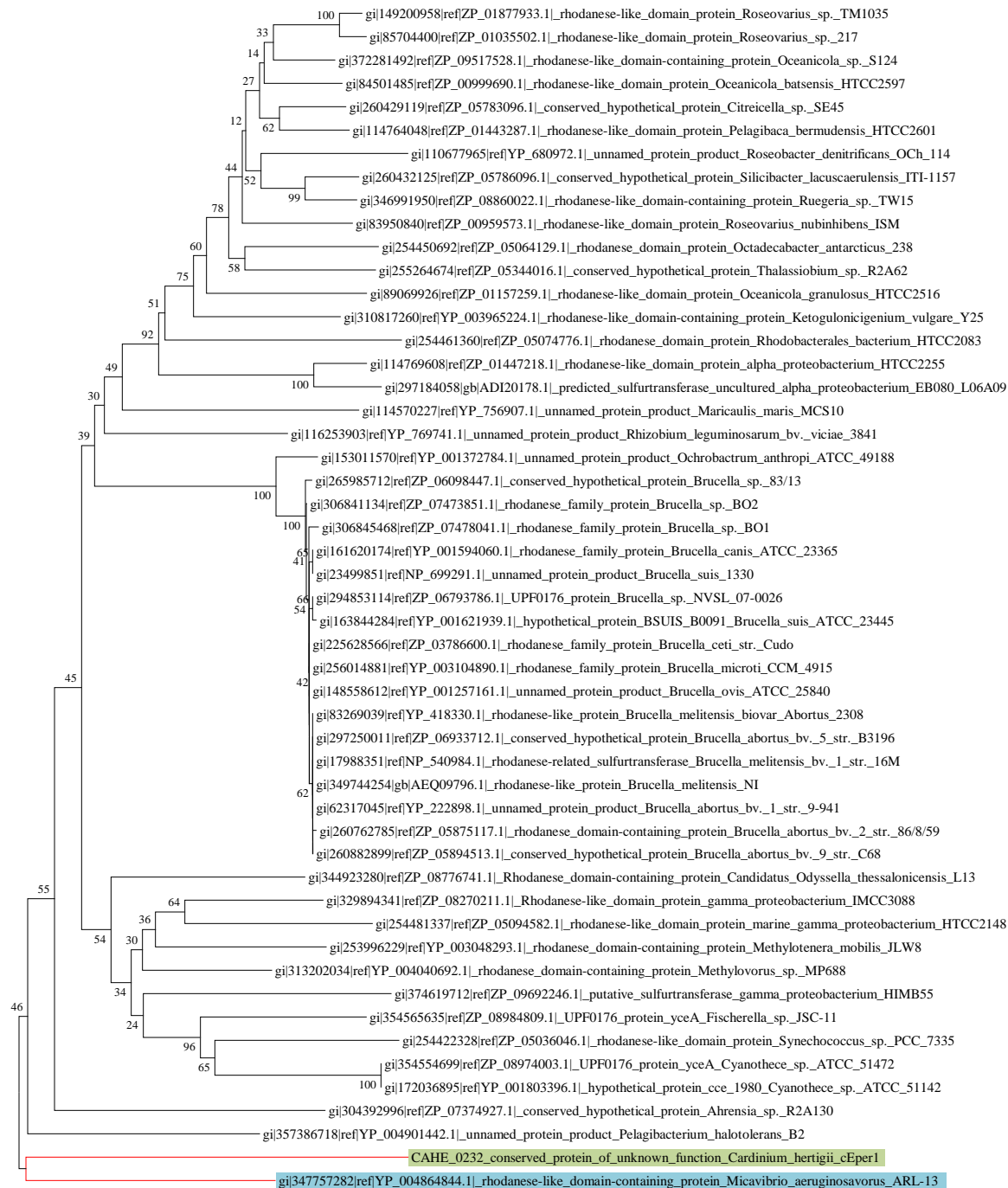

0.05

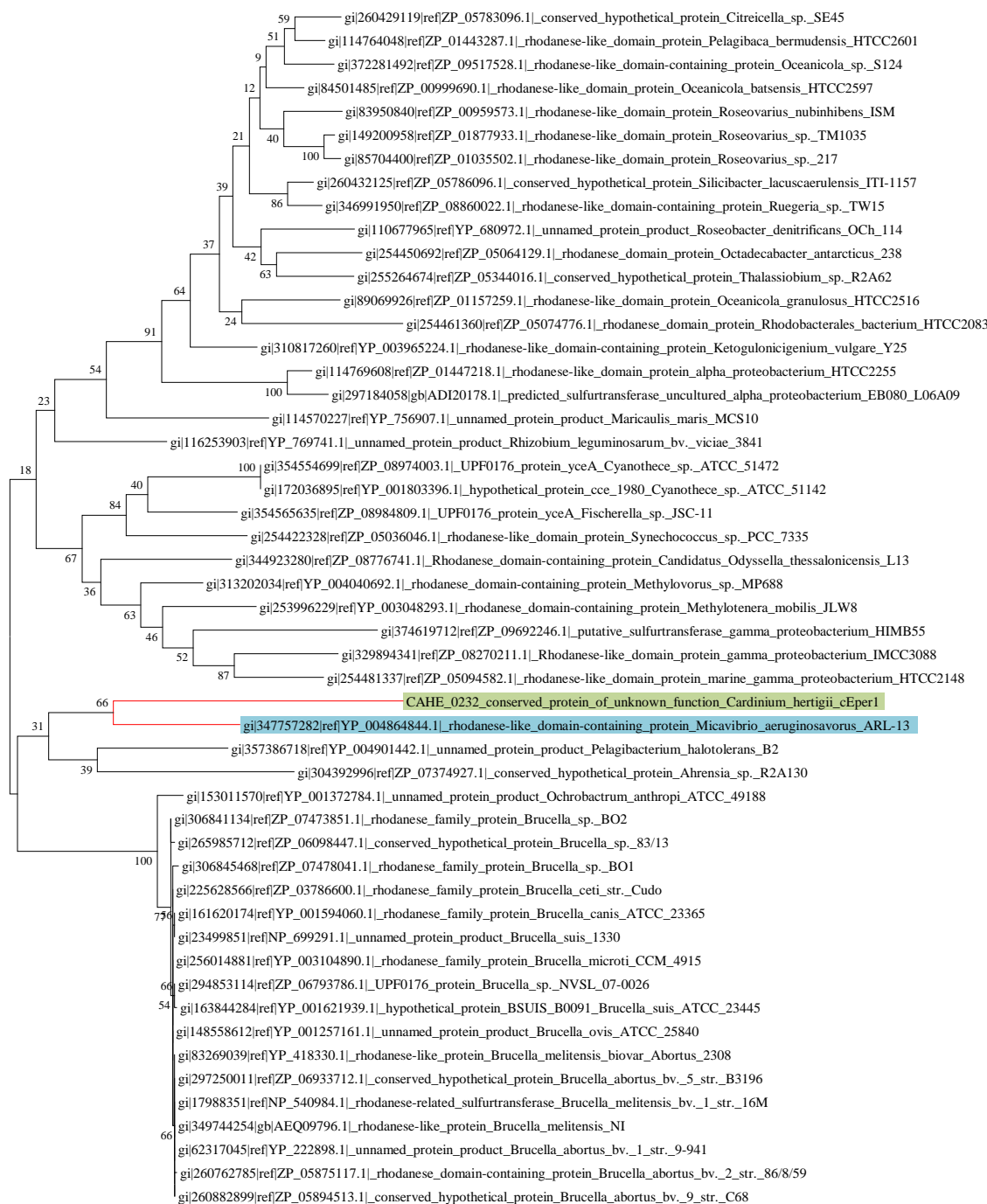

## CAHE\_0239 NJ

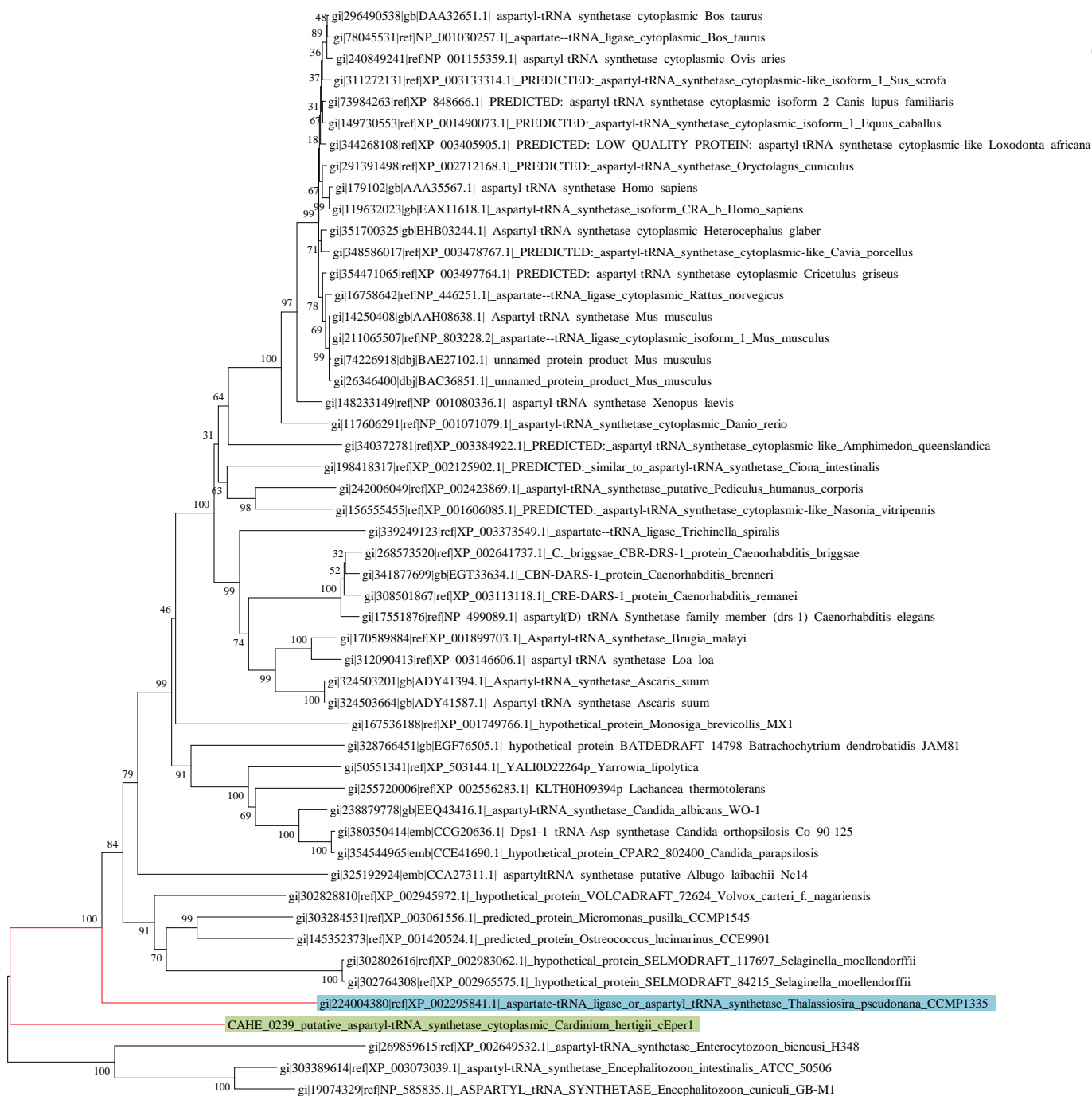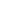

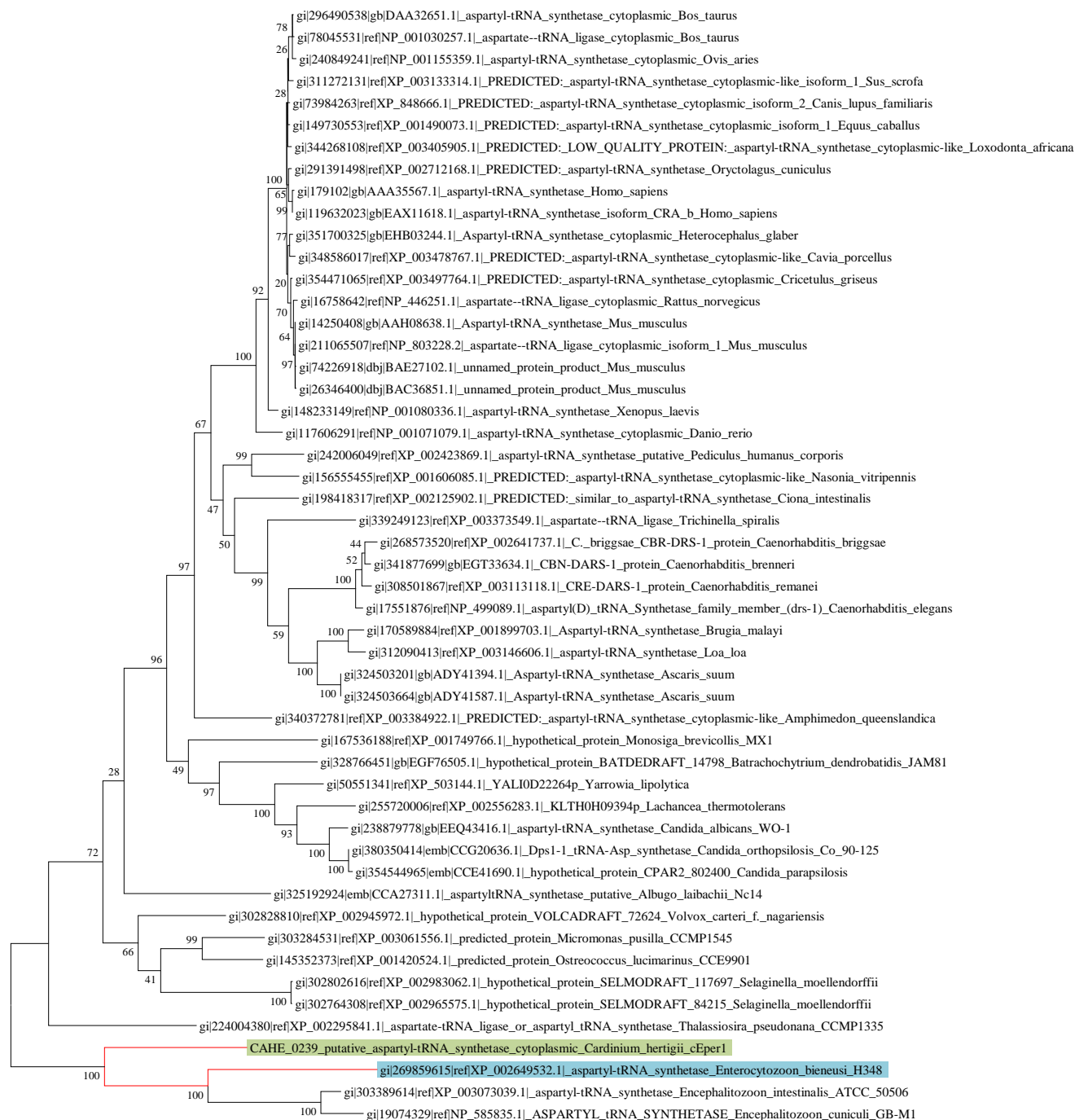

0.2

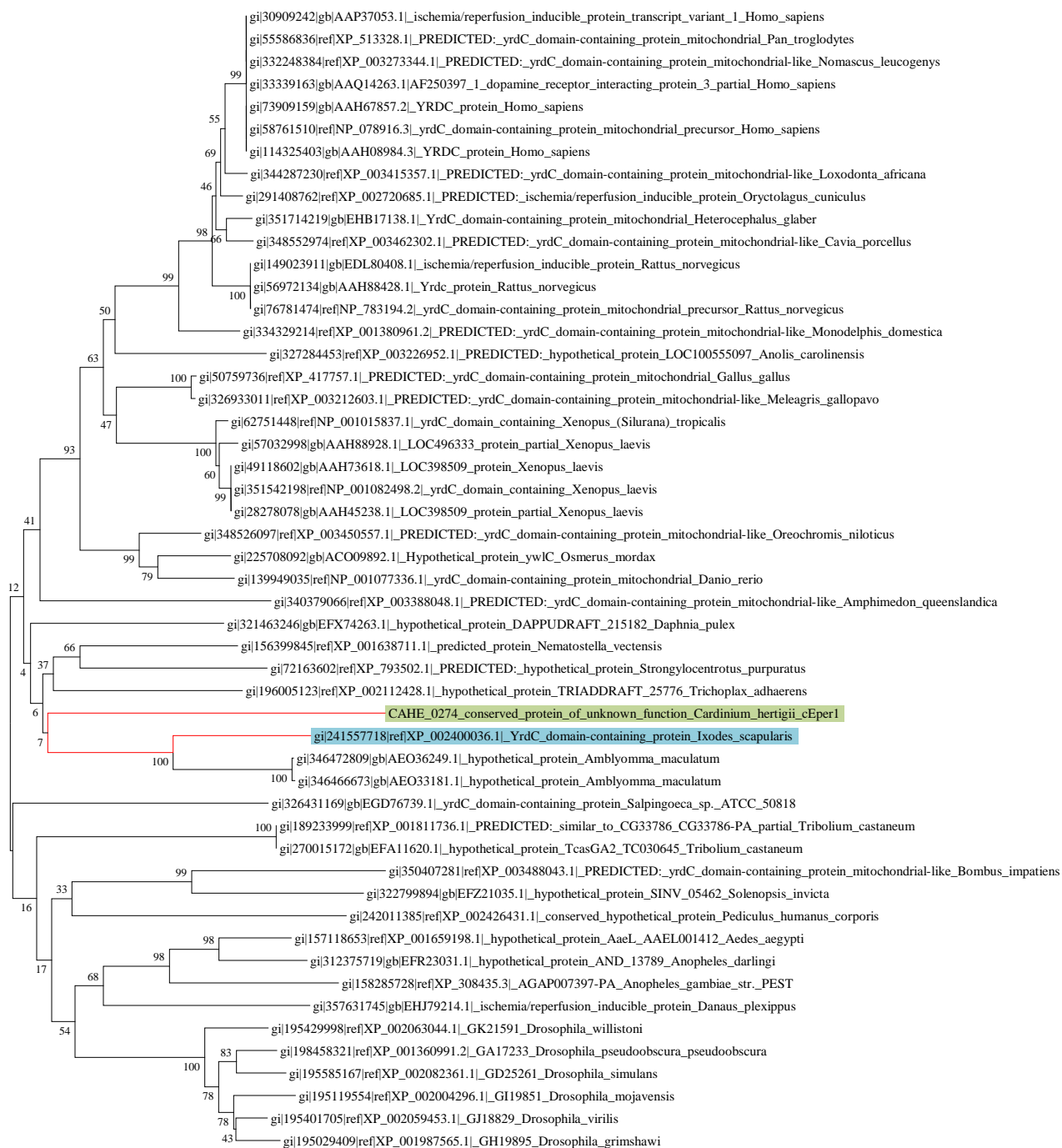

0.05

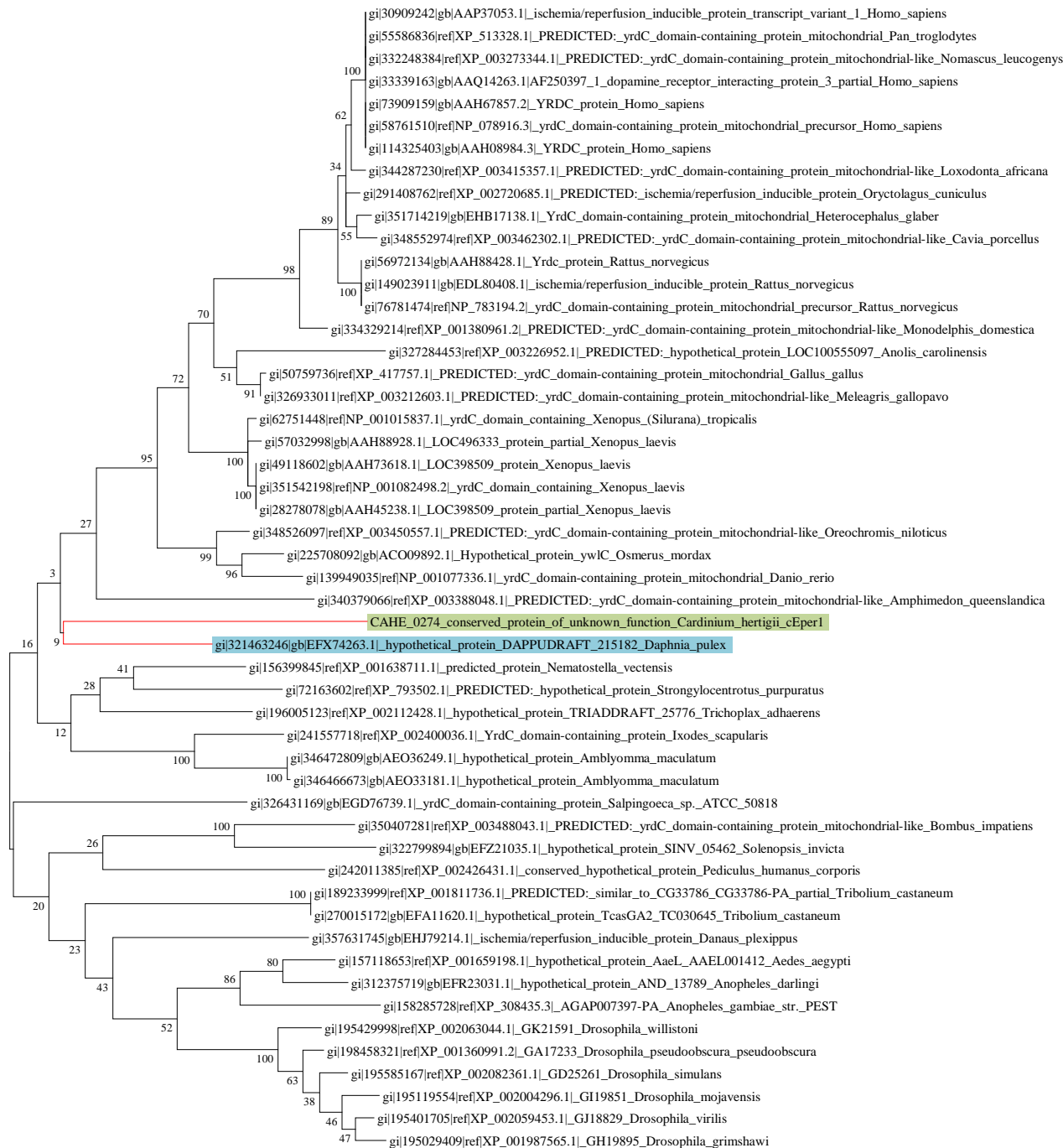

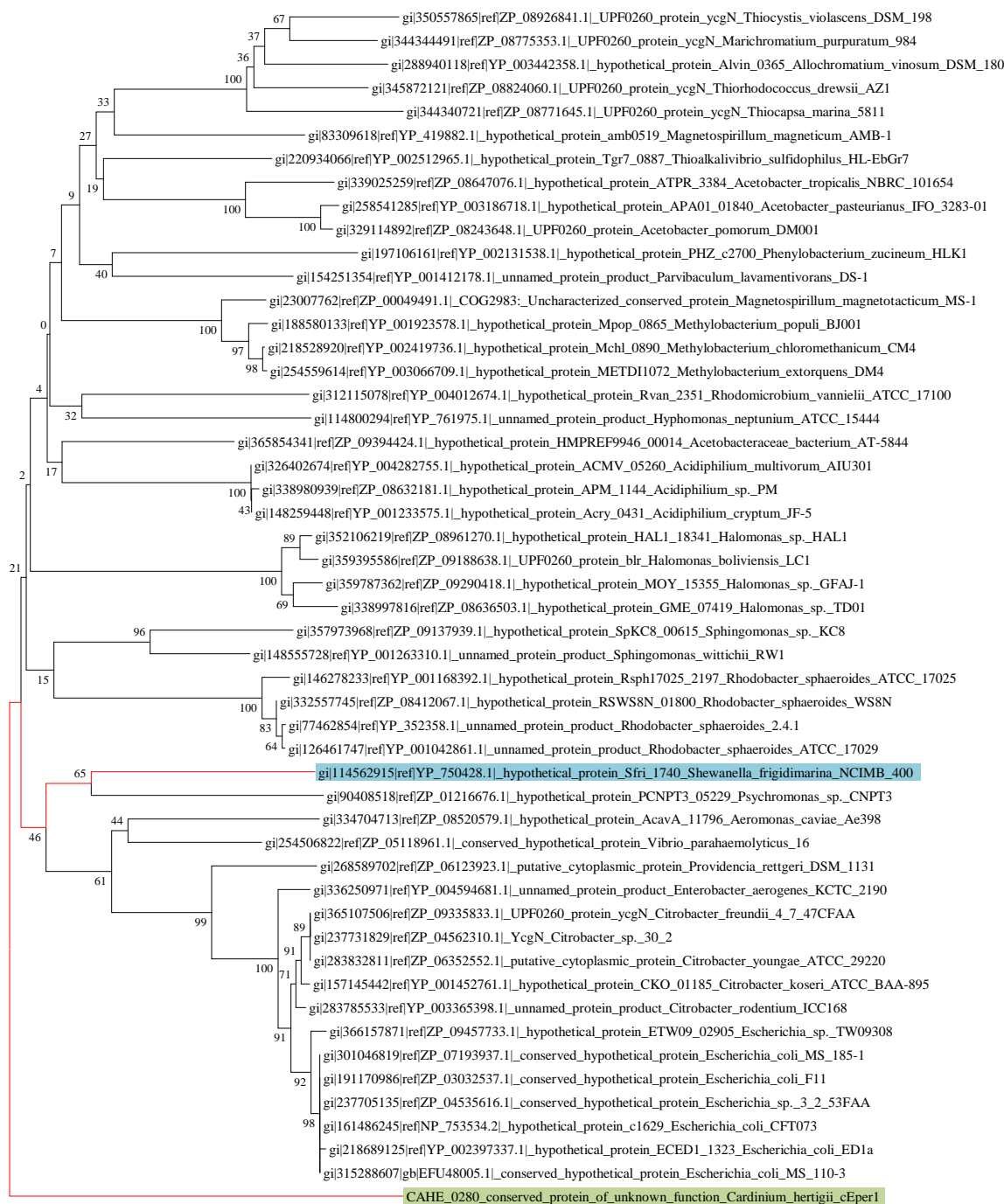

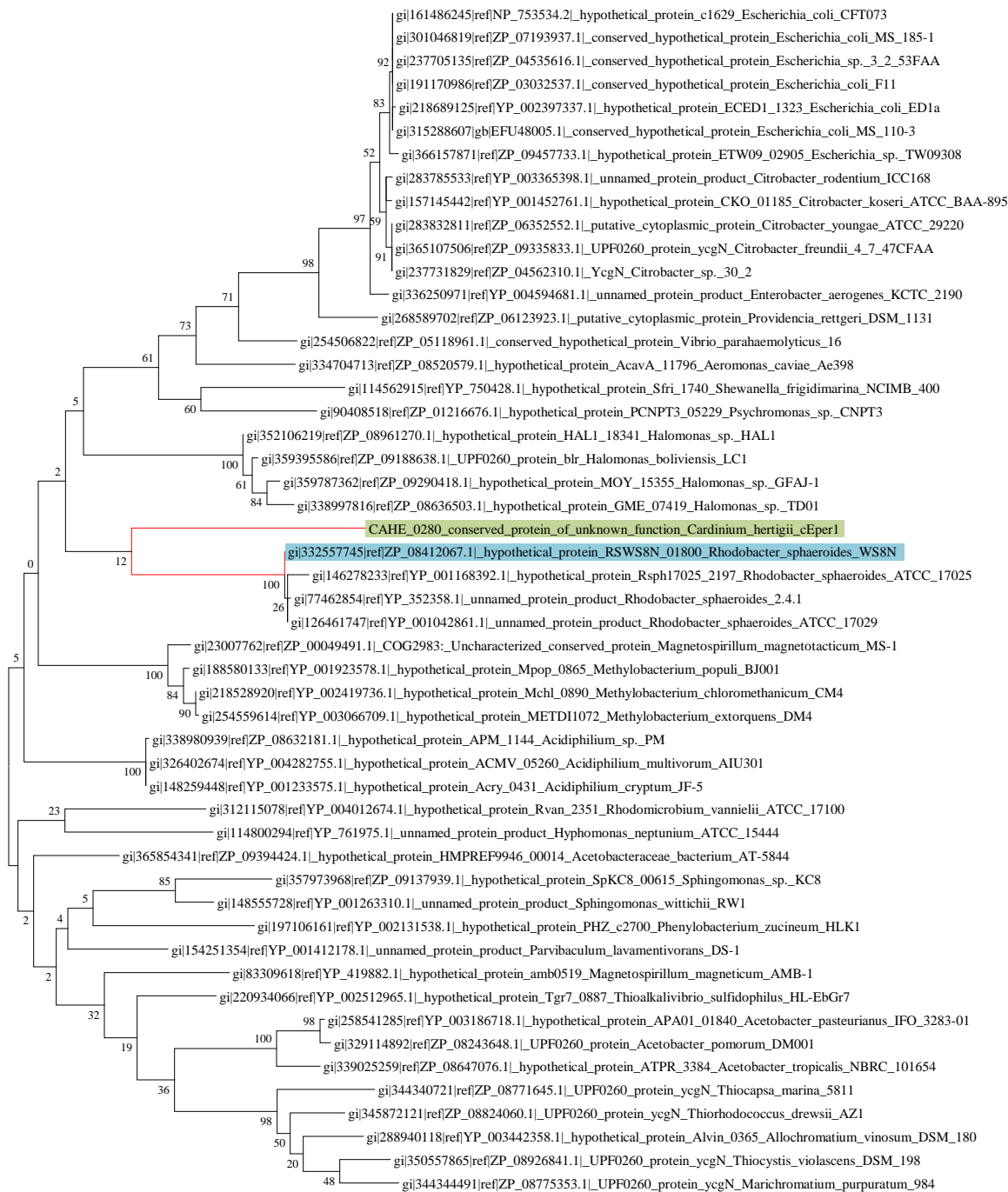

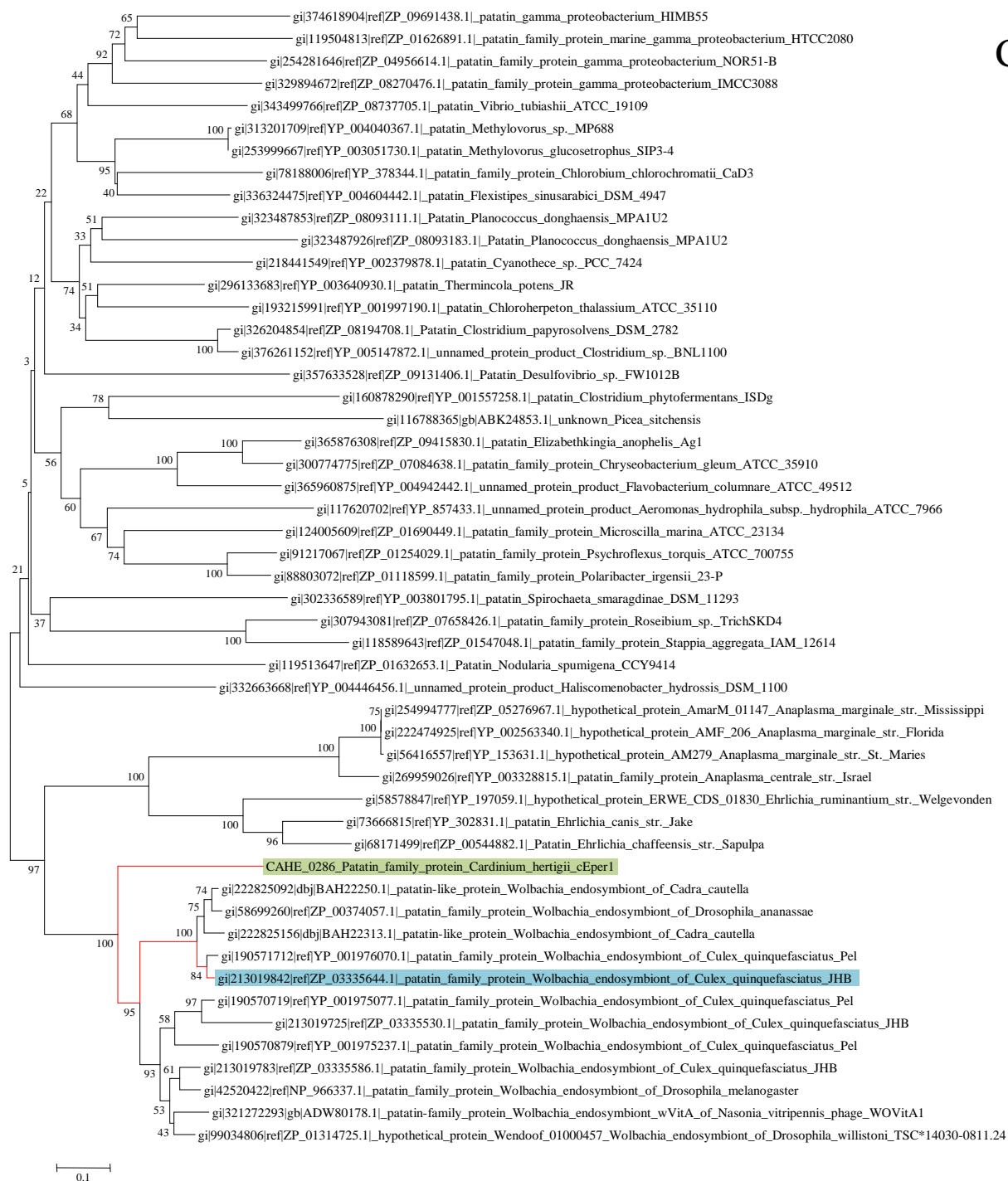

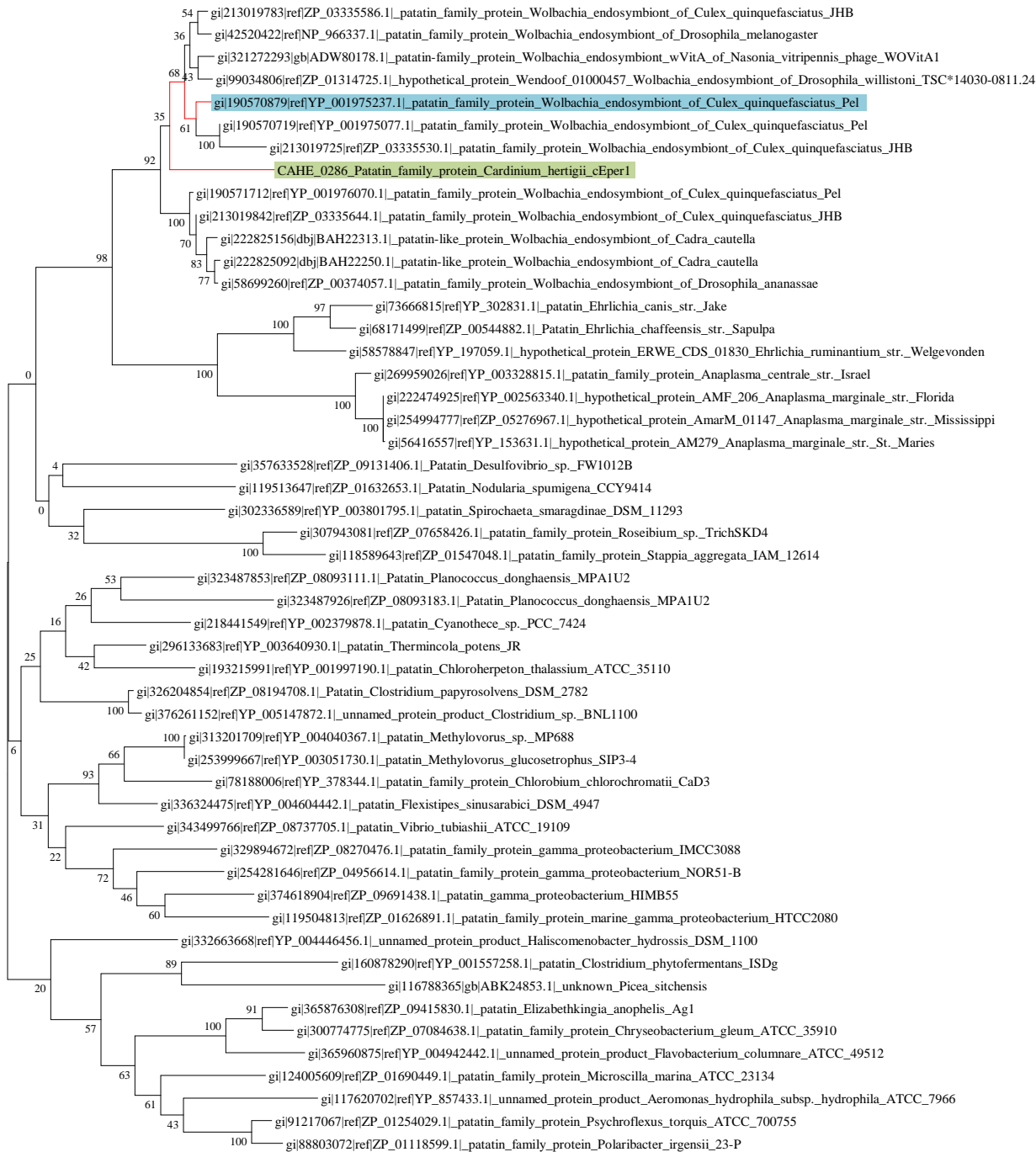

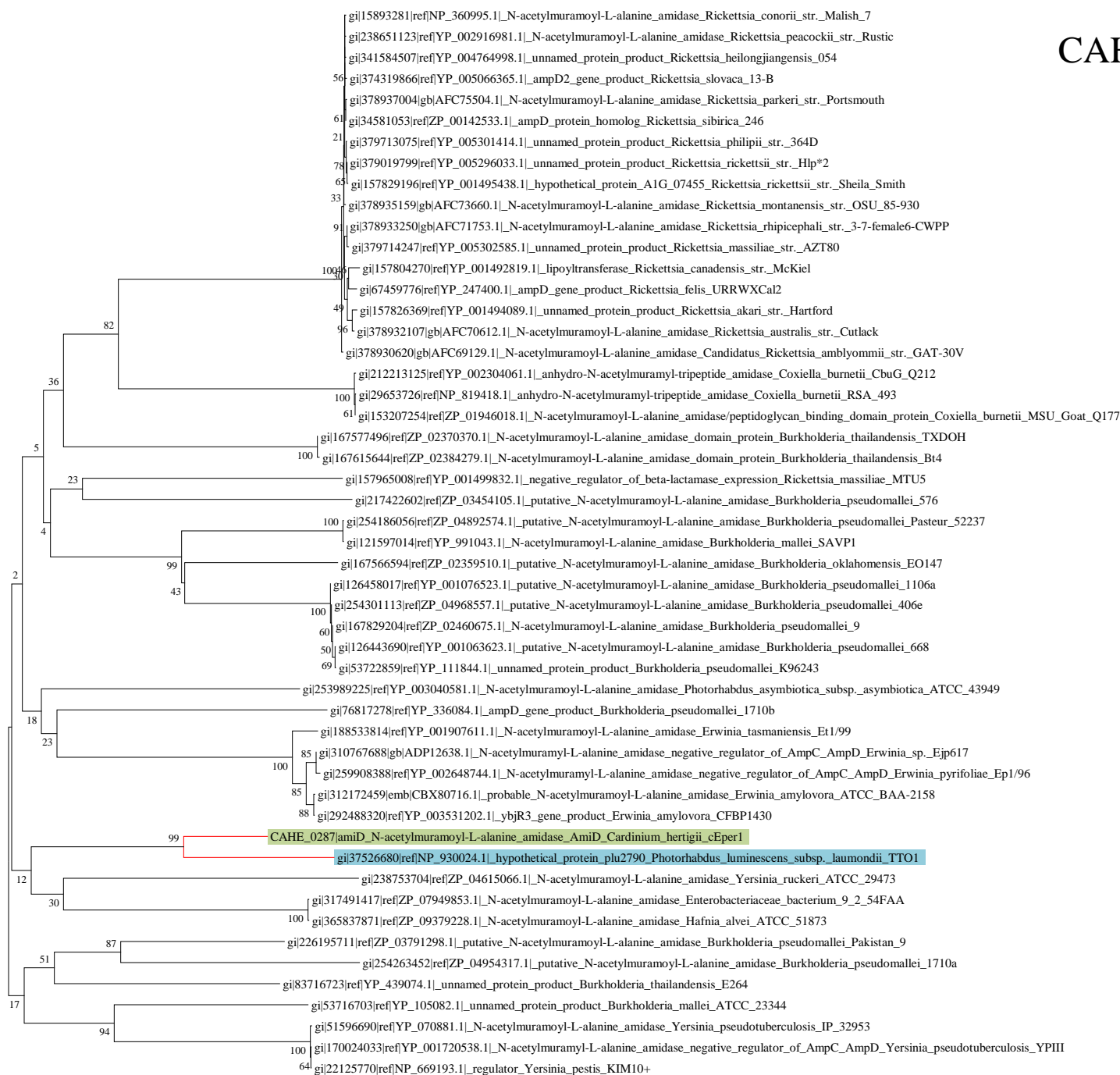

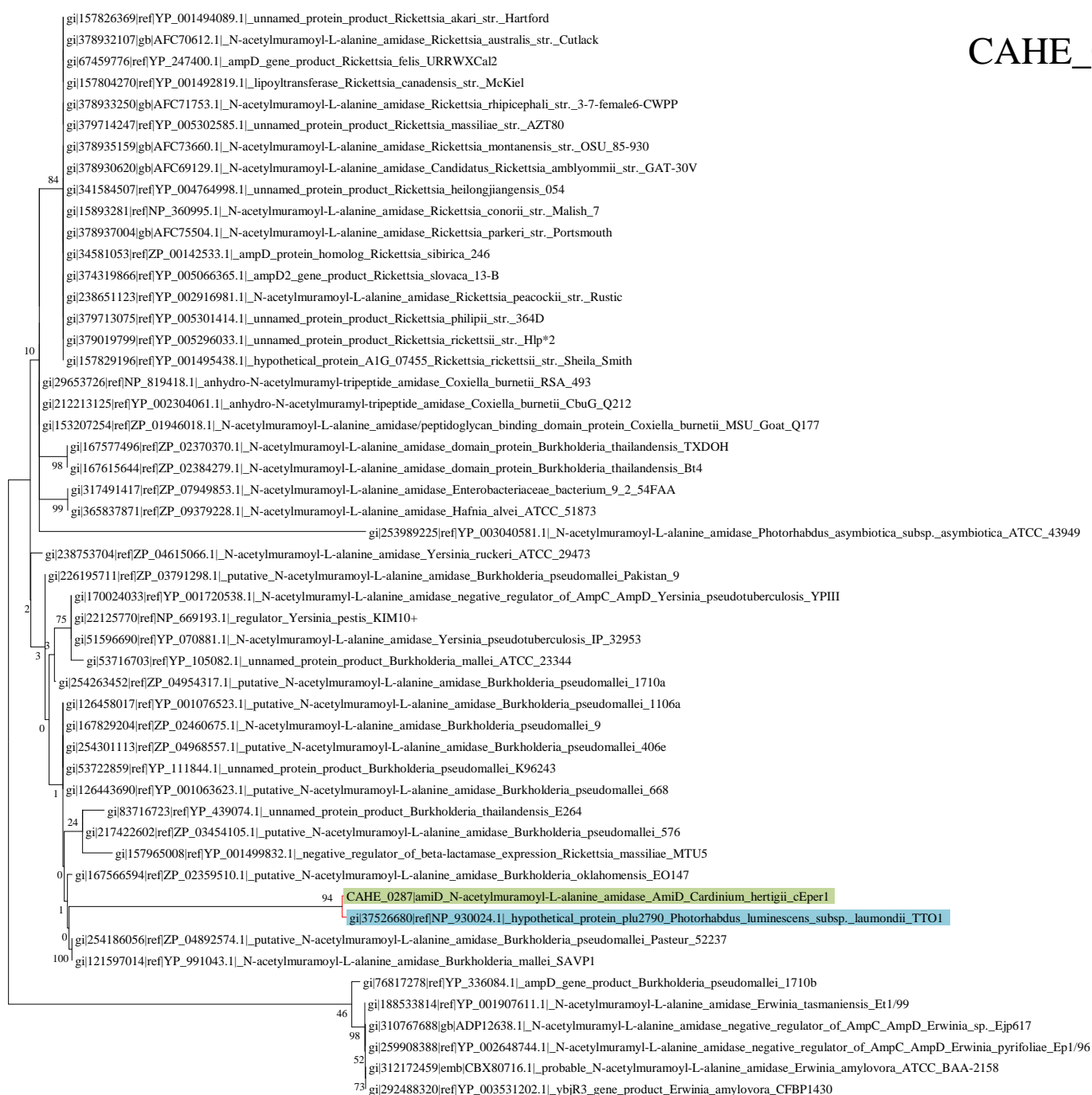

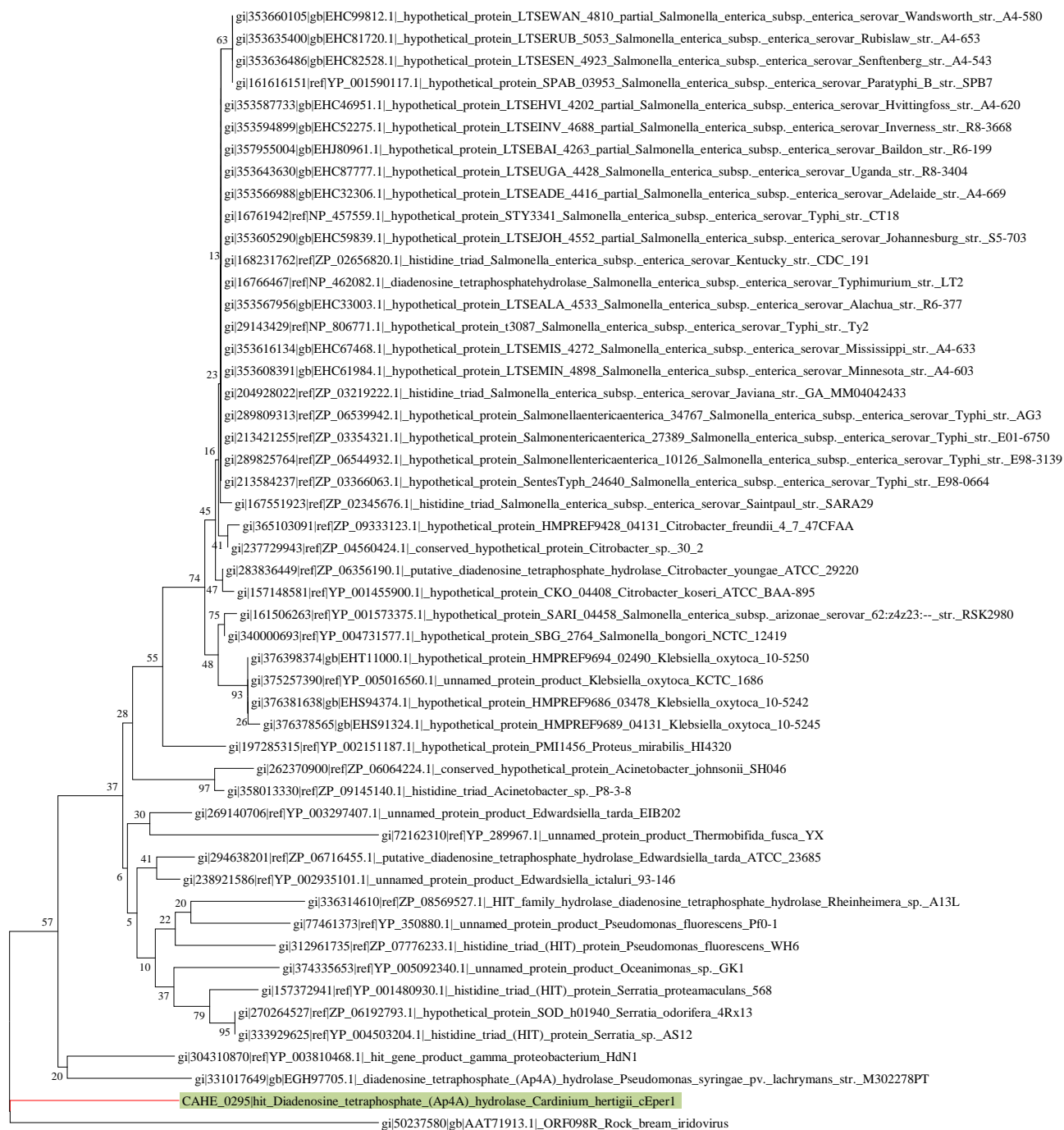

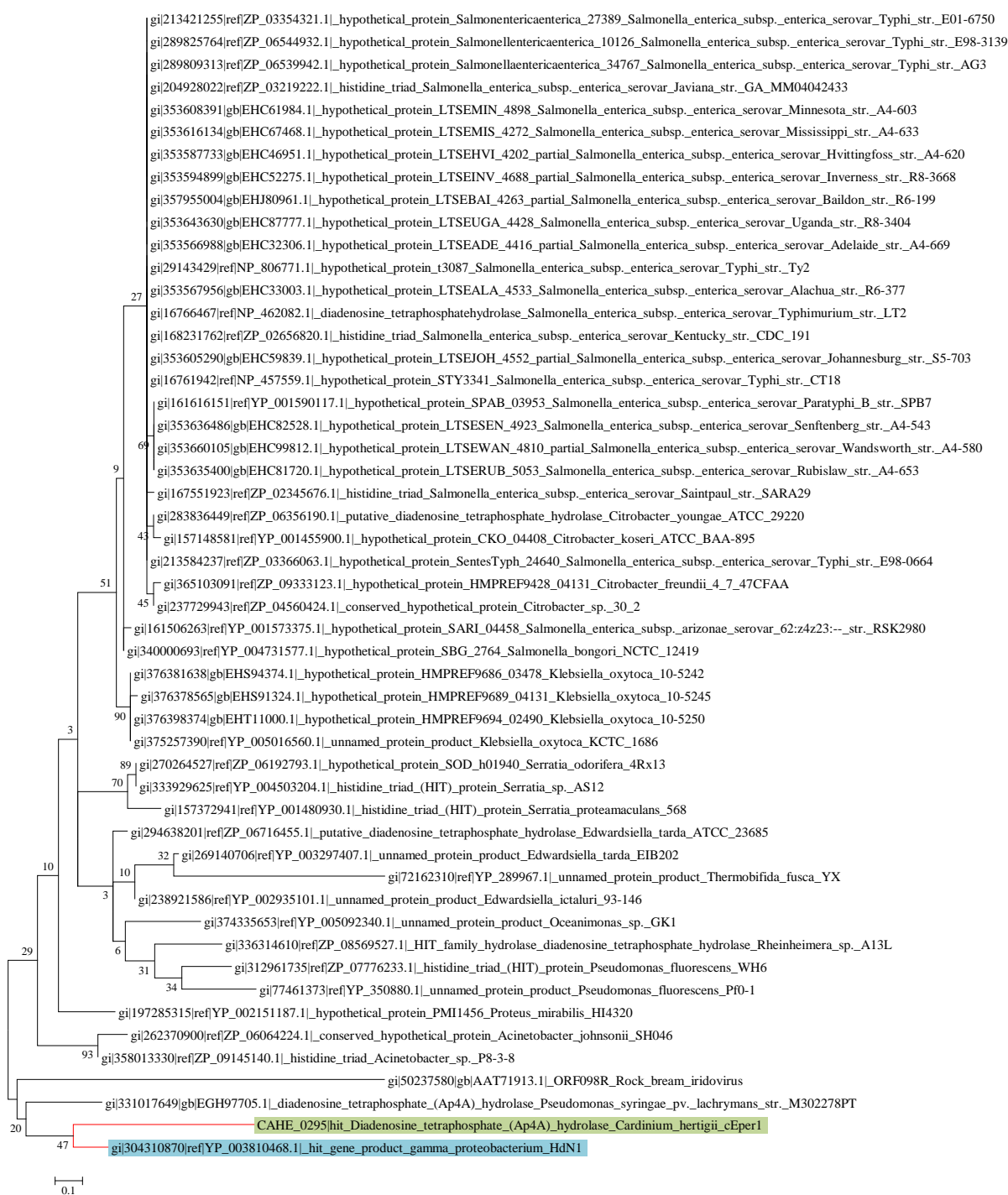

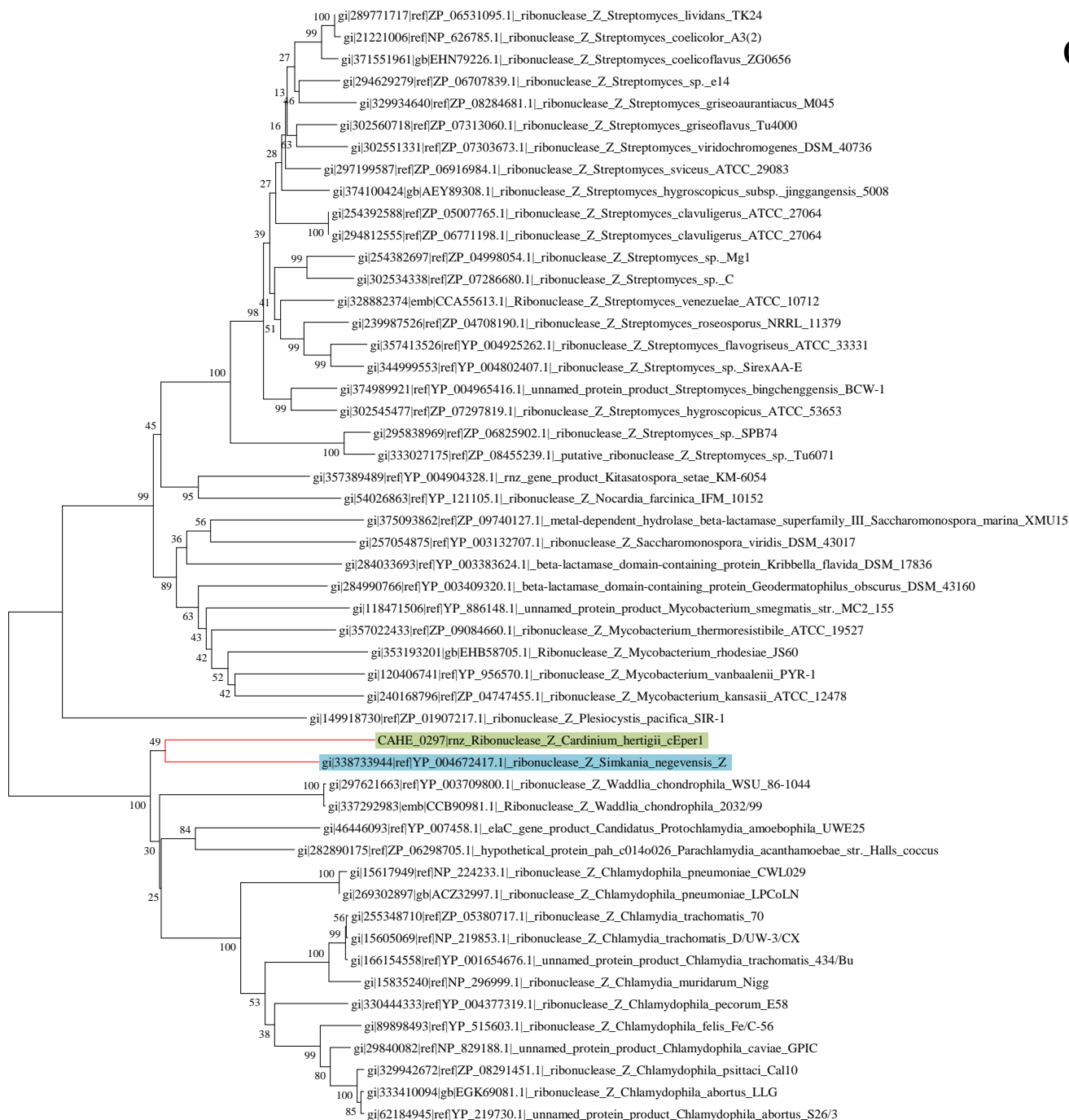

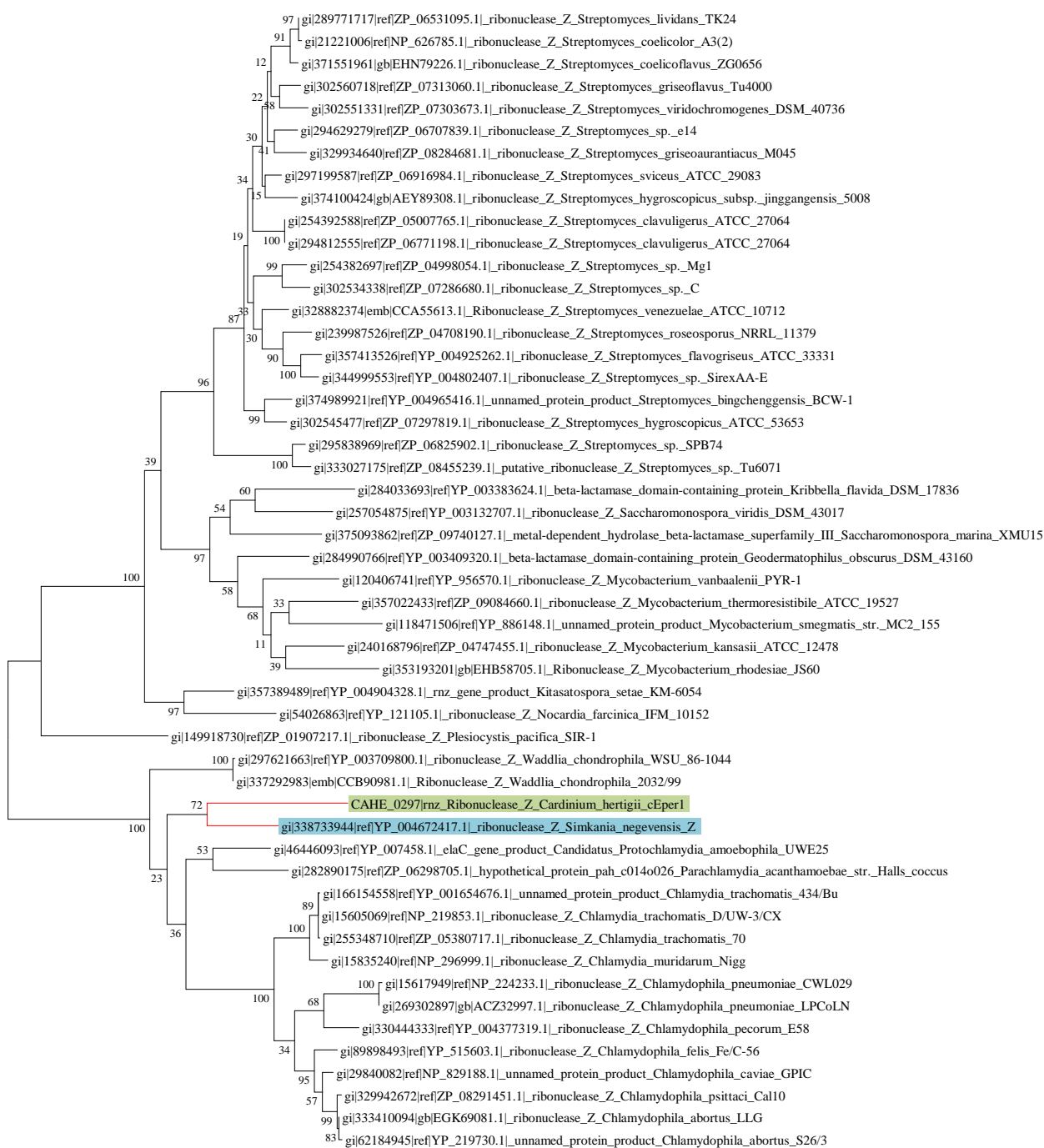

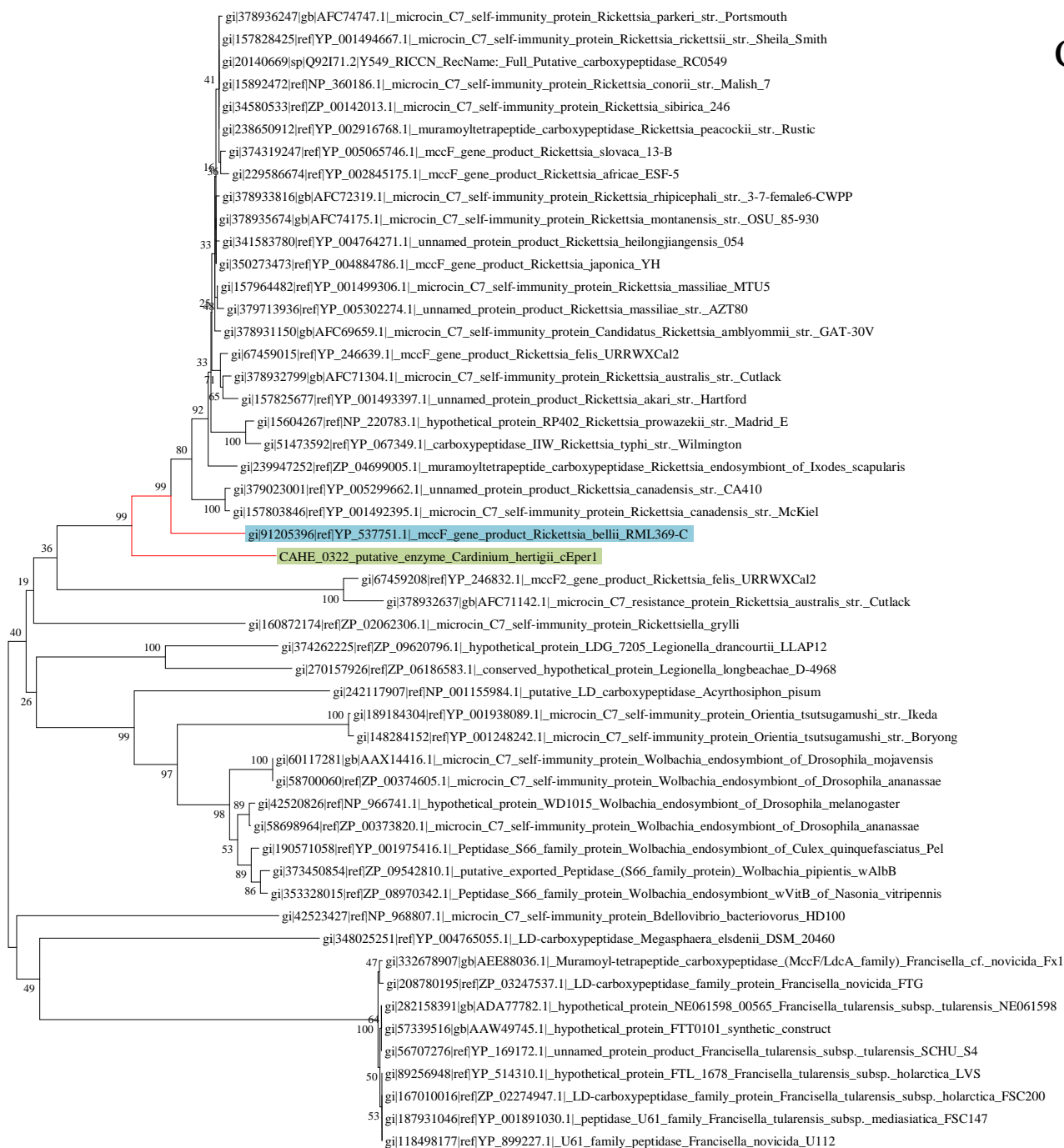

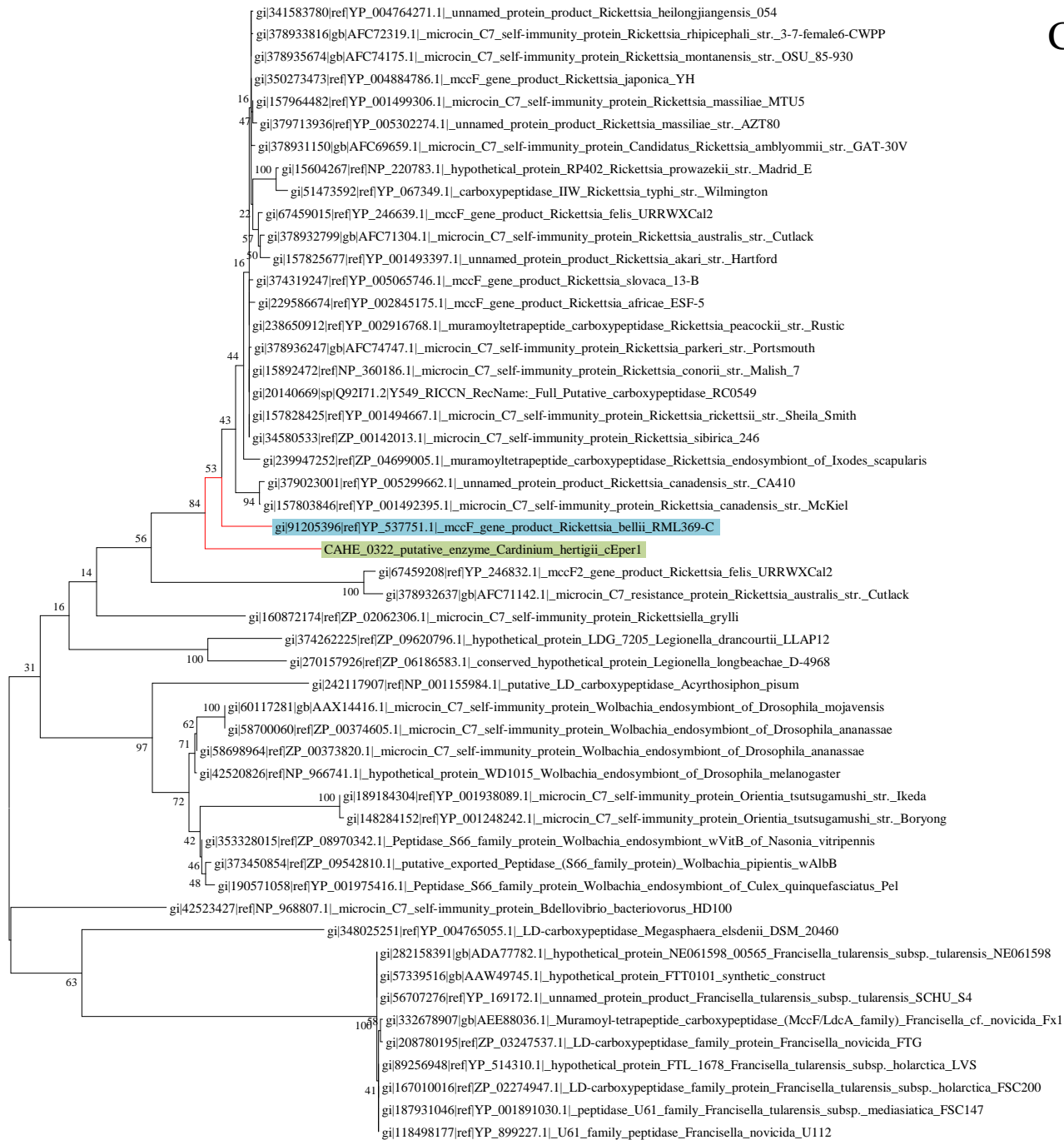

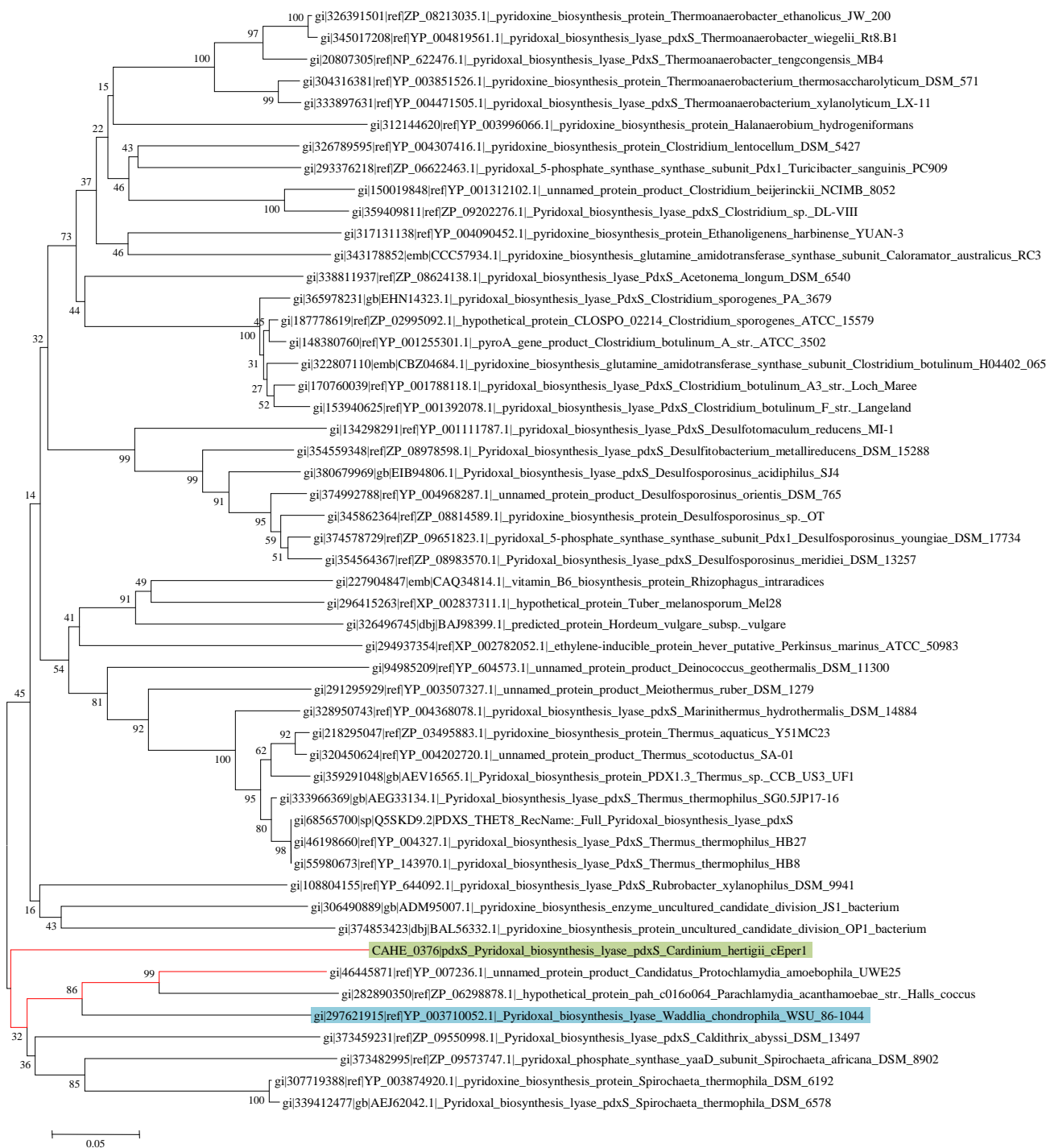

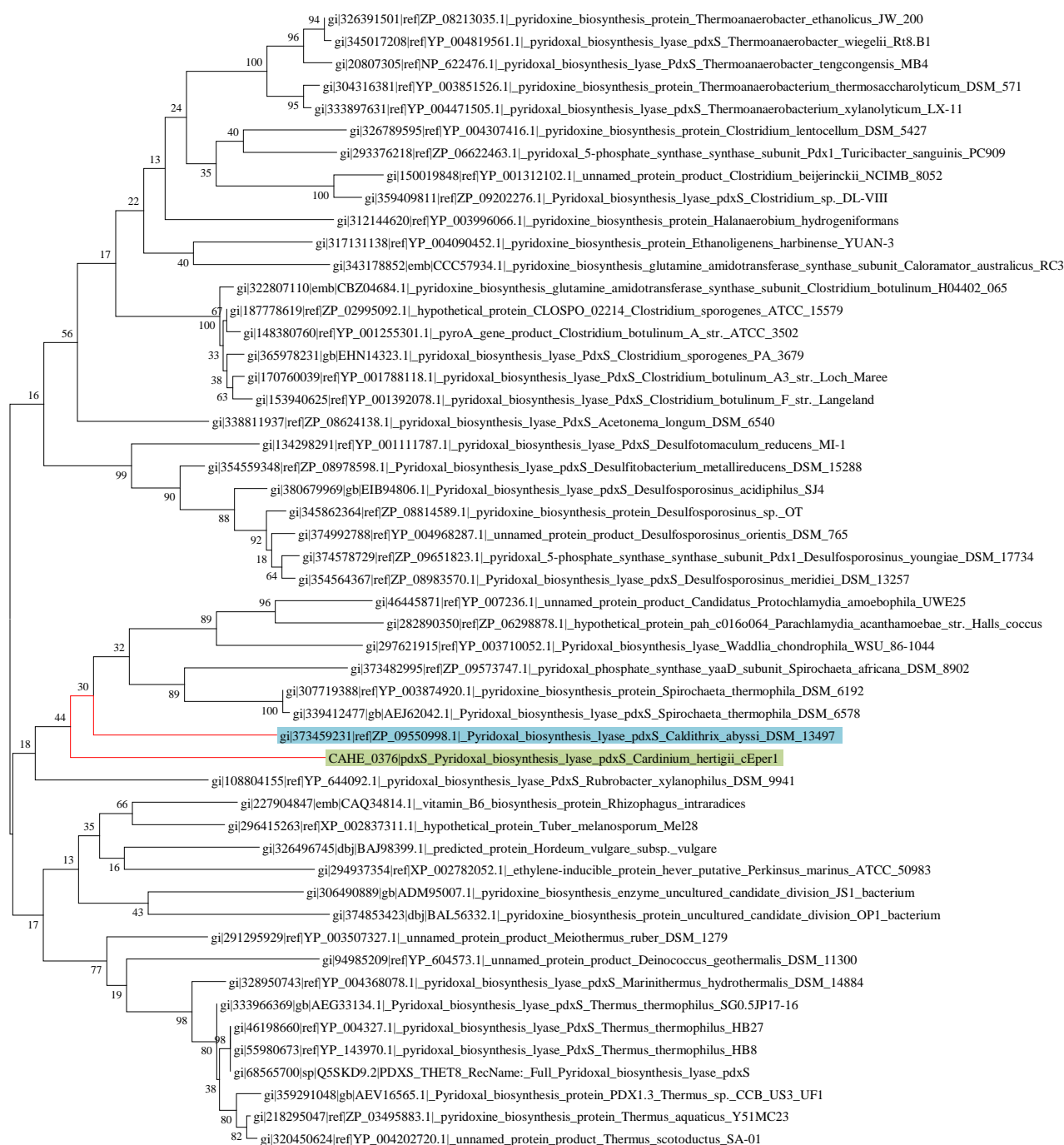

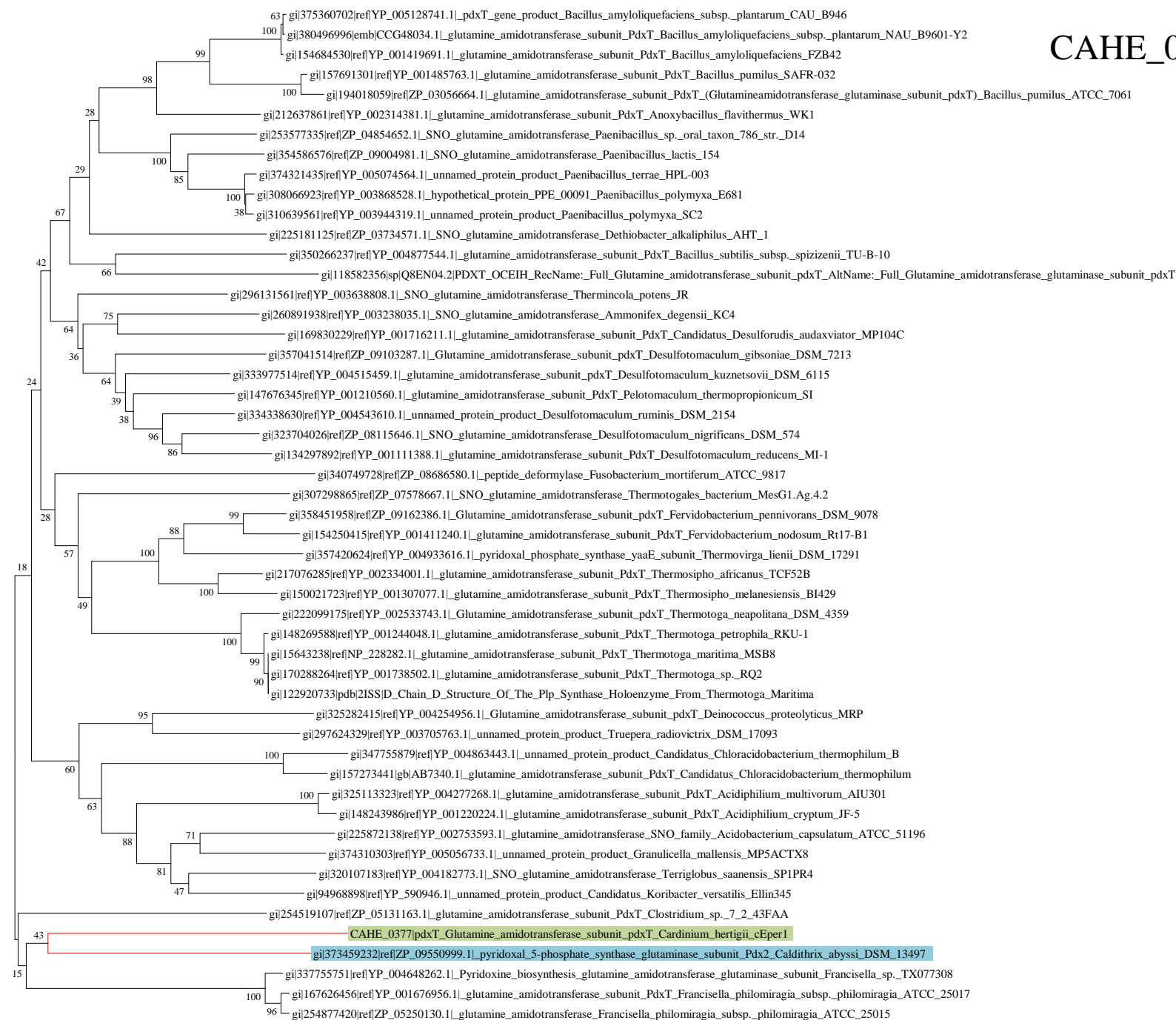

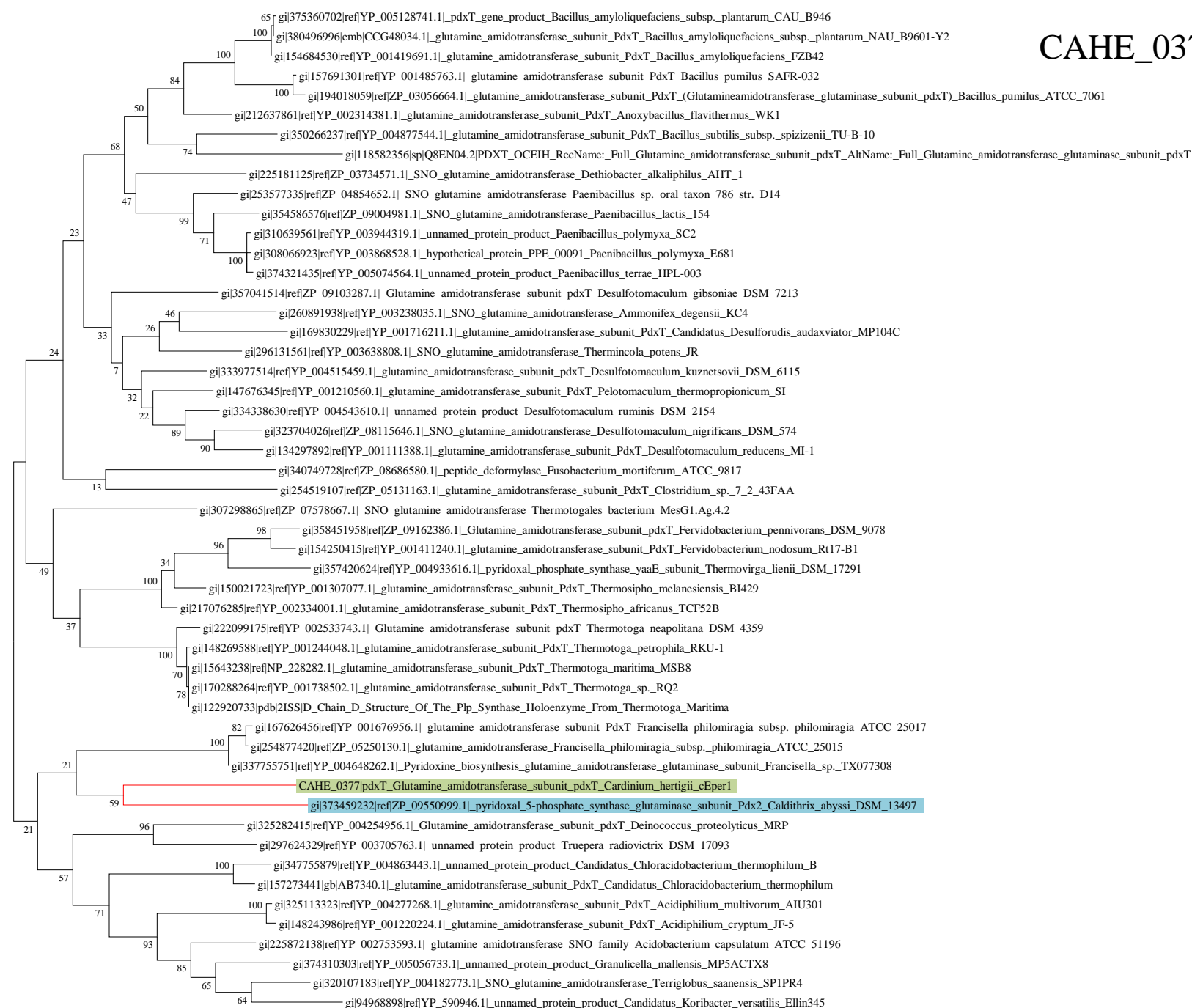

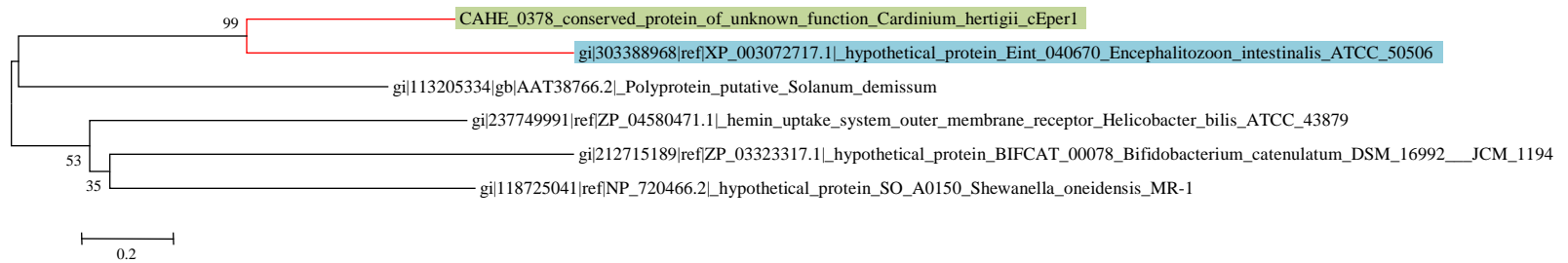

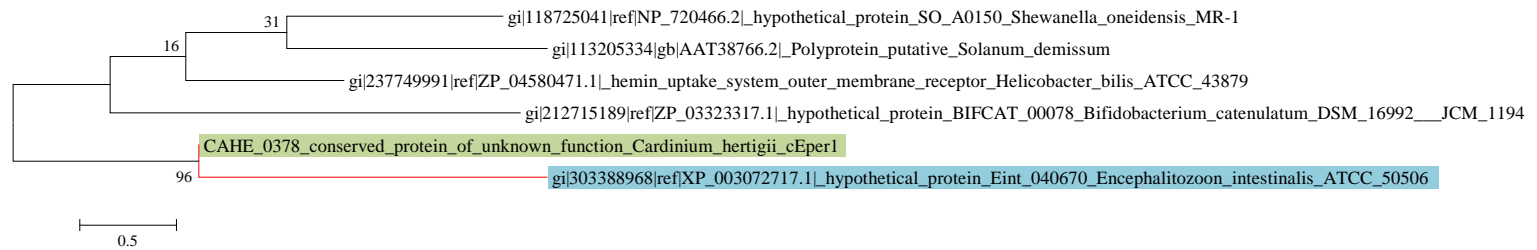

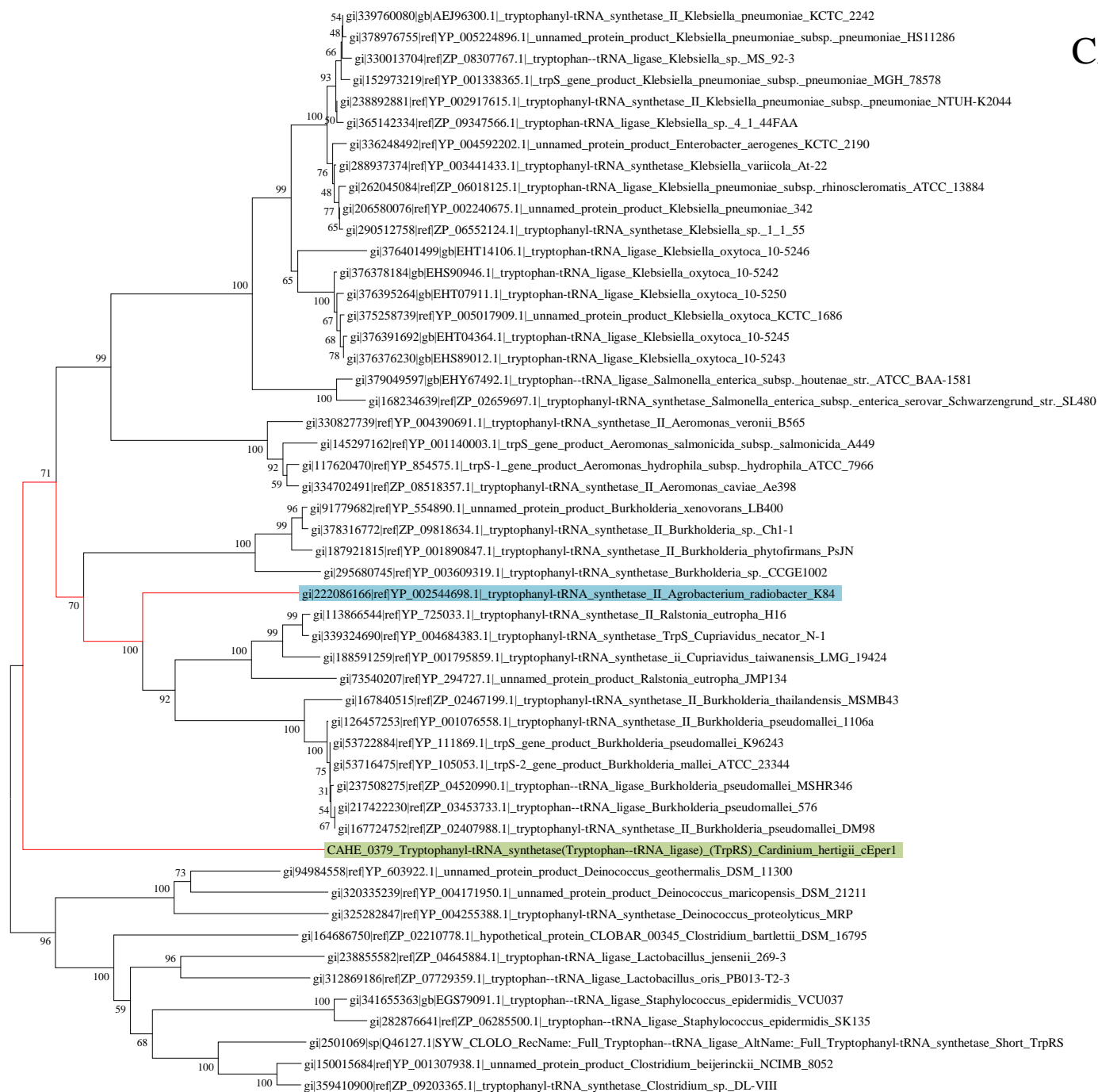

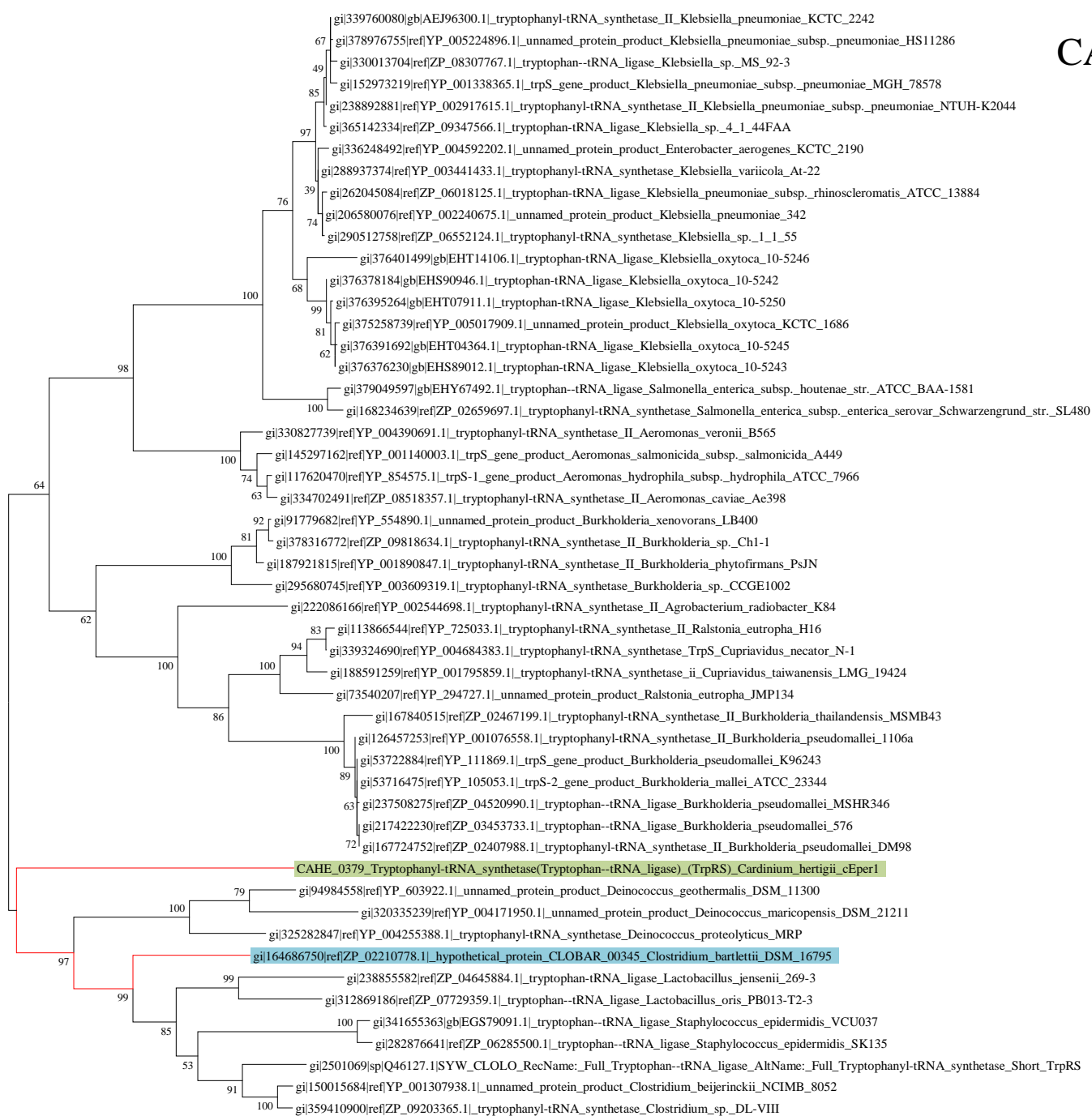

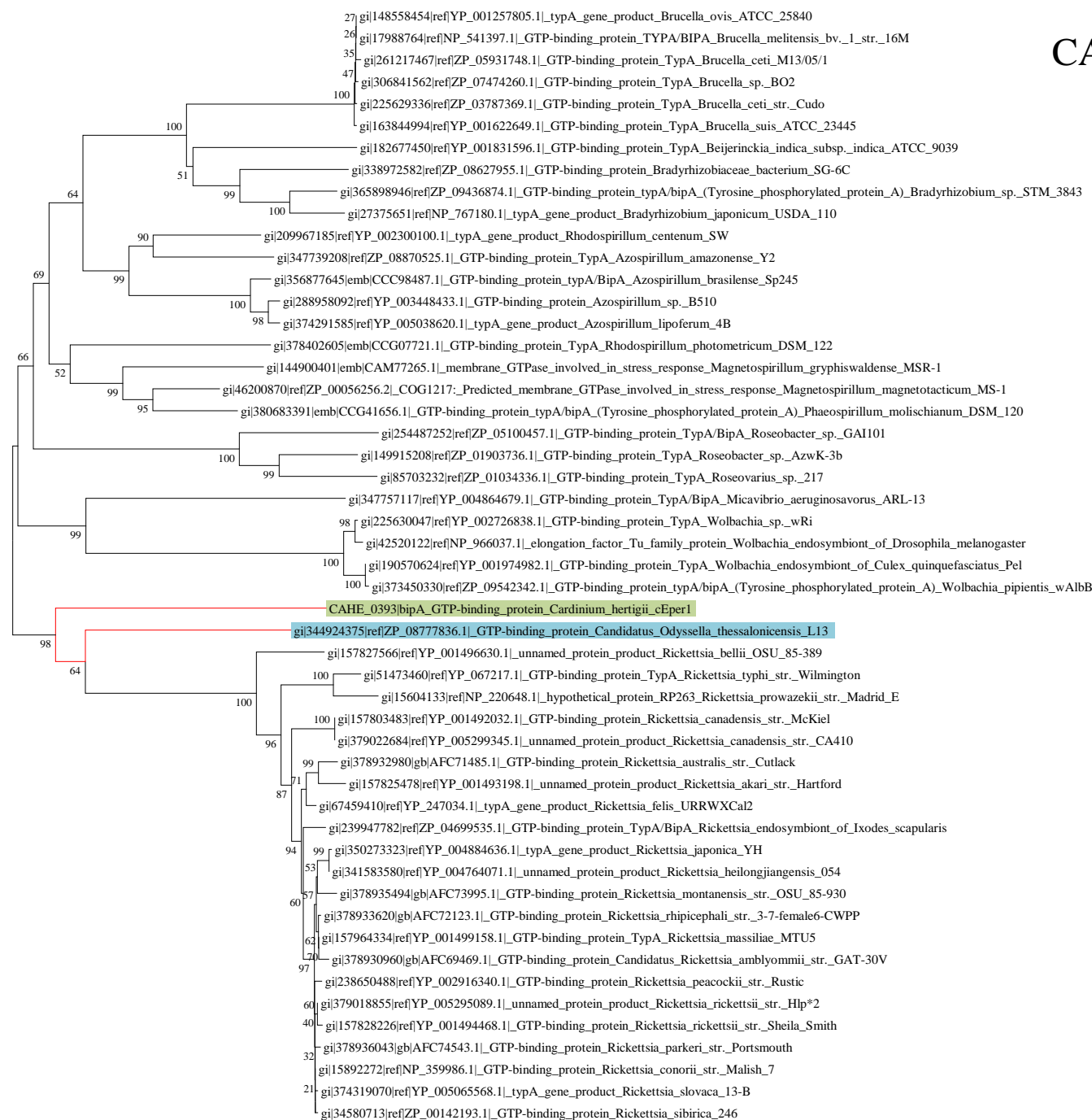

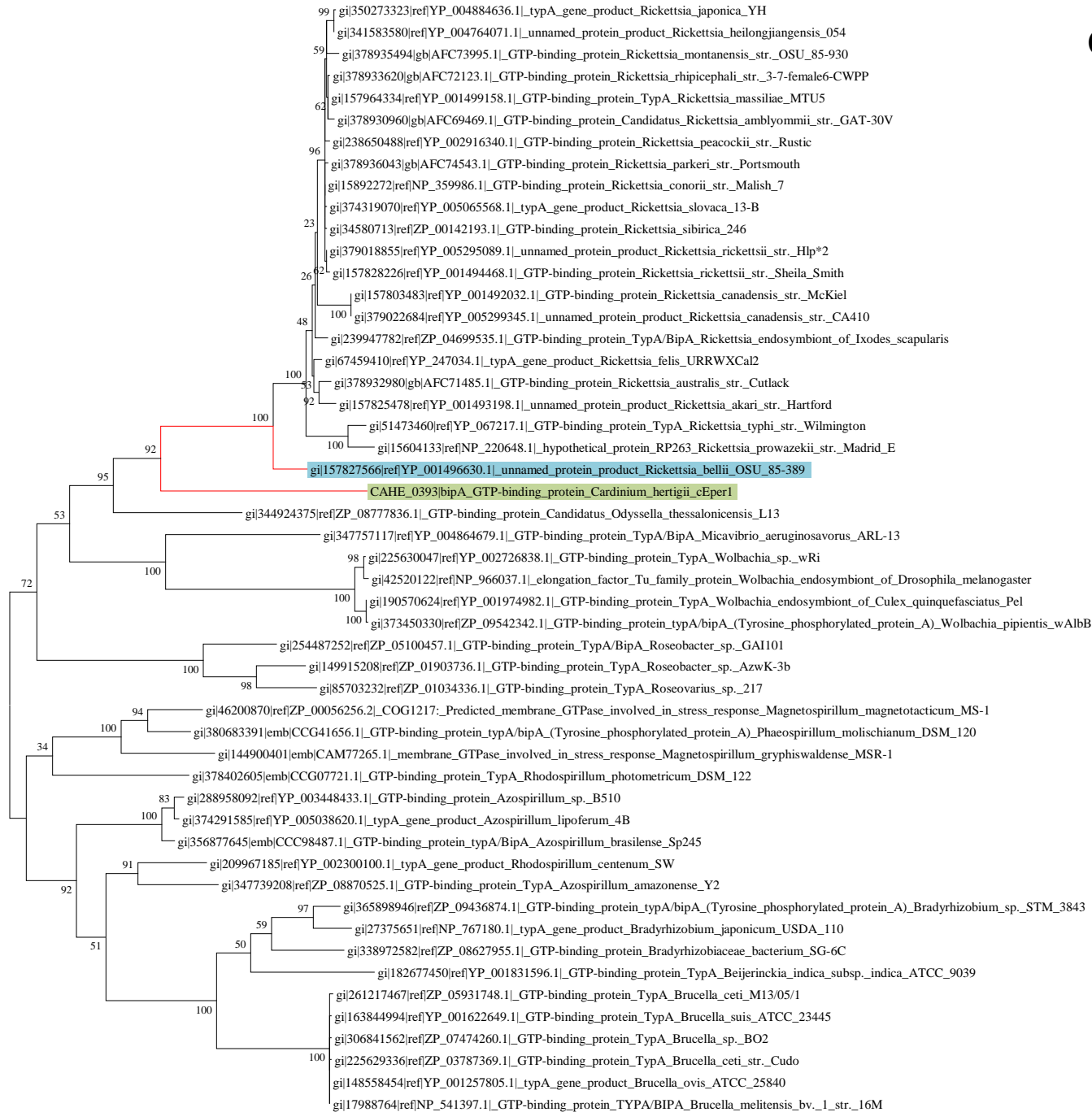

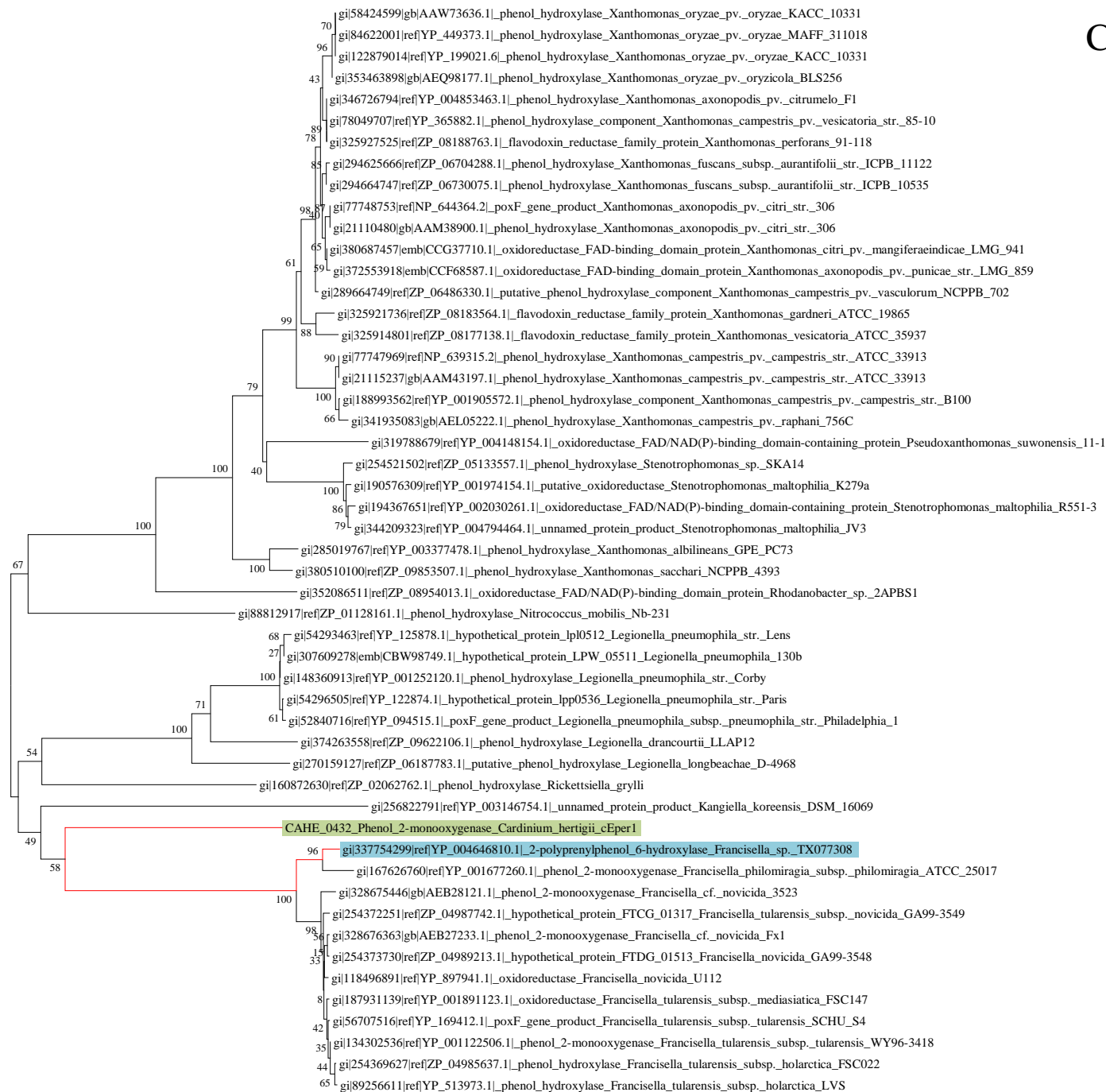

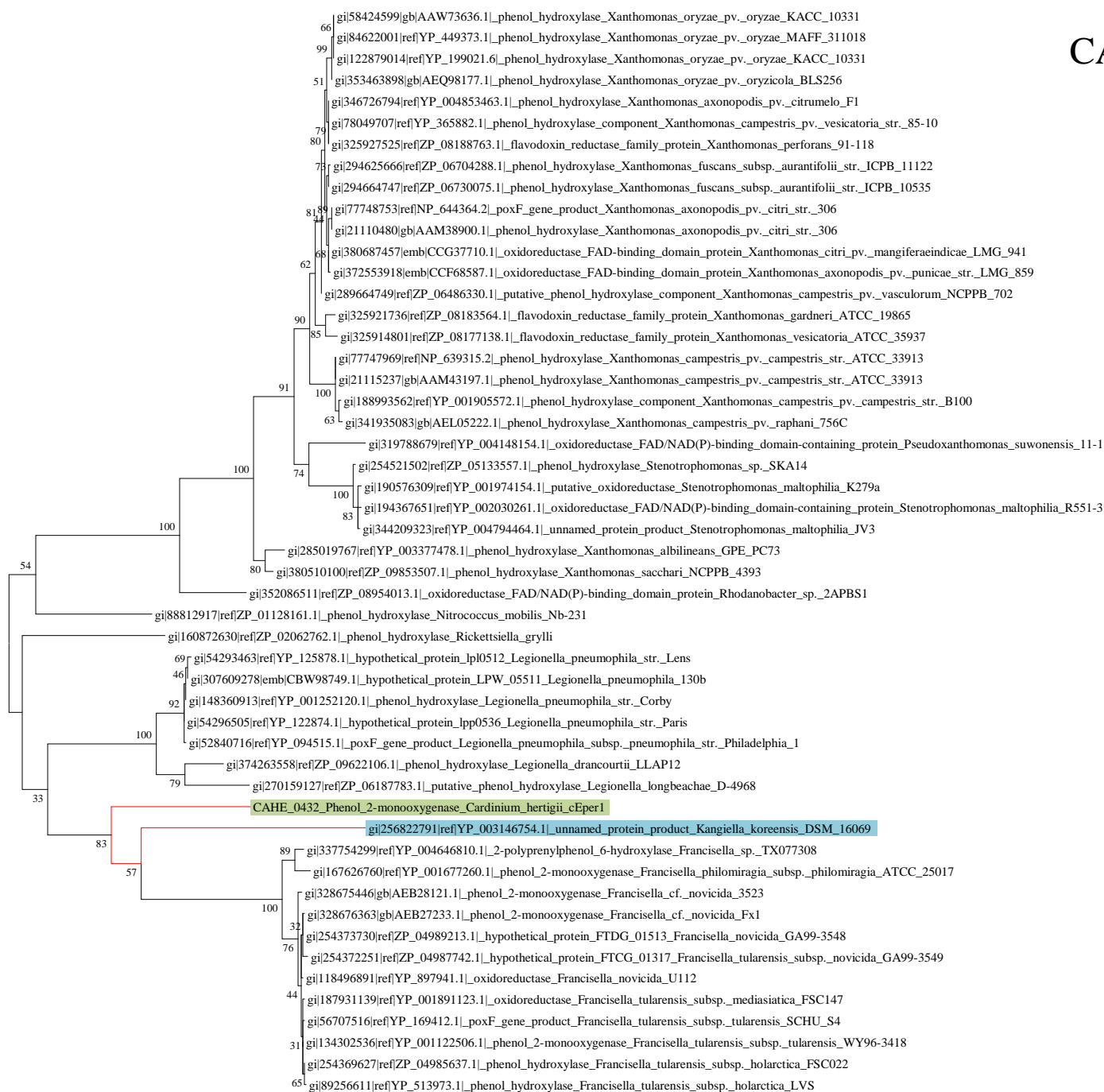

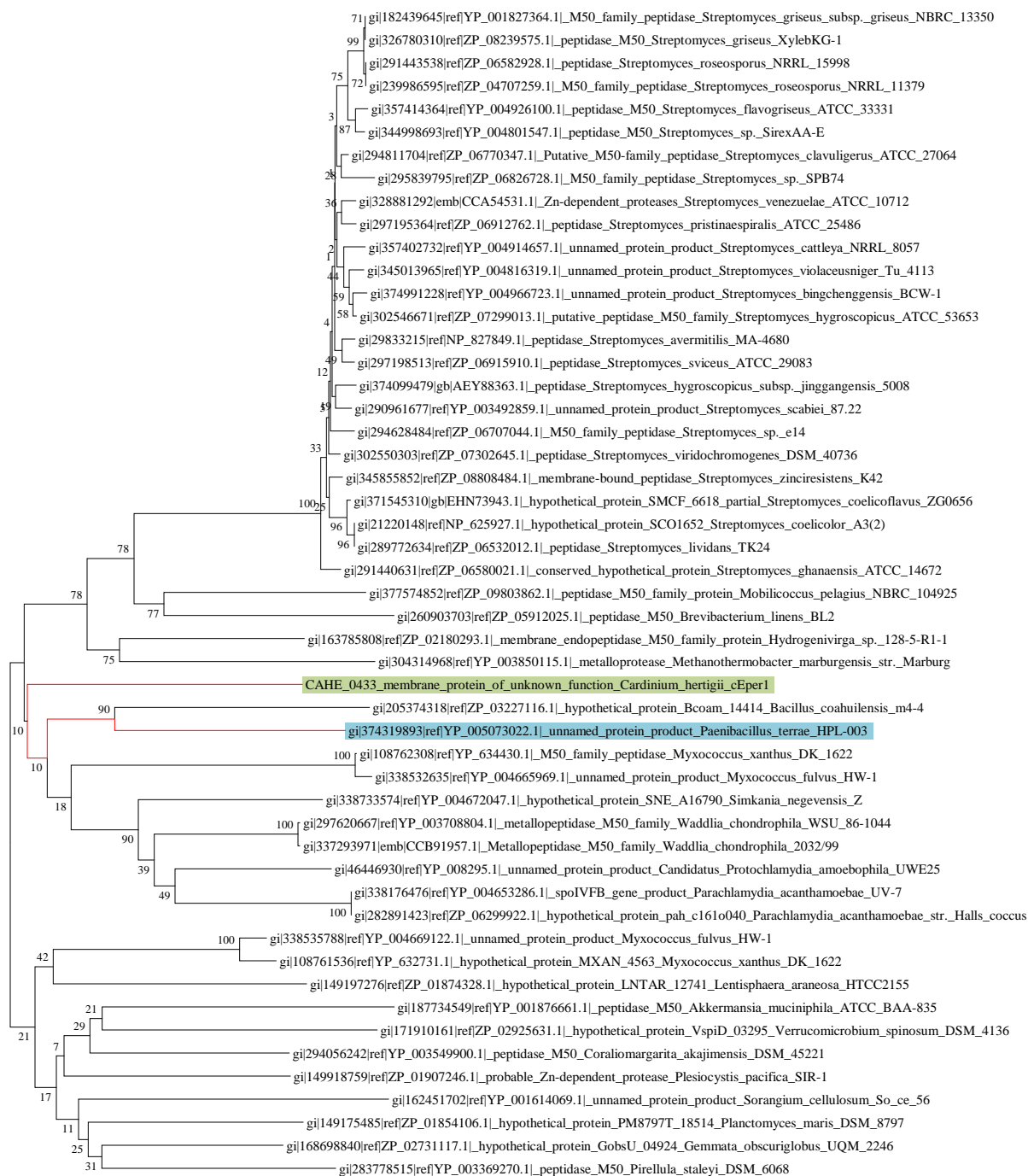

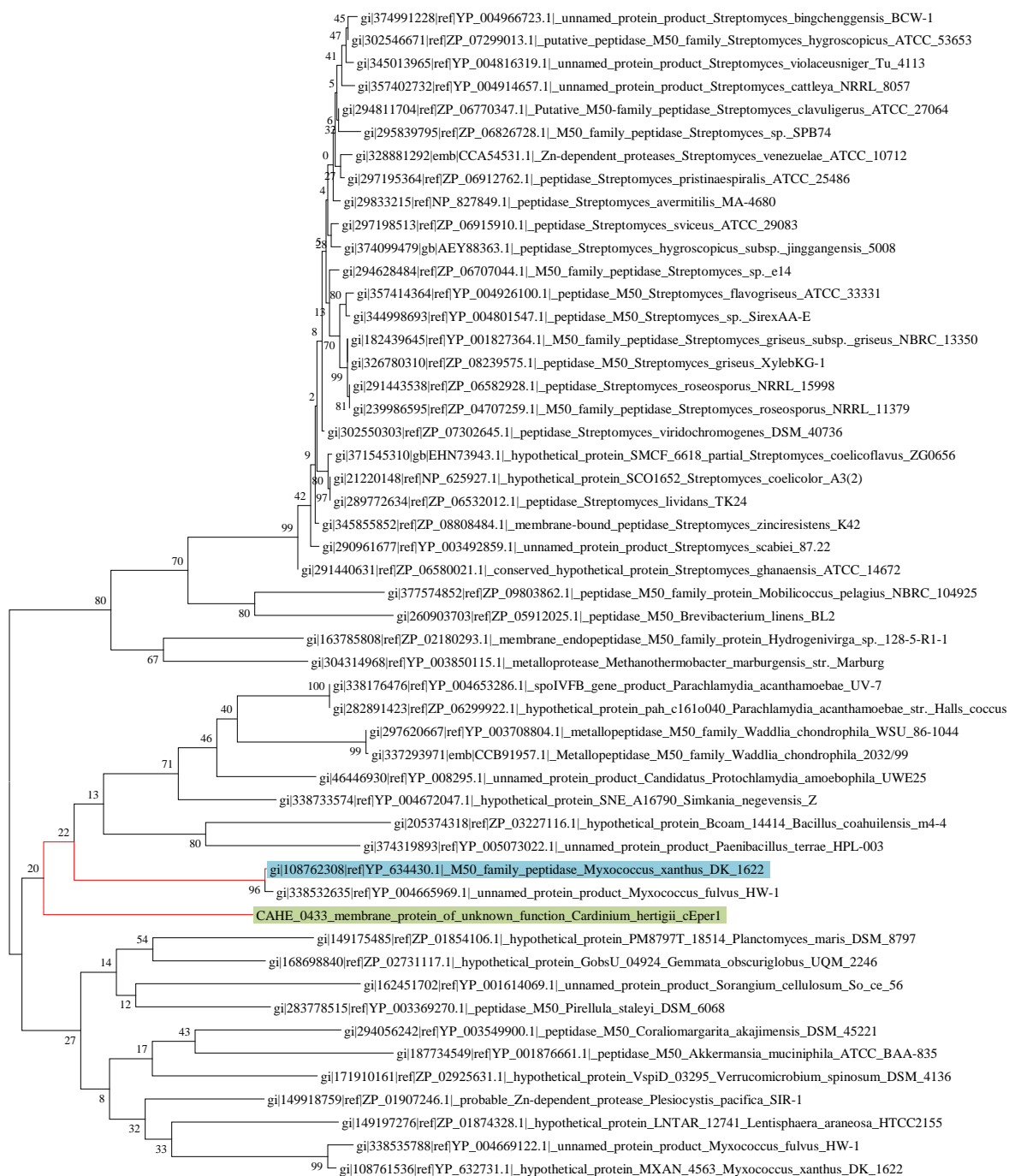

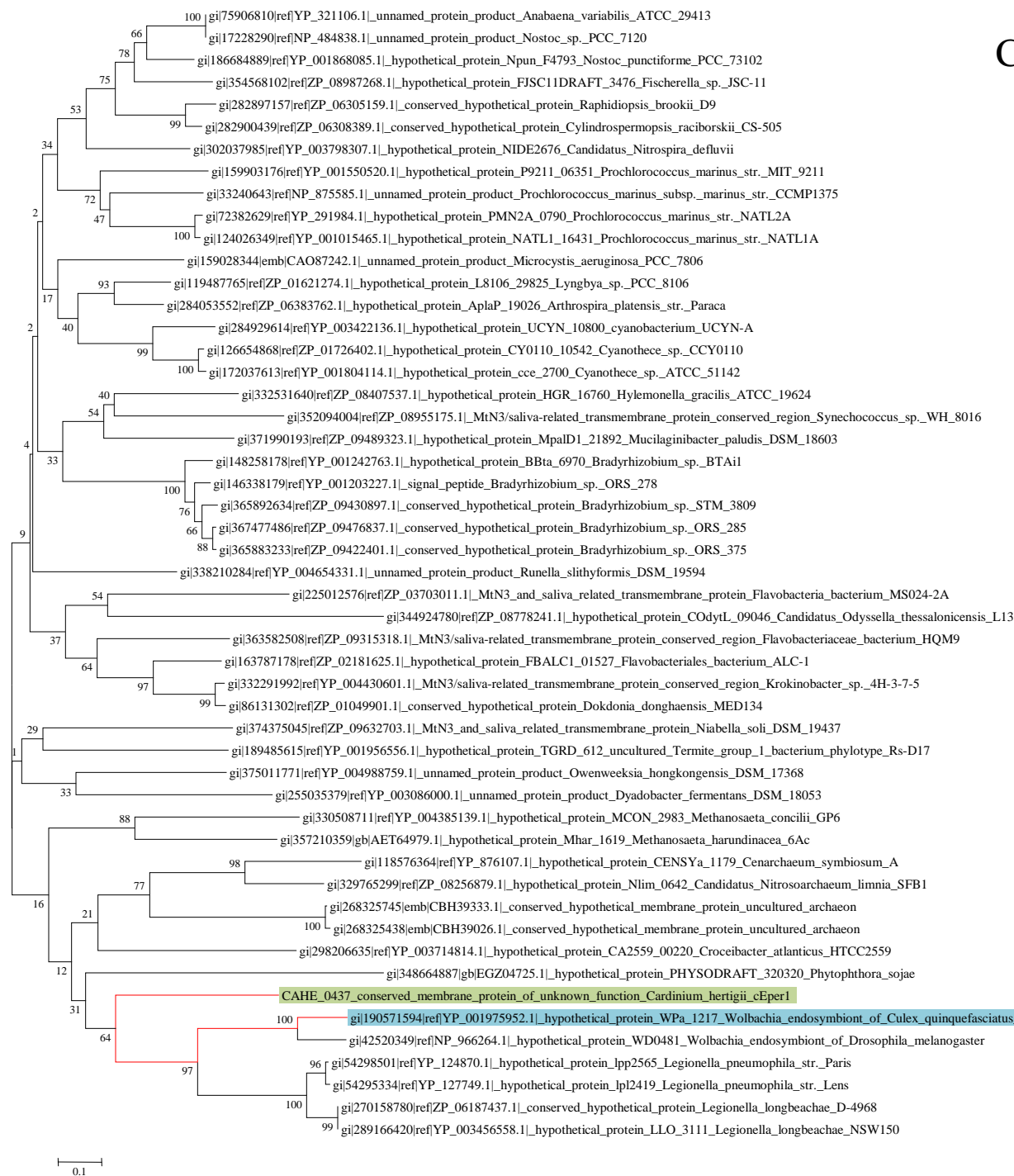

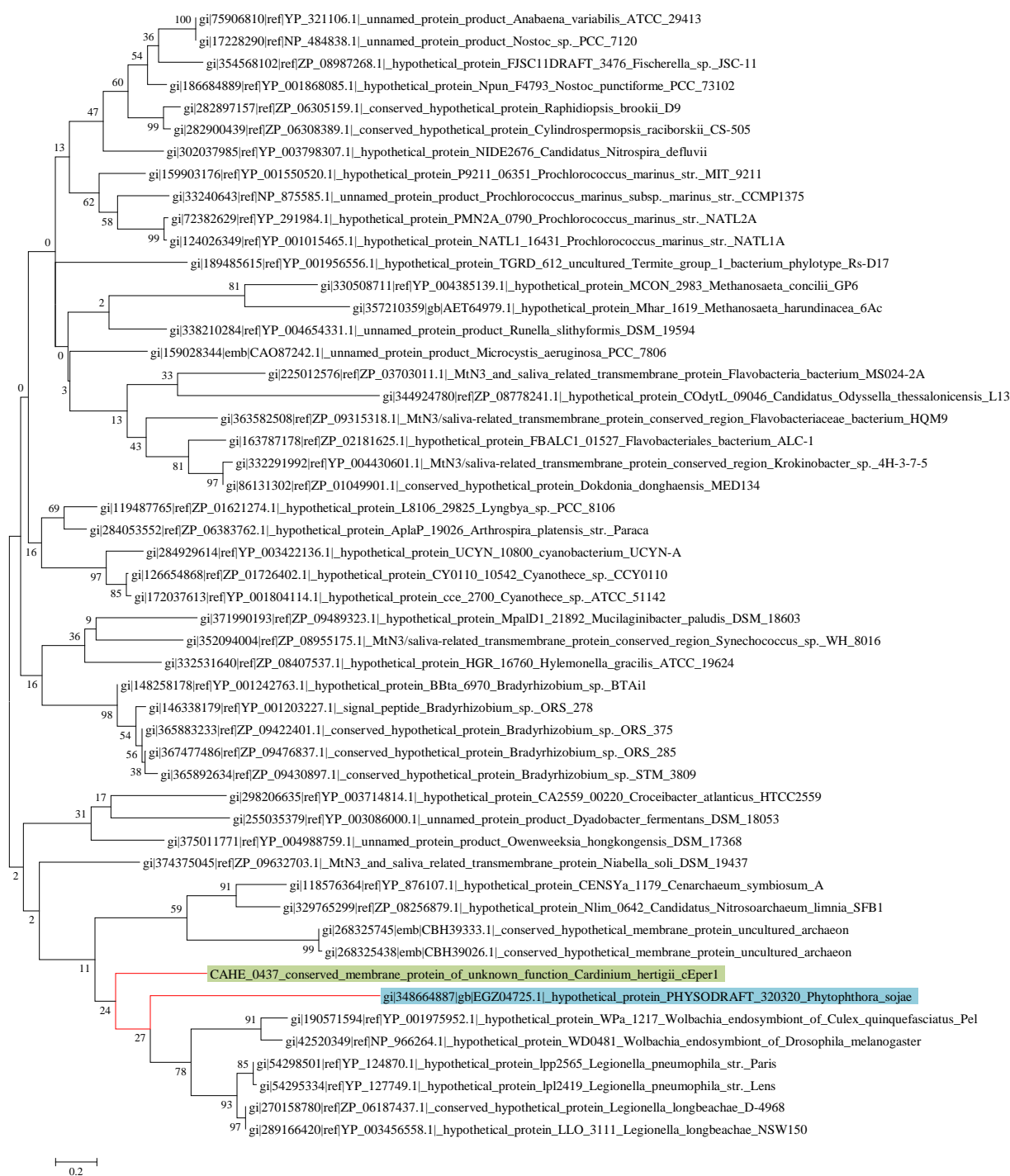

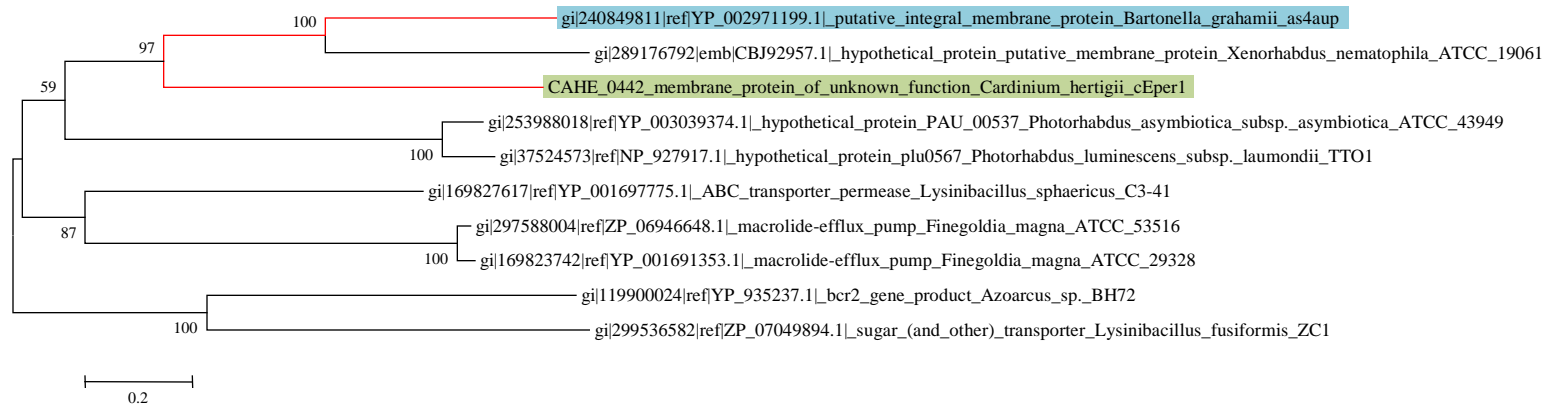

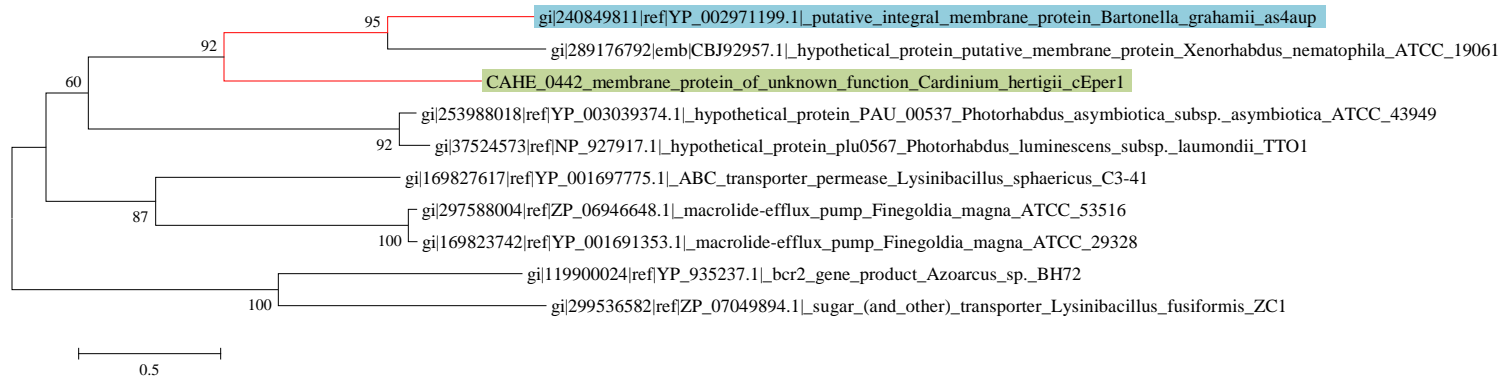

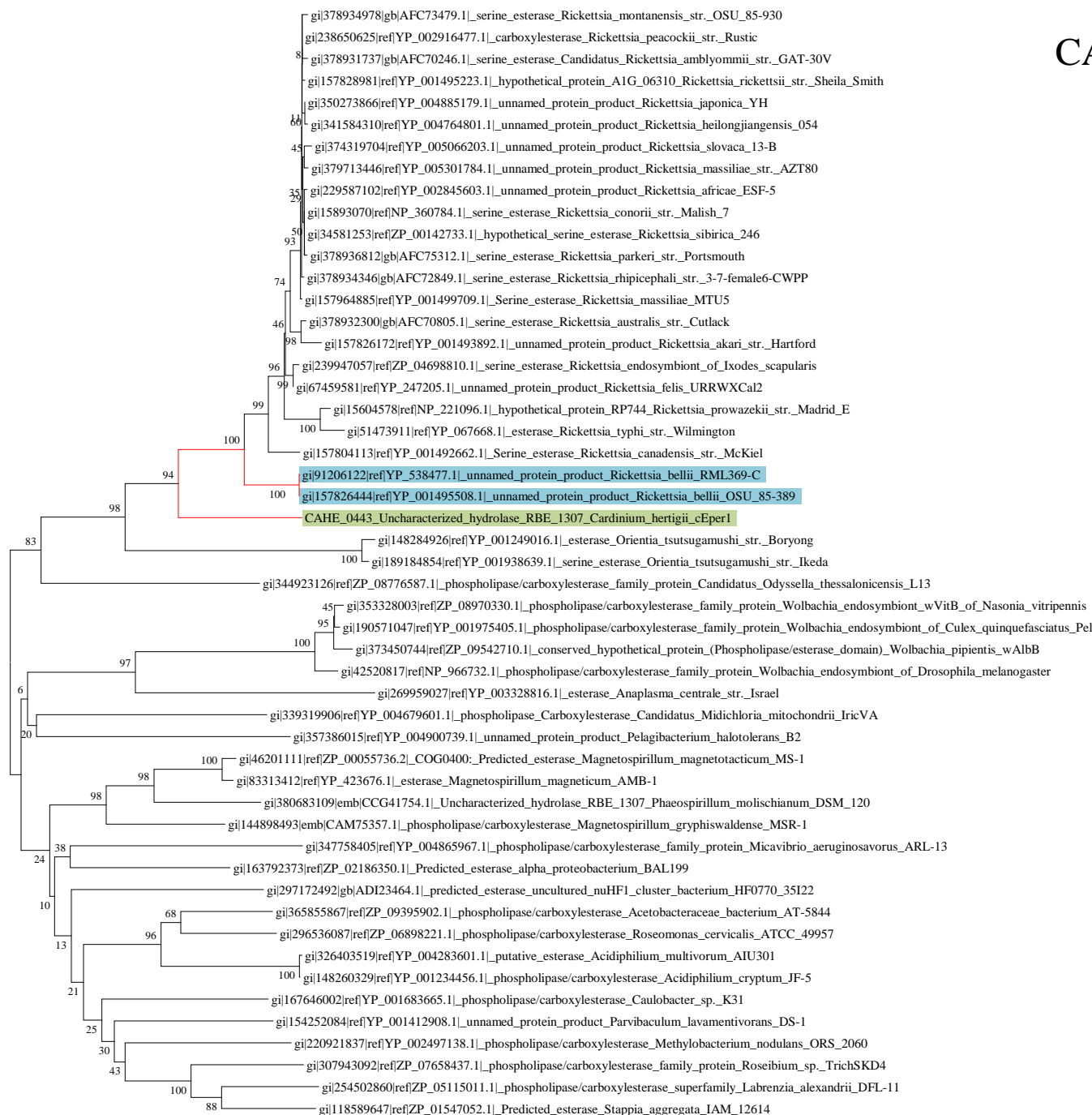

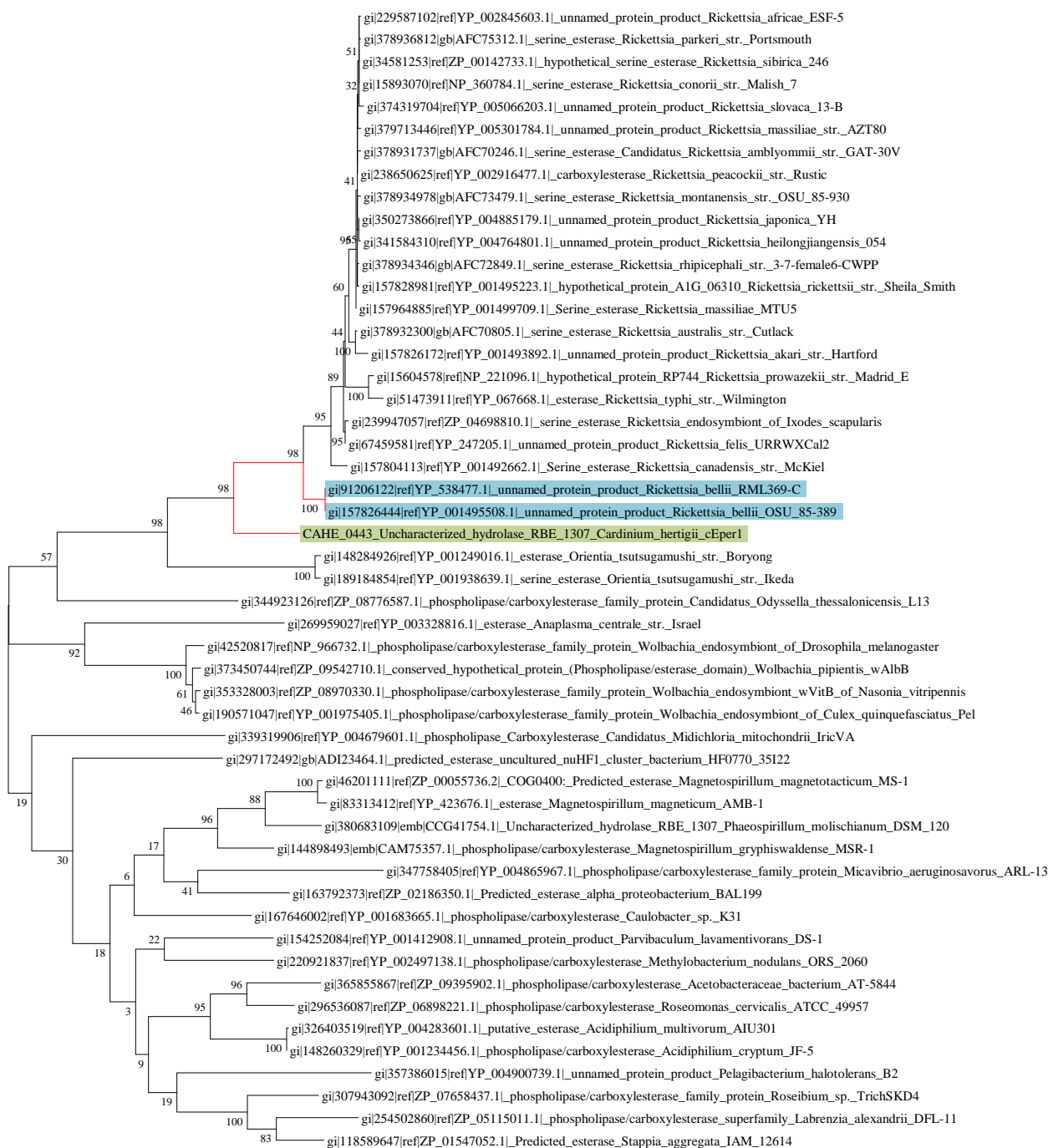

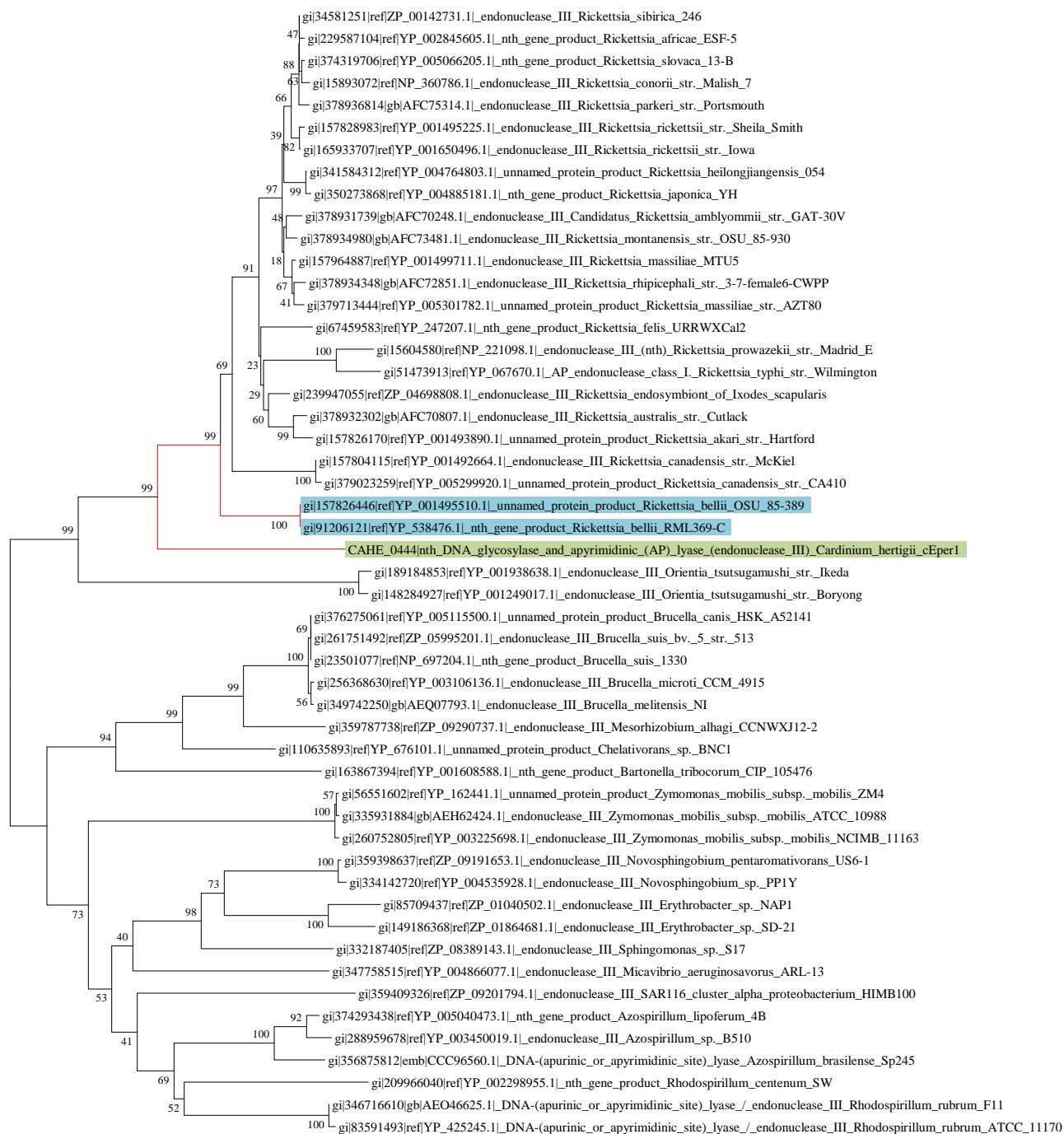

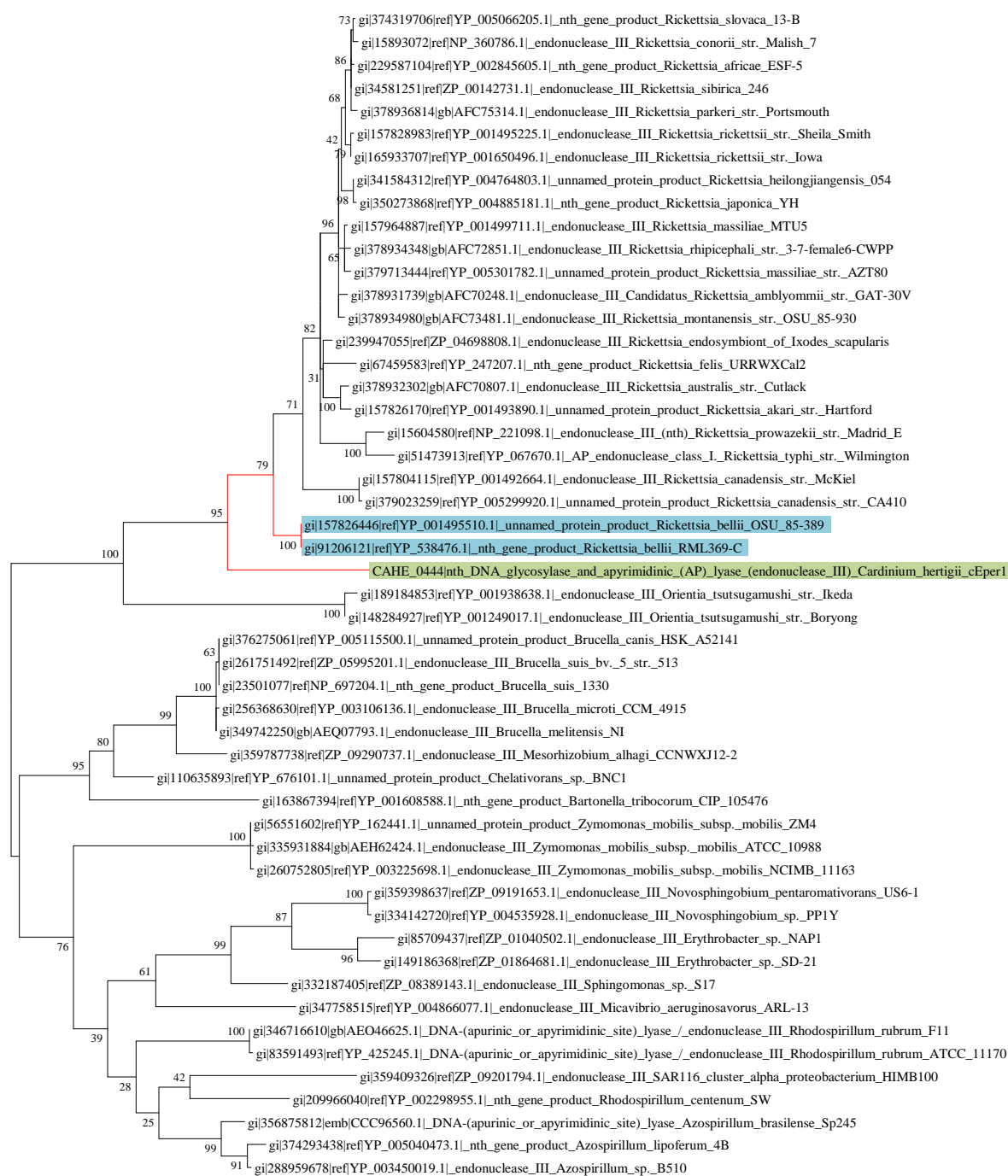

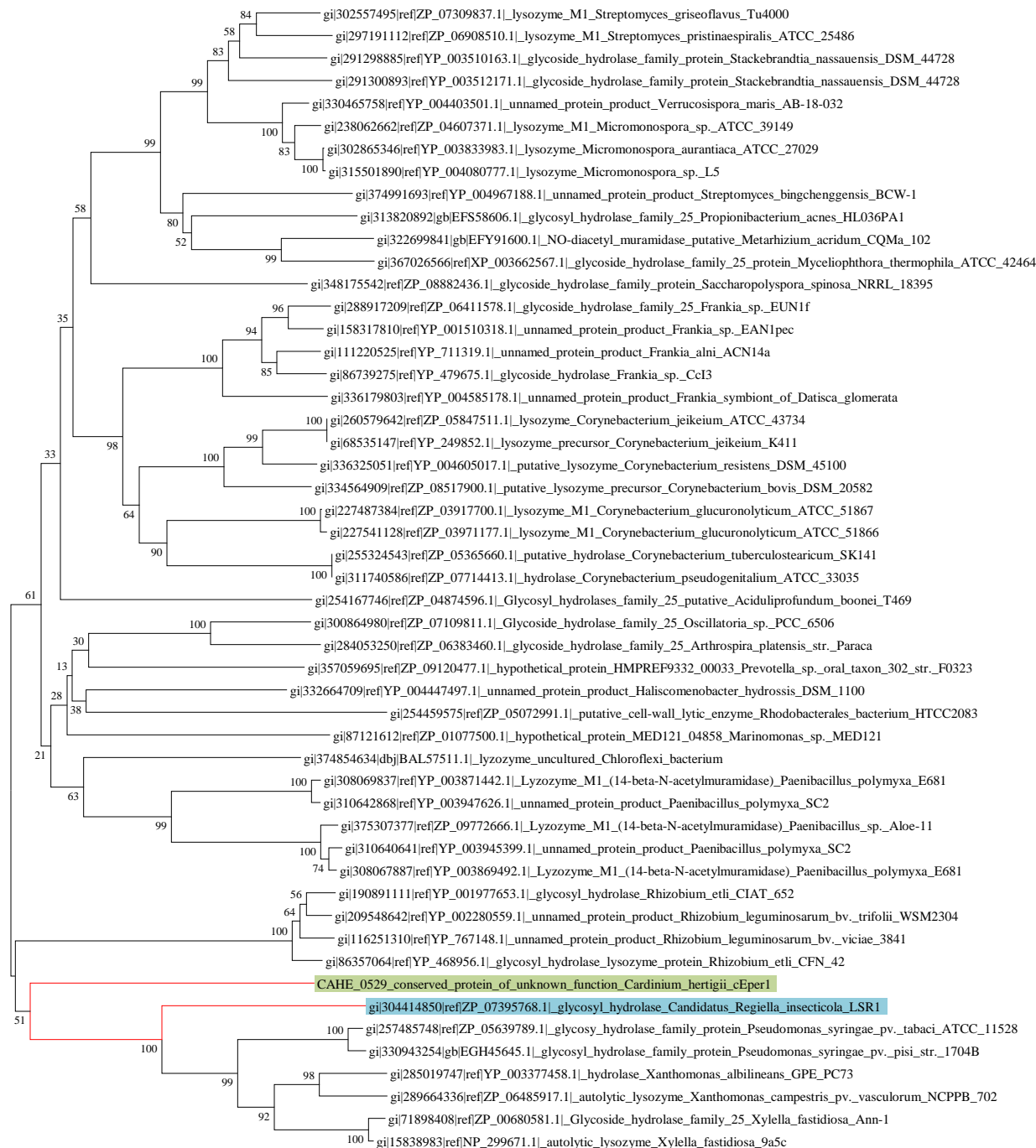

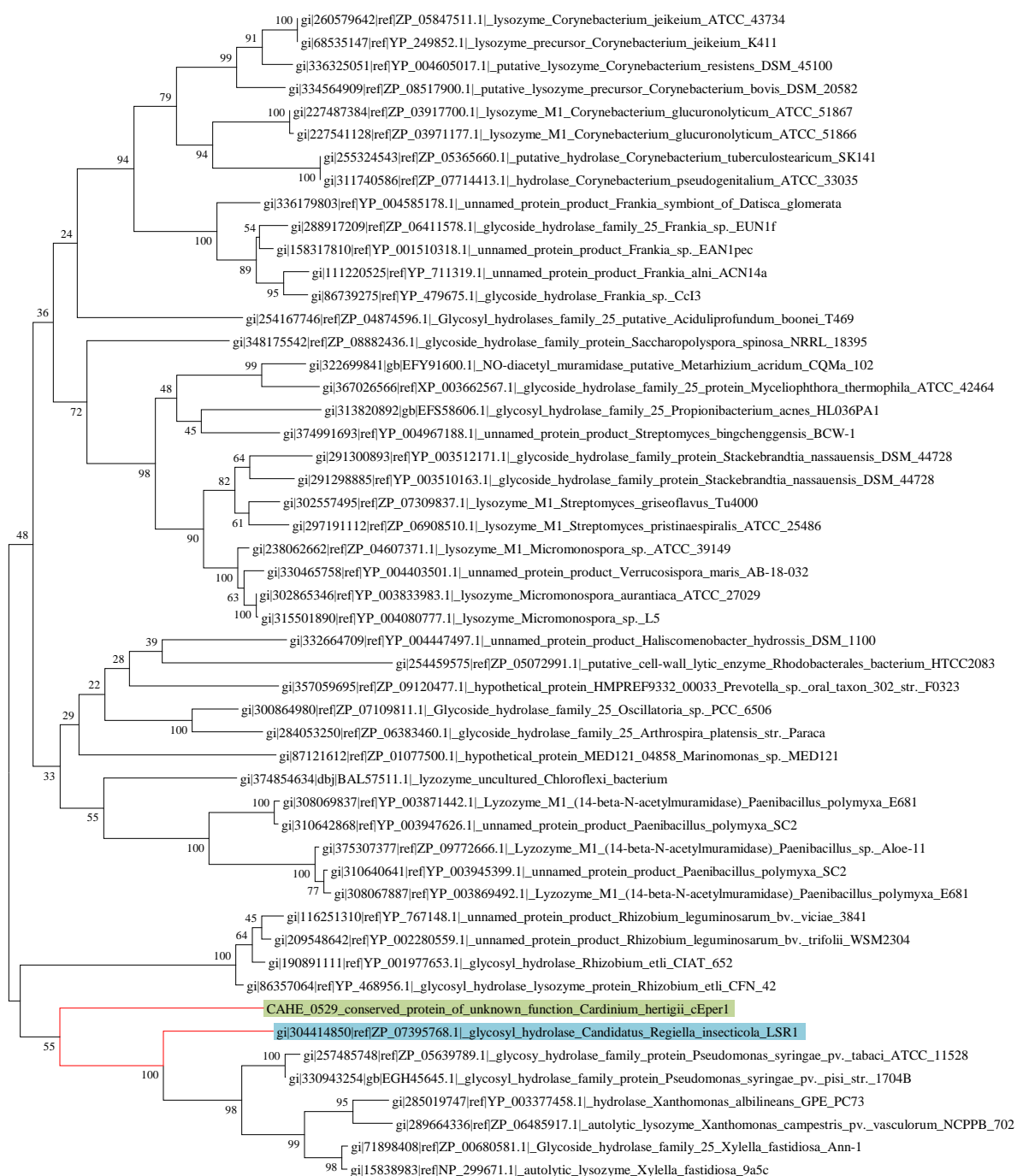

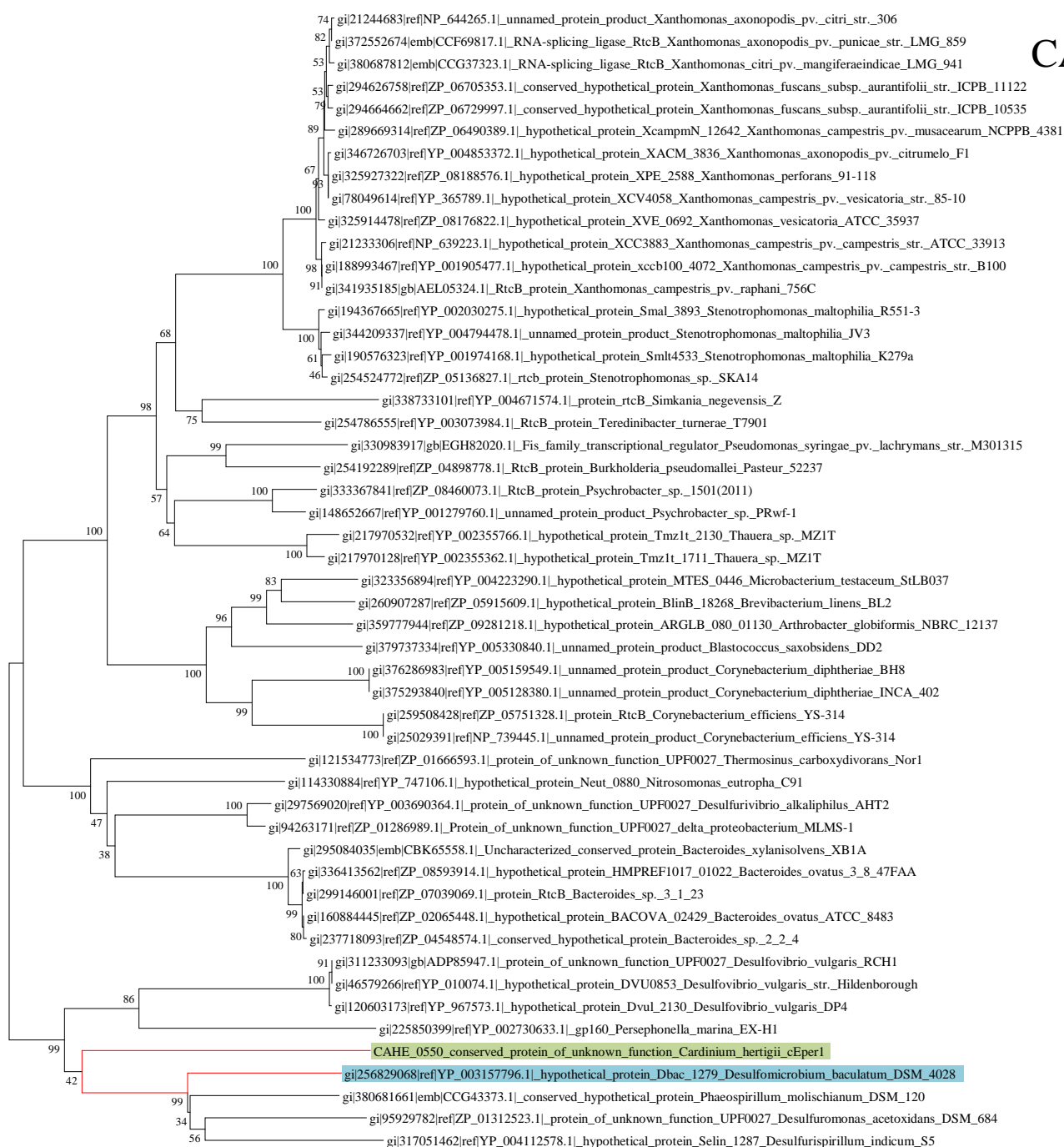

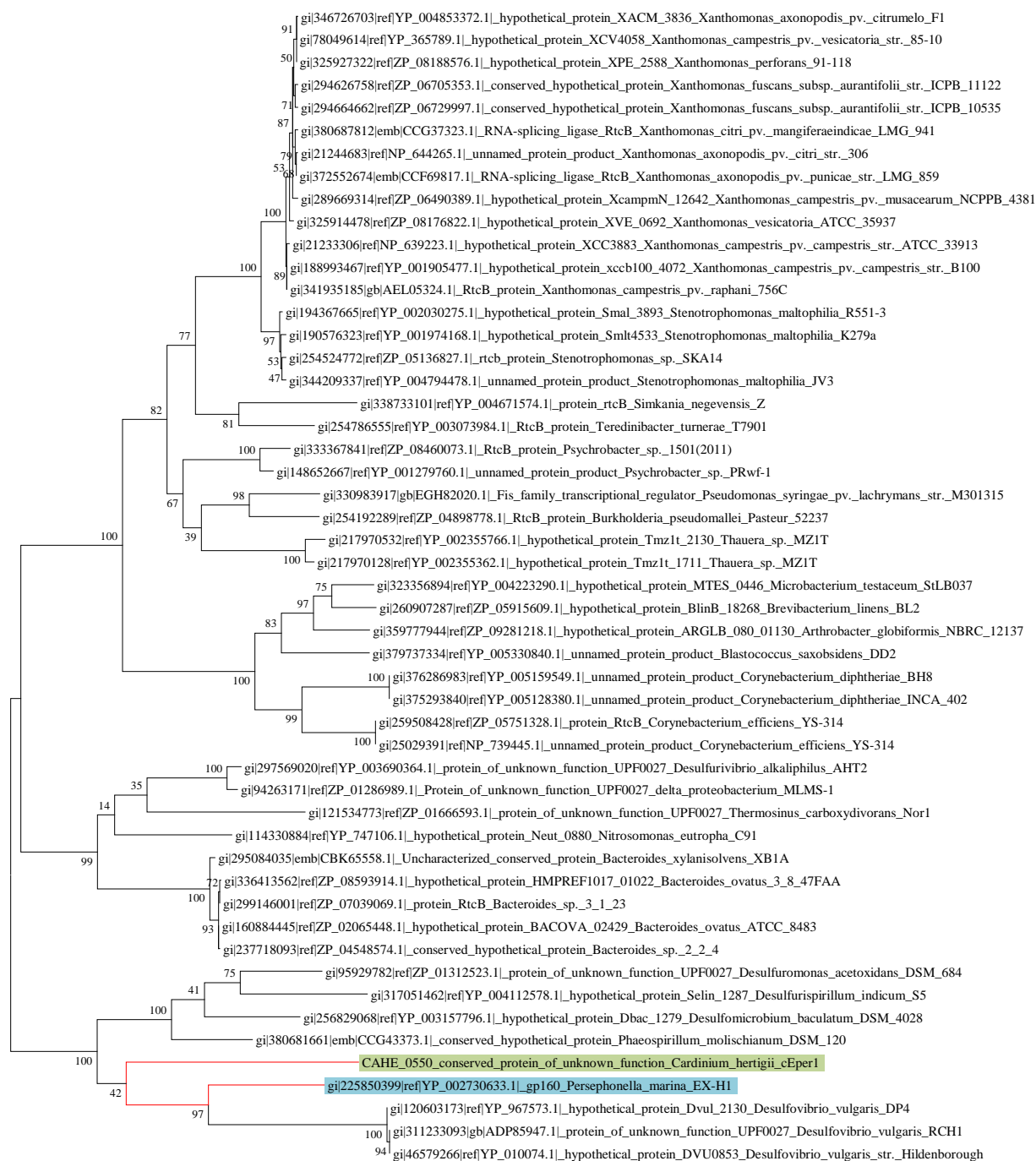

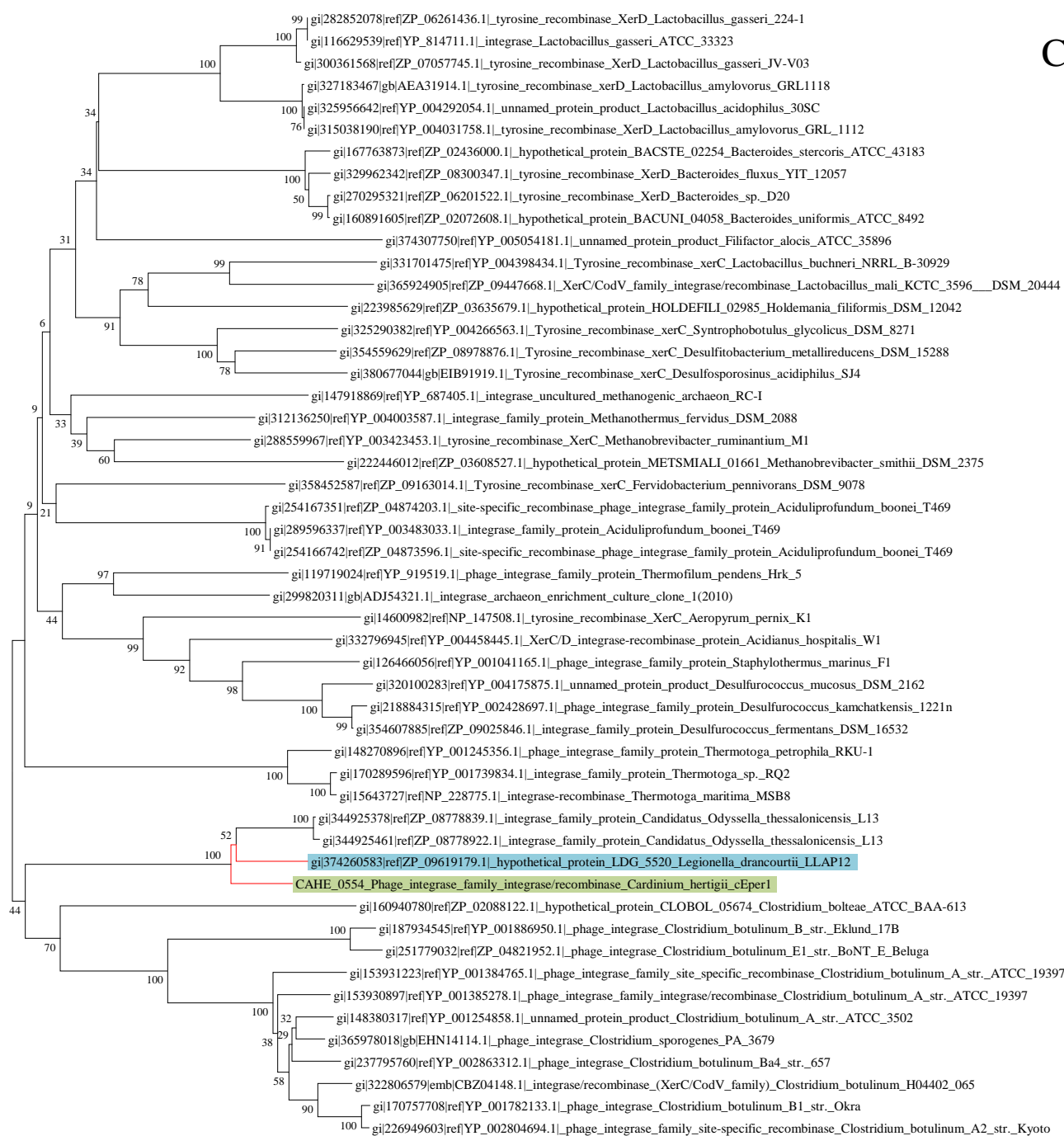

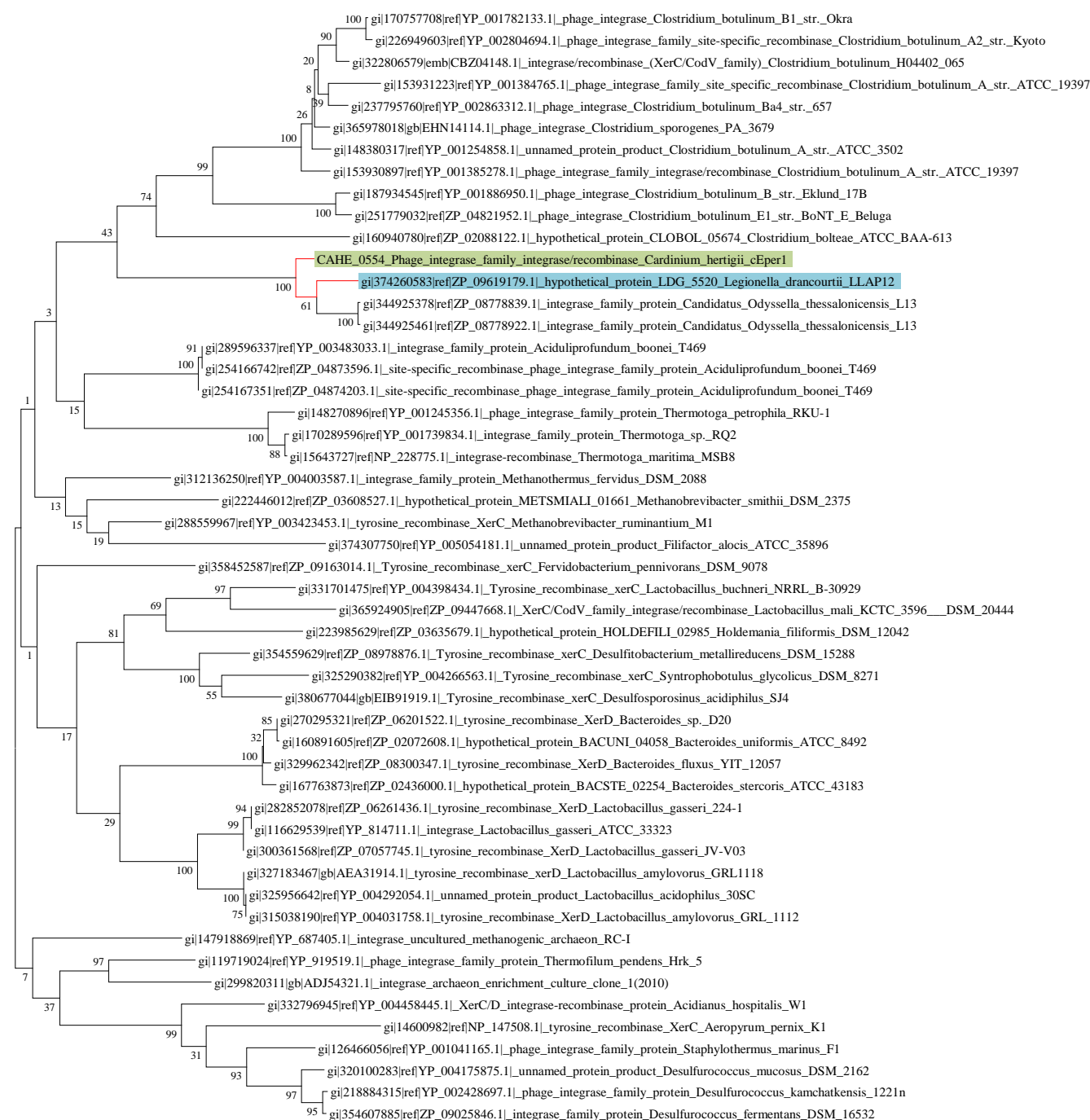

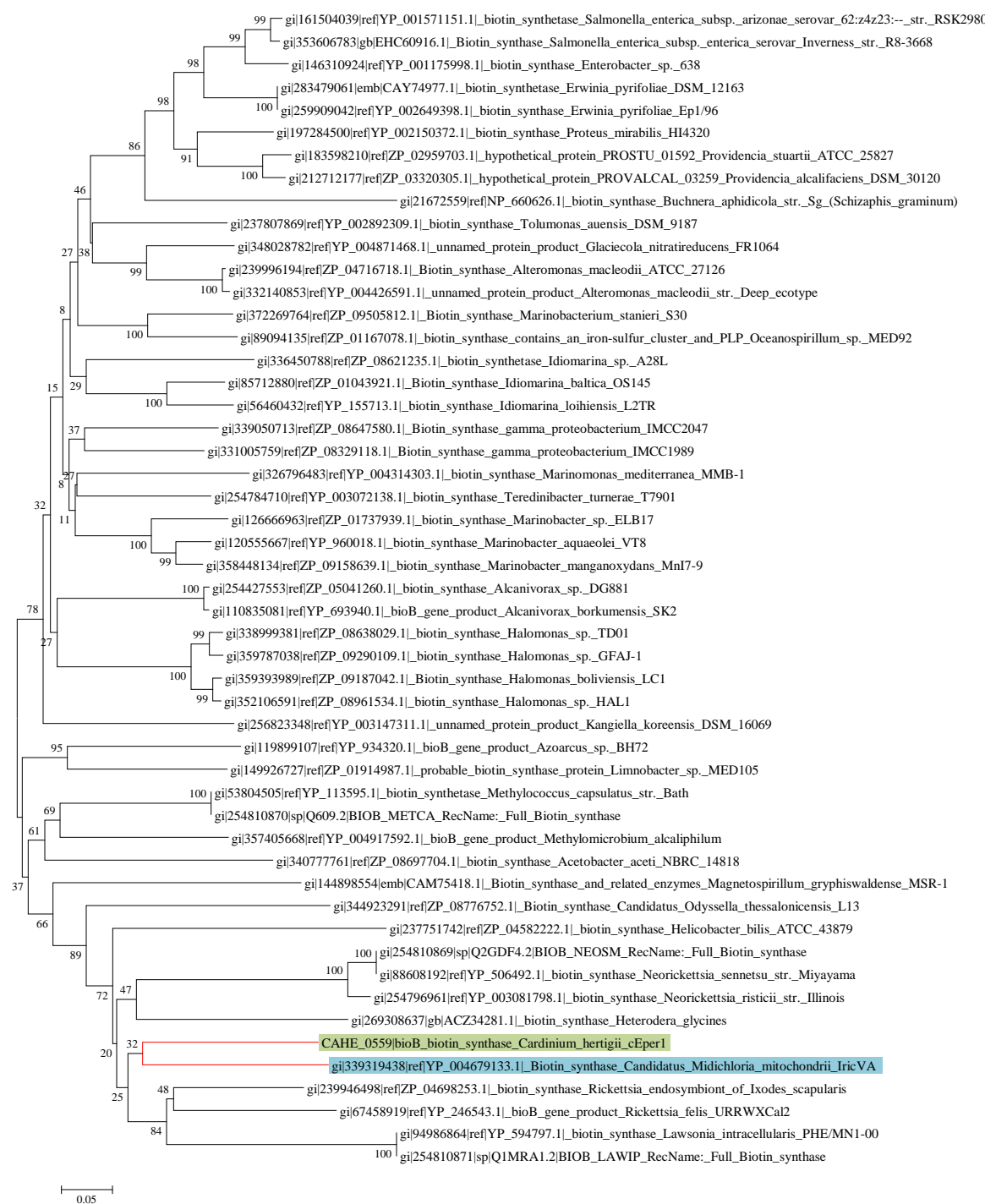

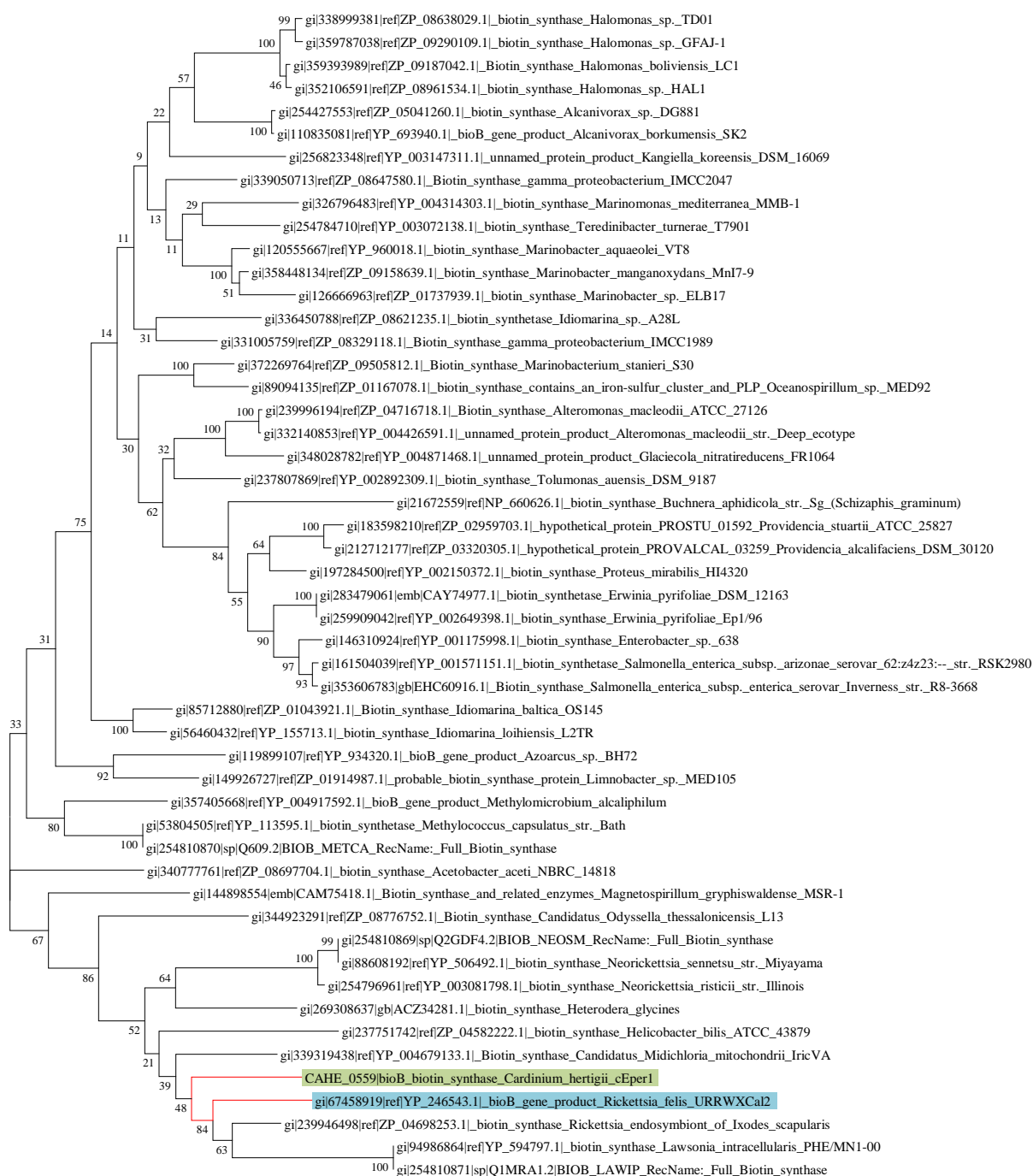

0.1

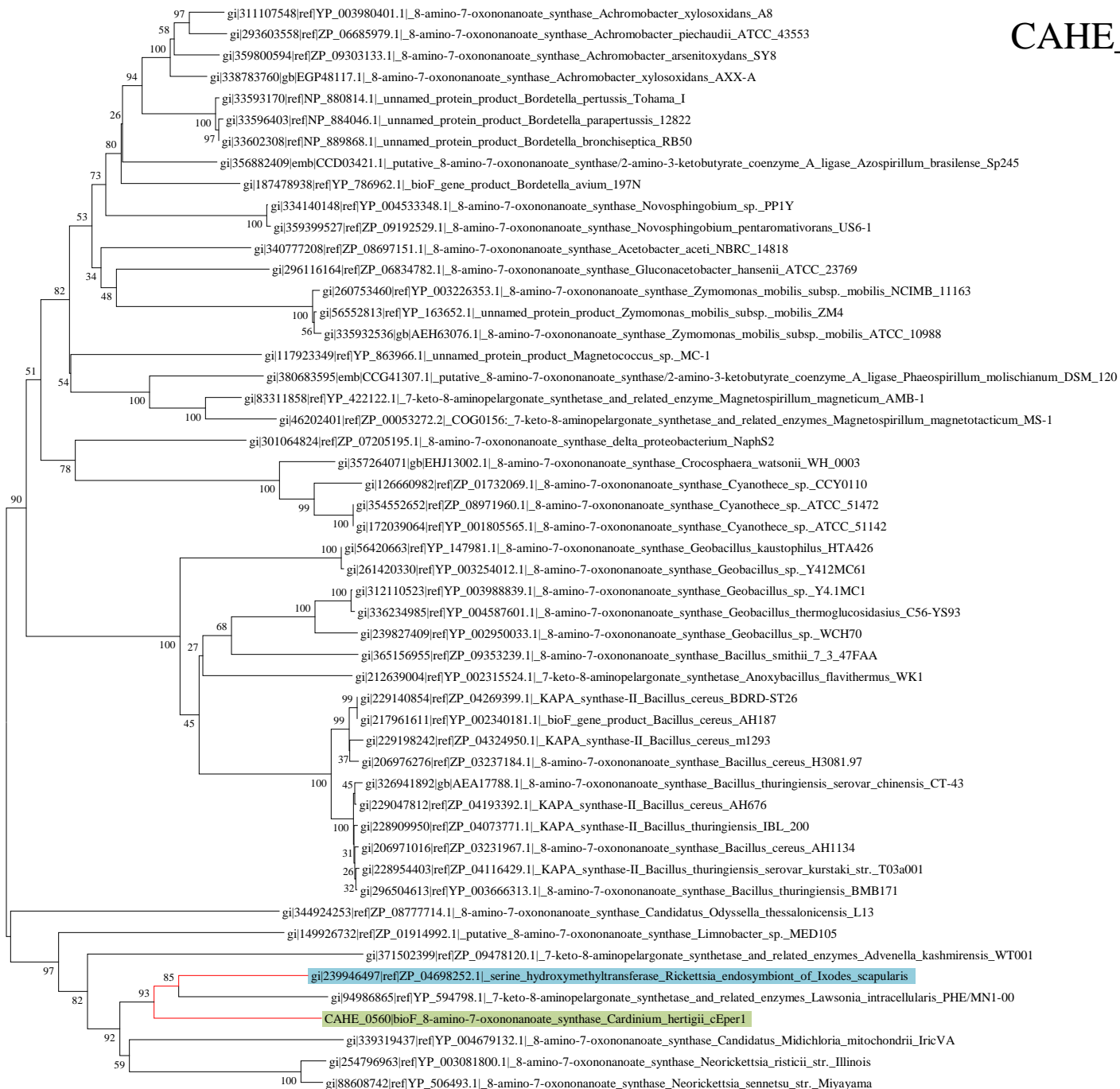

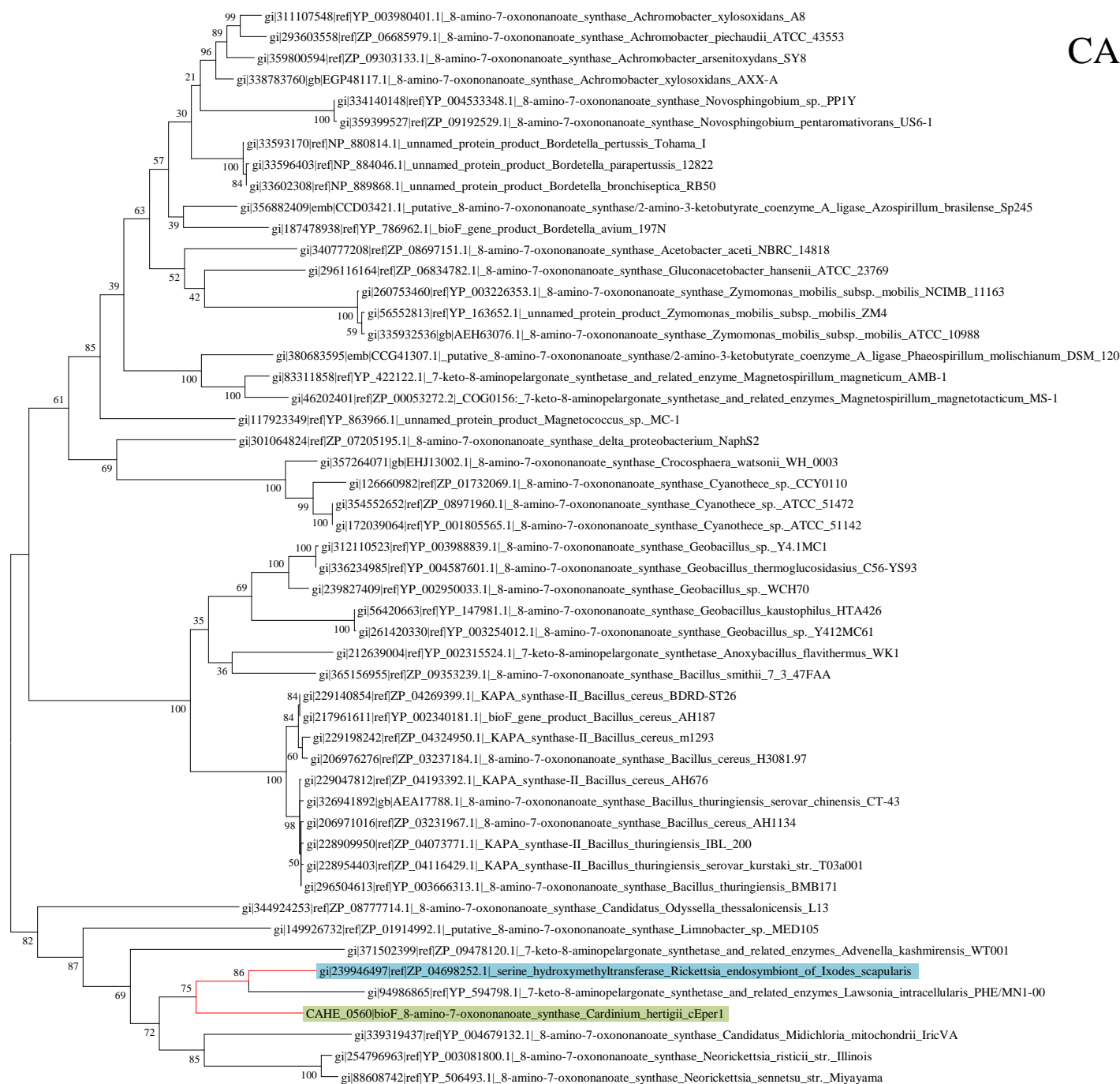

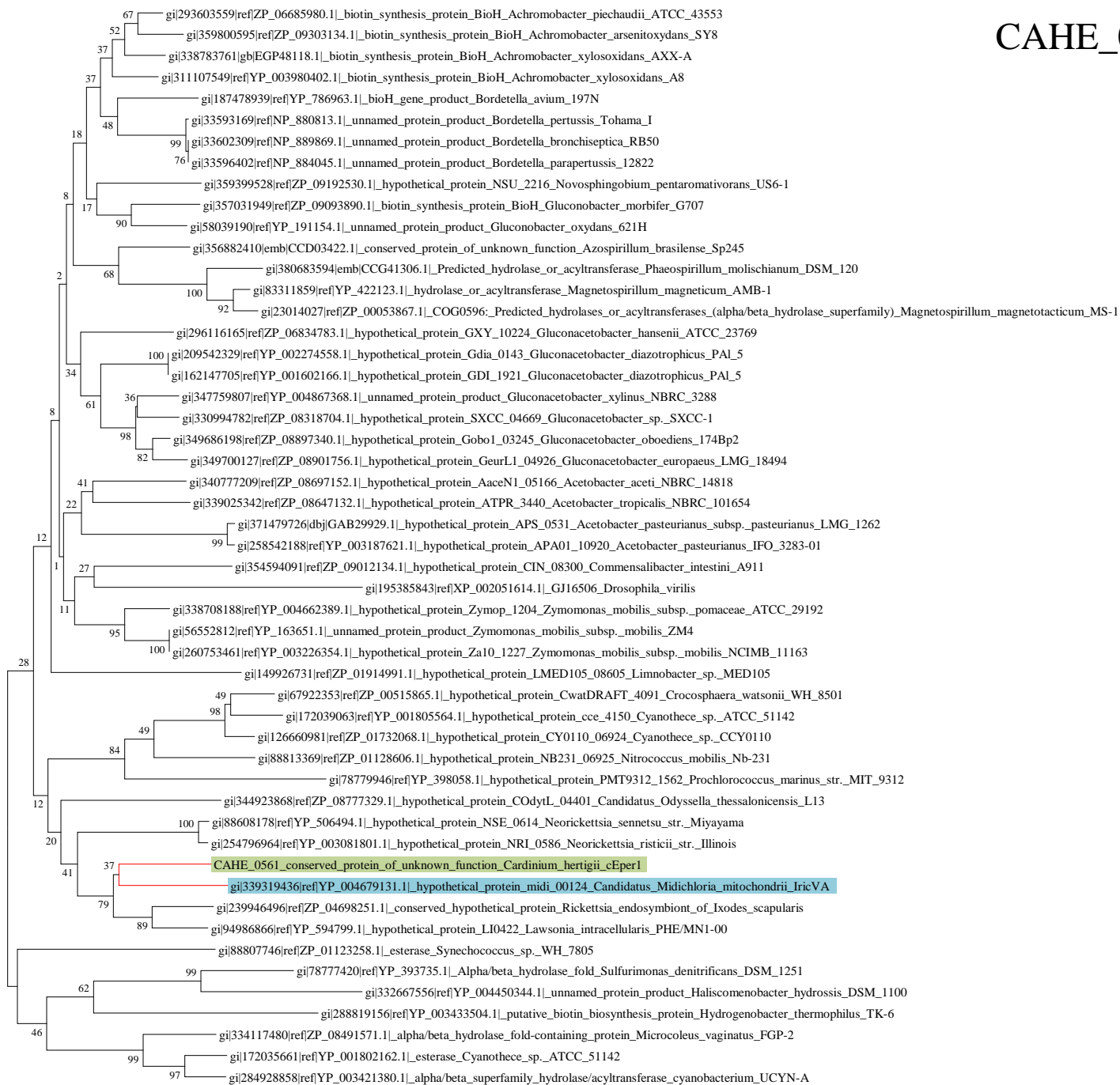

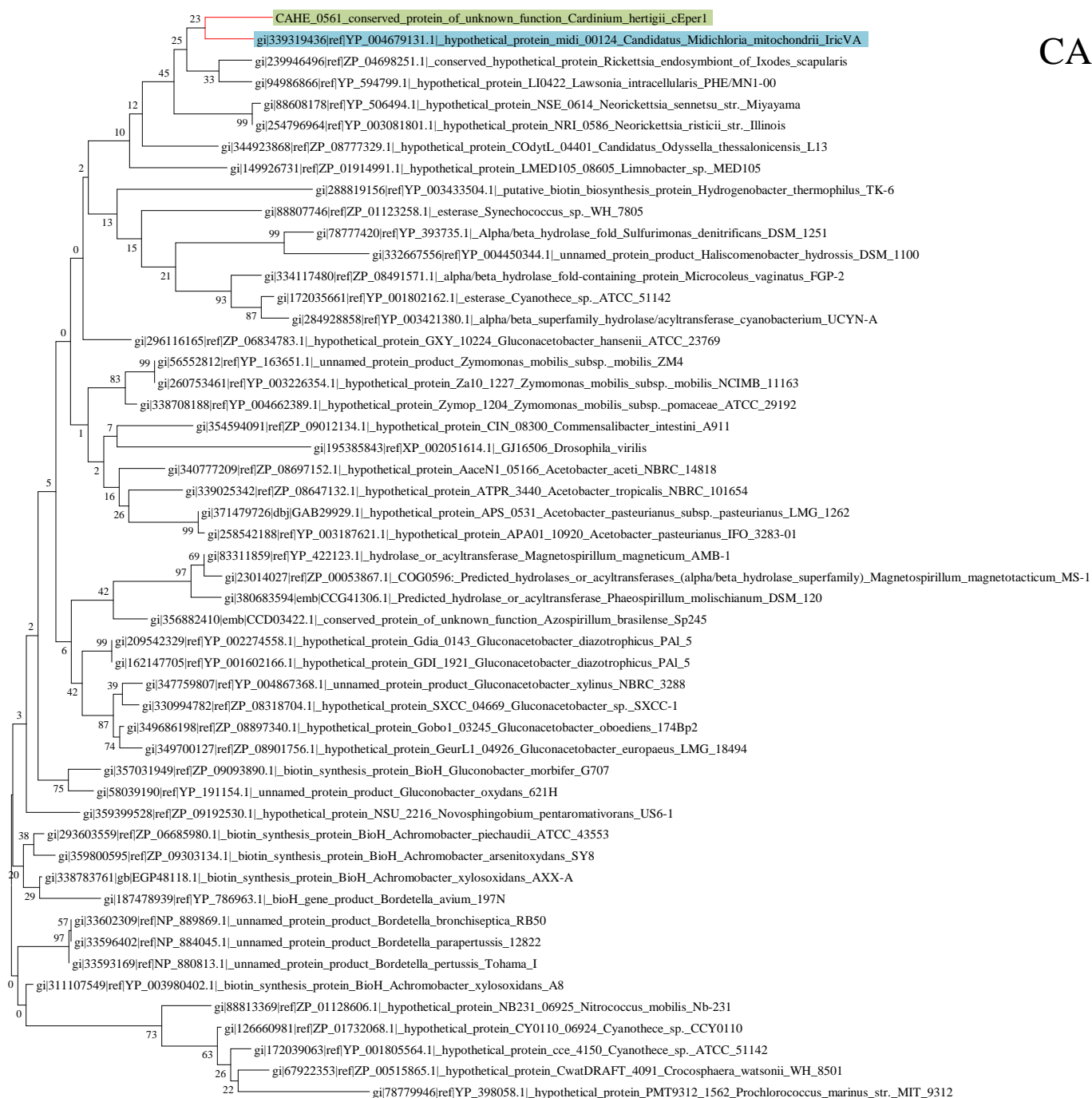

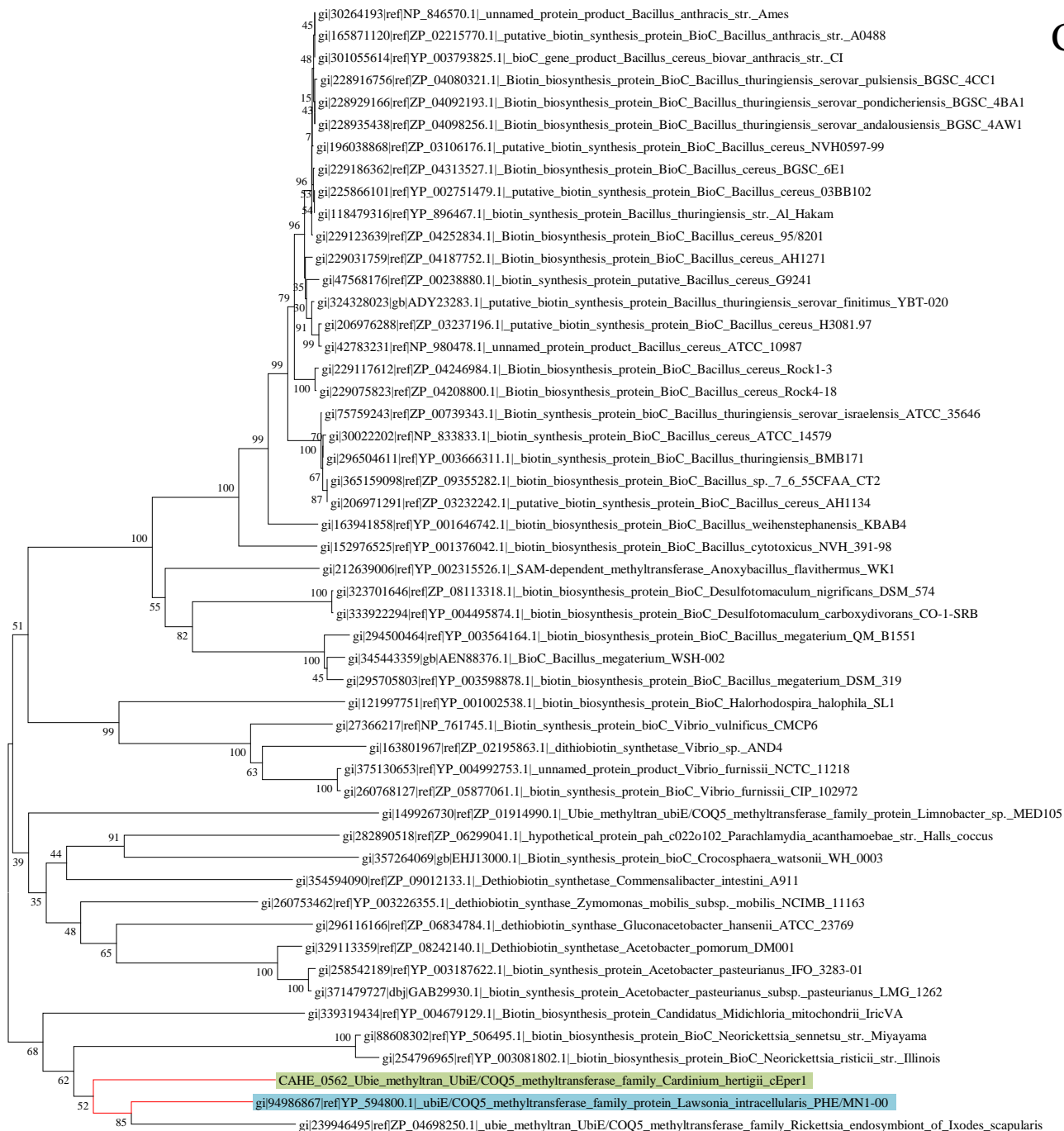

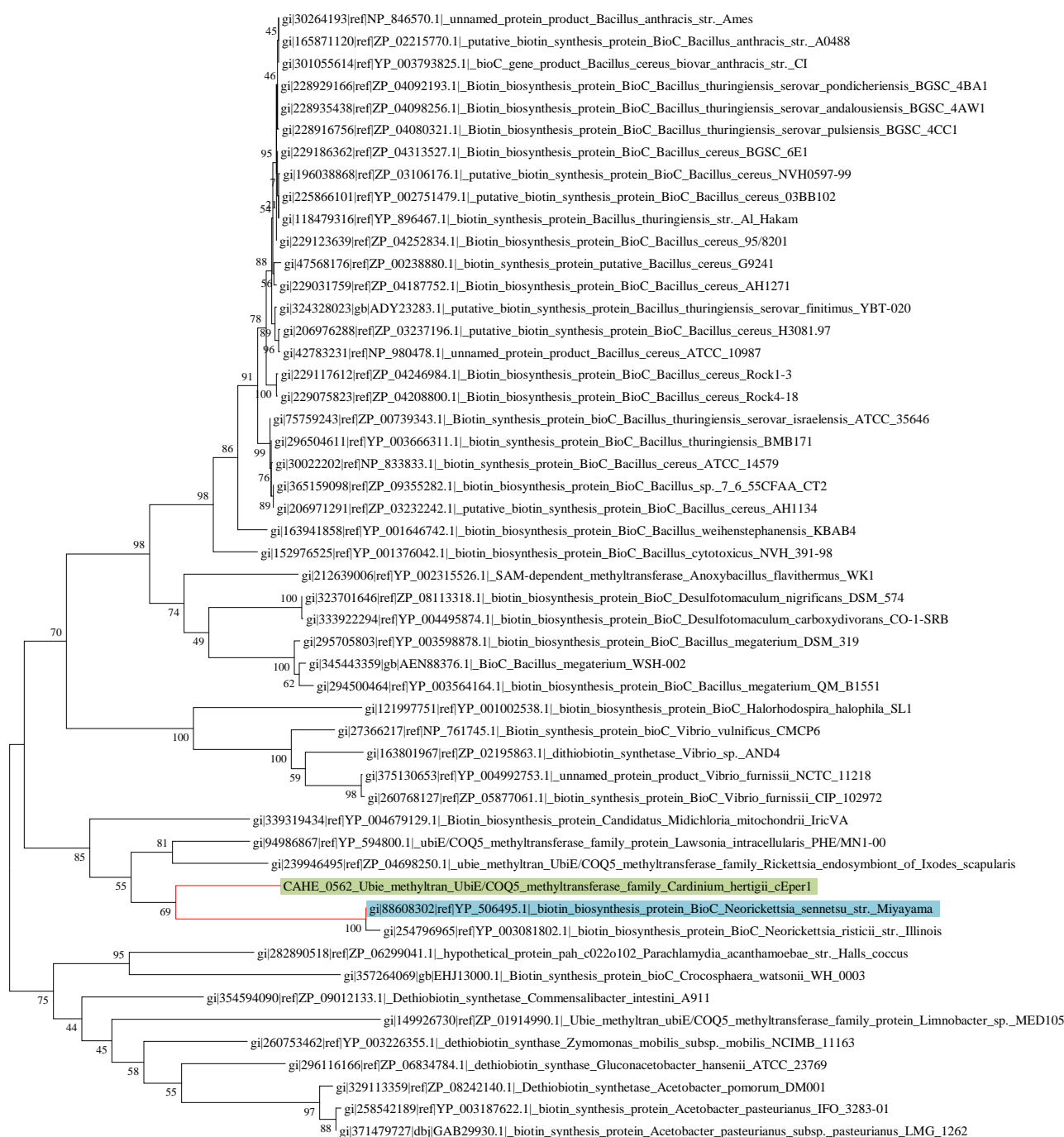

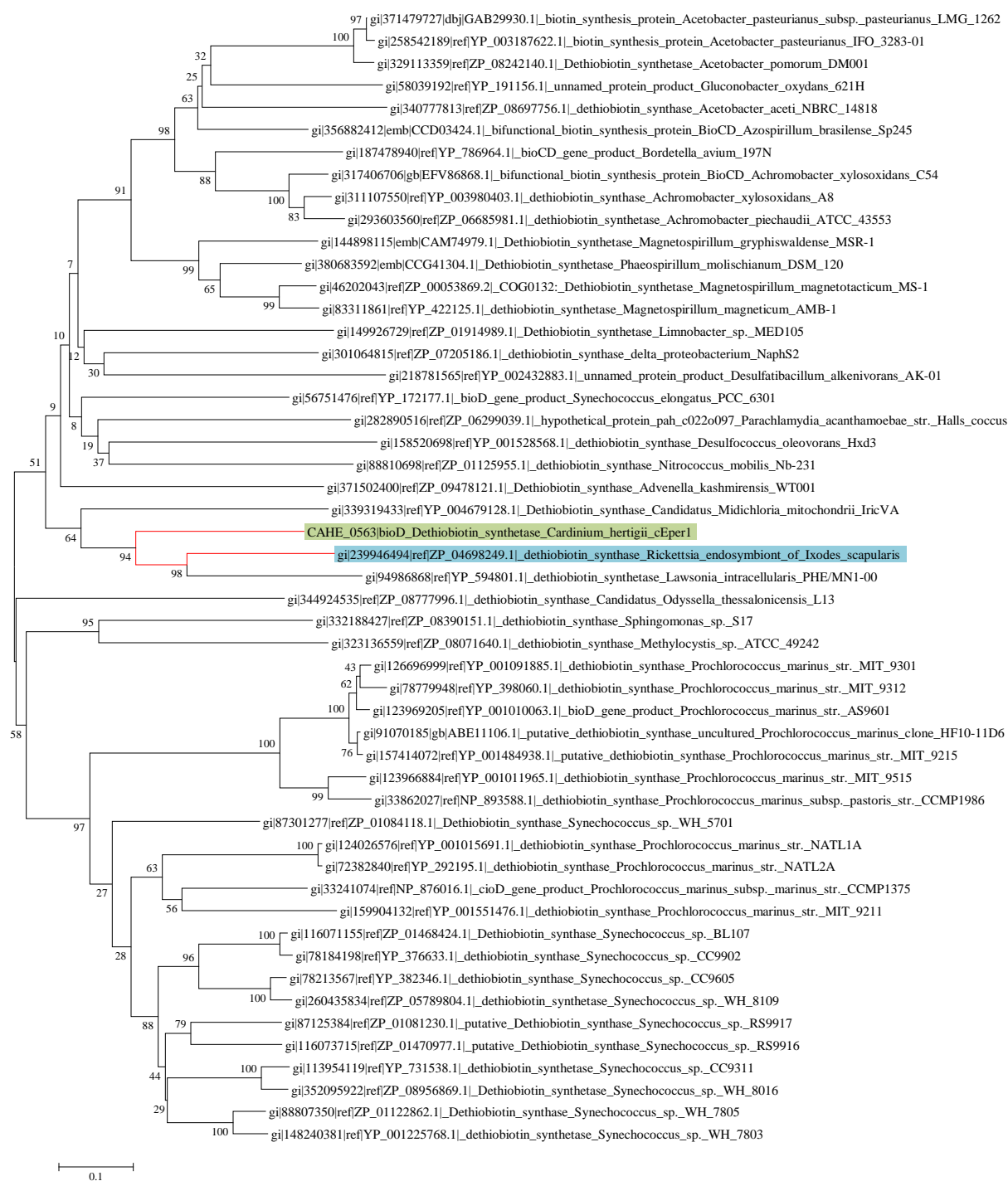

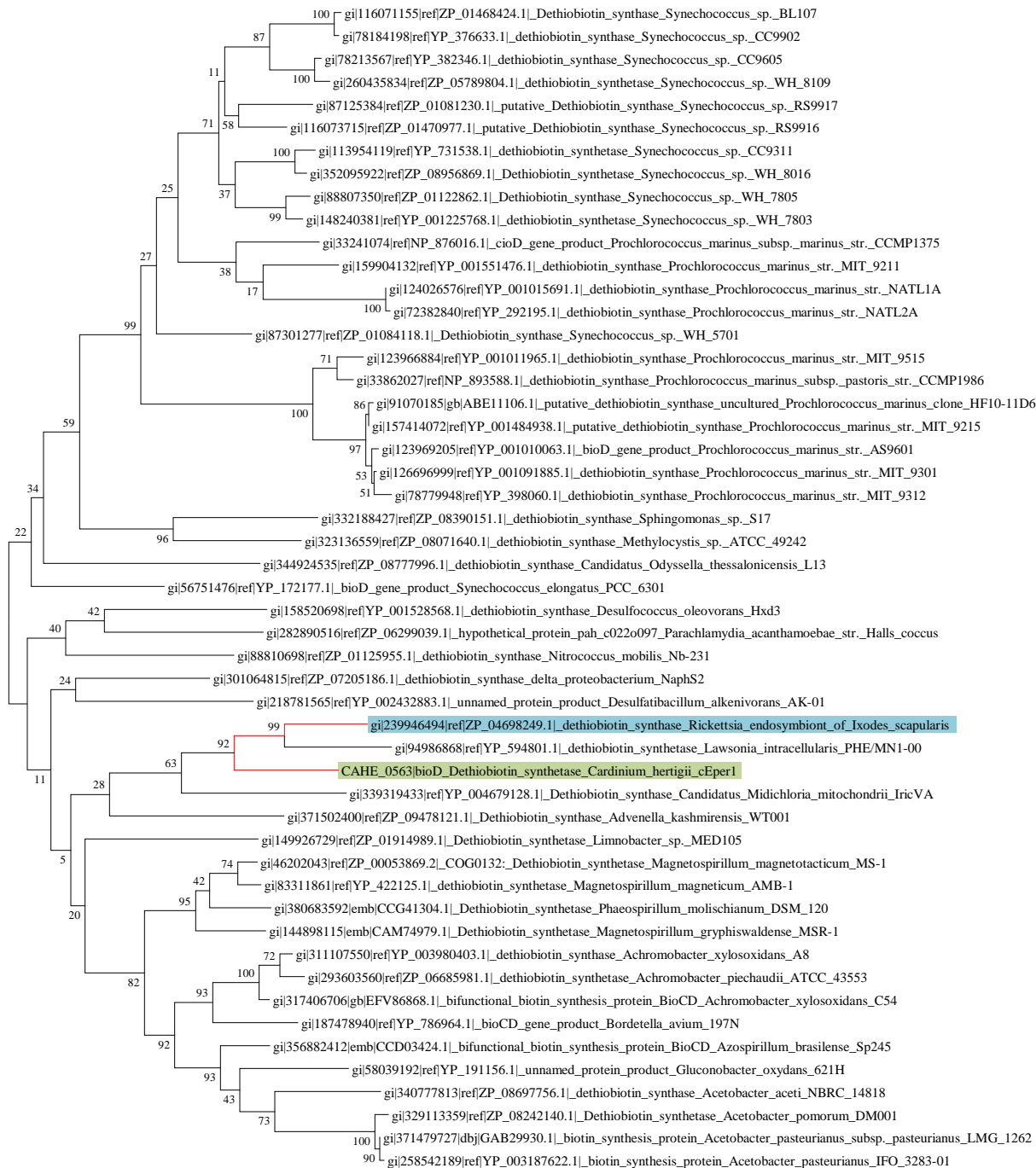

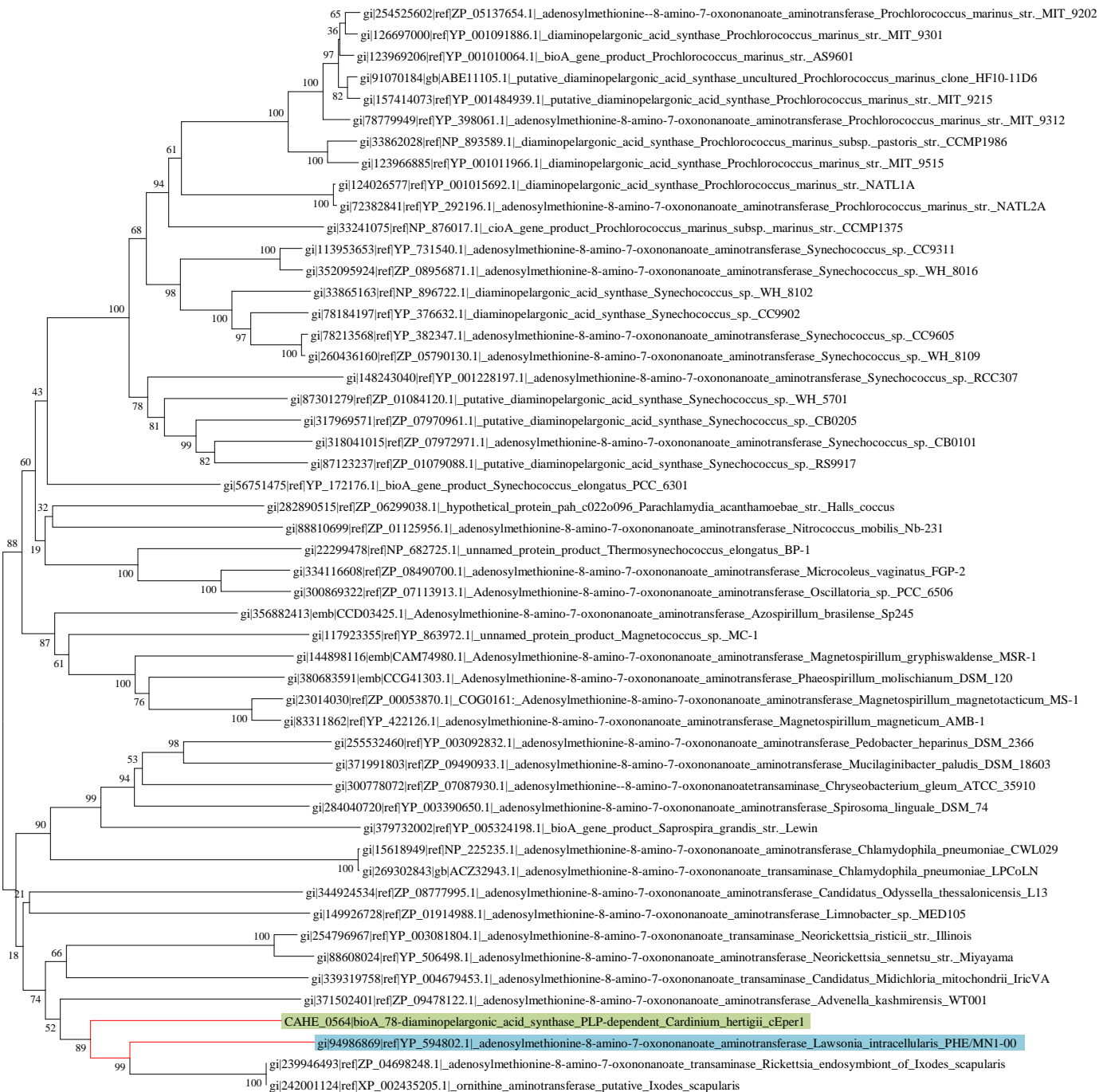

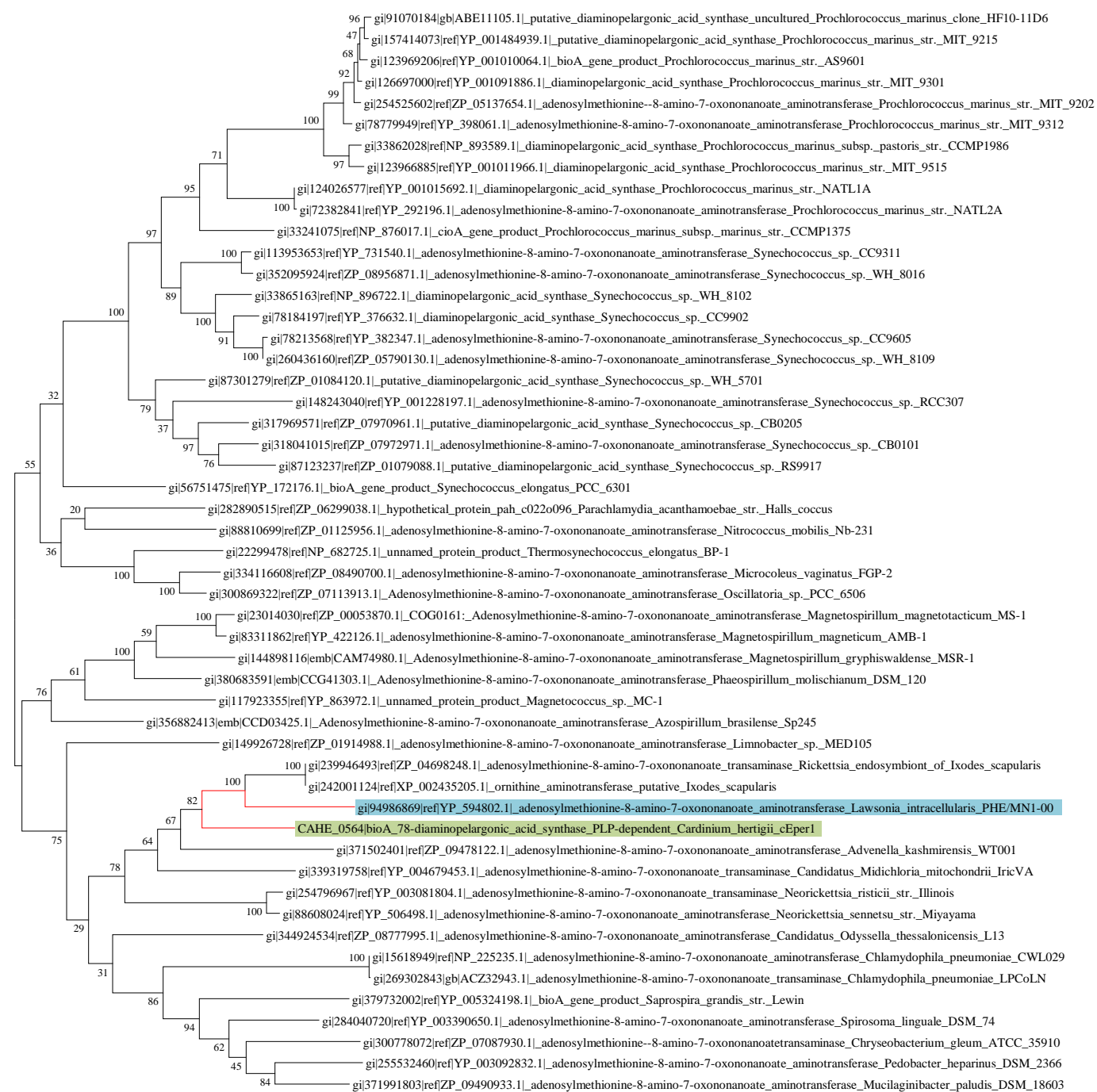

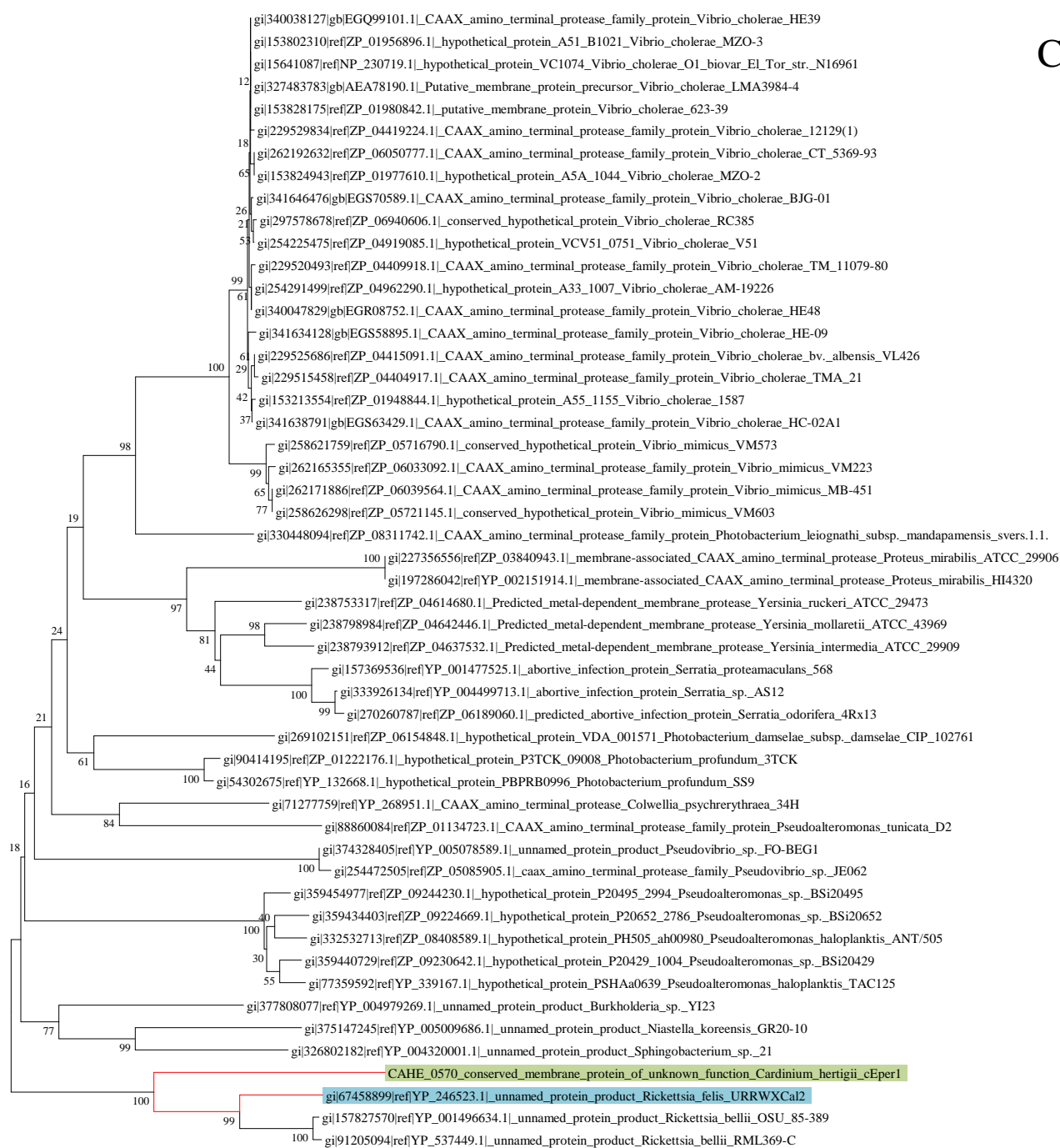

# CAHE\_0570 ML

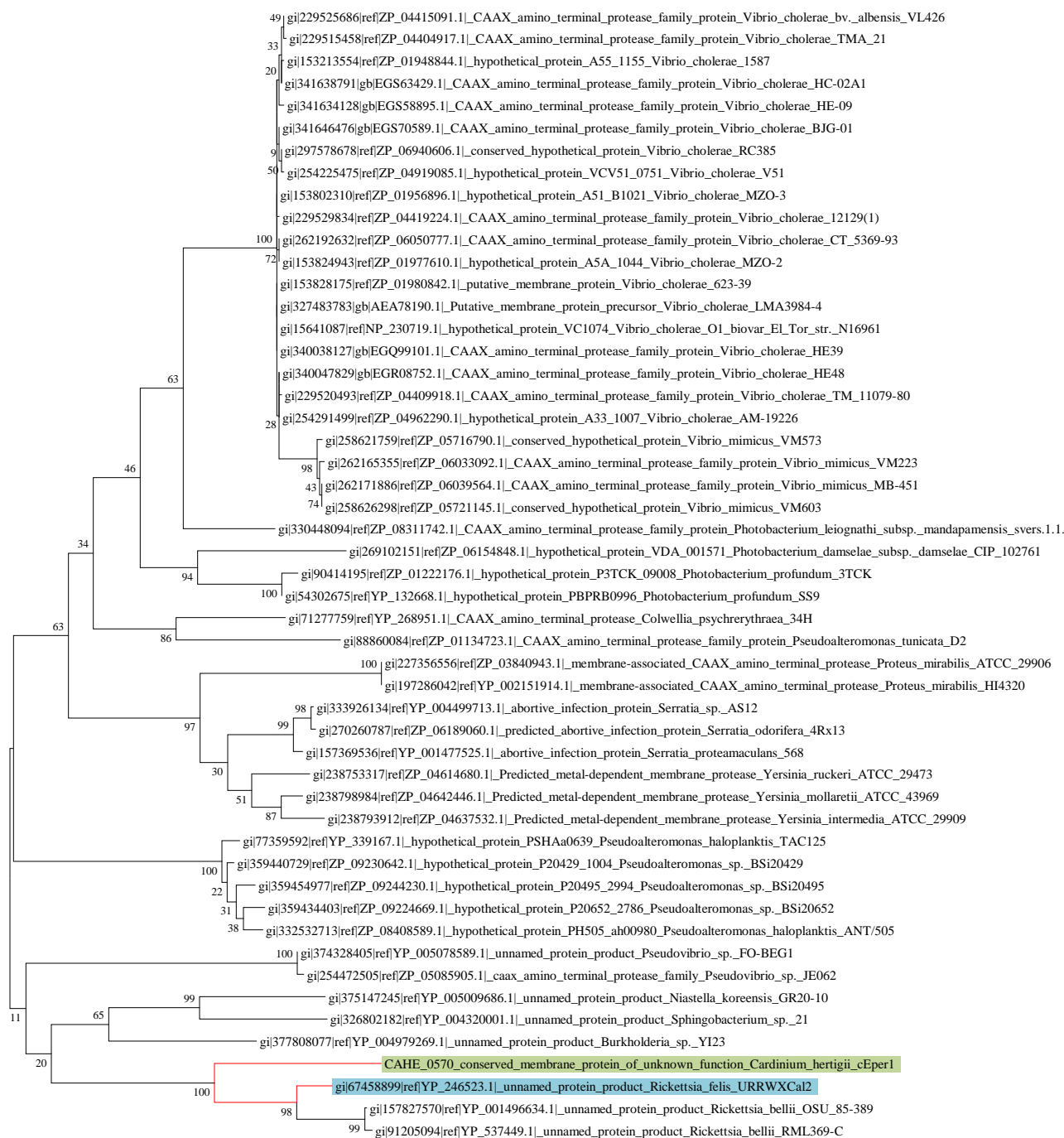

0.2

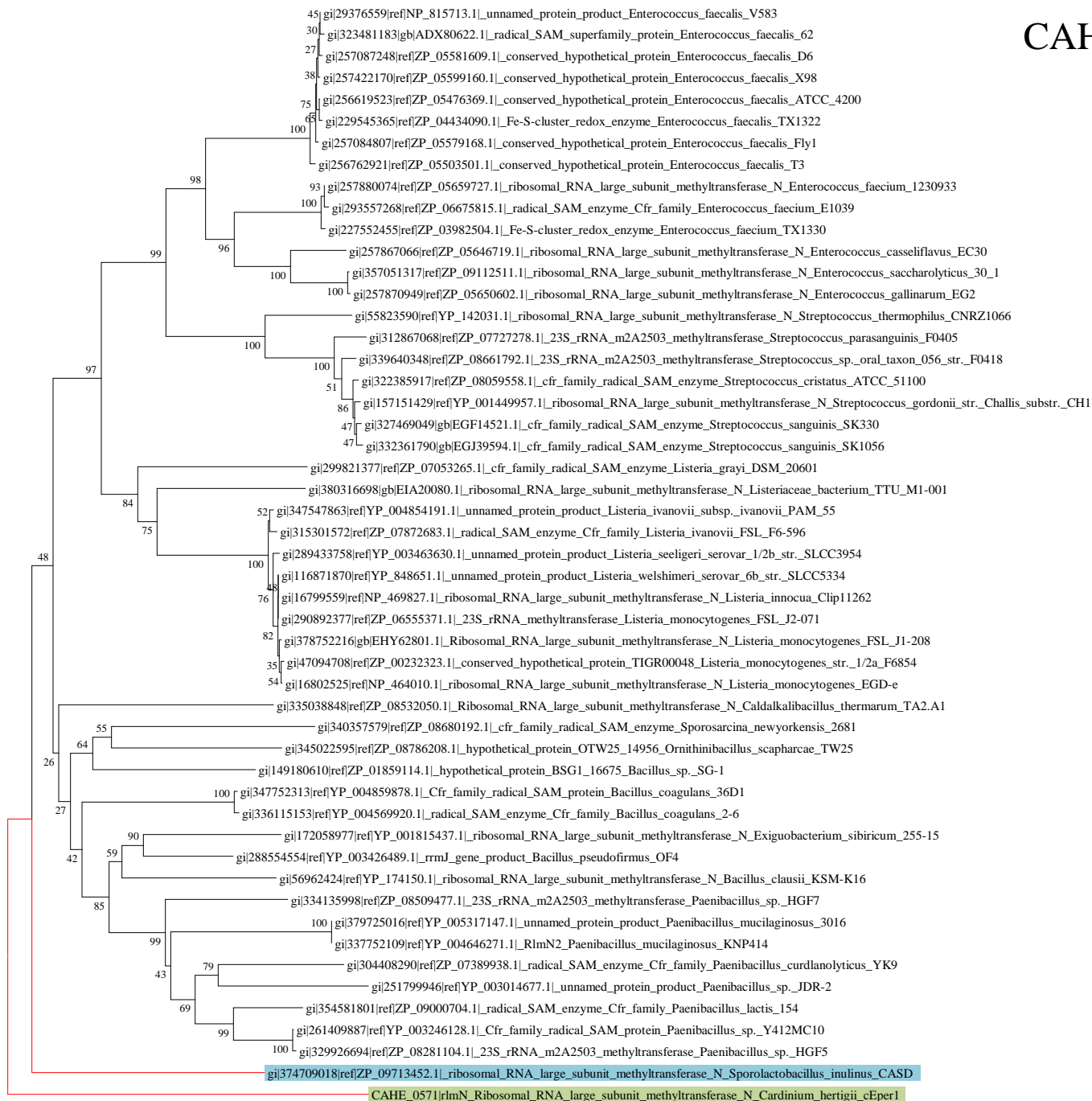

0.05

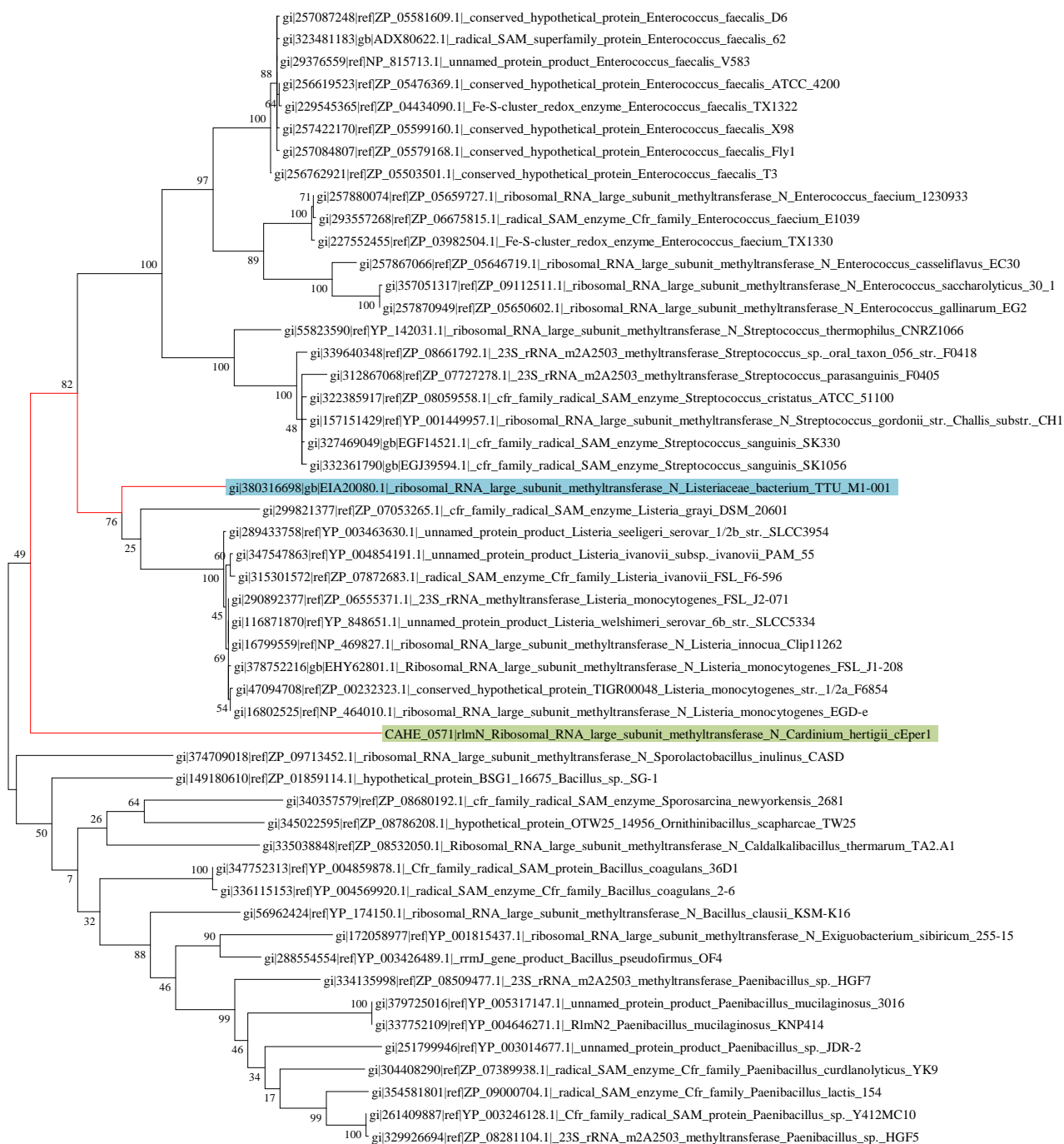

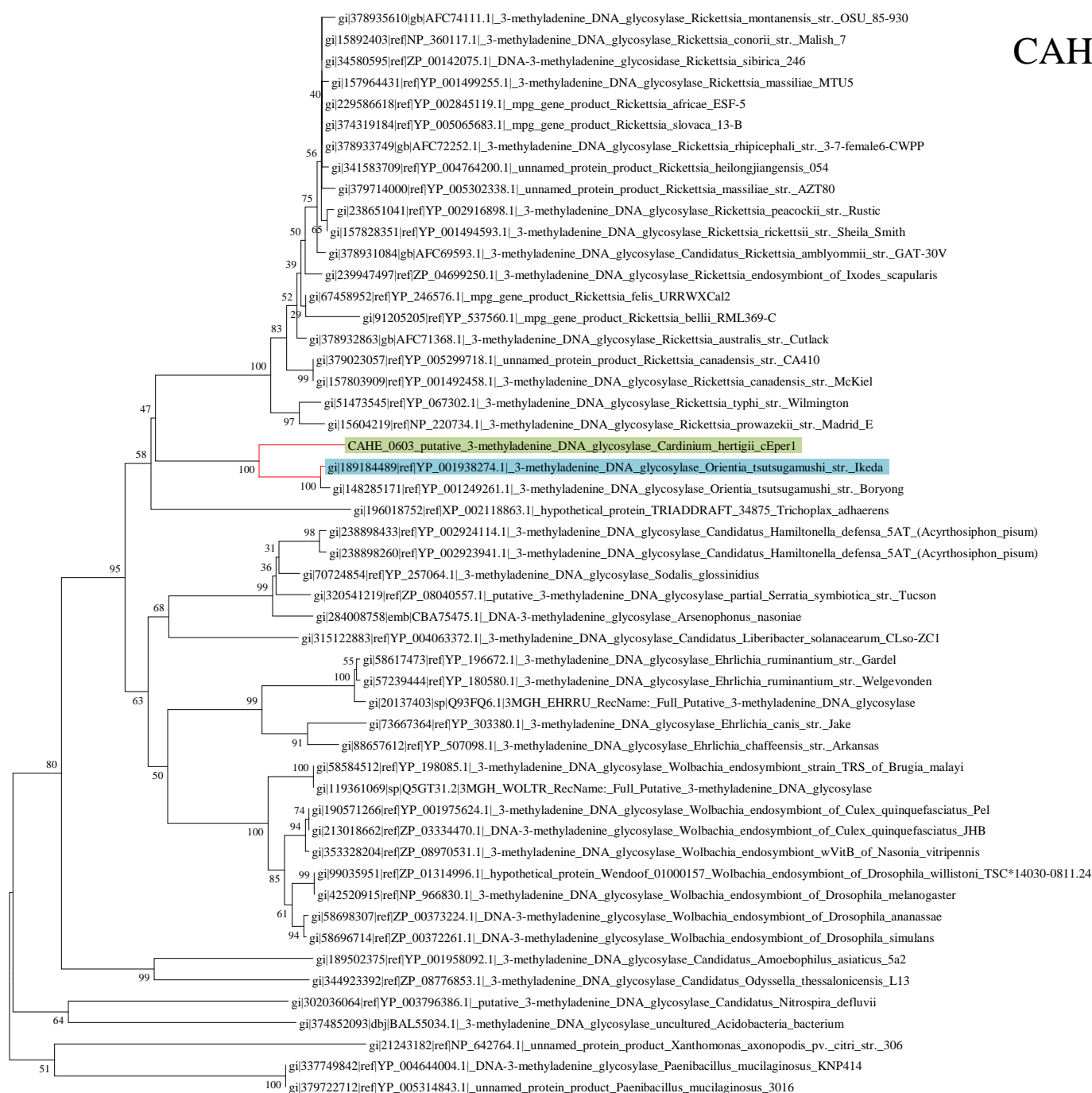

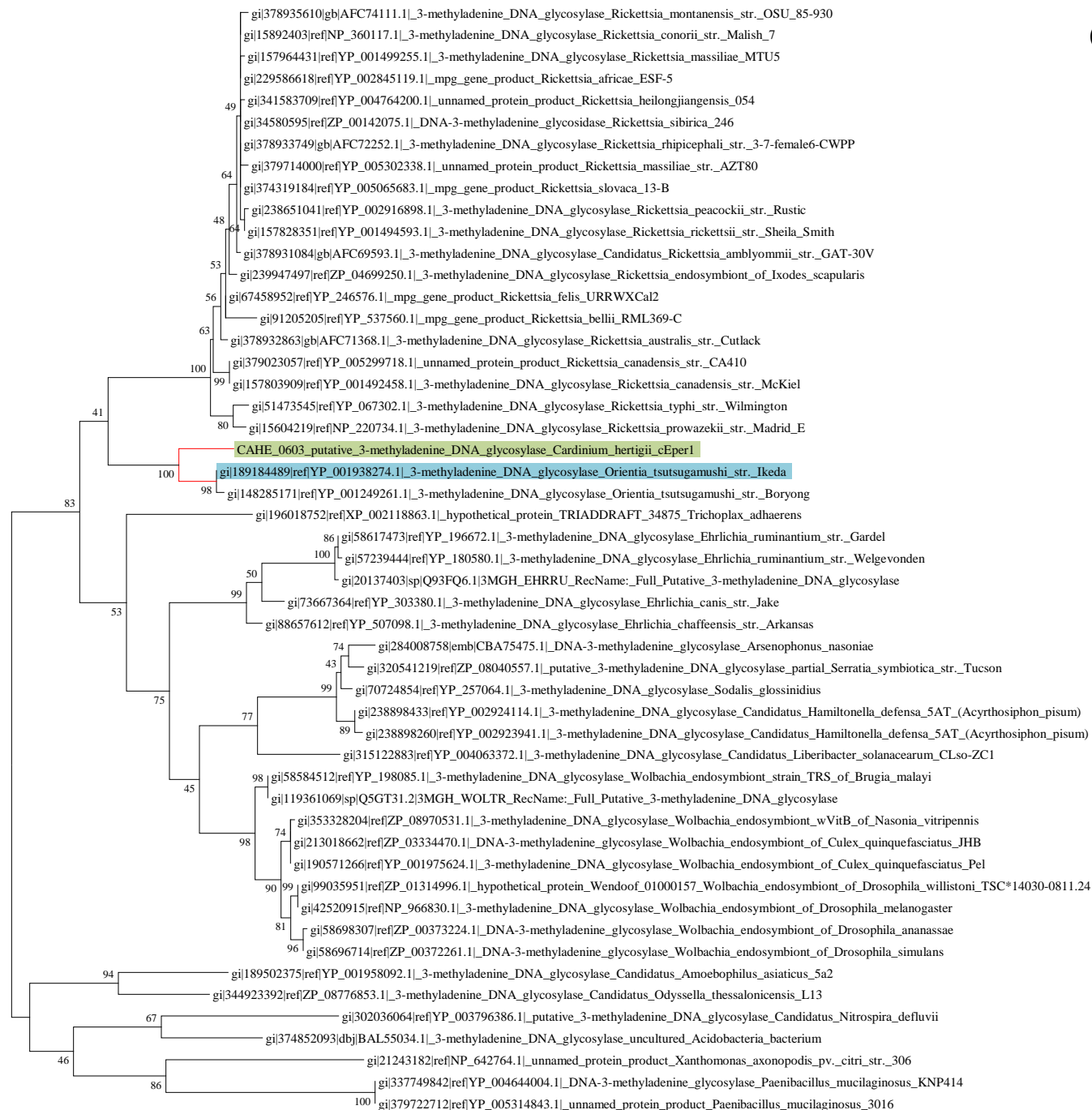

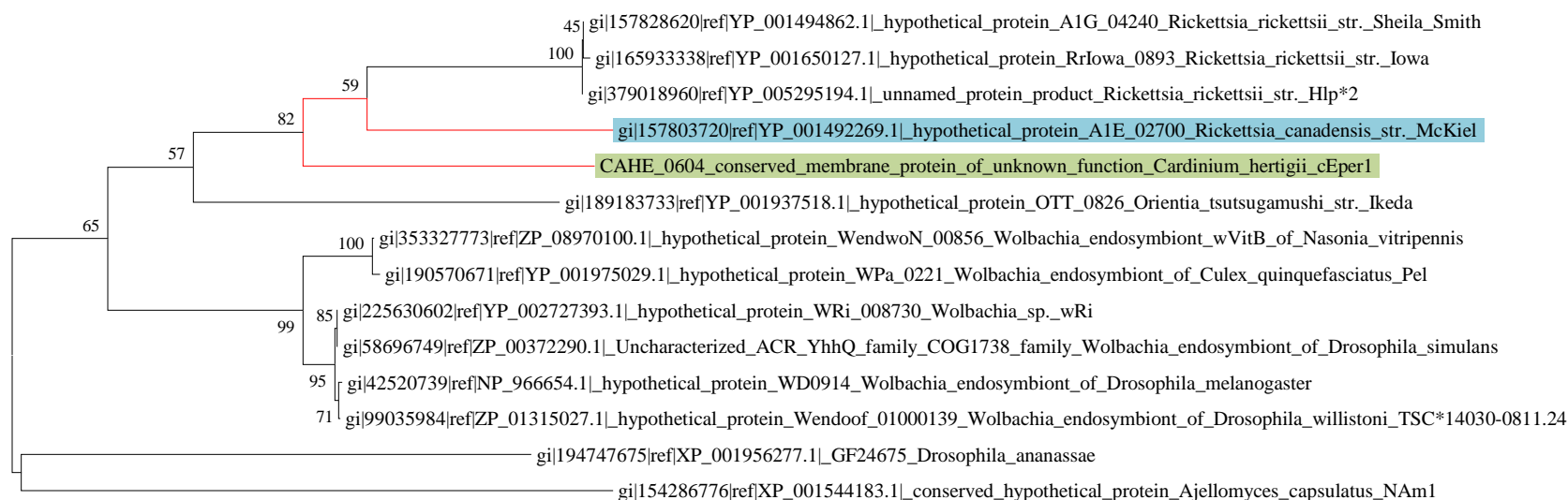

0.2

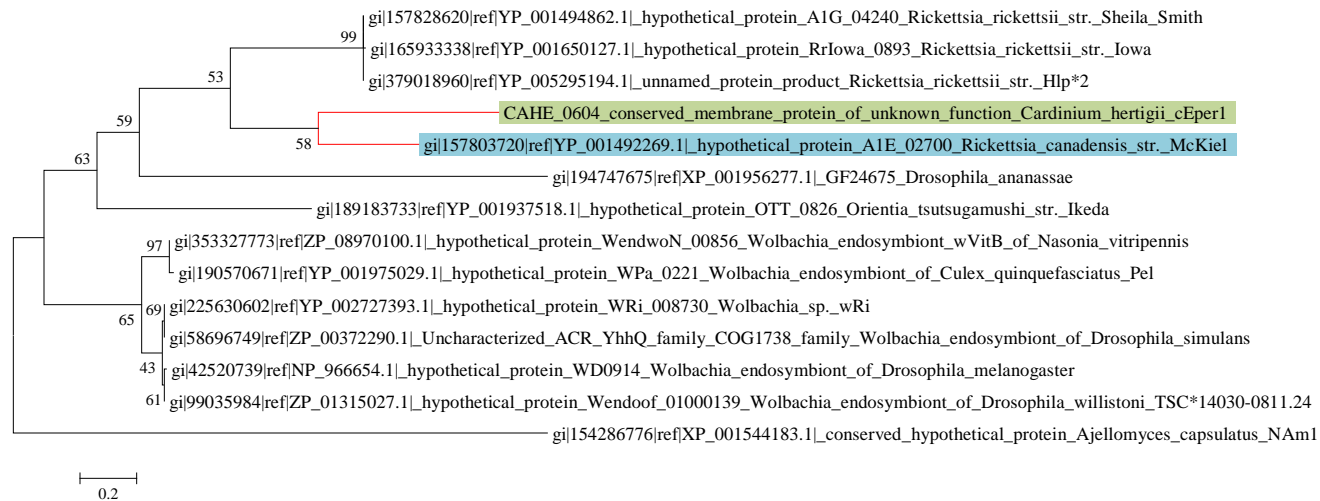

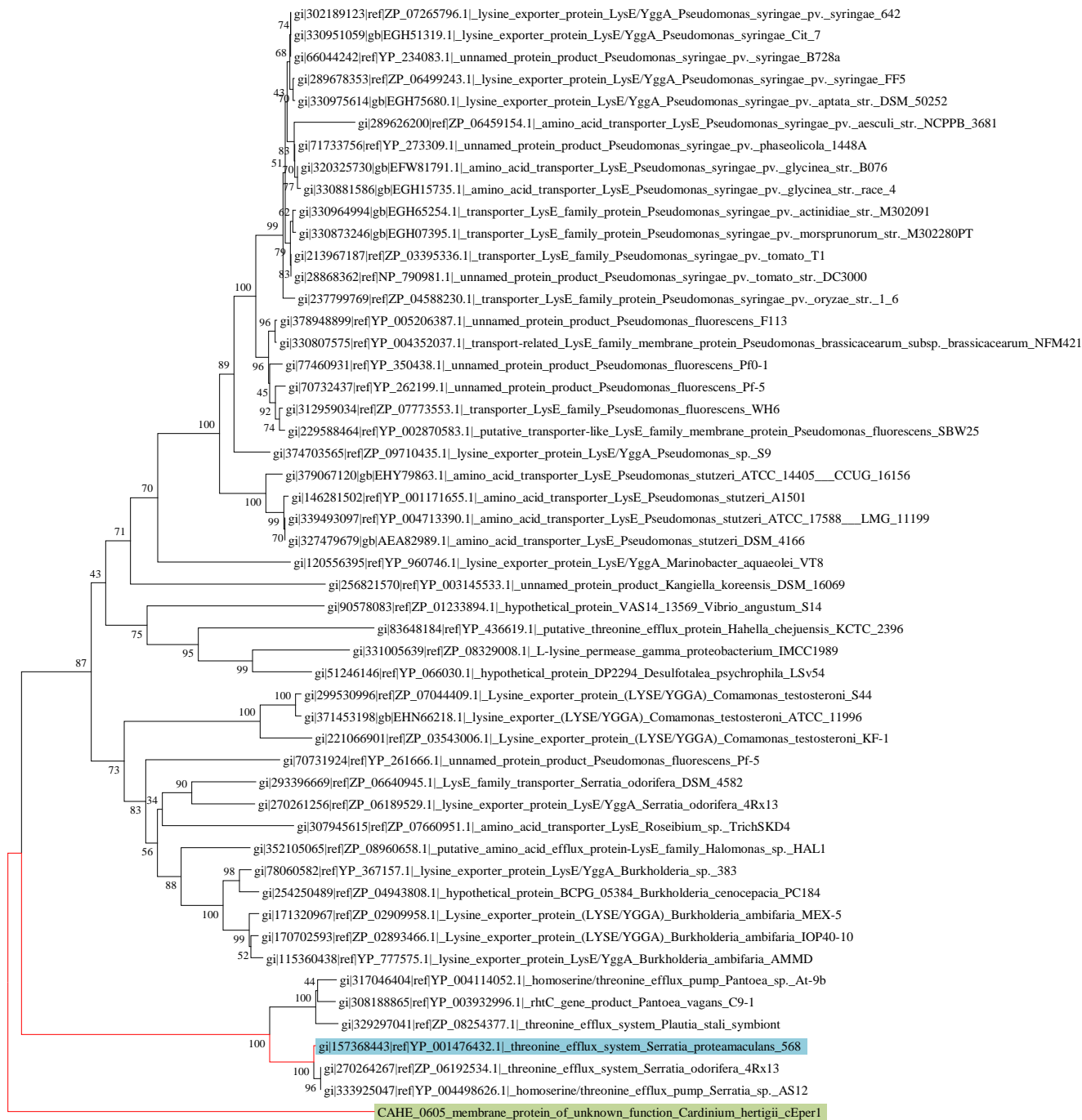

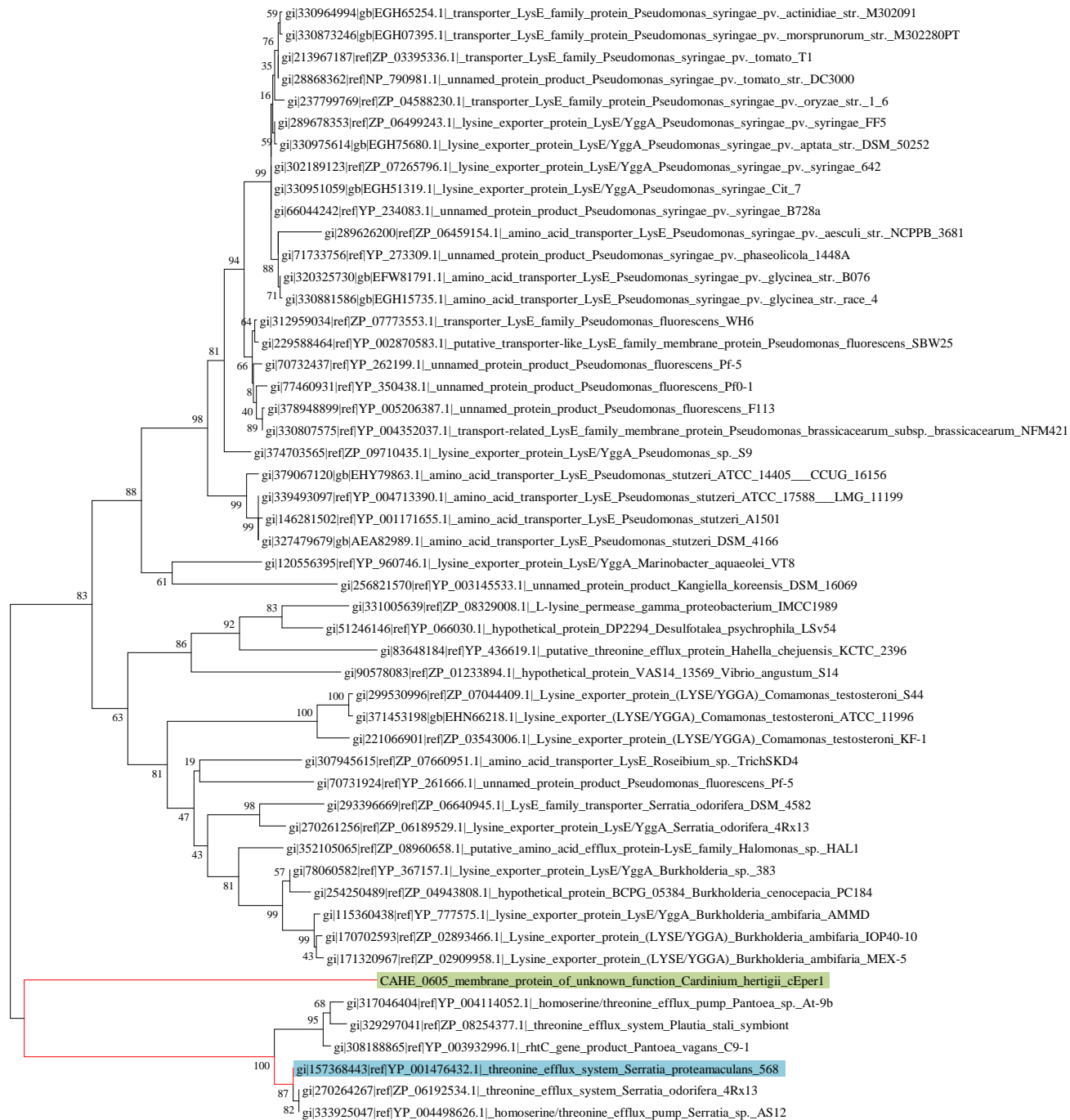

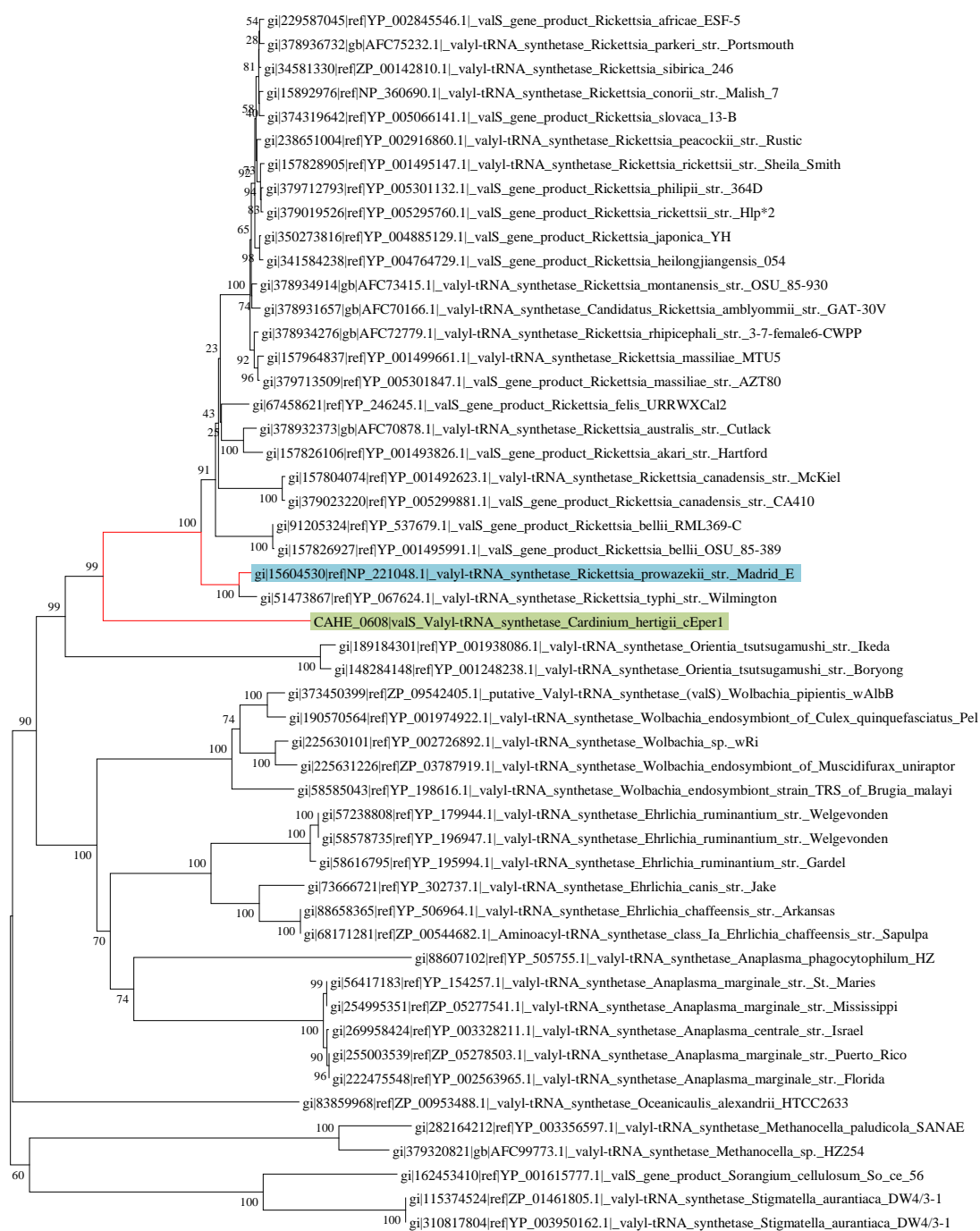

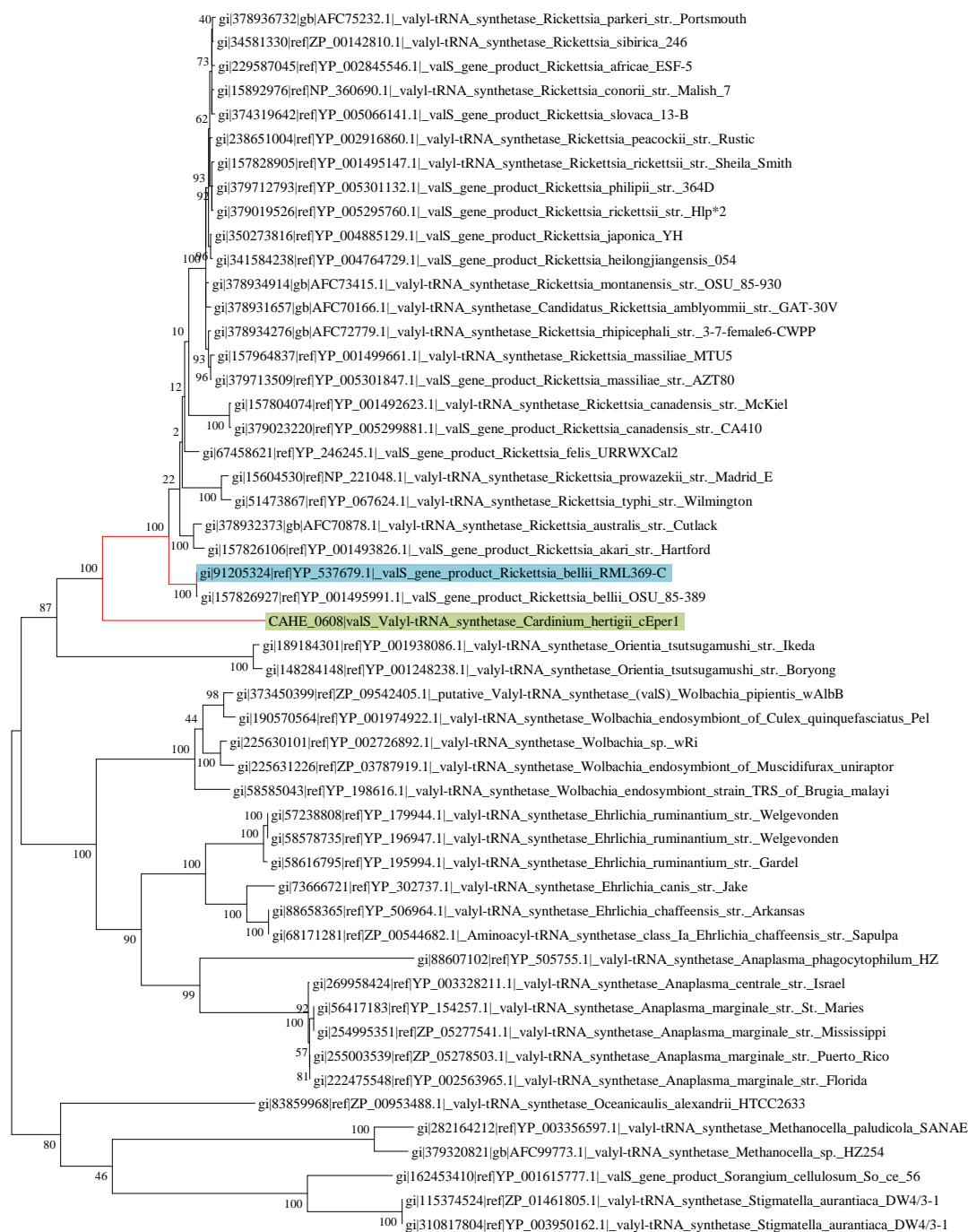

0.1

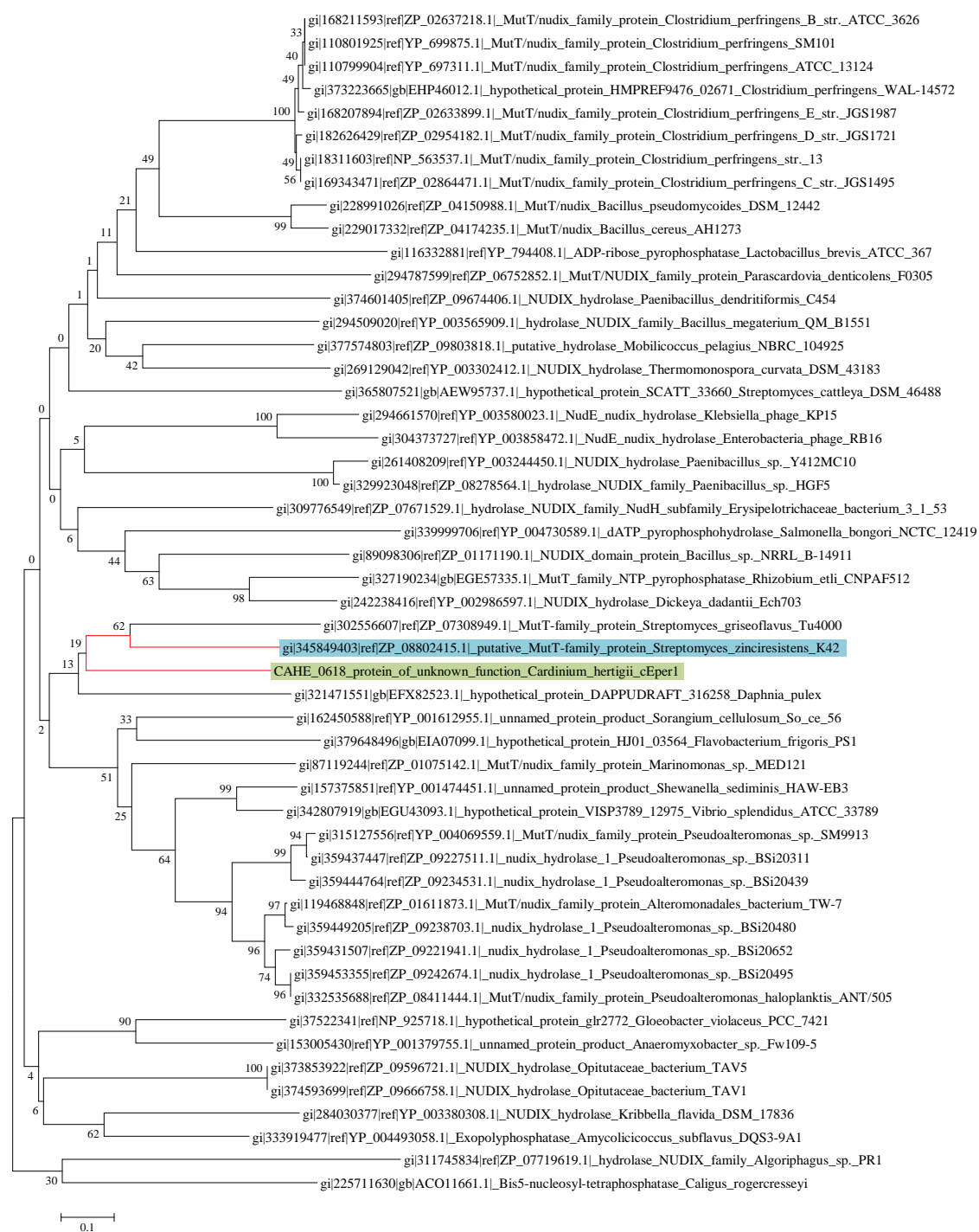

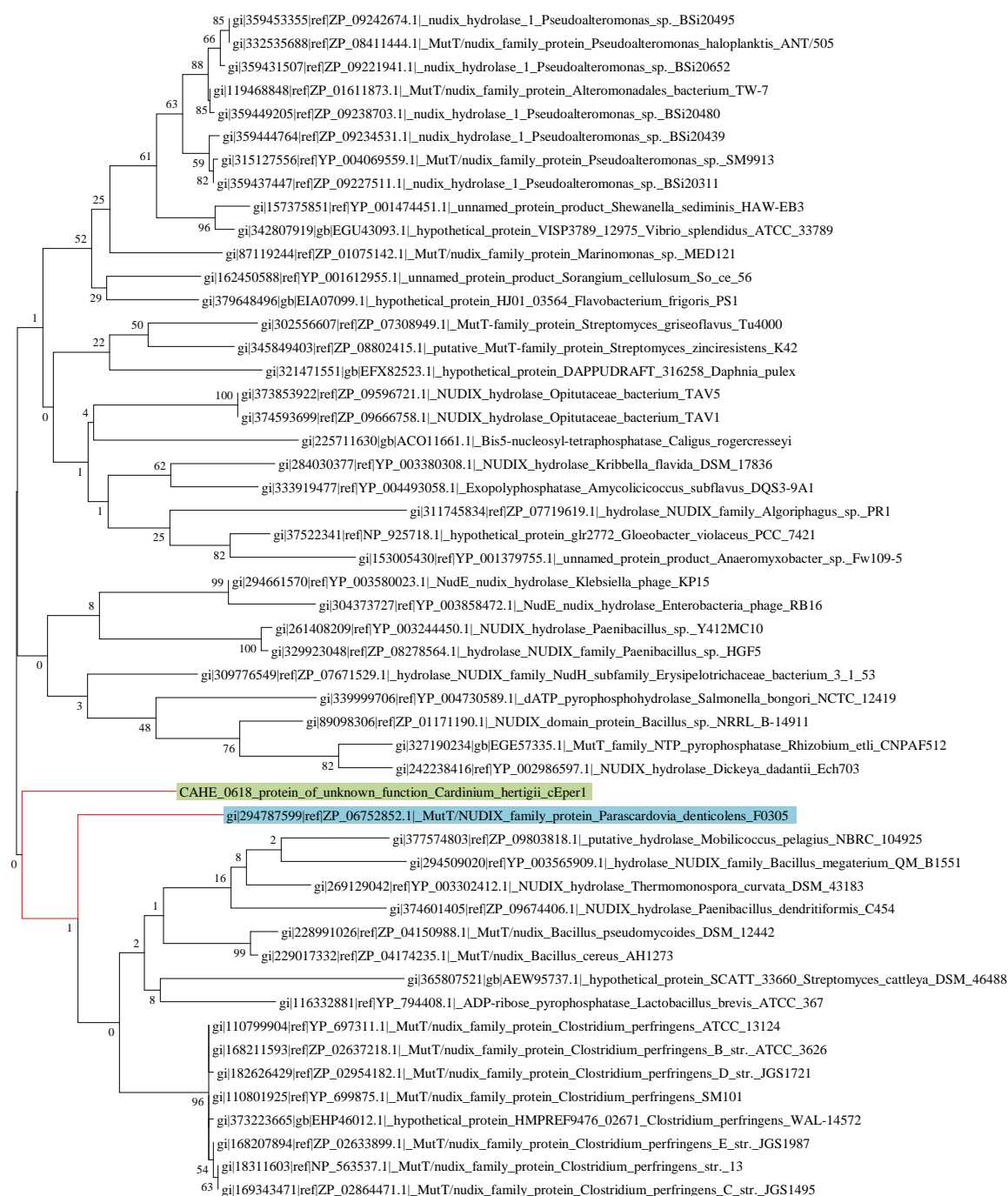

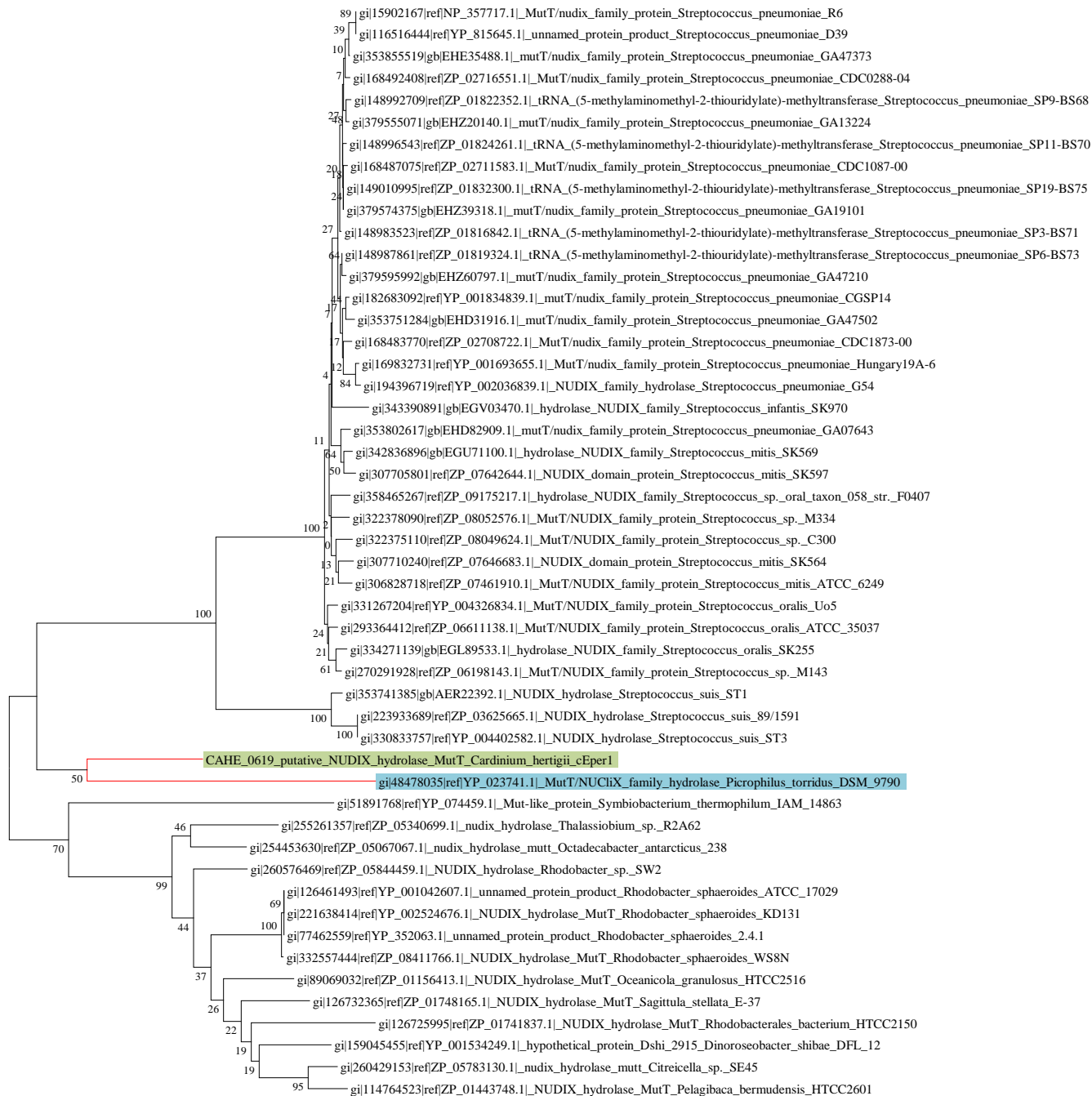

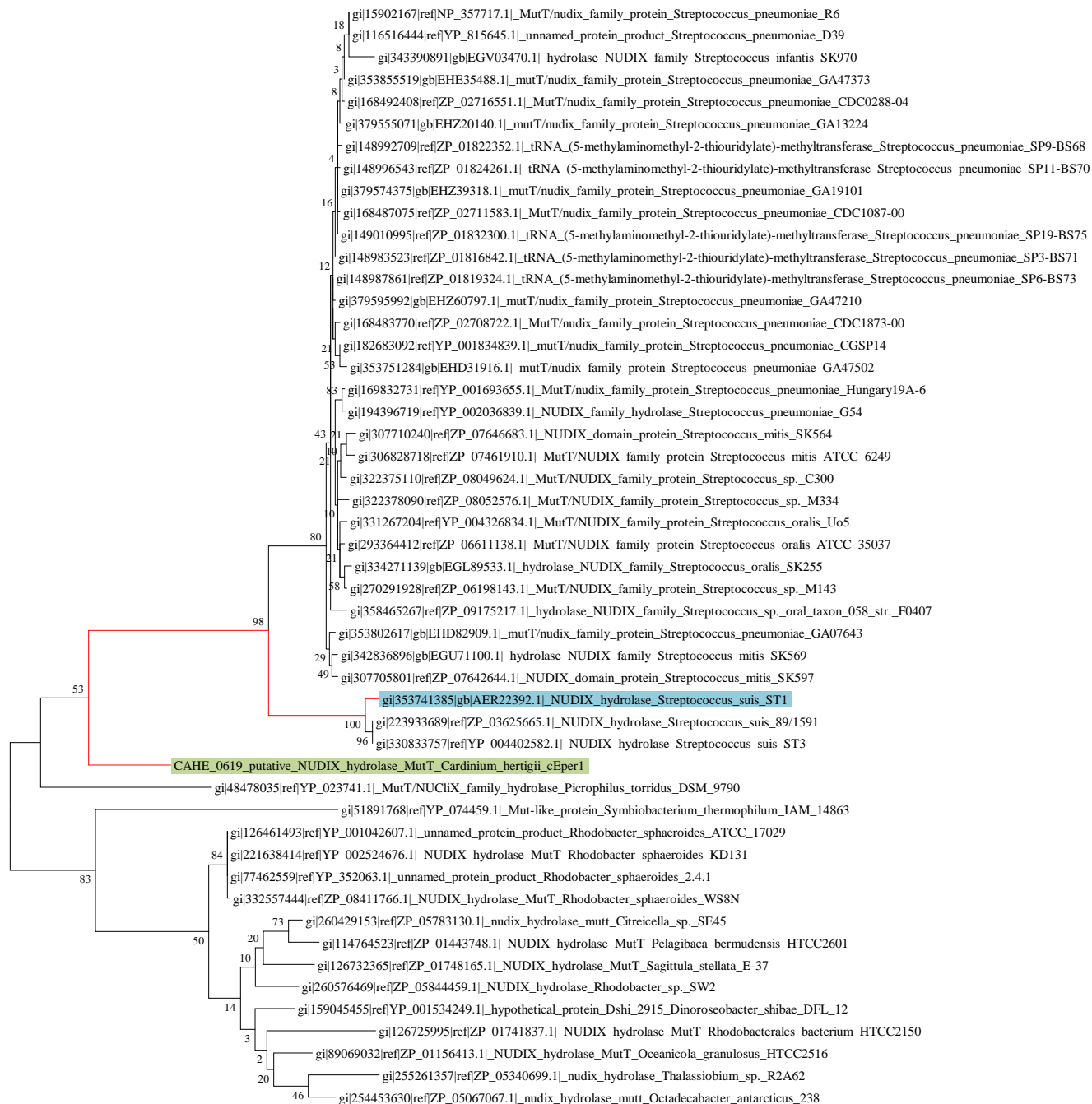

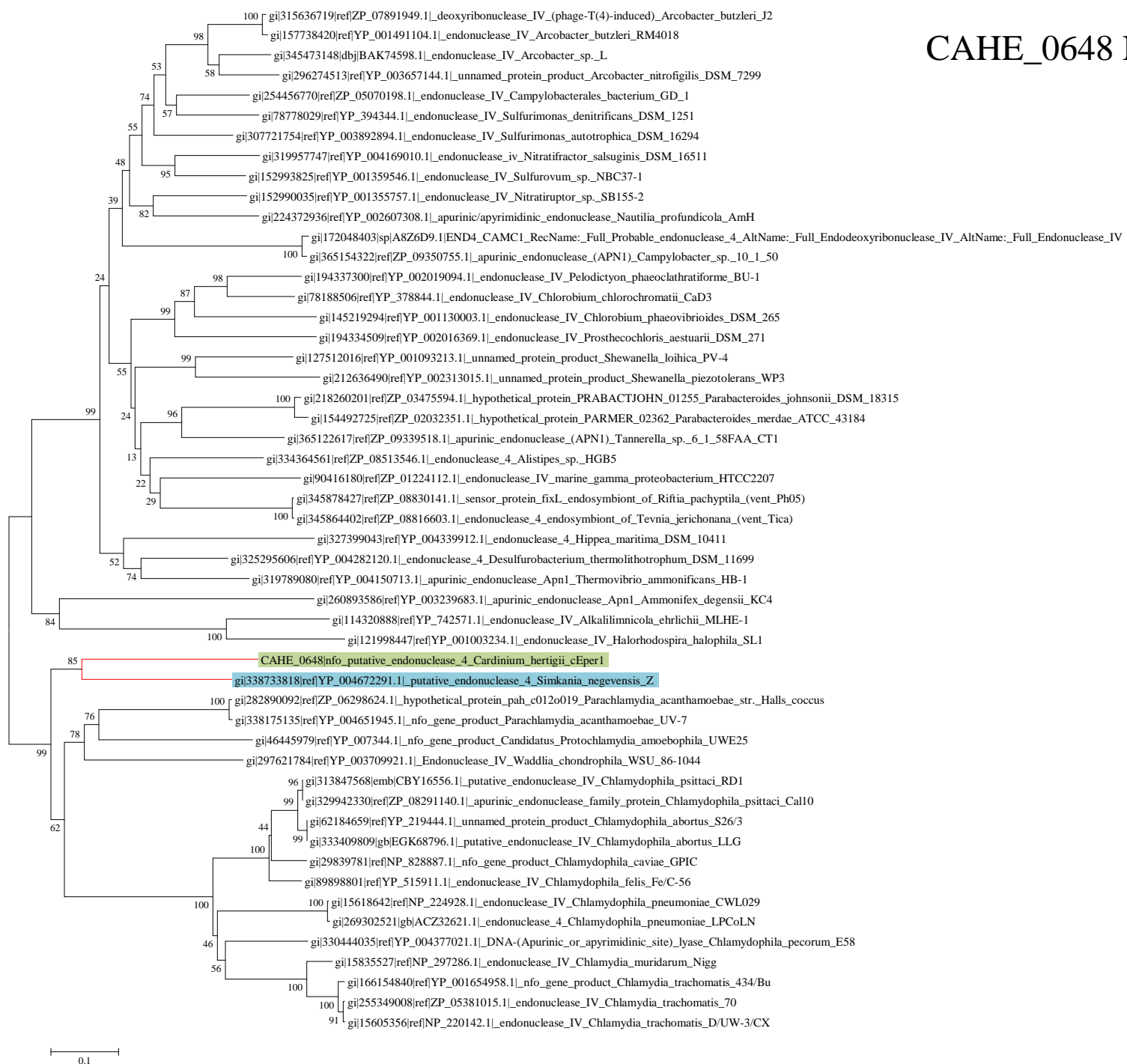

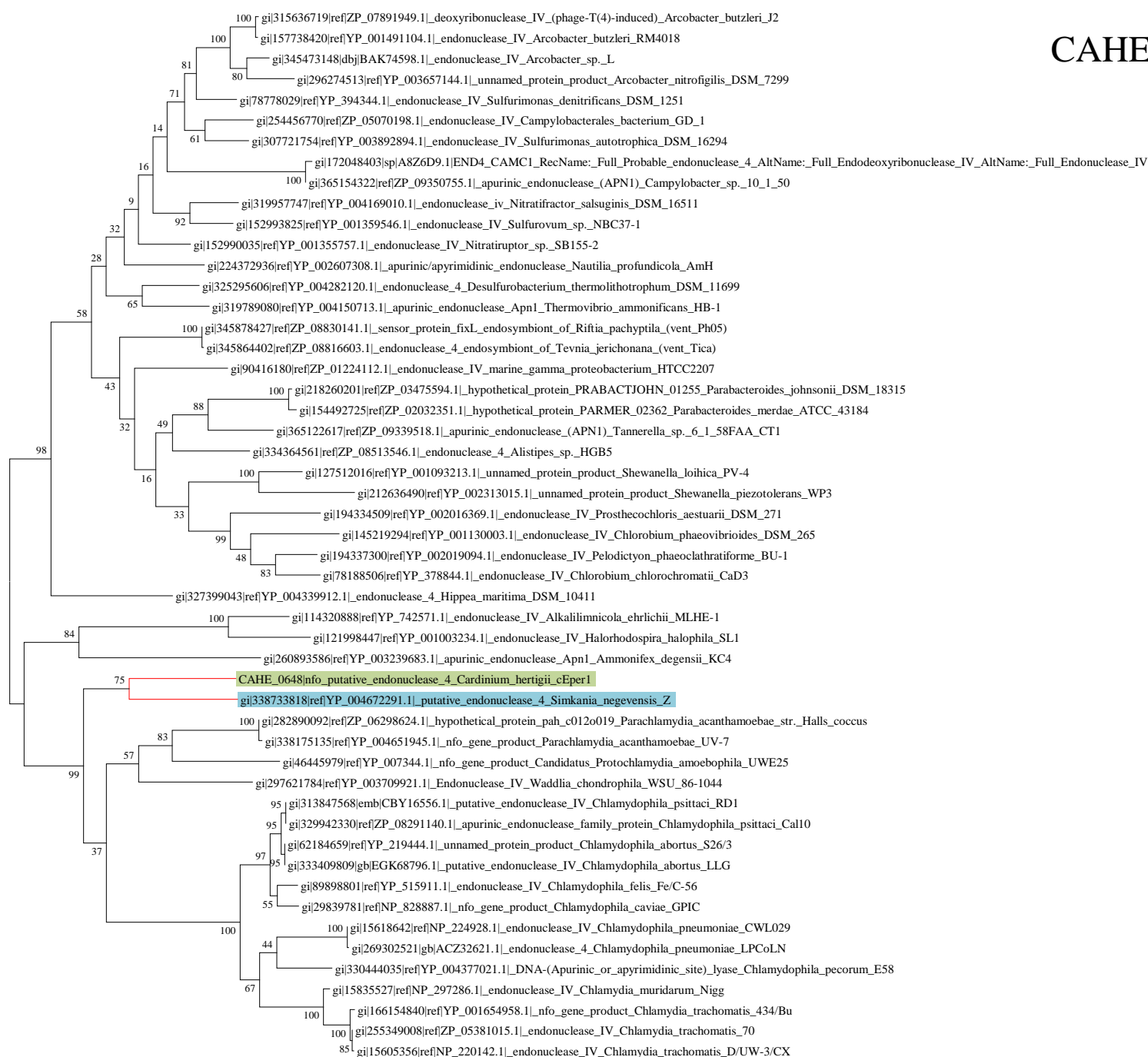

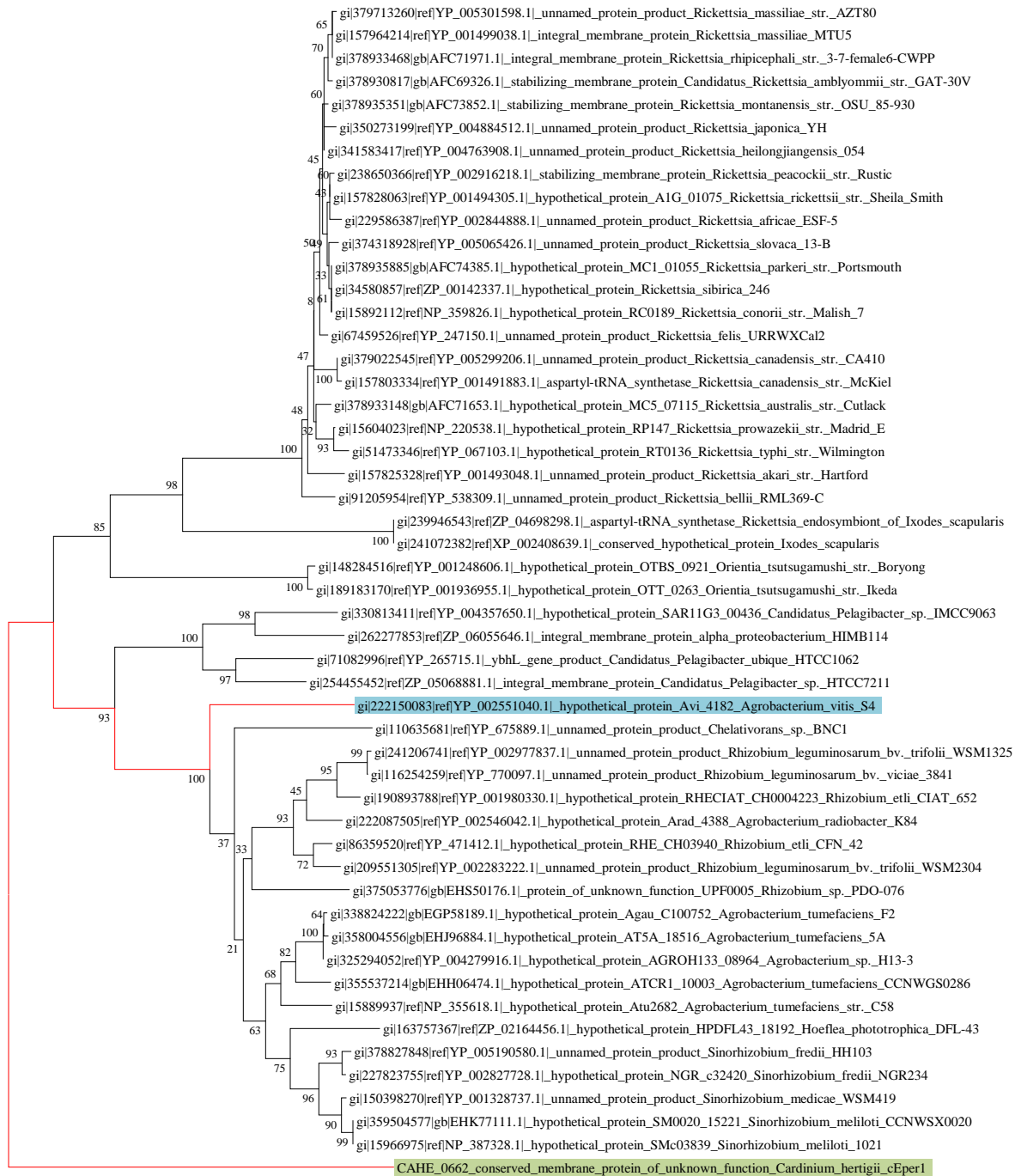

0.1

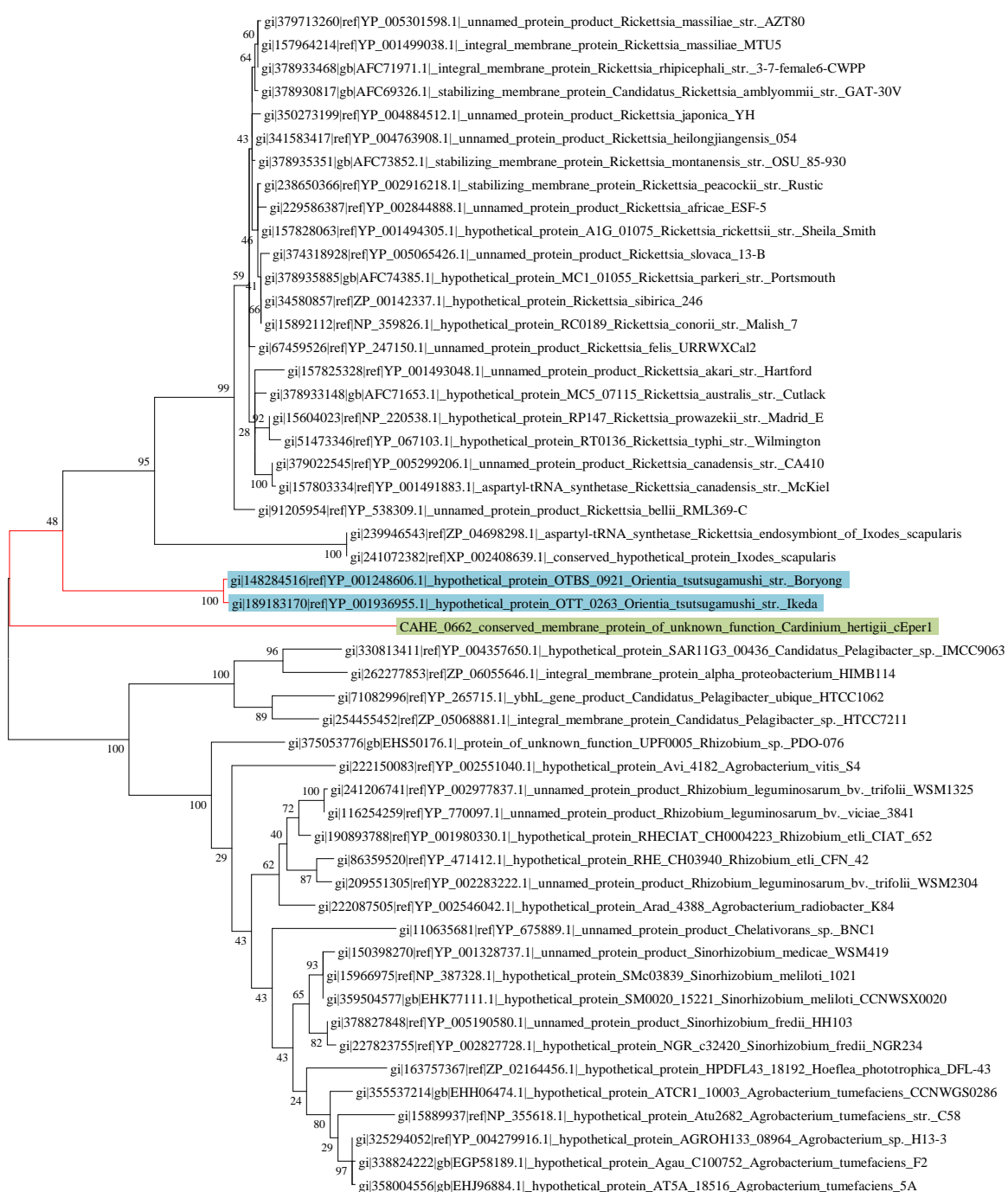

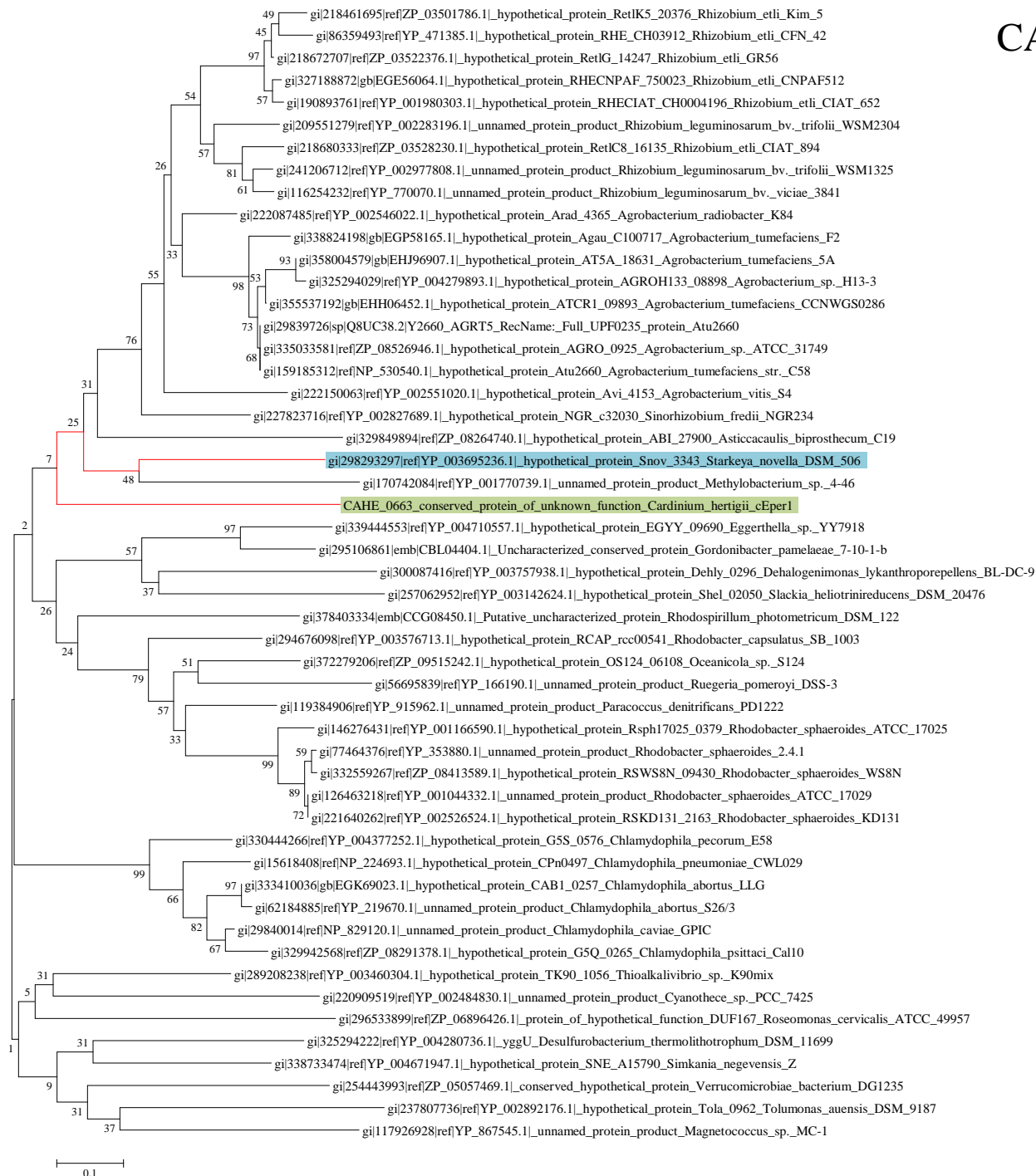

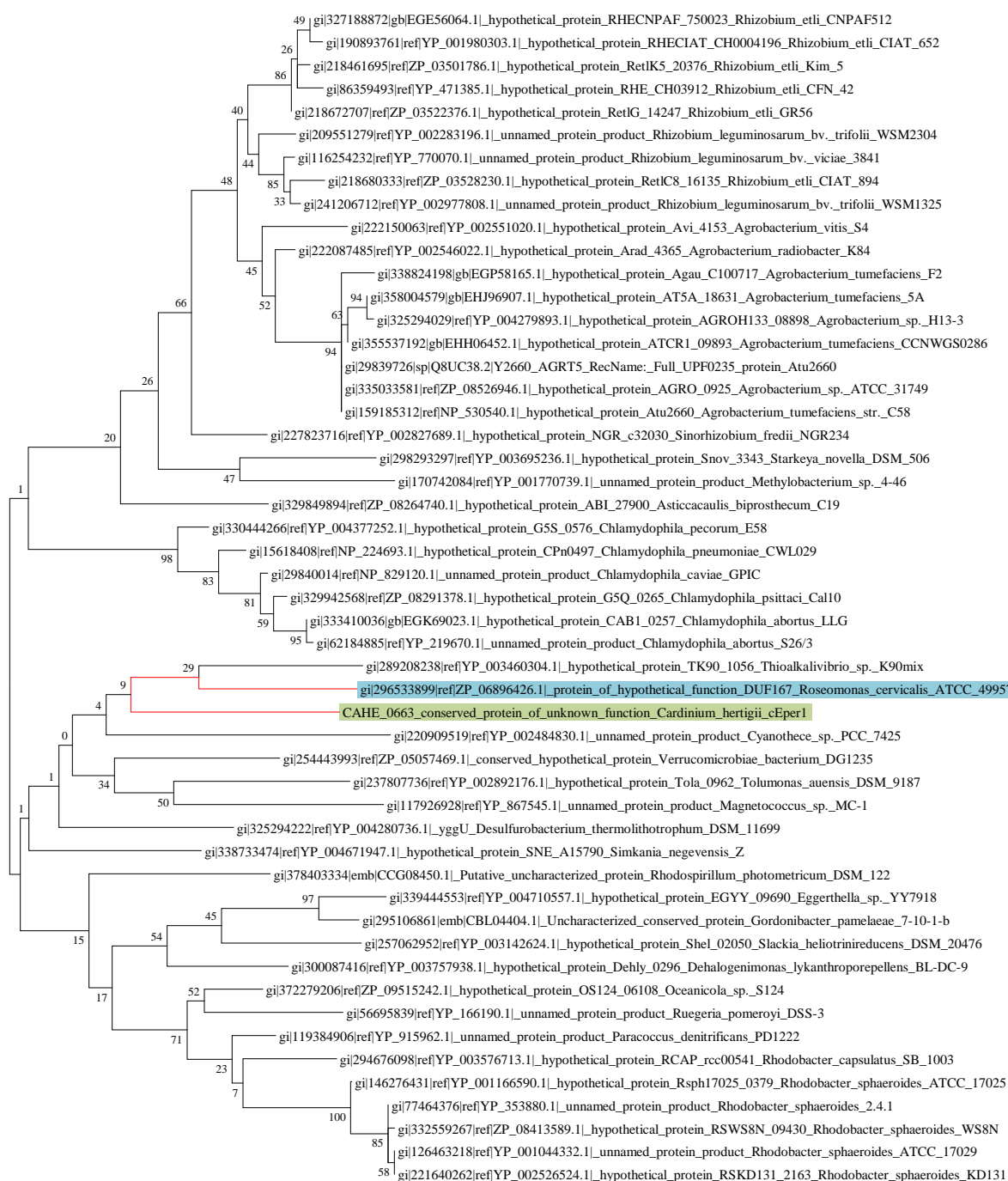

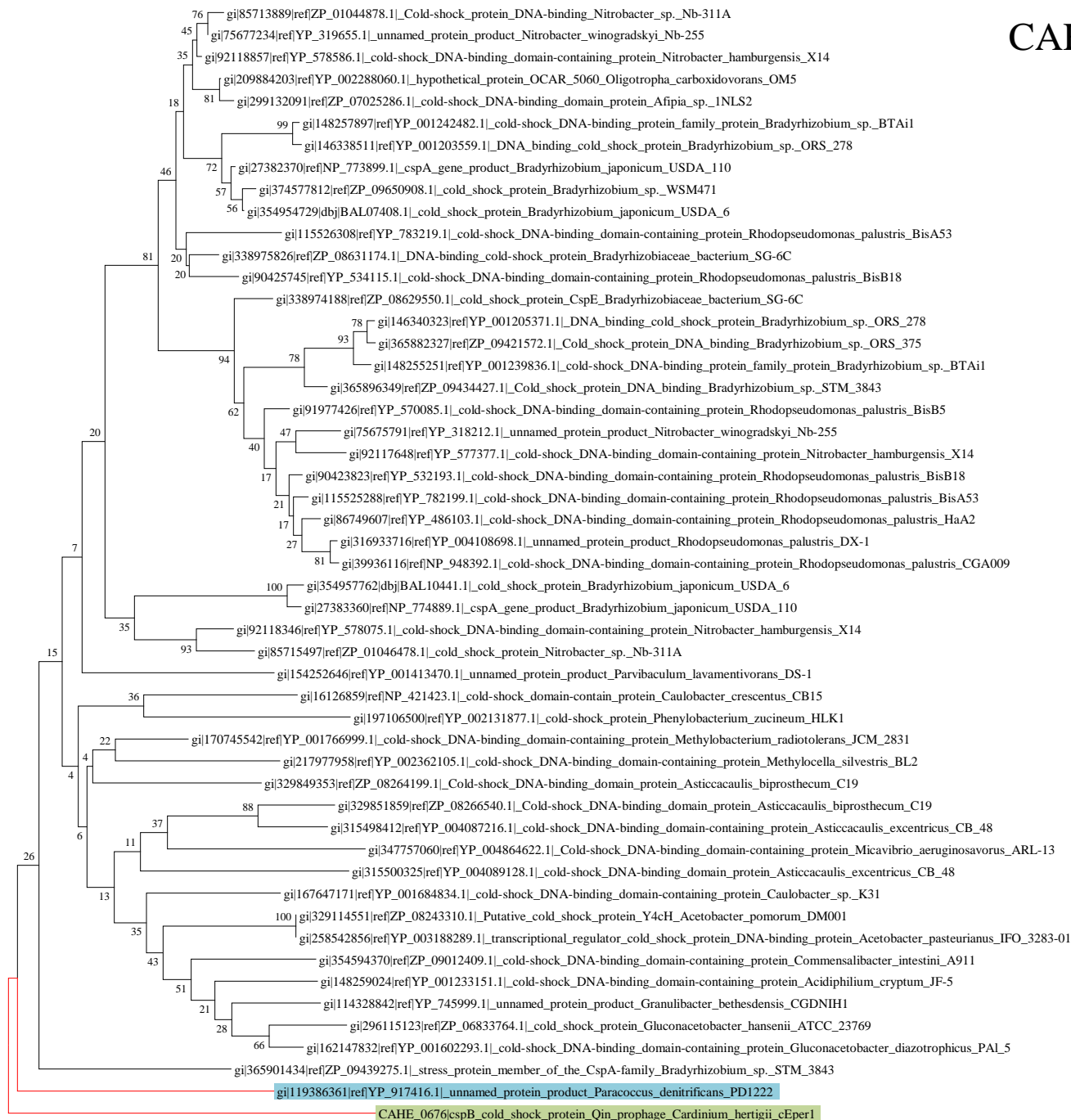

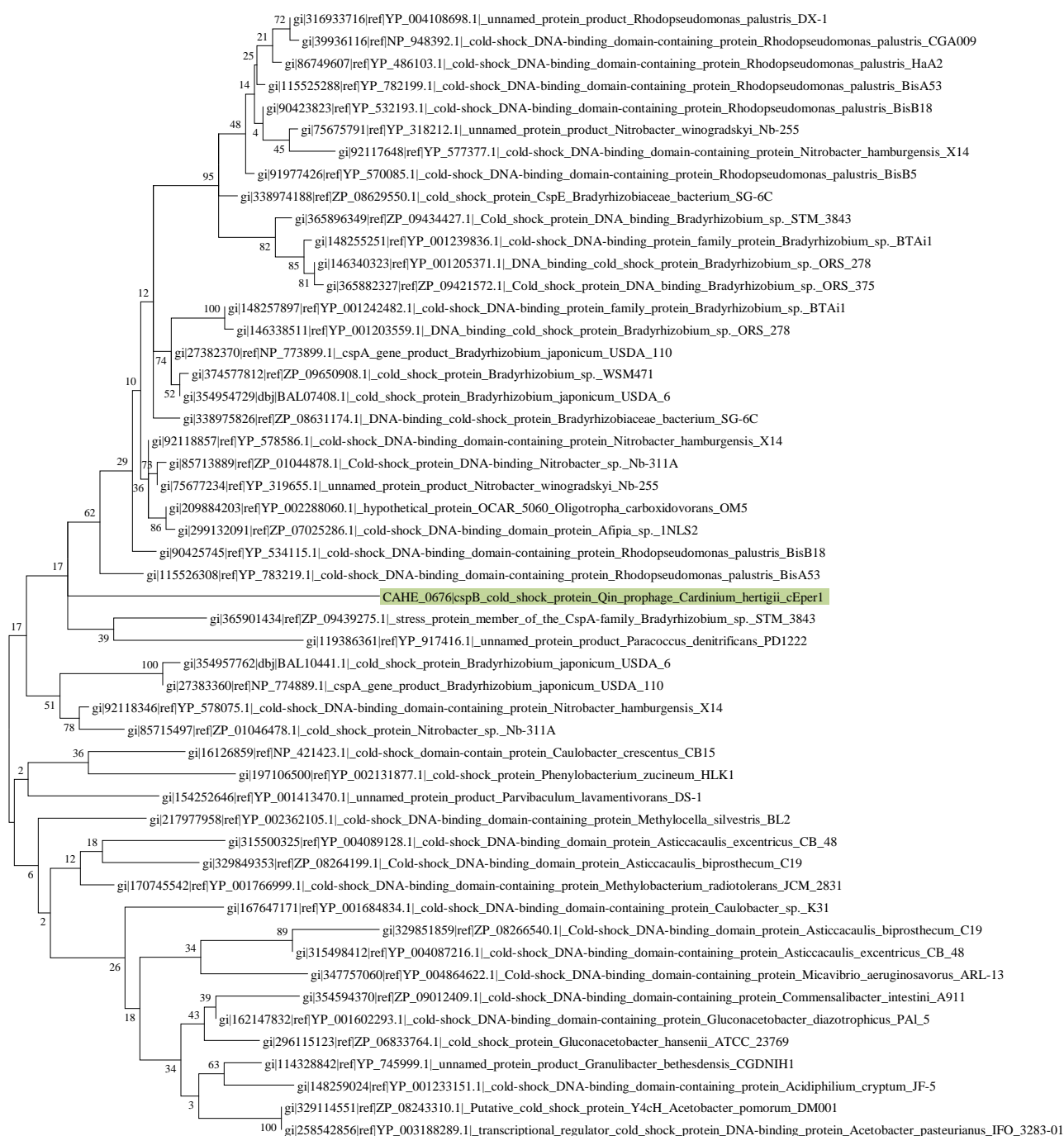

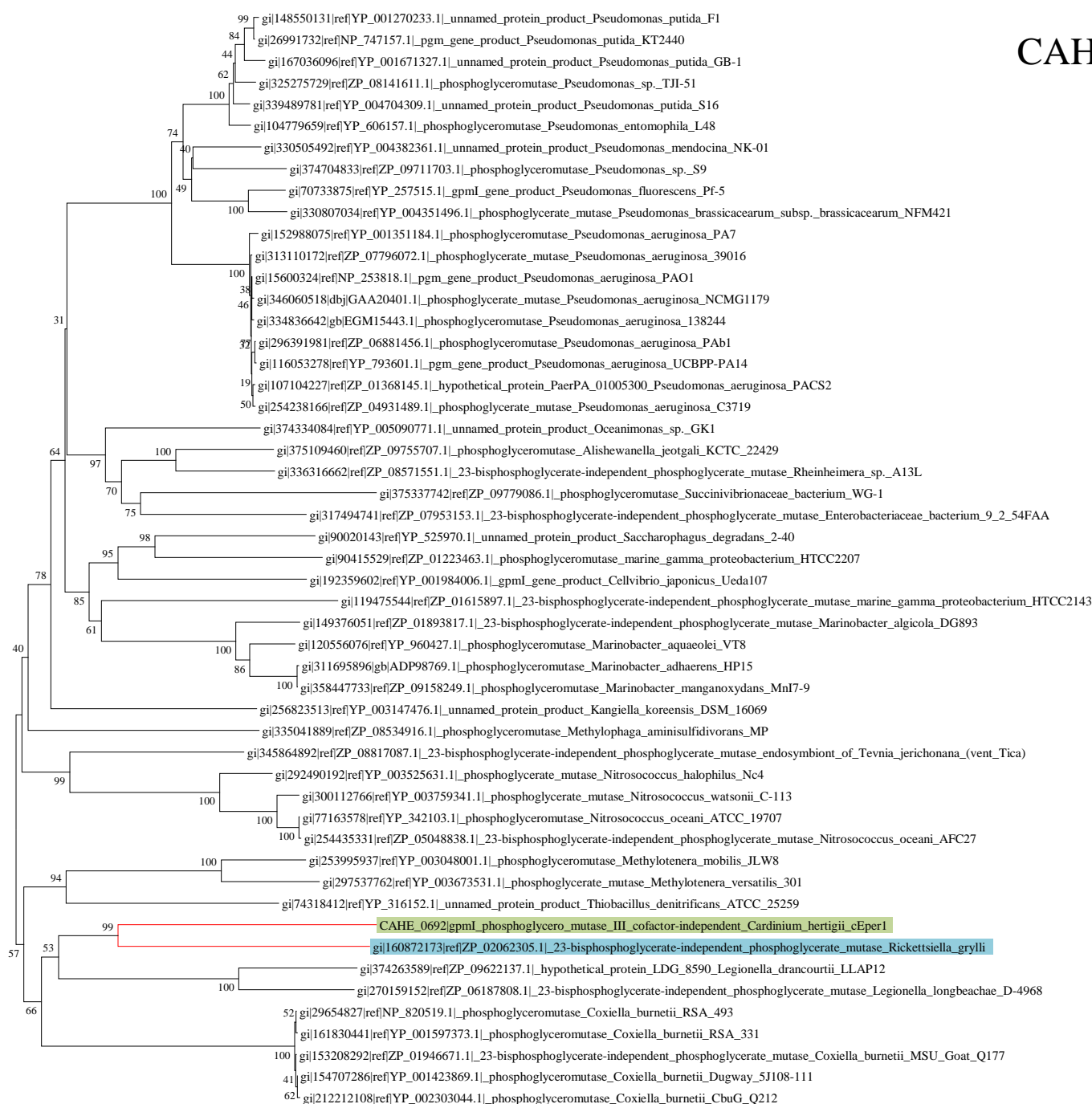

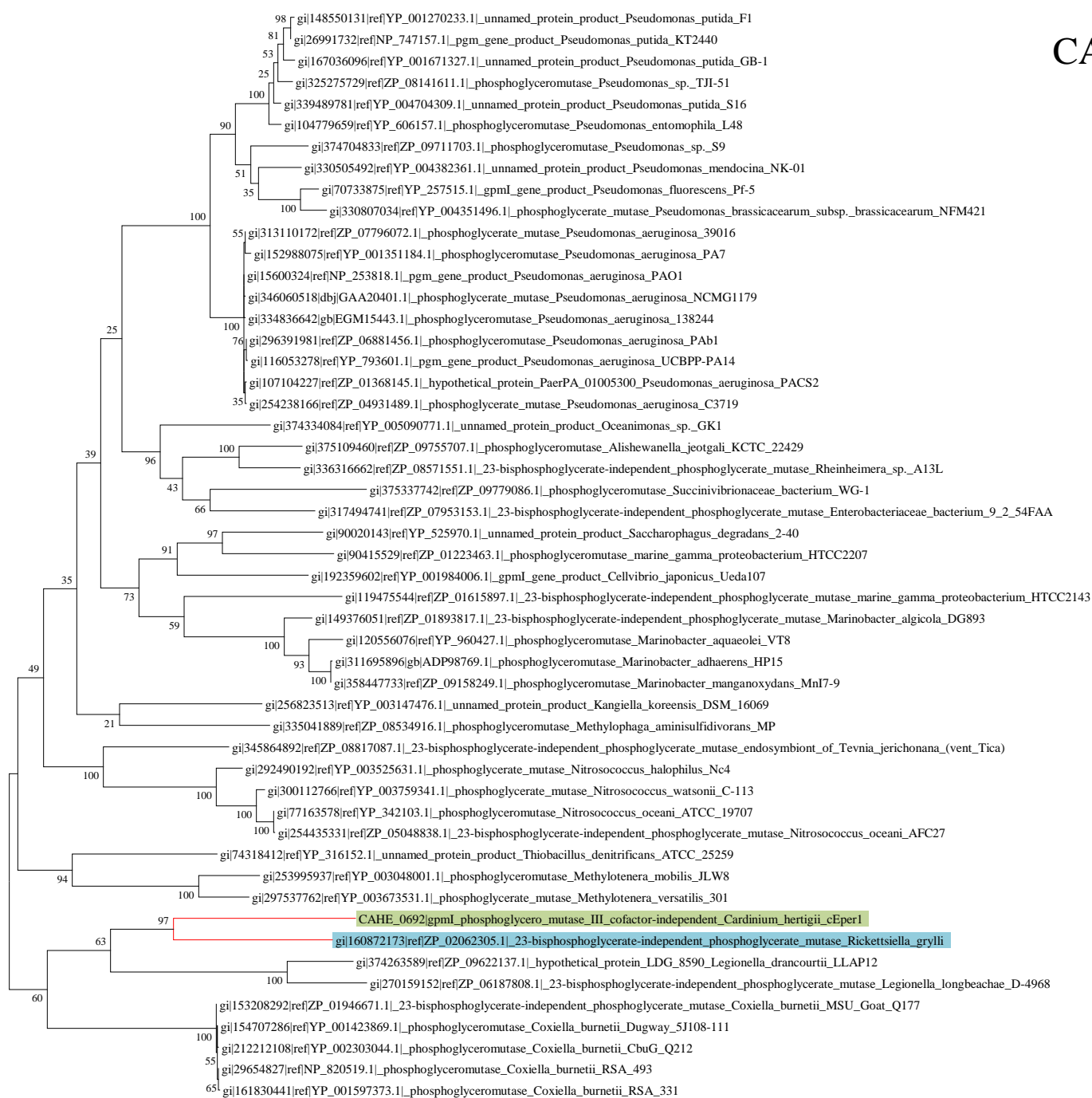

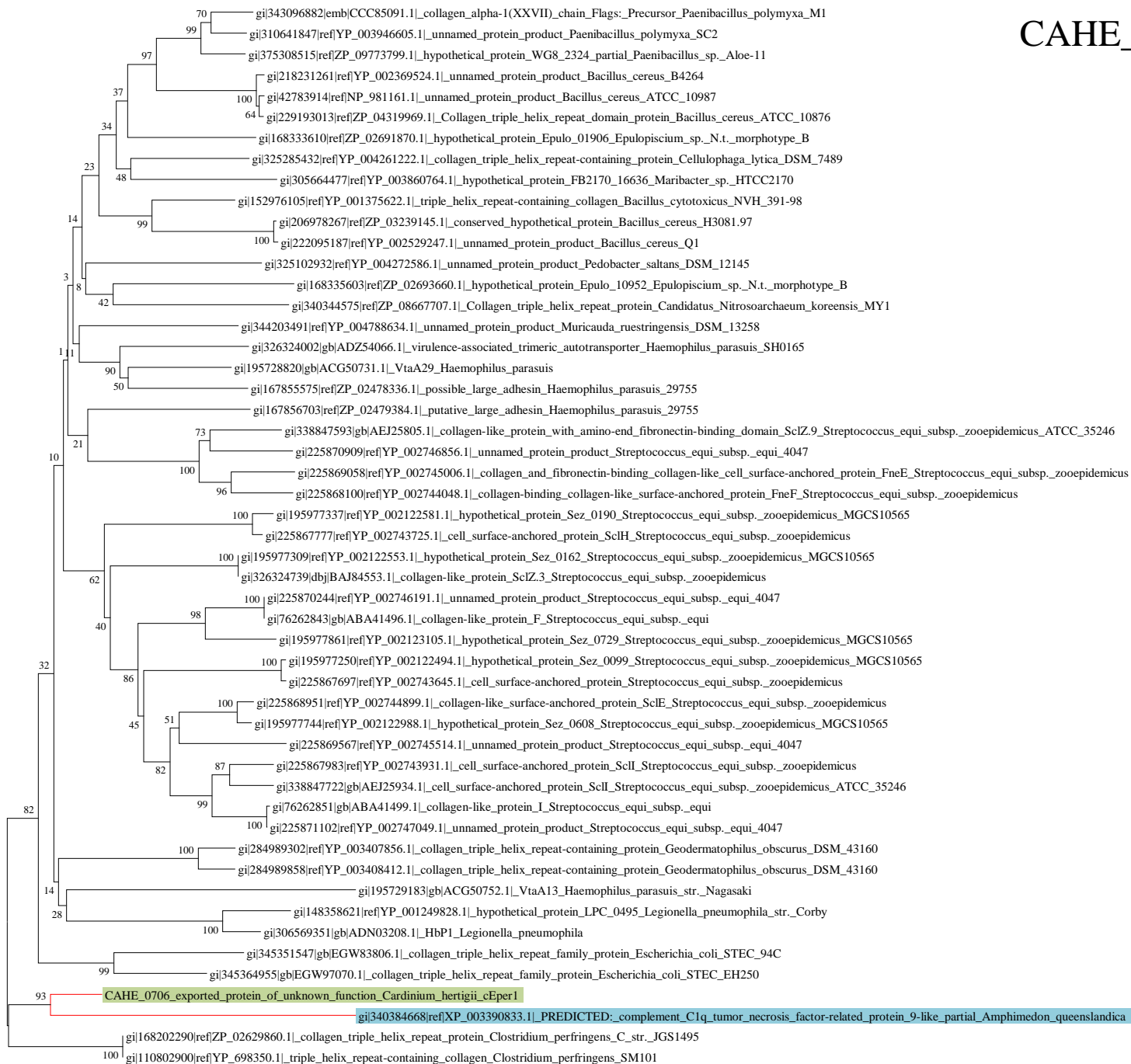

0.1

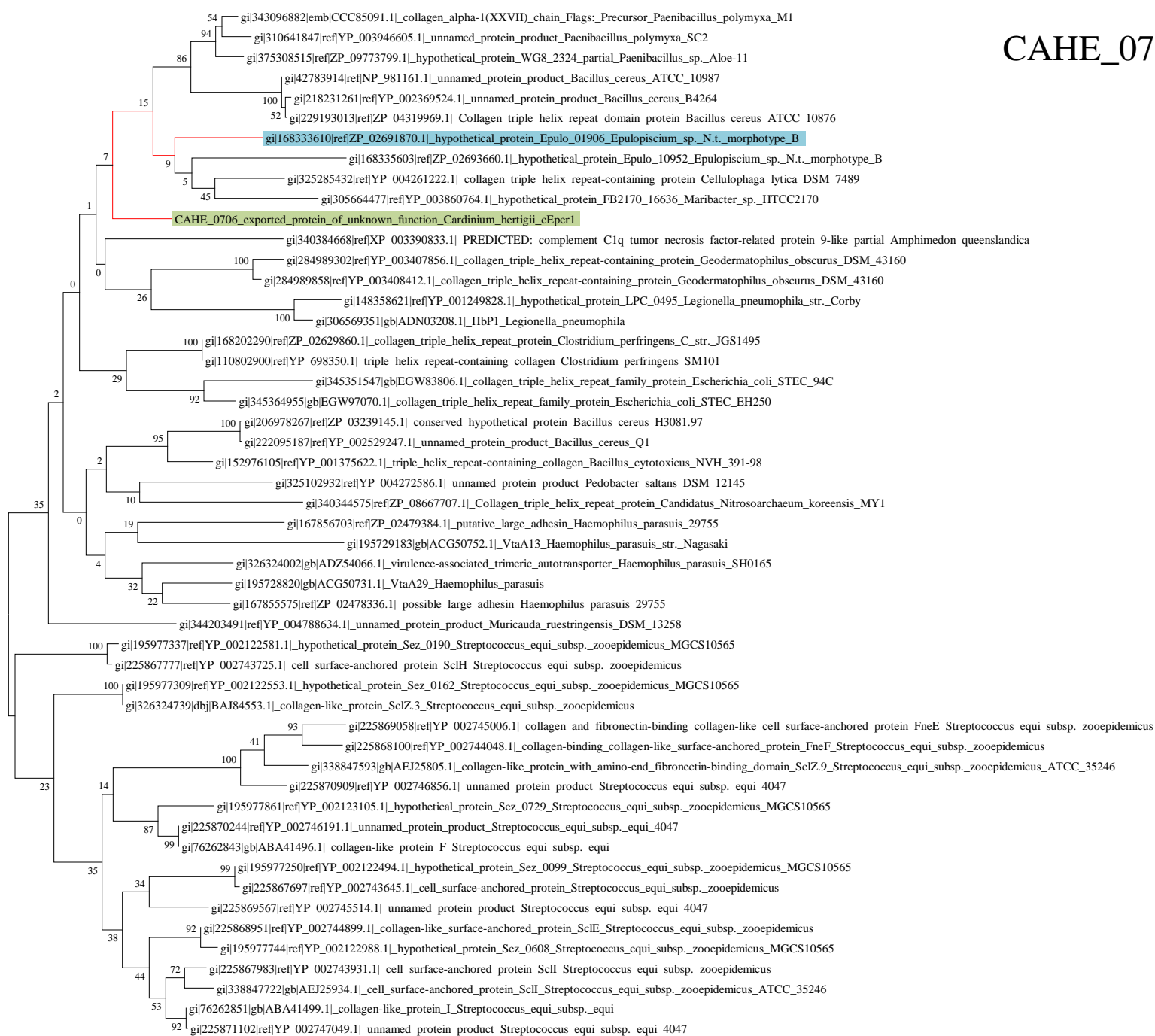

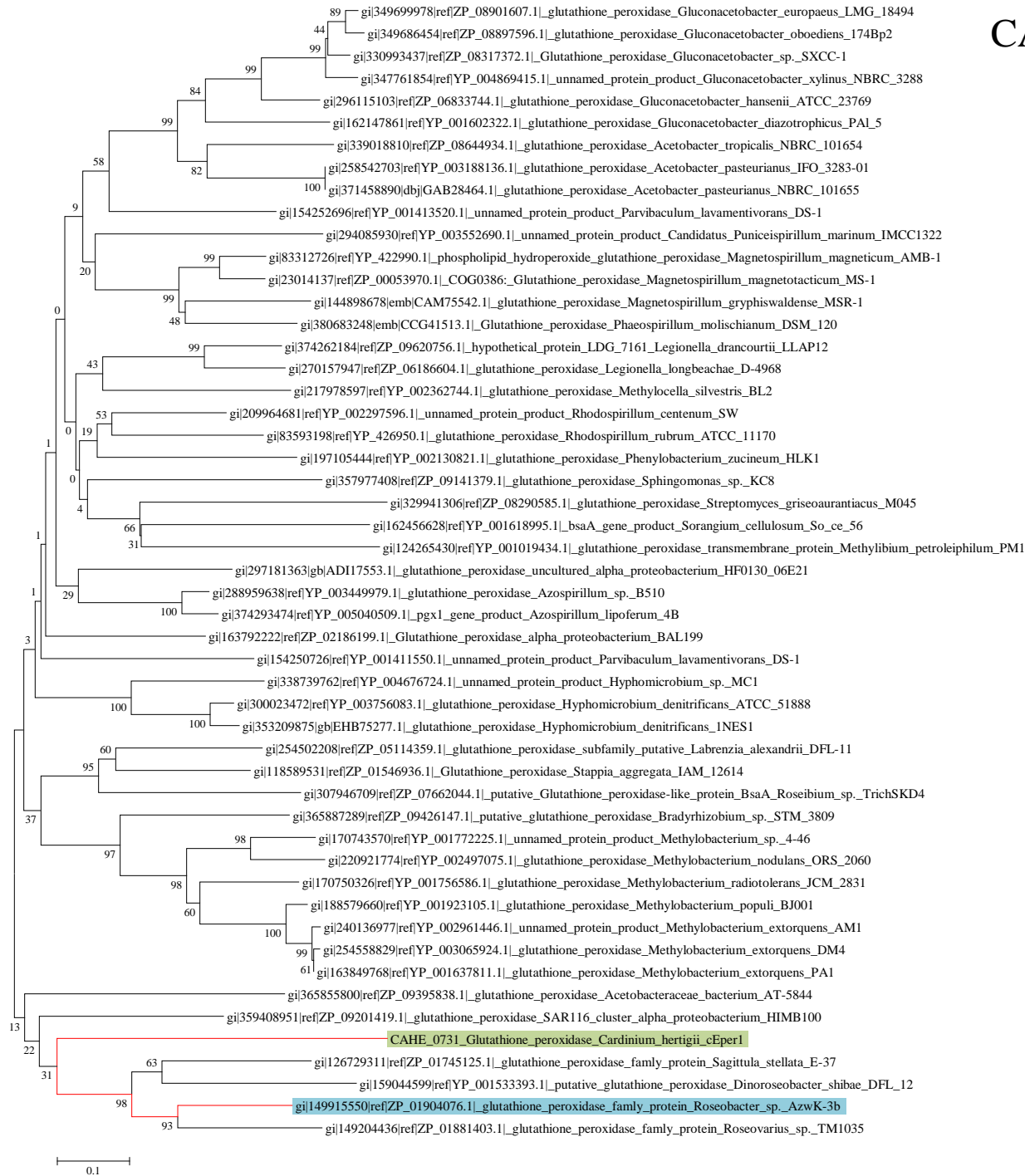

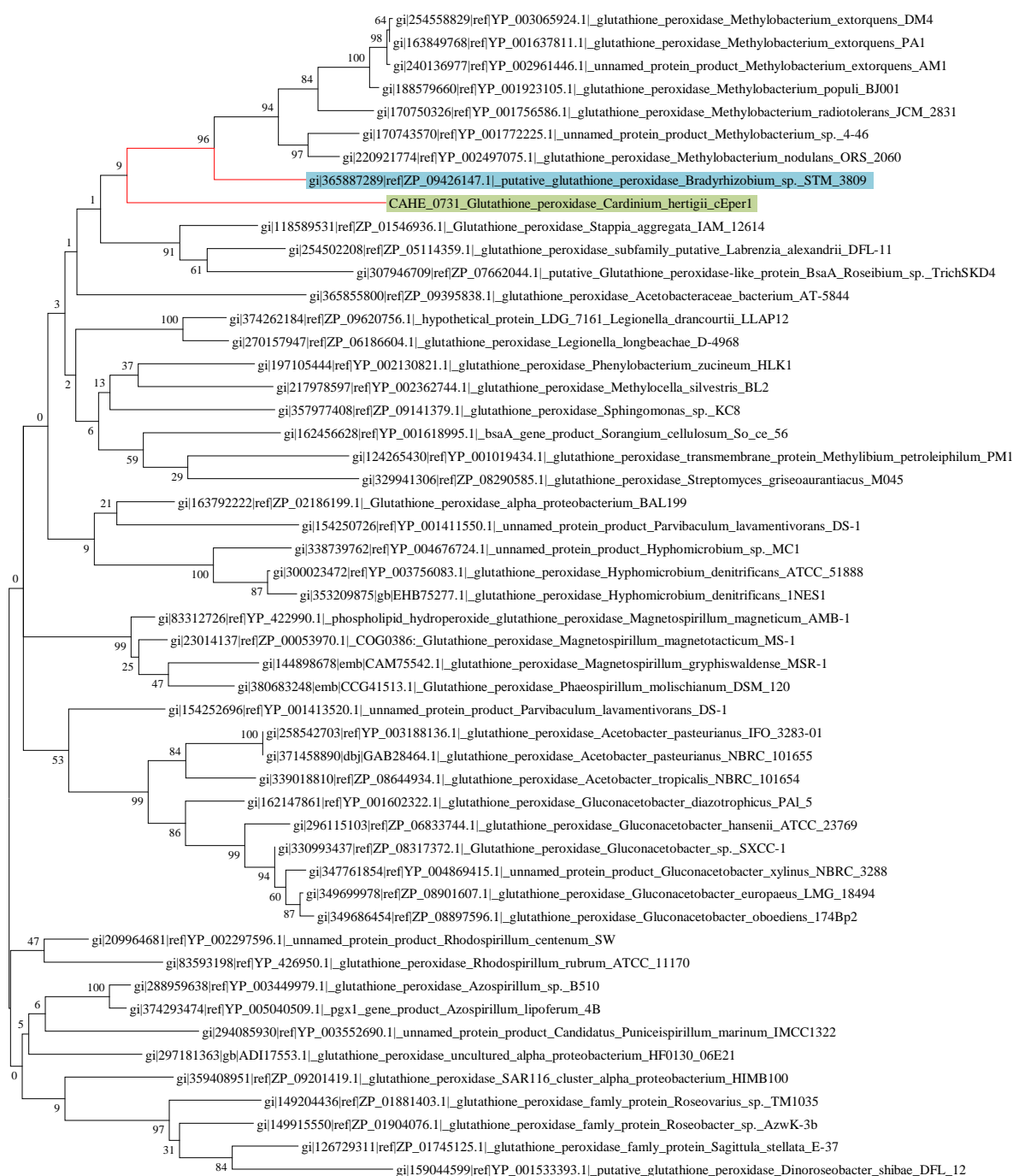

0.1

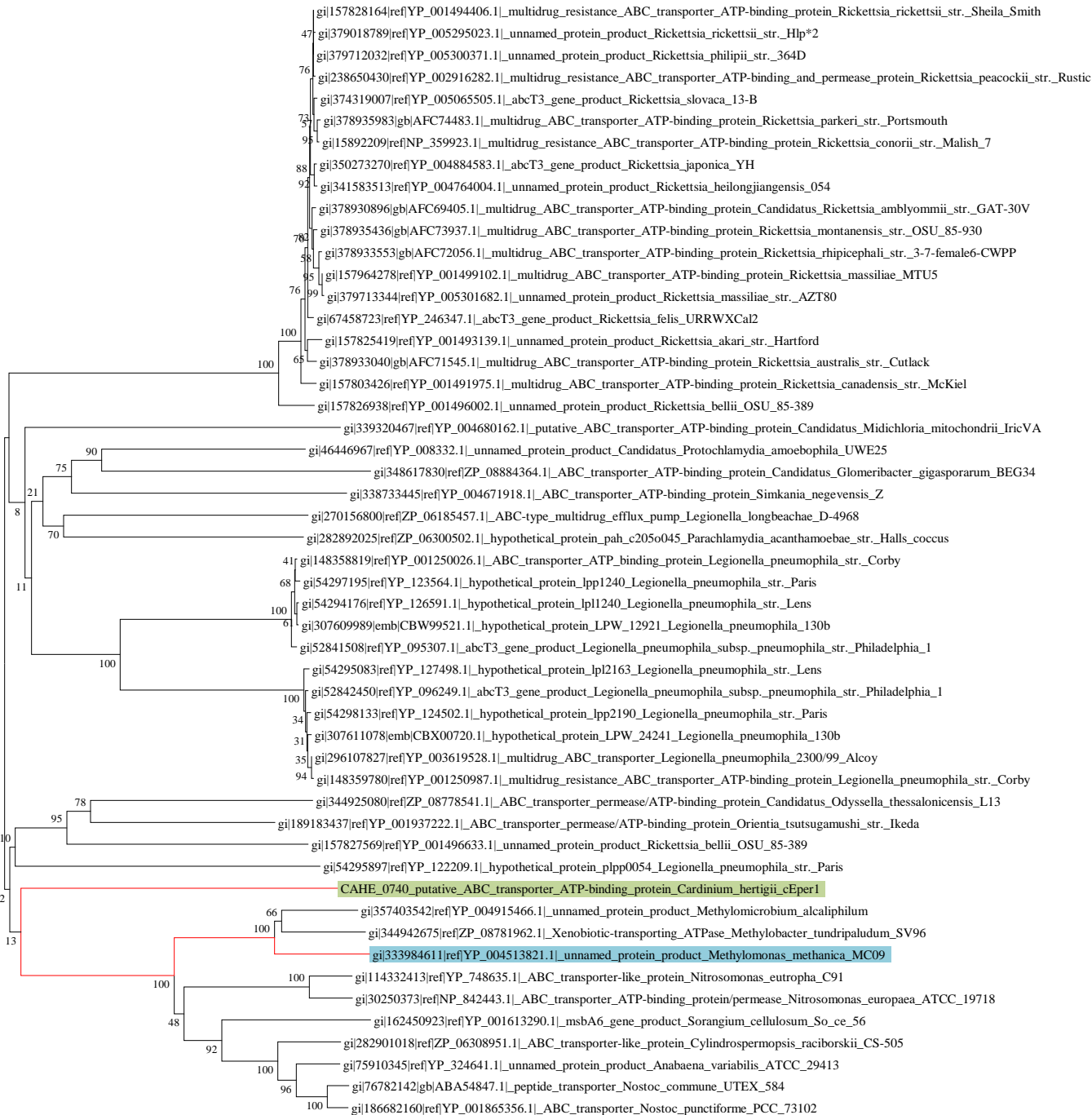

## CAHE\_0740 ML

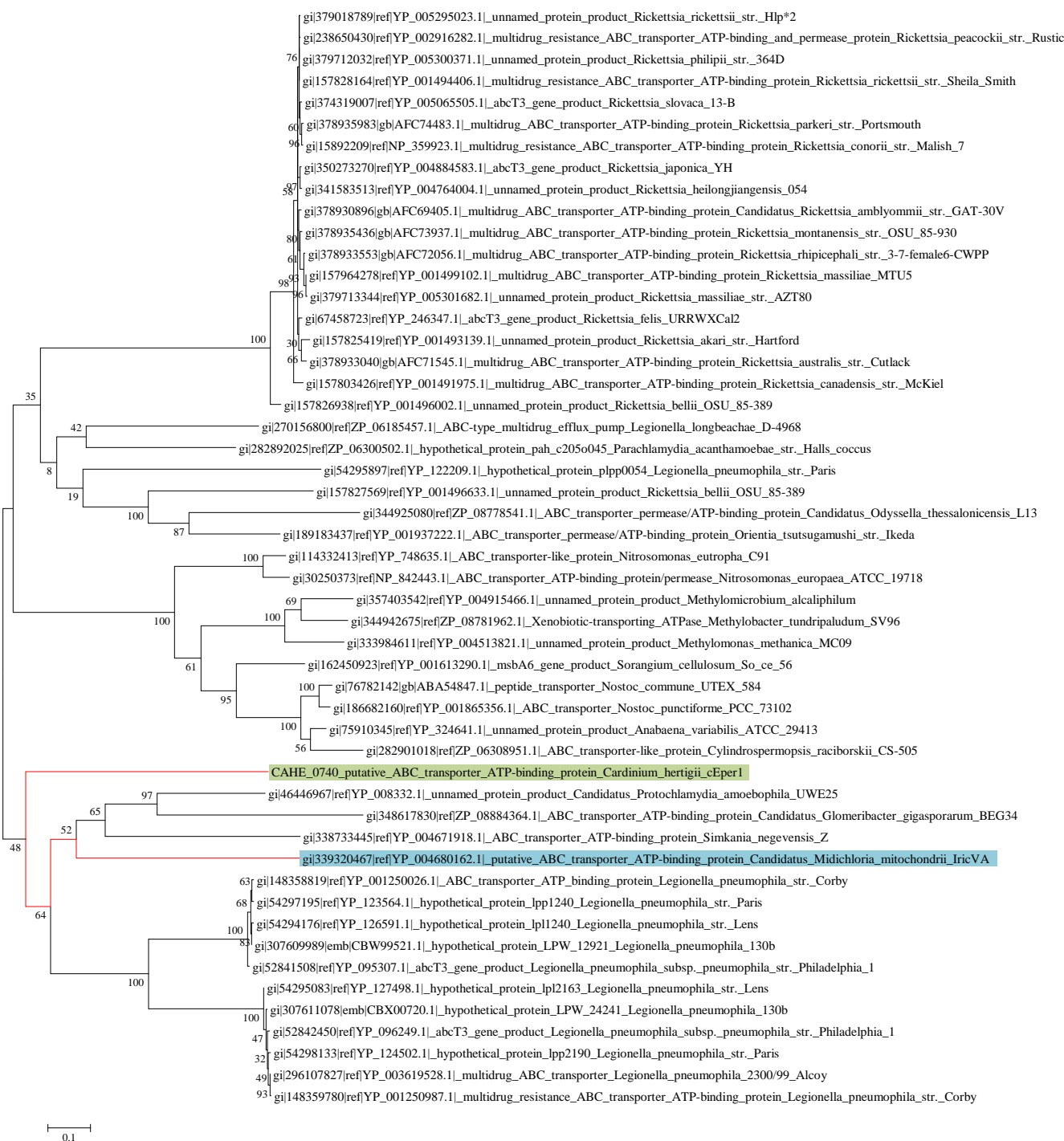

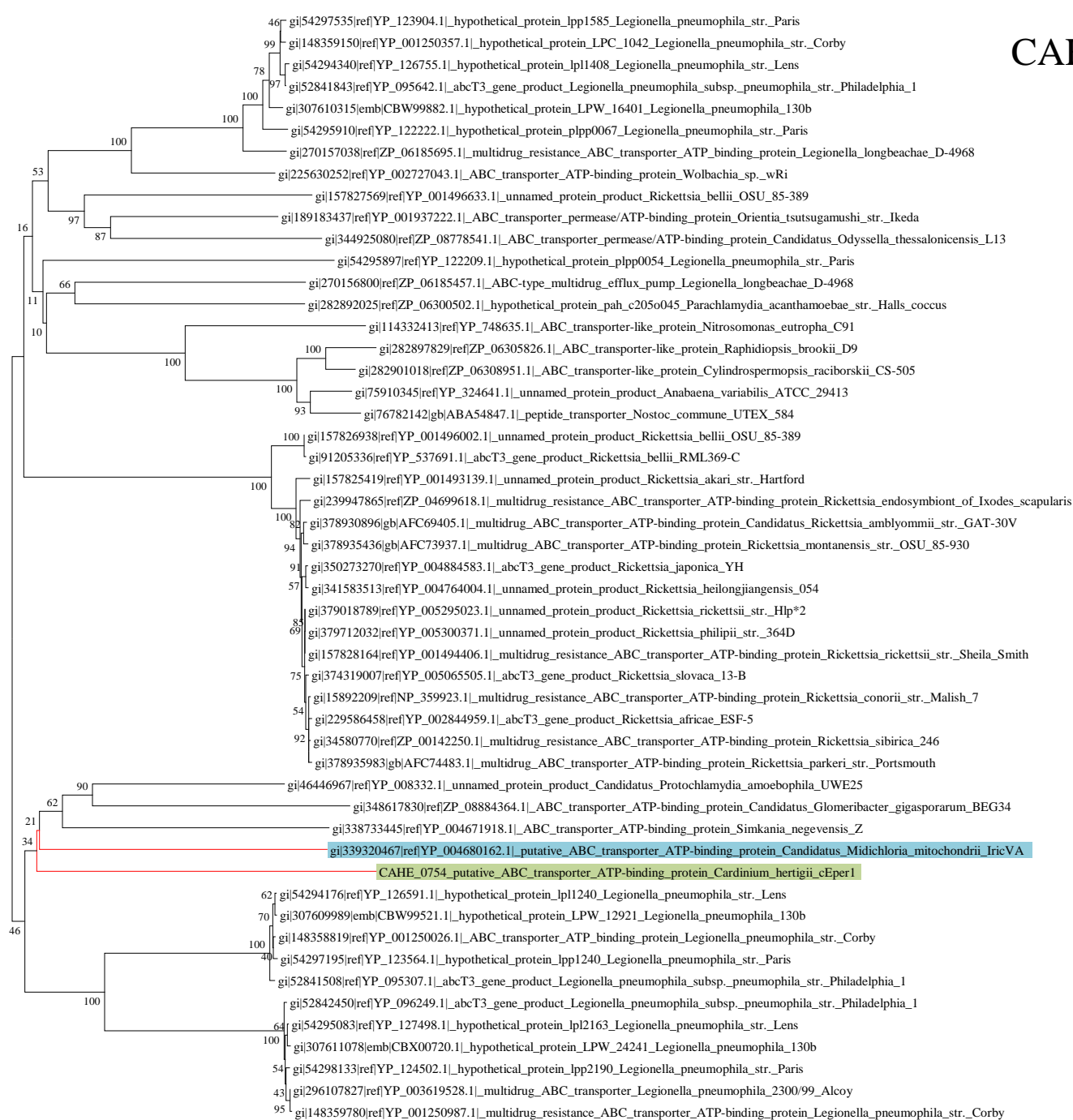

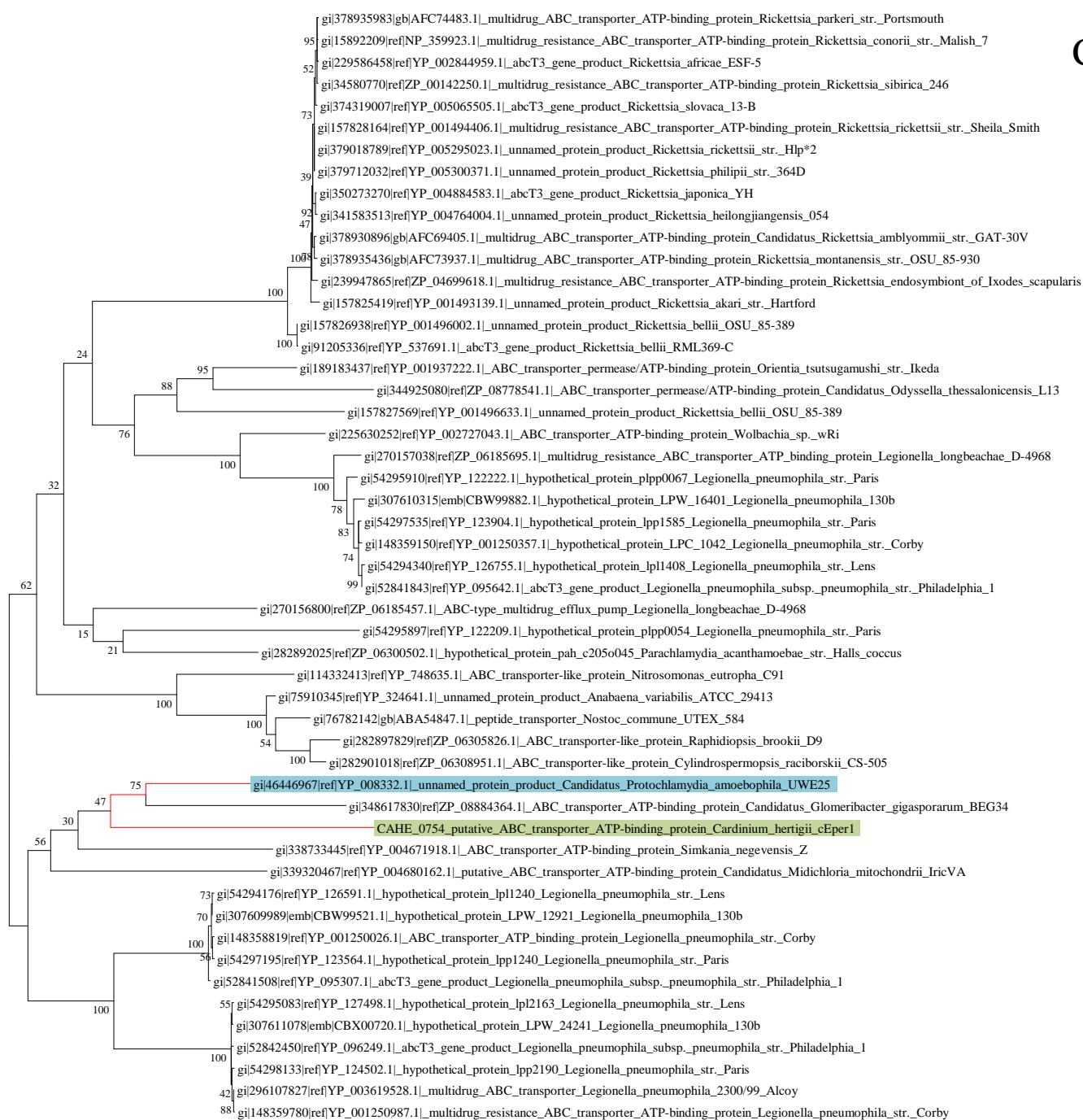

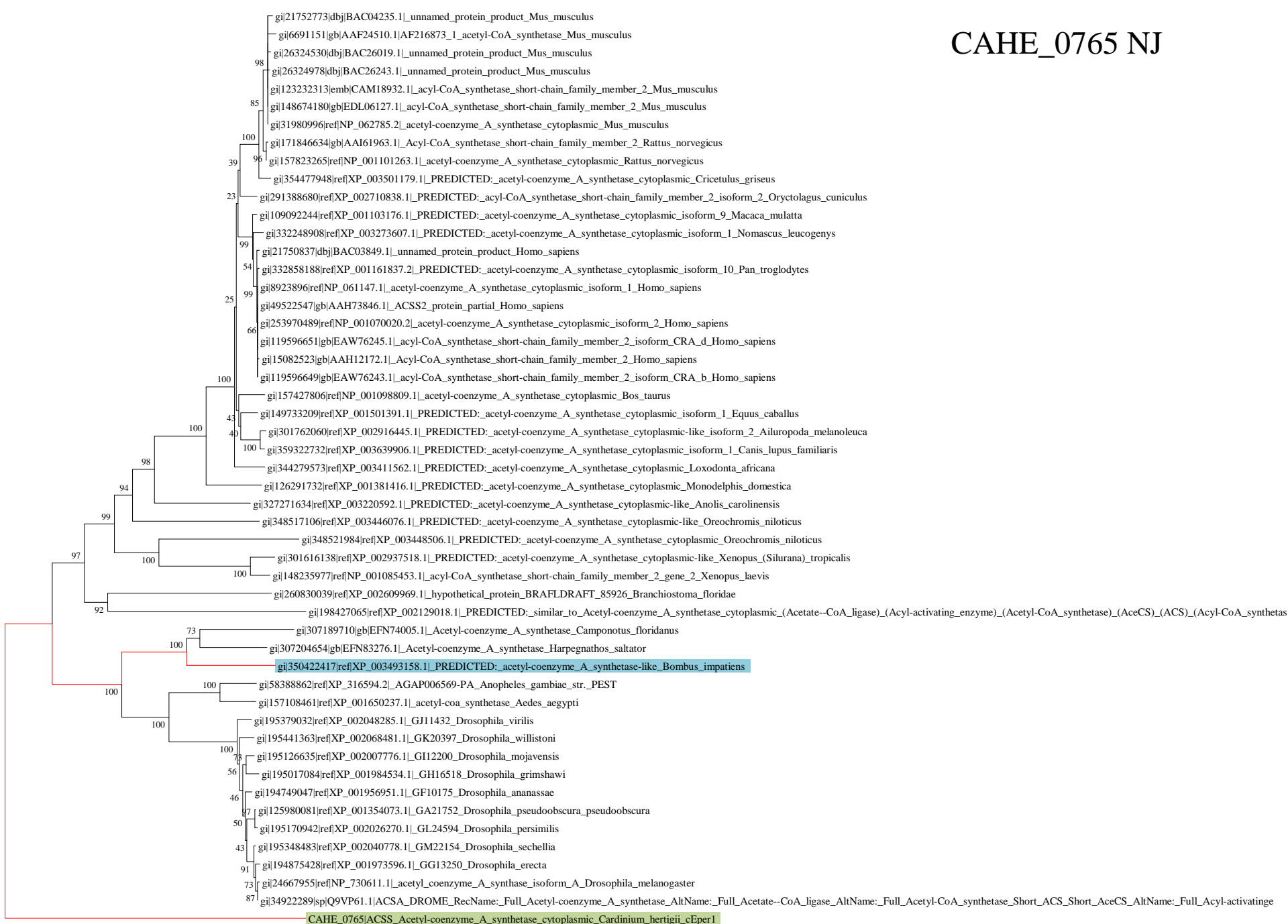

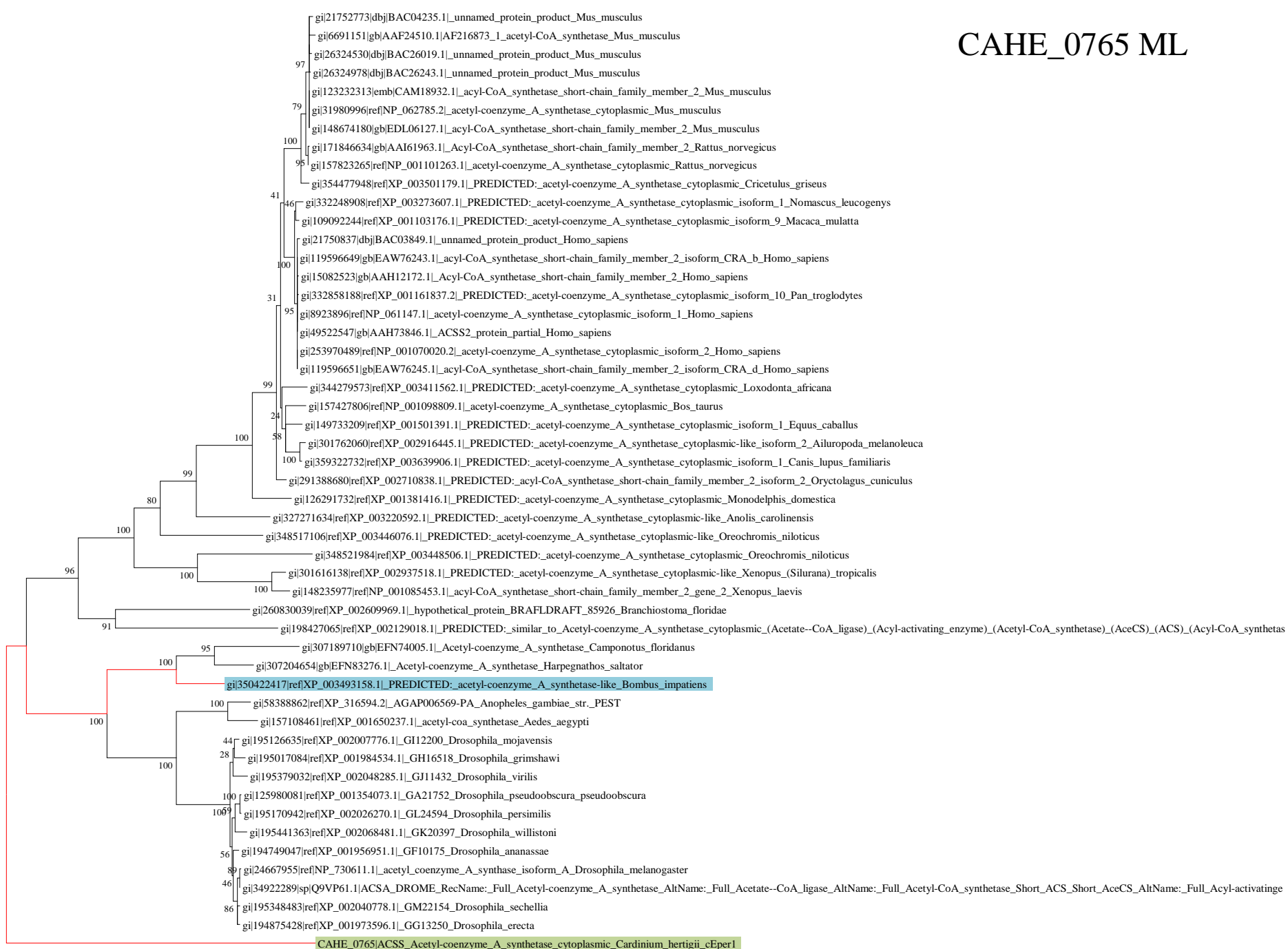

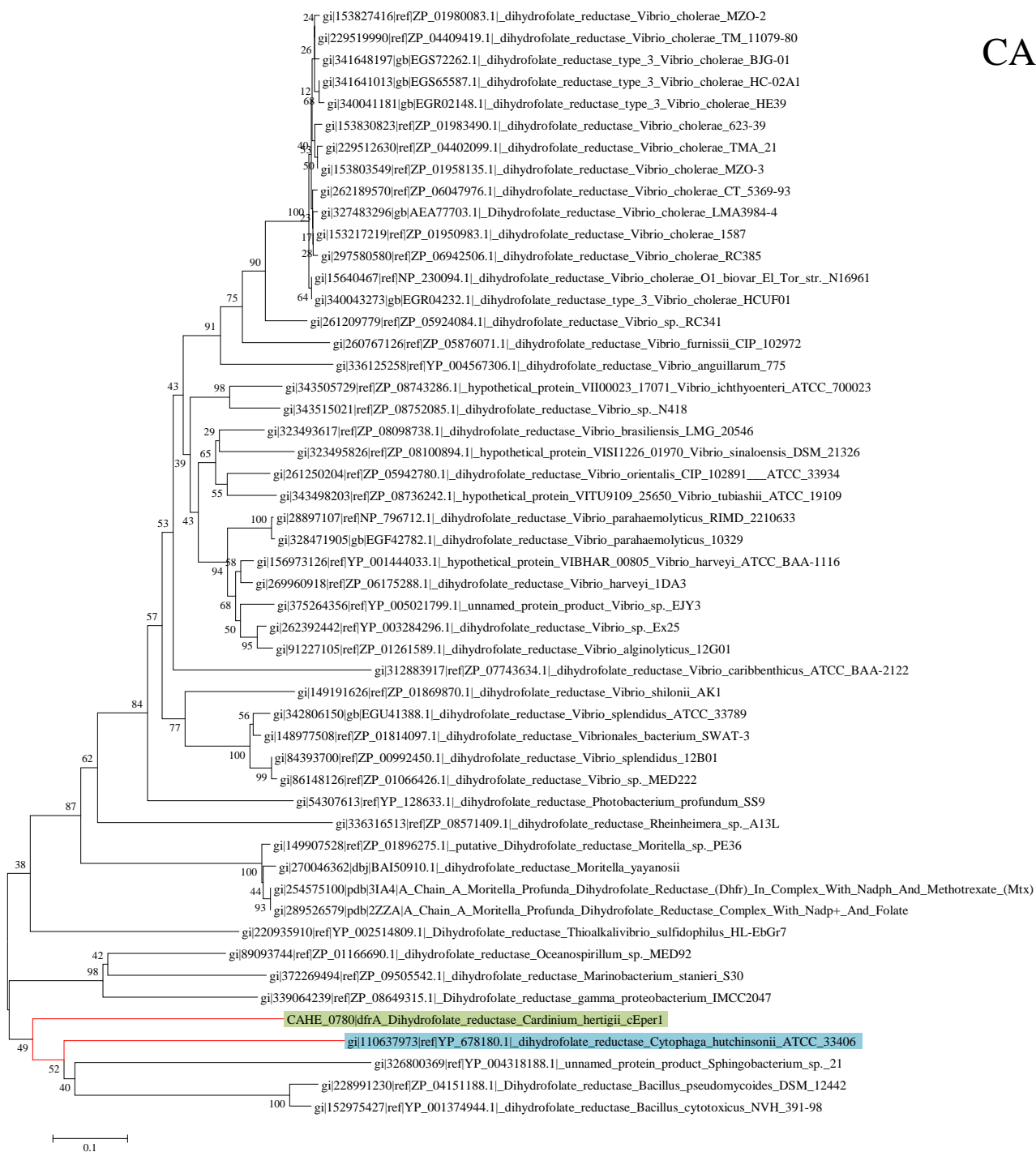

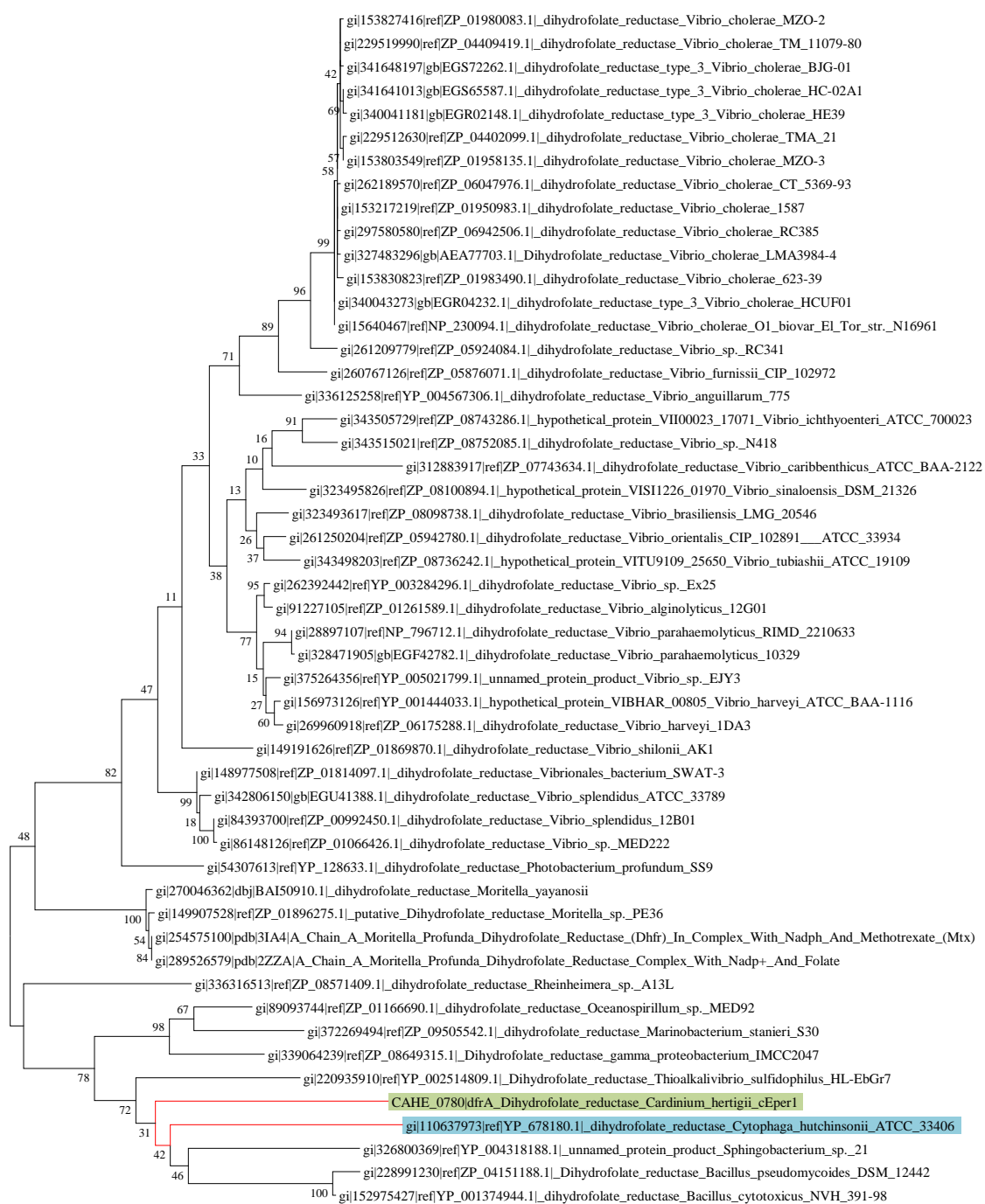

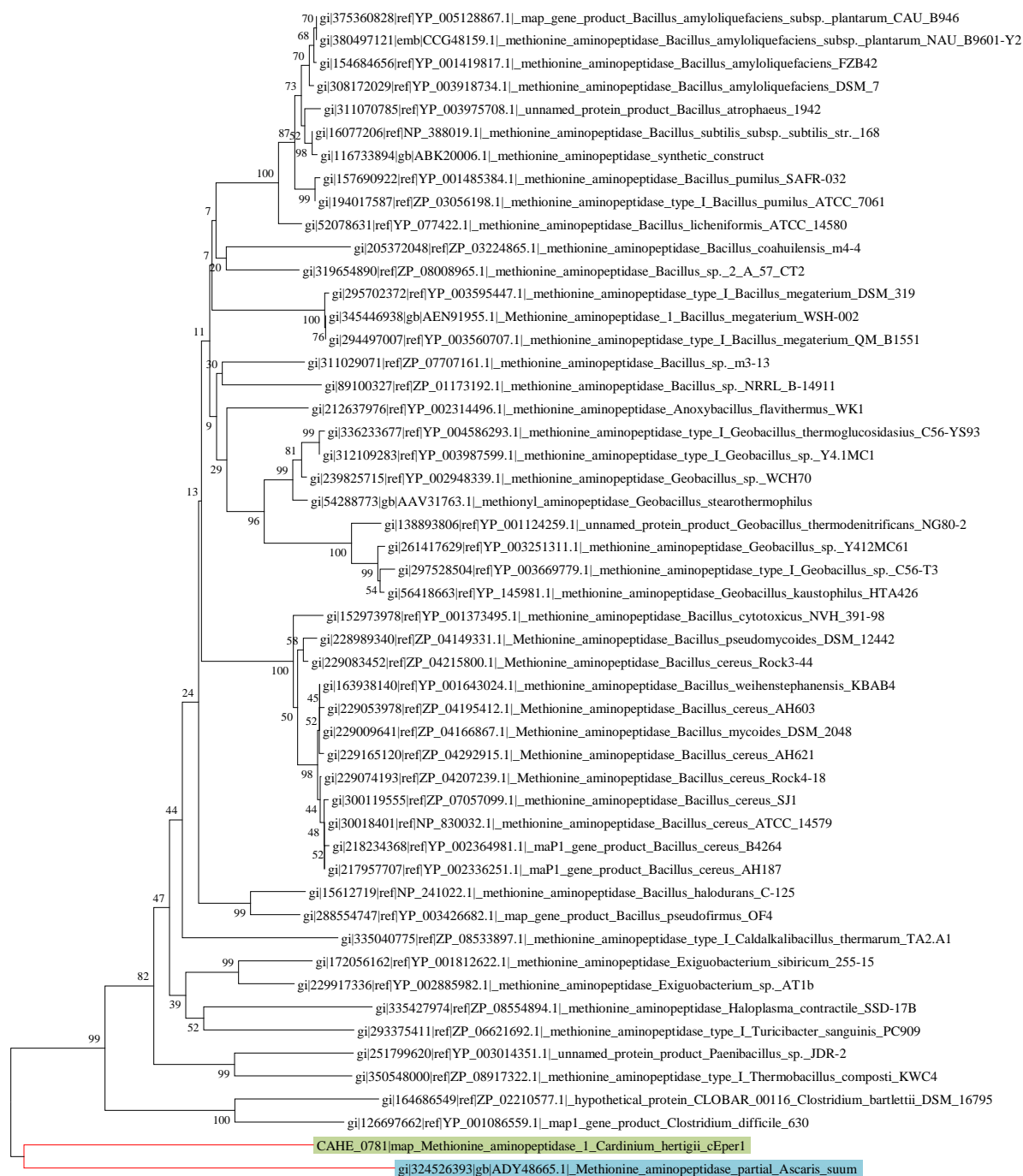

0.05

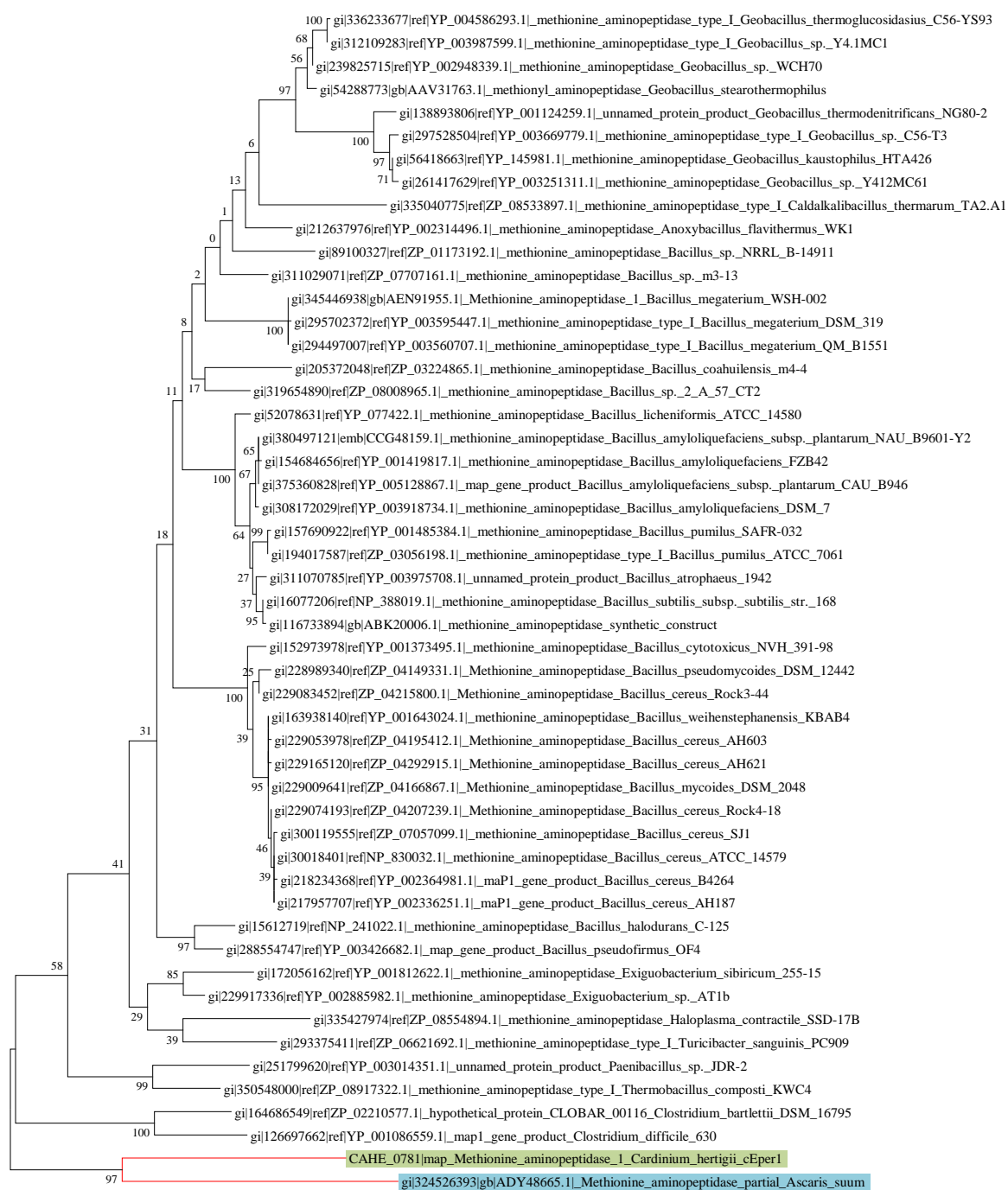

0.1

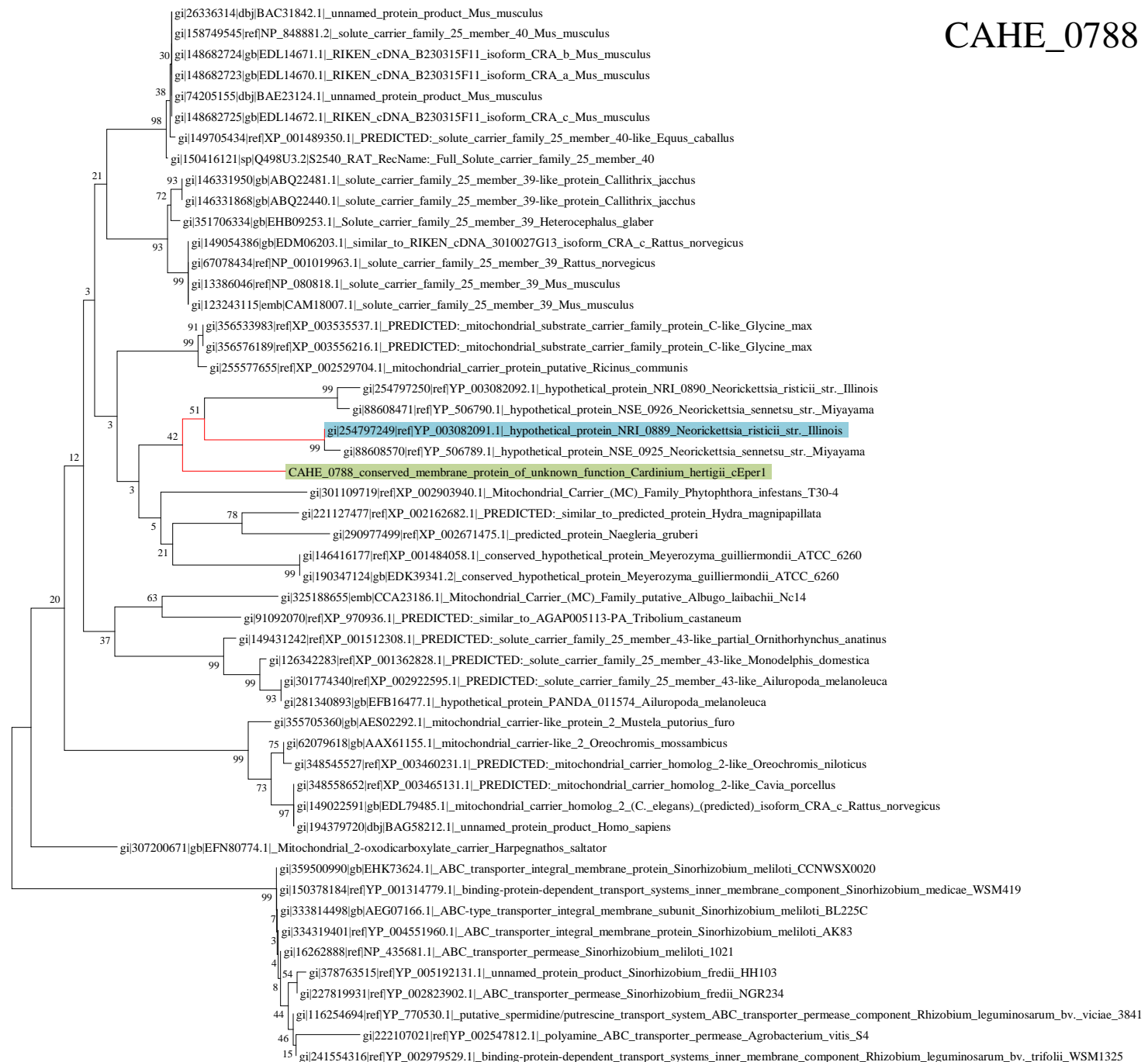

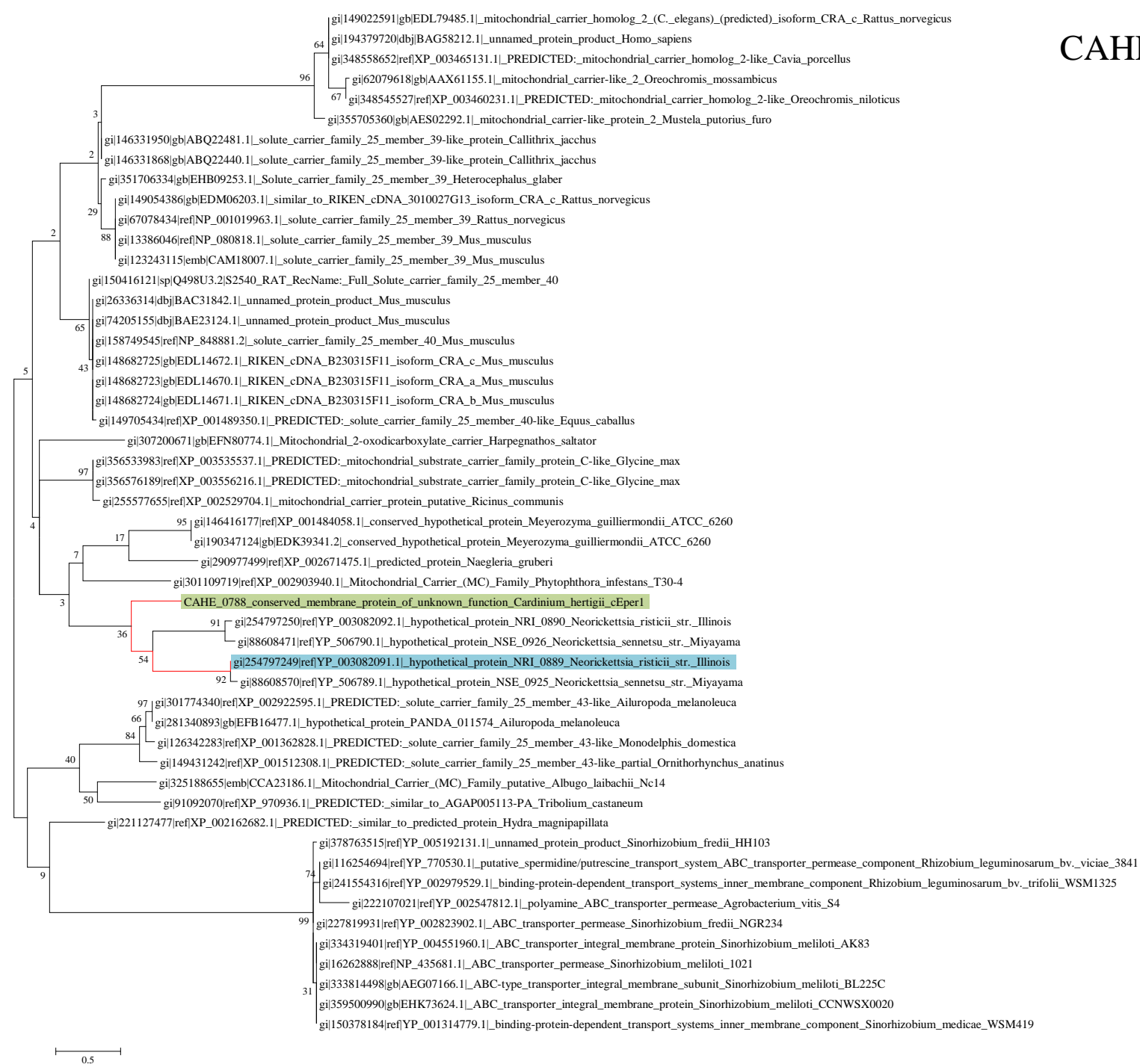

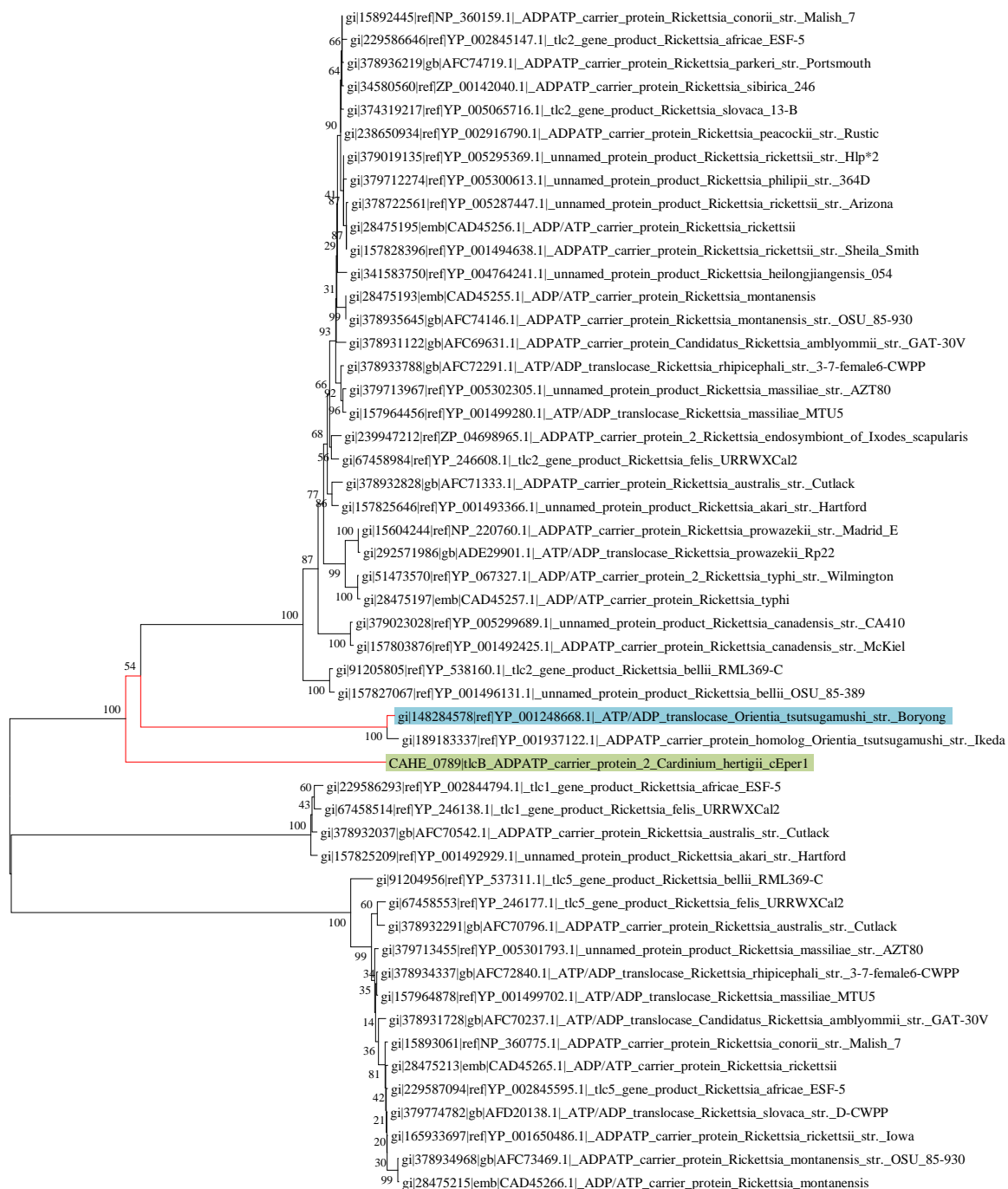

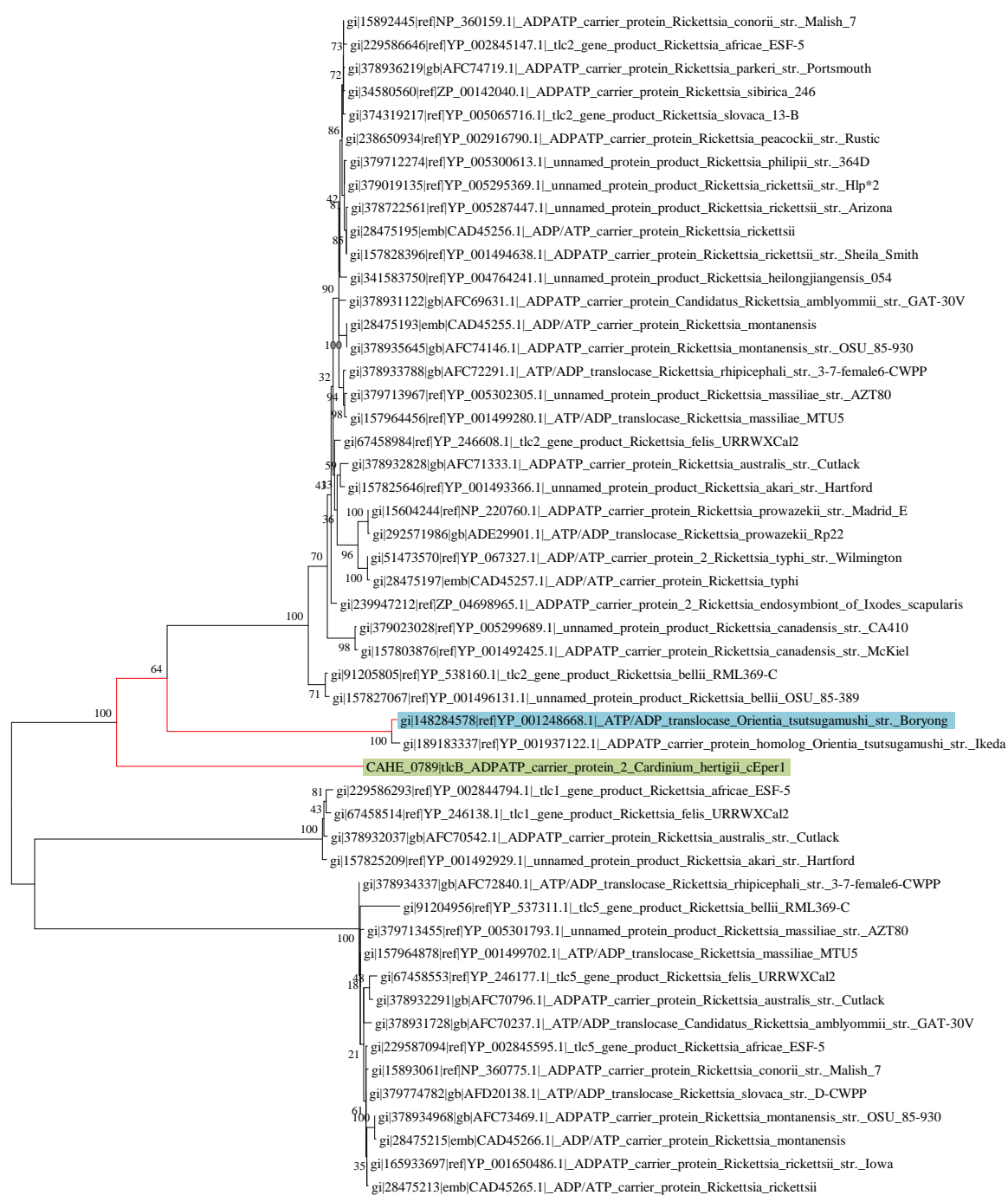

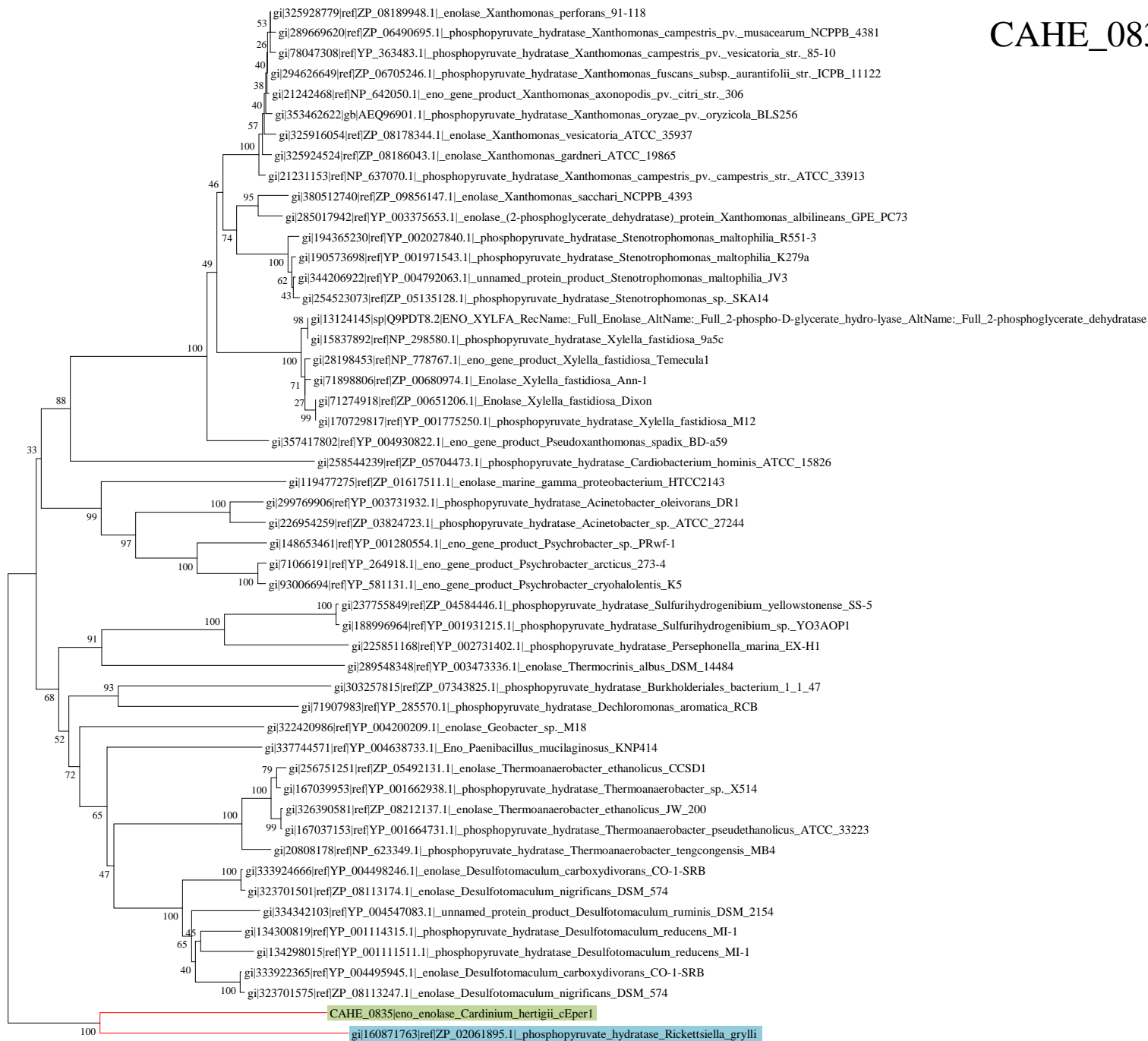

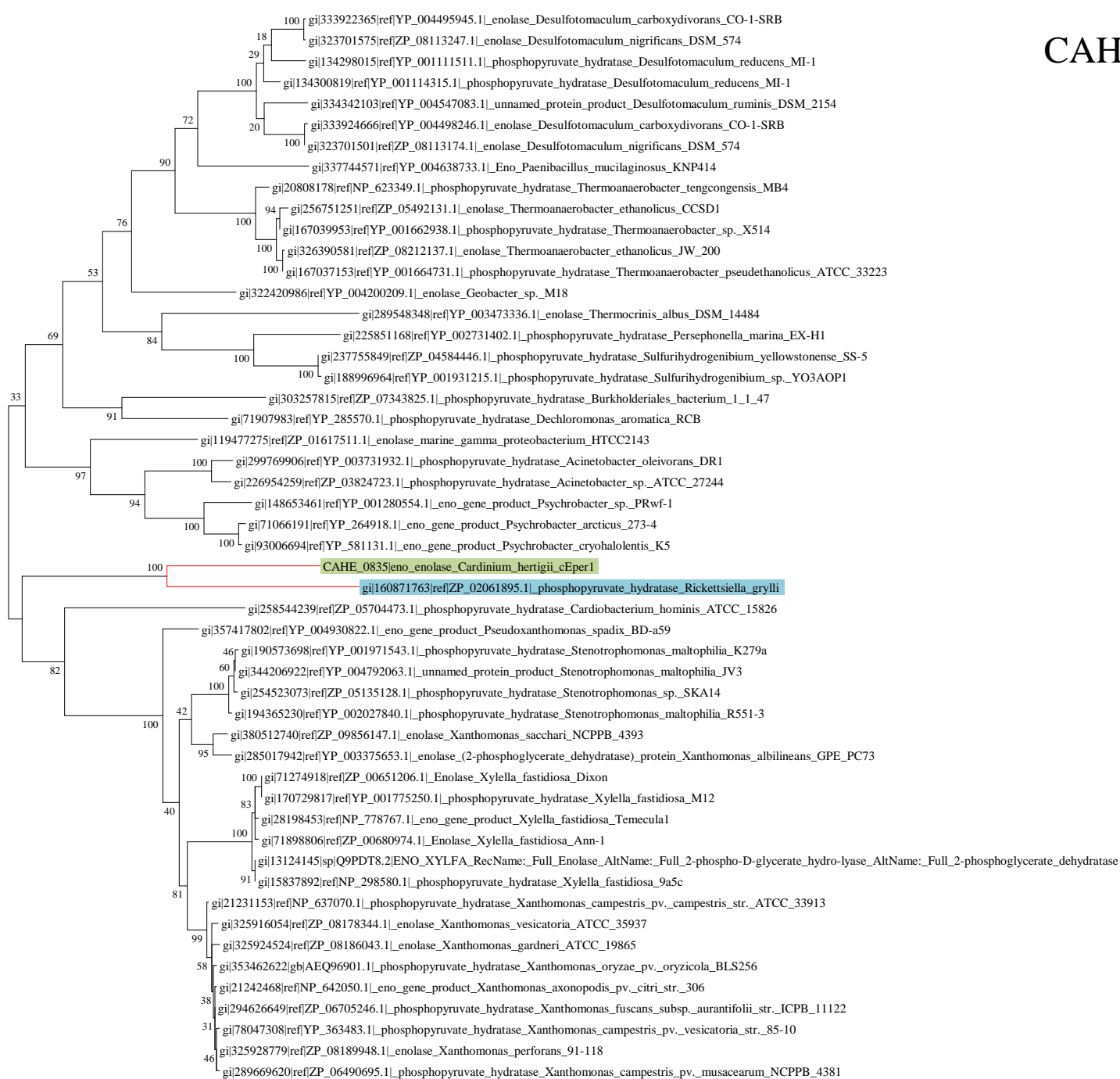

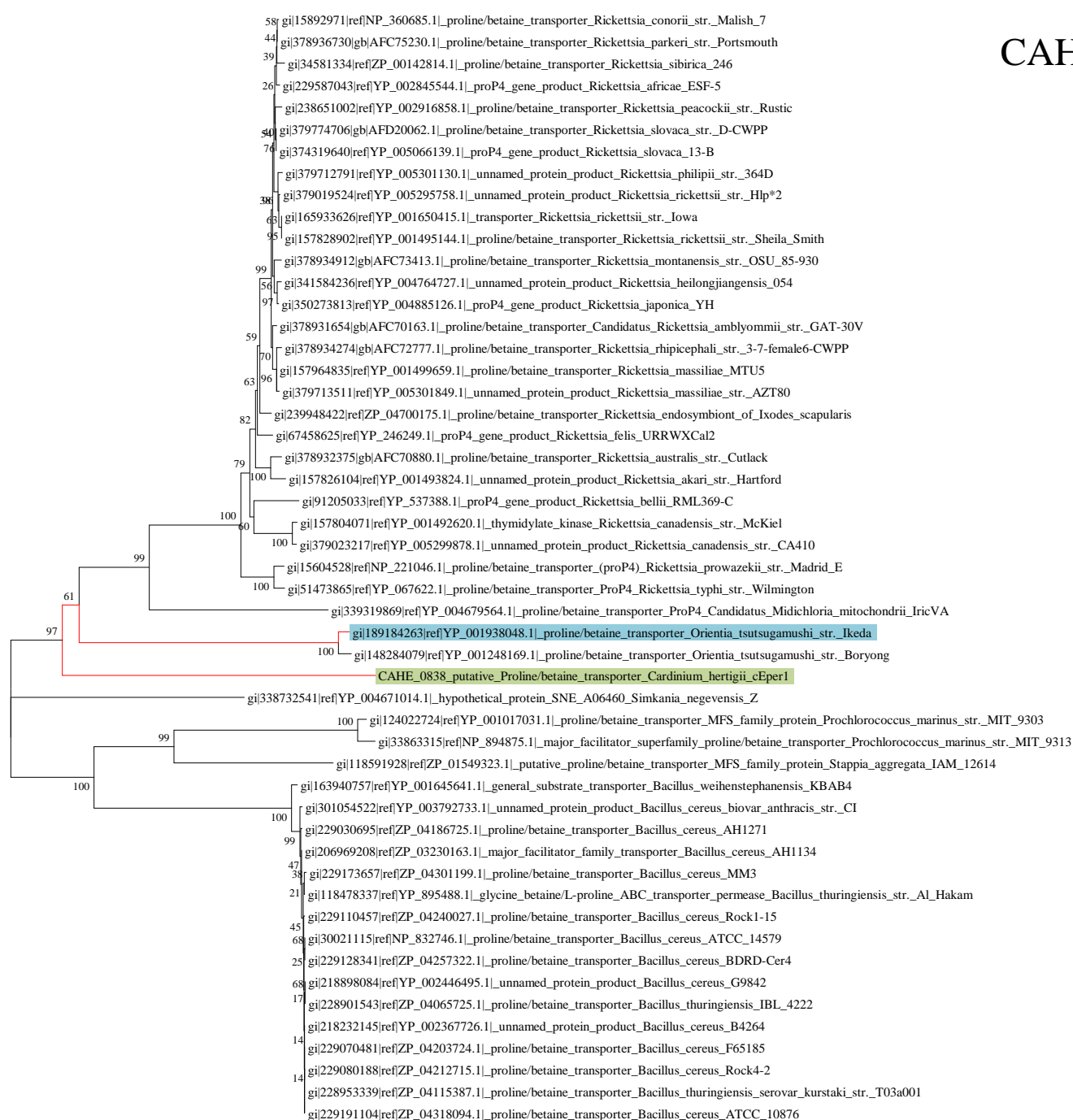

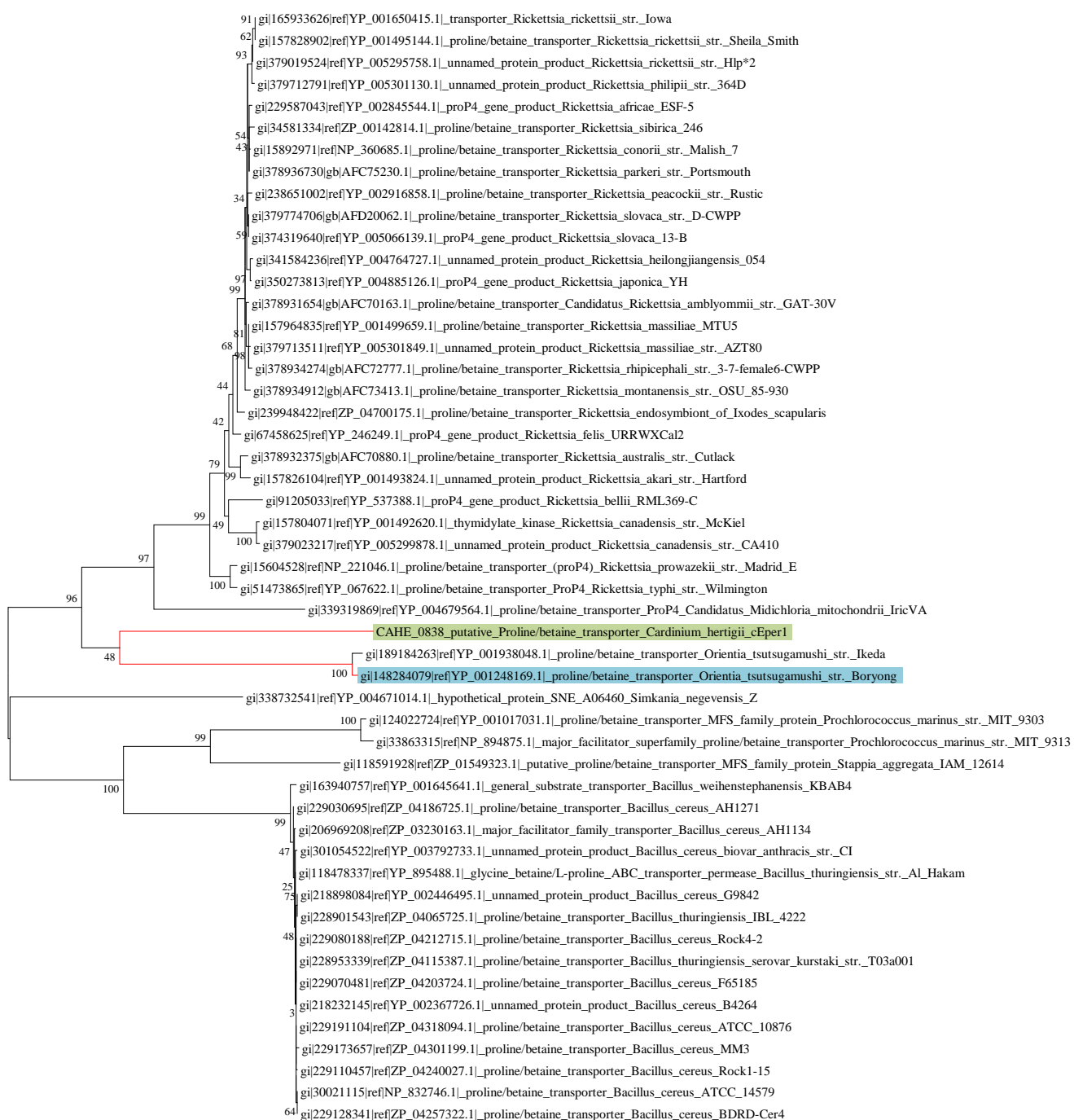

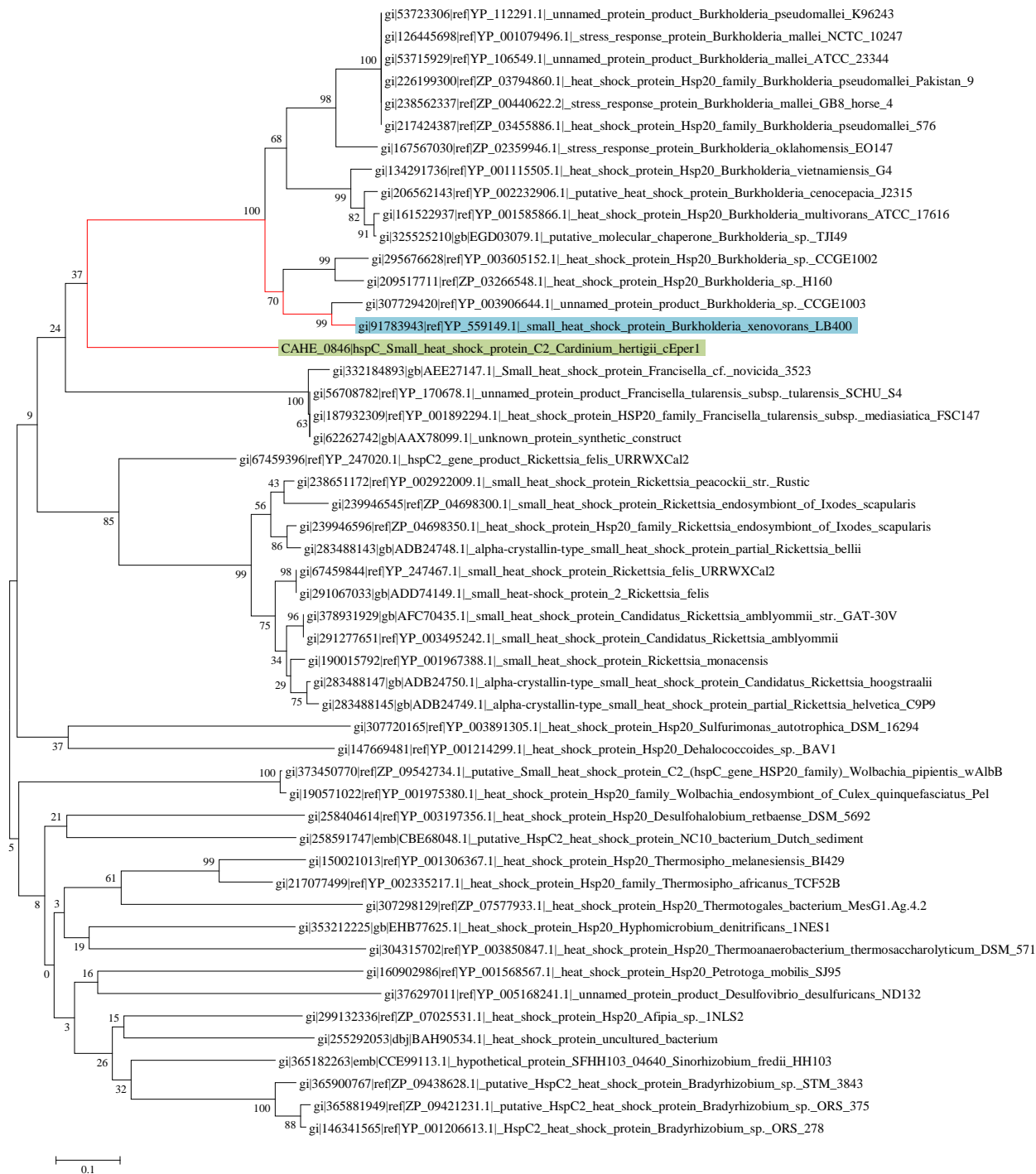

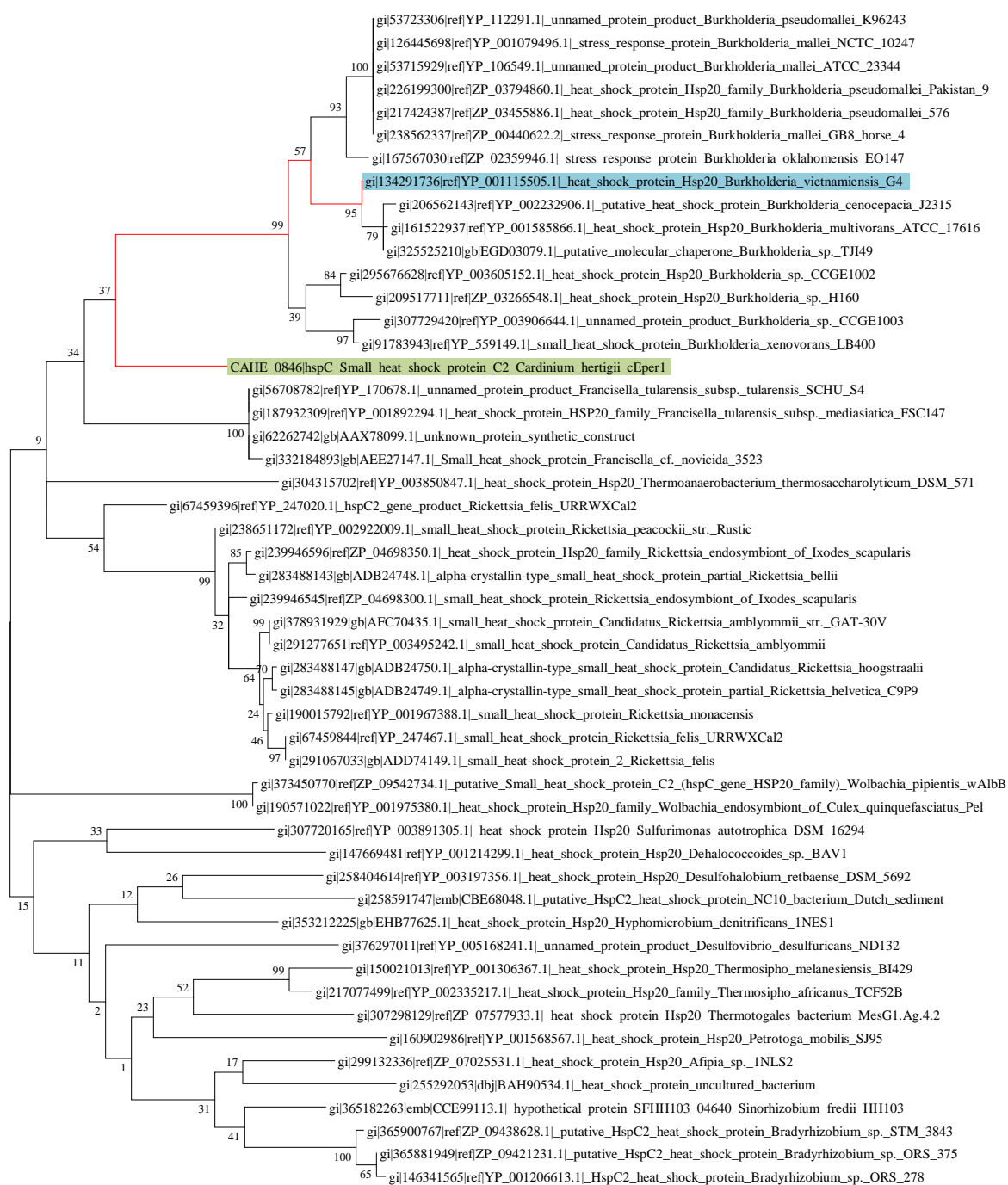

0.2

Supplement: Figure S7 — Phylogenetic relationships of candidate HGT genes of Cardinium hertigii. Phylogenetic trees are based on amino acid sequences and were calculated with MEGA using the neighbor-joining algorithm (NJ) with 2000× bootstrapping and maximum-likelihood algorithm (ML) with 100× bootstrapping. Bootstrap values are indicated at the respective nodes. GenBank accession numbers are indicated. (PDF) [file pgen.1003012.s007.pdf]
