# Supplementary material for: Comparative Genomics Suggests an Independent Origin of Cytoplasmic Incompatibility in Cardinium hertigii
Source: PLoS Genet. 2012 Oct 25;8(10):e1003012. doi: 10.1371/journal.pgen.1003012 (PMC3486910; doi:10.1371/journal.pgen.1003012)
Supplement: Figure S2 — Conservation of the RING-like domain encoded in CAHE_p0026. Comparison of the domain found in CAHE_p0026 with RING-like domains showing E3 ubiquitin ligase activity according to [44]. The domains RING-HC and RING-H2 represent the two major subcategories of RING finger domains (depending on whether a Cys or His occupies the fifth coordination site); Mdm2, murine double minute 2 protein; RBQ-1, retinoblastoma binding protein 6 (RBBP6); RBX1, RING-box protein 1; Cnot4, CCR4-NOT transcription complex subunit 4. Only conserved amino acid residues indicative for the RING finger domain are shown. Cys, cysteine; His, histidine; X, any amino acid; subscript number corresponds to number of amino acid. (PDF) [file pgen.1003012.s002.pdf]

**CAHE_p0026**	Cys - X_2 - Cys - X_{18}	- Cys- X_1	- His - X_2	- His - X_2 - Cys - X_{16}	- Cys - X_2 - Cys
RING-HC	Cys - X_2 - Cys - $X_{(9-39)}$	- Cys- $X_{(1-3)}$	- His - $X_{(2-3)}$	- Cys - X_2 - Cys - $X_{(4-48)}$	- Cys - X_2 - Cys
RING-H2	Cys - X_2 - Cys - $X_{(9-39)}$	- Cys- $X_{(1-3)}$	- His - $X_{(2-3)}$	- His - X_2 - Cys - $X_{(4-48)}$	- Cys - X_2 - Cys
C2H2C4 (Mdm2)	Cys - X_2 - Cys - X_{10}	- His- X_4	- His - X_3	- Cys - X_2 - Cys - X_{10}	- Cys - X_2 - Cys
RBQ-1	Cys - X_2 - Cys - X_{11}	- Cys- X_2	- Asn- X_3	- Cys - X_2 - Cys - X_{12}	- Cys - X_2 - Cys
RBX1	Cys - X_2 - Cys - X_{29}	- Cys- X_1	- His - X_2	- His - X_2 - Cys - X_{10}	- Cys - X_2 - Cys
C4H4 (Cnot4)	Cys - X_2 - Cys - X_{13}	- Cys- X_1	- Cys -X_4	- Cys - X_2 - Cys - X_{11}	- Cys - X_2 - Cys
