# Supplementary material for: Comparative Genomics Suggests an Independent Origin of Cytoplasmic Incompatibility in Cardinium hertigii
Source: PLoS Genet. 2012 Oct 25;8(10):e1003012. doi: 10.1371/journal.pgen.1003012 (PMC3486910; doi:10.1371/journal.pgen.1003012)
Supplement: Figure S5 — Phylogenetic analysis of the biotin biosynthesis cluster of Cardinium hertigii. Tree calculations were performed using the maximum likelihood algorithm (1000 bootstrap resamplings) with a concatenated dataset of six biotin synthesis proteins (BioB, BioF, BioH, BioC, BioD and BioH; Table S11) of bacteria from eight different phyla. Genes and their genomic organization are indicated as colored boxes. Breaks in the black bars denote noncontiguous genes. Boxes above and below the black bars indicate genes encoded on the plus and minus strand, respectively. Bootstrap values are indicated at the respective node. Note that the Cardinium genes are synthenic with those of the putative rickettsial donors. (PDF) [file pgen.1003012.s005.pdf]

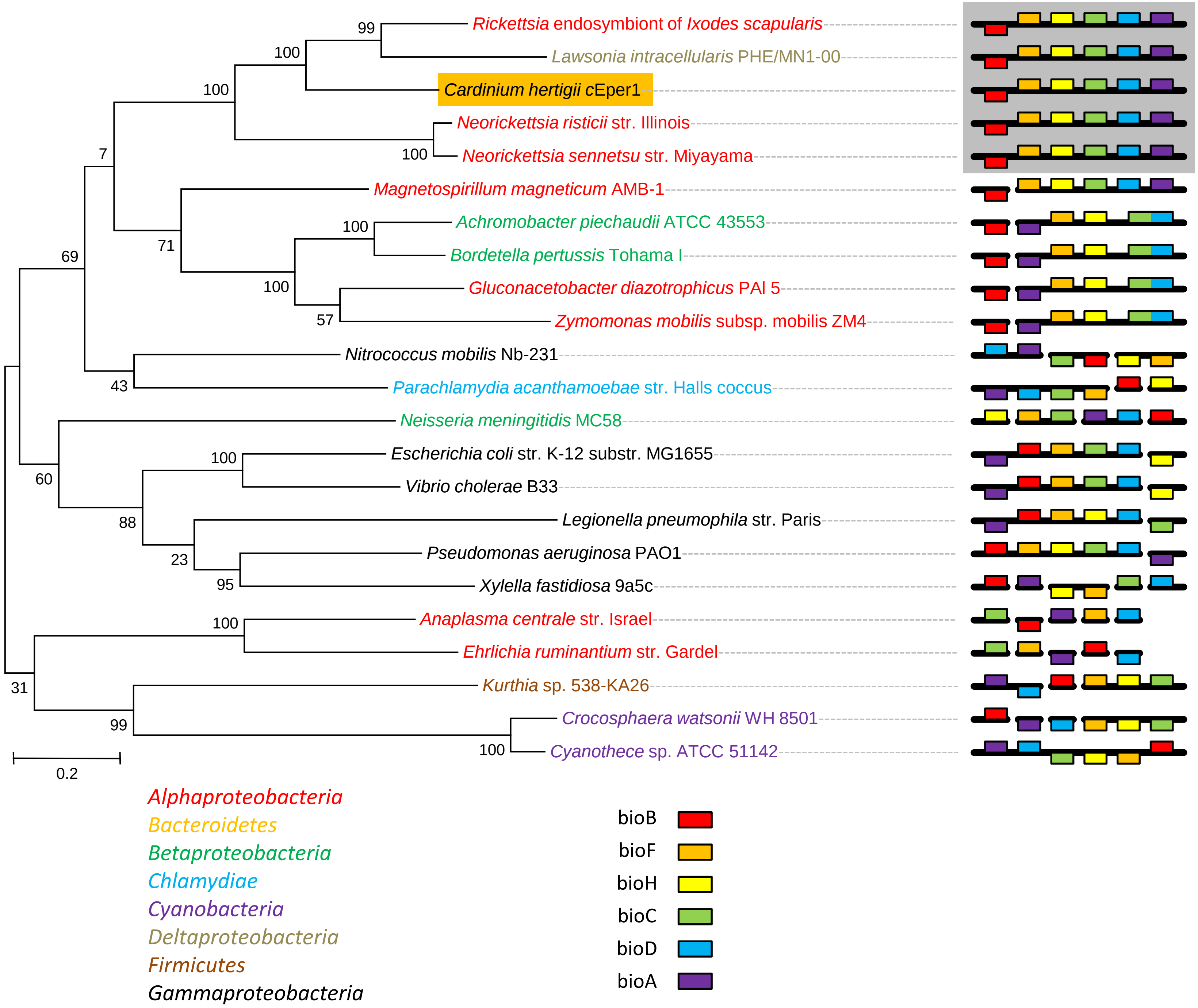
Rickettsia endosymbiont of Ixodes scapularis
Lawsonia intracellularis PHE/MN1-00
Cardinium hertigii cEper1
Neorickettsia risticii str. Illinois
Neorickettsia sennetsu str. Miyayama
Magnetospirillum magneticum AMB-1
Achromobacter piechaudii ATCC 43553
Bordetella pertussis Tohama I
Gluconacetobacter diazotrophicus PAl 5
Zymomonas mobilis subsp. mobilis ZM4
Nitrococcus mobilis Nb-231
Parachlamydia acanthamoebae str. Halls coccus
Neisseria meningitidis MC58
Escherichia coli str. K-12 substr. MG1655
Vibrio cholerae B33
Legionella pneumophila str. Paris
Pseudomonas aeruginosa PAO1
Xylella fastidiosa 9a5c
Anaplasma centrale str. Israel
Ehrlichia ruminantium str. Gardel
Kurthia sp. 538-KA26
Crocosphaera watsonii WH 8501
Cyanothece sp. ATCC 51142
99
100
100
100
7
71
100
100
57
69
43
60
100
88
23
95
100
31
99
100
0.2
Alphaproteobacteria
Bacteroidetes
Betaproteobacteria
Chlamydiae
Cyanobacteria
Deltaproteobacteria
Firmicutes
Gammaproteobacteria
bioB
bioF
bioH
bioC
bioD
bioA
