# Supplementary material for: Comparative Genomics Suggests an Independent Origin of Cytoplasmic Incompatibility in Cardinium hertigii
Source: PLoS Genet. 2012 Oct 25;8(10):e1003012. doi: 10.1371/journal.pgen.1003012 (PMC3486910; doi:10.1371/journal.pgen.1003012)
Supplement: Figure S6 — HGT-affected genes in Cardinium hertigii and its putative donors/recipients. Only HGT candidates with a bootstrap value higher than 75% and a consistent grouping in both neighbor joining and maximum likelihood trees (shown in Figure S6) were included from the list of HGT candidate genes (Table S9). (PDF) [file pgen.1003012.s006.pdf]

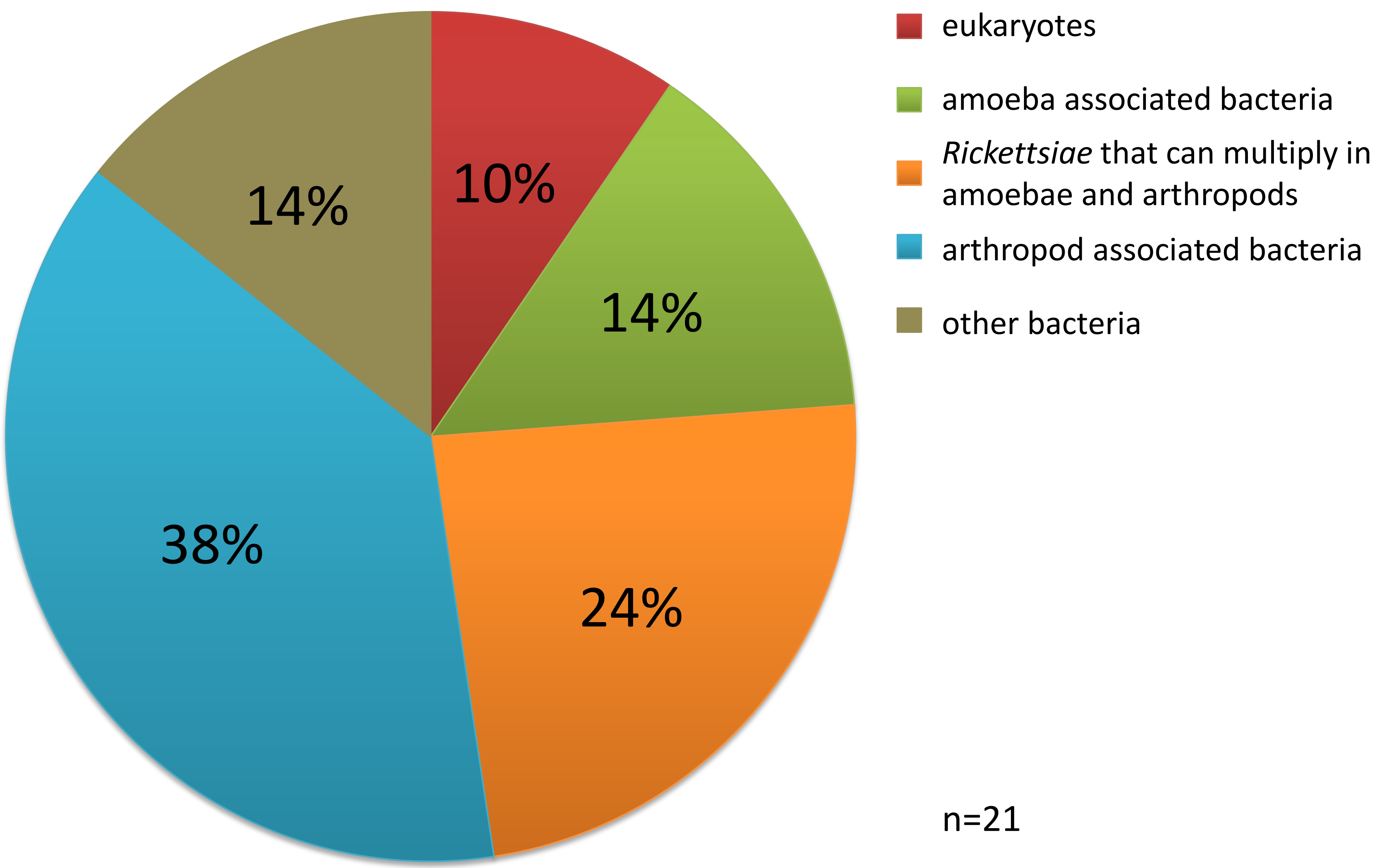
10%
14%
24%
38%
14%
eukaryotes
amoeba associated bacteria
Rickettsiae that can multiply in amoebae and arthropods
arthropod associated bacteria
other bacteria
n=21
