# Supplementary material for: Comparative Genomics Suggests an Independent Origin of Cytoplasmic Incompatibility in Cardinium hertigii
Source: PLoS Genet. 2012 Oct 25;8(10):e1003012. doi: 10.1371/journal.pgen.1003012 (PMC3486910; doi:10.1371/journal.pgen.1003012)
Supplement: Table S1 — Genome sizes of selected endosymbionts. Obligate (primary) symbionts are shaded in grey; obligate symbionts are indicated with a section sign; members of the Bacteroidetes are indicated by an asterisk; plasmids were not taken into account. (DOCX) [file pgen.1003012.s008.docx]

**Table S1.** Genome sizes of selected endosymbionts.

| **endosymbiont** | **genome size in bp** | **GC content in %** | **host** | **reference or accession number** |
| --- | --- | --- | --- | --- |
| *Candidatus* Tremblaya princeps§ | 138,927 | 58.8 | *Planococcus citri* (mealybug) | [1] |
| *Candidatus* Hodgkinia cicadicola§ | 143,795 | 58.4 | Diceroprocta semicincta (cicada) | [2] |
| *Candidatus* Carsonella ruddii§ | 159,662 | 16.5 | *Pachypsylla venusta* (psyllid) | [3] |
| *Candidatus* Zinderia insecticola CARI§ | 208,564 | 13.5 | *Clastoptera arizonana* (spittlebug) | [4] |
| Candidatus Sulcia muelleri DMIN§* | 243,933 | 22.5 | Draeculacephala minerva (sharpshooter) | [5] |
| *Candidatus* Sulcia muelleri GWSS§* | 245,530 | 22.4 | Homalodisca vitripennis (sharpshooter) | [6] |
| *Candidatus* Sulcia muelleri CARI§* | 276,511 | 21.1 | *Clastoptera arizonana* (spittlebug) | [4] |
| *Candidatus* Sulcia muelleri SMDSEM§* | 276,984 | 22.9 | Diceroprocta semicincta (cicada) | [7] |
| *Buchnera aphidicola* BCc§ | 416,380 | 20.1 | *Cinara cedri* (aphid) | [8] |
| *Buchnera aphidicola* BCt§ | 444,925 | 25.0 | *Cinara tujafilina* (aphid) | [9] |
| *Blattabacterium* sp. MADAR§* | 587,248 | 27.5 | *Mastotermes darwiniensis* (termite) | [10] |
| *Blattabacterium* sp. Cpu§* | 605,745 | 23.8 | *Cryptocercus punctulatus* (cockroach) | [11] |
| *Buchnera aphidicola* BBp§ | 615,980 | 25.3 | *Baizongia pistaciae* (aphid) | [12] |
| *Blattabacterium* sp. Bge§* | 636,850 | 27.1 | Blattella germanica (cockroach) | [13] |
| *Buchnera aphidicola* APS§ | 640,681 | 26.3 | *Acyrthosiphon pisum* (pea aphid) | [14] |
| *Buchnera aphidicola* Sg§ | 641,454 | 26.2 | *Schizaphis graminum* (greenbug) | [15] |
| *Buchnera aphidicola* Tuc7§ | 641,895 | 26.3 | *Acyrthosiphon pisum* (pea aphid) | [16] |
| *Buchnera aphidicola* 5A§ | 642,122 | 26.0 | *Acyrthosiphon pisum* (pea aphid) | [16] |
| *Baumannia cicadellinicola*§ | 686,194 | 33.3 | *Homalodisca coagulata* (sharpshooter) | [17] |
| *Wigglesworthia glossinidia*§ | 697,724 | 22.0 | *Glossina brevipalpis* (tsetse fly) | [18] |
| Candidatus *Blochmannia floridanus*§ | 705,557 | 27,4 | *Camponotus floridanus* (carpenter ant) | [19] |
| Candidatus *Blochmannia pennsylvanicus*§ | 791,654 | 29.6 | *Camponotus pennsylvanicus* (carpenter ant) | [20] |
| ***Cardinium hertigii* *c*Eper1*** | **887,130** | **36.6** | ***Encarsia pergandiella* (wasp)** | **this study** |
| *Rickettsia typhi* | 1,111,496 | 28.9 | Xenopsylla cheopis, Ctenocephalides felis (flea) and Polyplax spinulos (louse) | [21] |
| *Rickettsia prowazekii* | 1,111,523 | 29.1 | *Pediculus humanus* (flea) | [22] |
| *Candidatus* Azobacteroides pseudotrichonymphae genomovar. CFP2* | 1,114,206 | 33.0 | *Pseudotrichonympha grassii* (protist) | [23] |
| *Rickettsia canadensis* | 1,159,772 | 29.0 | *Haemaphysalis leporispalustris* and Dermacentor andersoni (tick) | [24] |
| *Rickettsia akari* | 1,231,060 | 32.3 | *Liponyssoides sanguineus* (mite) | NC_009881 |
| *Wolbachia w*Mel | 1,267,782 | 35.2 | *Drosophila melanogaster* (fruit fly) | [25] |
| *Rickettsia rickettsii* Iowa | 1,268,188 | 32.5 | Dermacentor variabilis (tick) | [26] |
| *Rickettsia conorii* | 1,268,755 | 32.4 | *Rhipicephalus sanguineus* (tick) | [27] |
| *Rickettsia slovaca* | 1,275,089 | 32.5 | *Dermacentor reticulatus, Dermacentor marginatus* and *Ixodes ricinus* (tick) | NC_016639 |
| *Rickettsia heilongjiangensis* | 1,278,471 | 32.3 | *Dysmicoccus sylvarum* (tick) | [28] |
| *Rickettsia africae* | 1,278,540 | 32.4 | Amblyomma sp. (tick) | [29] |
| *Rickettsia japonica* | 1,283,087 | 31.2 | *Haemaphysalis longicornis* (tick) | NC_016050 |
| *Rickettsia peacockii* | 1,288,492 | 32.0 | Dermacentor andersoni (tick) | [30] |
| *Rickettsia massiliae* | 1,360,898 | 32.5 | Rhipicephalus sanguineus (tick) | [31] |
| *Wolbachia w*Ri | 1,445,873 | 35.0 | *Drosophila simulans* (fruit fly) | [32] |
| *Wolbachia w*Pip | 1,482,455 | 34.2 | *Culex quinquefasciatus* (mosquito) | [33] |
| *Rickettsia felis* | 1,485,148 | 32.5 | Ctenocephalides felis, Pulex irritans (flea) | [34] |
| *Rickettsia bellii* RML369-C | 1,522,076 | 31.7 | Dermacentor sp. and Amblyomma sp. (tick) | [35] |
| *Rickettsia bellii* OSU 85-389 | 1,528,980 | 31.6 | Dermacentor sp. and Amblyomma sp. (tick) | NC_009883 |
| *Serratia symbiotica* | 1,762,765 | 29.2 | *Acyrthosiphon pisum* (pea aphid) | NC_016632 |
| *Amoebophilus asiaticus* 5a2* | 1,884,364 | 35.0 | Acanthamoeba spp. (amoeba) | [36] |
| *Orientia tsutsugamushi* Ikeda | 2,008,987 | 30.5 | Leptotrombidium pallidum and Leptotrombidium scutellare (mite) | [37] |
| *Orientia tsutsugamushi* Boryong | 2,127,051 | 30.5 | Leptotrombidium pallidum and Leptotrombidium scutellare (mite) | [38] |
| *Sodalis glossinidius* | 4,171,146 | 54.7 | *Glossina* spp. (tsetse flie) | [39] |

**References**

1. McCutcheon JP, von Dohlen CD (2011) An interdependent metabolic patchwork in the nested symbiosis of mealybugs. Curr Biol 21: 1366-1372.

2. McCutcheon JP, McDonald BR, Moran NA (2009) Origin of an alternative genetic code in the extremely small and GC-rich genome of a bacterial symbiont. PLoS Genet 5: e1000565.

3. Nakabachi A, Yamashita A, Toh H, Ishikawa H, Dunbar HE, et al. (2006) The 160-kilobase genome of the bacterial endosymbiont *Carsonella*. Science 314: 267.

4. McCutcheon JP, Moran NA (2010) Functional convergence in reduced genomes of bacterial symbionts spanning 200 My of evolution. Genome Biol Evol 2: 708-718.

5. Woyke T, Tighe D, Mavromatis K, Clum A, Copeland A, et al. (2010) One bacterial cell, one complete genome. PLoS One 5: e10314.

6. McCutcheon JP, Moran NA (2007) Parallel genomic evolution and metabolic interdependence in an ancient symbiosis. Proc Natl Acad Sci U S A 104: 19392-19397.

7. McCutcheon JP, McDonald BR, Moran NA (2009) Convergent evolution of metabolic roles in bacterial co-symbionts of insects. Proc Natl Acad Sci U S A 106: 15394-15399.

8. Perez-Brocal V, Gil R, Ramos S, Lamelas A, Postigo M, et al. (2006) A small microbial genome: the end of a long symbiotic relationship? Science 314: 312-313.

9. Lamelas A, Gosalbes MJ, Moya A, Latorre A (2011) New clues about the evolutionary history of metabolic losses in bacterial endosymbionts, provided by the genome of *Buchnera aphidicola* from the aphid *Cinara tujafilina*. Appl Environ Microbiol 77: 4446-4454.

10. Sabree ZL, Huang CY, Arakawa G, Tokuda G, Lo N, et al. (2012) Genome Shrinkage and Loss of Nutrient-Providing Potential in the Obligate Symbiont of the Primitive Termite *Mastotermes darwiniensis*. Appl Environ Microbiol 78: 204-210.

11. Neef A, Latorre A, Pereto J, Silva FJ, Pignatelli M, et al. (2011) Genome economization in the endosymbiont of the wood roach *Cryptocercus punctulatus* due to drastic loss of amino acid synthesis capabilities. Genome Biol Evol.

12. van Ham RC, Kamerbeek J, Palacios C, Rausell C, Abascal F, et al. (2003) Reductive genome evolution in *Buchnera aphidicola*. Proc Natl Acad Sci U S A 100: 581-586.

13. Lopez-Sanchez MJ, Neef A, Pereto J, Patino-Navarrete R, Pignatelli M, et al. (2009) Evolutionary convergence and nitrogen metabolism in *Blattabacterium* strain Bge, primary endosymbiont of the cockroach *Blattella germanica*. PLoS Genet 5: e1000721.

14. Shigenobu S, Watanabe H, Hattori M, Sakaki Y, Ishikawa H (2000) Genome sequence of the endocellular bacterial symbiont of aphids *Buchnera* sp. APS. Nature 407: 81-86.

15. Tamas I, Klasson L, Canback B, Naslund AK, Eriksson AS, et al. (2002) 50 million years of genomic stasis in endosymbiotic bacteria. Science 296: 2376-2379.

16. Moran NA, McLaughlin HJ, Sorek R (2009) The dynamics and time scale of ongoing genomic erosion in symbiotic bacteria. Science 323: 379-382.

17. Wu D, Daugherty SC, Van Aken SE, Pai GH, Watkins KL, et al. (2006) Metabolic complementarity and genomics of the dual bacterial symbiosis of sharpshooters. PLoS Biol 4: e188.

18. Akman L, Yamashita A, Watanabe H, Oshima K, Shiba T, et al. (2002) Genome sequence of the endocellular obligate symbiont of tsetse flies, *Wigglesworthia glossinidia*. Nat Genet 32: 402-407.

19. Gil R, Silva FJ, Zientz E, Delmotte F, Gonzalez-Candelas F, et al. (2003) The genome sequence of *Blochmannia floridanus*: comparative analysis of reduced genomes. Proc Natl Acad Sci U S A 100: 9388-9393.

20. Degnan PH, Lazarus AB, Wernegreen JJ (2005) Genome sequence of *Blochmannia pennsylvanicus* indicates parallel evolutionary trends among bacterial mutualists of insects. Genome Res 15: 1023-1033.

21. McLeod MP, Qin X, Karpathy SE, Gioia J, Highlander SK, et al. (2004) Complete genome sequence of *Rickettsia typhi* and comparison with sequences of other rickettsiae. J Bacteriol 186: 5842-5855.

22. Andersson SG, Zomorodipour A, Andersson JO, Sicheritz-Ponten T, Alsmark UC, et al. (1998) The genome sequence of *Rickettsia prowazekii* and the origin of mitochondria. Nature 396: 133-140.

23. Hongoh Y, Sharma VK, Prakash T, Noda S, Toh H, et al. (2008) Genome of an endosymbiont coupling N2 fixation to cellulolysis within protist cells in termite gut. Science 322: 1108-1109.

24. Eremeeva ME, Madan A, Shaw CD, Tang K, Dasch GA (2005) New perspectives on rickettsial evolution from new genome sequences of rickettsia, particularly *R. canadensis*, and *Orientia tsutsugamushi*. Ann N Y Acad Sci 1063: 47-63.

25. Wu M, Sun LV, Vamathevan J, Riegler M, Deboy R, et al. (2004) Phylogenomics of the reproductive parasite *Wolbachia pipientis w*Mel: a streamlined genome overrun by mobile genetic elements. PLoS Biol 2: E69.

26. Ellison DW, Clark TR, Sturdevant DE, Virtaneva K, Porcella SF, et al. (2008) Genomic comparison of virulent *Rickettsia rickettsii* Sheila Smith and avirulent *Rickettsia rickettsii* Iowa. Infect Immun 76: 542-550.

27. Ogata H, Audic S, Renesto-Audiffren P, Fournier PE, Barbe V, et al. (2001) Mechanisms of evolution in *Rickettsia conorii* and *R. prowazekii*. Science 293: 2093-2098.

28. Duan C, Tong Y, Huang Y, Wang X, Xiong X, et al. (2011) Complete genome sequence of *Rickettsia heilongjiangensis*, an emerging tick-transmitted human pathogen. J Bacteriol 193: 5564-5565.

29. Fournier PE, El Karkouri K, Leroy Q, Robert C, Giumelli B, et al. (2009) Analysis of the *Rickettsia africae* genome reveals that virulence acquisition in *Rickettsia* species may be explained by genome reduction. BMC Genomics 10: 166.

30. Felsheim RF, Kurtti TJ, Munderloh UG (2009) Genome sequence of the endosymbiont *Rickettsia peacockii* and comparison with virulent *Rickettsia rickettsii*: identification of virulence factors. PLoS One 4: e8361.

31. Blanc G, Ogata H, Robert C, Audic S, Claverie JM, et al. (2007) Lateral gene transfer between obligate intracellular bacteria: evidence from the *Rickettsia massiliae* genome. Genome Res 17: 1657-1664.

32. Klasson L, Westberg J, Sapountzis P, Naslund K, Lutnaes Y, et al. (2009) The mosaic genome structure of the *Wolbachia* *w*Ri strain infecting *Drosophila simulans*. Proc Natl Acad Sci U S A 106: 5725-5730.

33. Klasson L, Walker T, Sebaihia M, Sanders MJ, Quail MA, et al. (2008) Genome evolution of *Wolbachia* strain *w*Pip from the *Culex pipiens* group. Mol Biol Evol 25: 1877-1887.

34. Ogata H, Renesto P, Audic S, Robert C, Blanc G, et al. (2005) The genome sequence of *Rickettsia felis* identifies the first putative conjugative plasmid in an obligate intracellular parasite. PLoS Biol 3: e248.

35. Ogata H, La Scola B, Audic S, Renesto P, Blanc G, et al. (2006) Genome sequence of *Rickettsia bellii* illuminates the role of amoebae in gene exchanges between intracellular pathogens. PLoS Genet 2: e76.

36. Schmitz-Esser S, Tischler P, Arnold R, Montanaro J, Wagner M, et al. (2010) The genome of the amoeba symbiont "*Candidatus* Amoebophilus asiaticus" reveals common mechanisms for host cell interaction among amoeba-associated bacteria. J Bacteriol 192: 1045-1057.

37. Nakayama K, Yamashita A, Kurokawa K, Morimoto T, Ogawa M, et al. (2008) The Whole-genome sequencing of the obligate intracellular bacterium *Orientia tsutsugamushi* revealed massive gene amplification during reductive genome evolution. DNA Res 15: 185-199.

38. Cho NH, Kim HR, Lee JH, Kim SY, Kim J, et al. (2007) The *Orientia tsutsugamushi* genome reveals massive proliferation of conjugative type IV secretion system and host-cell interaction genes. Proc Natl Acad Sci U S A 104: 7981-7986.

39. Toh H, Weiss BL, Perkin SA, Yamashita A, Oshima K, et al. (2006) Massive genome erosion and functional adaptations provide insights into the symbiotic lifestyle of *Sodalis glossinidius* in the tsetse host. Genome Res 16: 149-156.
