# Supplementary material for: Comparative Genomics Suggests an Independent Origin of Cytoplasmic Incompatibility in Cardinium hertigii
Source: PLoS Genet. 2012 Oct 25;8(10):e1003012. doi: 10.1371/journal.pgen.1003012 (PMC3486910; doi:10.1371/journal.pgen.1003012)
Supplement: Table S2 — Transposases in the genome of Cardinium hertigii. (DOCX) [file pgen.1003012.s009.docx]

**Table S2:** Transposases in the genome of *Cardinium hertigii*.

| ***Cardinium*  locus_tag** | **best blast hit (GenBank accession no.)** | **amino acid identities to best blast hit in %** | **E-value** | **length (amino acid)** | **G+C content (%)** | **remarks** |
| --- | --- | --- | --- | --- | --- | --- |
| ***Cardinium* chromosome** |  |  |  |  |  |  |
| CAHE_0001 | transposase *Rickettsia* endosymbiont of *Ixodes scapularis* (ZP_04698353) | 78 | 6e-87 | 165 | 34 | partial |
| CAHE_0020 | transposase *Rickettsia* endosymbiont of *Ixodes scapularis* (ZP_04698353) | 82 | 2e-38 | 79 | 35 | partial |
| CAHE_0021 | transposase *Rickettsia* endosymbiont of *Ixodes scapularis* (ZP_04698353) | 68 | 4e-73 | 154 | 32 | partial |
| CAHE_0031 | transposase *Rickettsia* endosymbiont of *Ixodes scapularis* (ZP_04698353) | 72 | 9e-109 | 211 | 34 | full-length |
| CAHE_0033 | transposase *Wolinella succinogenes* DSM 1740 (NP_907532) | 56 | 4e-10 | 55 | 35 | partial |
| CAHE_0041 | transposase *Rickettsia massiliae* MTU5 (YP_001499279) | 84 | 0 | 363 | 36 | full-length |
| CAHE_0042 | transposase (fragment) *Wolbachia* endosymbiont of *Culex quinquefasciatus* Pel (YP_001975160) | 59 | 0.042 | 41 | 31 | partial |
| CAHE_0054 | transposase *Rickettsia* endosymbiont of *Ixodes scapularis* (ZP_04698353) | 73 | 3e-125 | 250 | 32 | full-length |
| CAHE_0085 | transposase ISRpe1 *Rickettsia peacockii* str. Rustic (YP_002916567) | 84 | 0 | 363 | 36 | full-length |
| CAHE_0086 | transposase mutator type *Spirochaeta* *smaragdinae* DSM 11293 (YP_003803913) | 69 | 2e-75 | 169 | 32 | partial |
| CAHE_0089 | transposase *Wolinella succinogenes* DSM 1740 (NP_907532) | 54 | 9e-148 | 406 | 31 | full-length |
| CAHE_0090 | transposase (fragment) *Wolbachia* endosymbiont of *Culex quinquefasciatus* Pel (YP_001975160) | 76 | 2e-15 | 48 | 31 | partial |
| CAHE_0093 | transposase (fragment) *Wolbachia* endosymbiont of *Culex quinquefasciatus* Pel (YP_001975185) | 70 | 2e-34 | 91 | 34 | partial |
| CAHE_0179 | transposase ISRpe1 *Rickettsia peacockii* str. Rustic (YP_002922010) | 63 | 1e-130 | 291 | 32 | full-length |
| CAHE_0204 | integrase *Rickettsia felis* (ADD74146) | 56 | 1e-05 | 54 | 25 | partial |
| CAHE_0264 | transposase *Rickettsia* endosymbiont of *Ixodes scapularis* (ZP_04698353) | 56 | 1e-05 | 55 | 34 | partial |
| CAHE_0265 | transposase *Rickettsia* endosymbiont of *Ixodes scapularis* (ZP_04698353) | 71 | 1e-84 | 174 | 33 | partial |
| CAHE_0284 | transposase *Rickettsia* endosymbiont of *Ixodes scapularis* (ZP_04698353) | 76 | 4e-37 | 88 | 35 | partial |
| CAHE_0285 | transposase *Rickettsia* endosymbiont of *Ixodes scapularis* (ZP_04698353) | 72 | 3e-123 | 236 | 32 | full-length |
| CAHE_0306 | transposase IS4 family protein *Burkholderia phymatum* STM815 (YP_001863424) | 47 | 2e-86 | 289 | 32 | partial |
| CAHE_0343 | IS256 family transposase, partial *Aeromonas salmonicida* subsp. *salmonicida* 01-B526 (EHI50055) | 63 | 4e-68 | 171 | 38 | partial |
| CAHE_0344 | IS256 family transposase *Marinobacter manganoxydans* MnI7-9 (ZP_09159722) | 78 | 2e-21 | 68 | 34 | partial |
| CAHE_0345 | transposase mutator type *Spirochaeta smaragdinae* DSM 11293 (YP_003803913) | 67 | 3e-55 | 131 | 33 | partial |
| CAHE_0370 | transposase *Wolinella succinogenes* DSM 1740 (NP_907532) | 55 | 7e-149 | 406 | 31 | full-length |
| CAHE_0447 | hypothetical protein HMPREF0666_00098 *Prevotella* sp. C561 (ZP_08833922) | 42 | 3e-11 | 73 | 35 | partial |
| CAHE_0471 | chromosome segregation ATPase *Rickettsia monacensis* (YP_001967401) | 70 | 5e-144 | 292 | 33 | full-length |
| CAHE_0500 | transposase *Rickettsia massiliae* MTU5 (YP_001499279) | 81 | 0 | 363 | 35 | full-length |
| CAHE_0515 | ISPg1, transposase *Prevotella buccalis* ATCC 35310 (ZP_06285825) | 68 | 1e-18 | 58 | 37 | partial |
| CAHE_0516 | ISRSO18-transposase protein *Methylibium petroleiphilum* PM1 (YP_001023604) | 43 | 6e-04 | 73 | 37 | partial |
| CAHE_0517 | transposase, IS4 family *Prevotella buccalis* ATCC 35310 (ZP_06286857) | 51 | 4e-49 | 179 | 36 | partial |
| CAHE_0526 | hypothetical protein Aasi_0552 *Amoebophilus asiaticus* 5a2 (YP_001957682) | 67 | 4e-22 | 65 | 32 | partial |
| CAHE_0527 | transposase *Cardinium* endosymbiont of *Ixodes scapularis* (ACZ36949) | 89 | 8e-142 | 219 | 37 | partial |
| CAHE_0528 | transposase *Rickettsia peacockii* (AAY56396) | 77 | 1e-35 | 76 | 36 | partial |
| CAHE_0533 | transposase (fragment) *Wolbachia* endosymbiont of *Culex quinquefasciatus* Pel (YP_001975160) | 72 | 4e-42 | 99 | 30 | partial |
| CAHE_0534 | transposase *Rickettsia* endosymbiont of *Ixodes scapularis* (ZP_04698353) | 82 | 2e-32 | 67 | 38 | partial |
| CAHE_0539 | transposase *Piscirickettsia salmonis* (AAG16999) | 46 | 1e-48 | 181 | 27 | partial |
| CAHE_0540 | putative transposase *Yersinia pseudotuberculosis* (CAF28495) | 49 | 8e-11 | 73 | 35 | partial |
| CAHE_0541 | transposase *Wolinella succinogenes* DSM 1740 (NP_907532) | 55 | 1e-149 | 406 | 31 | full-length |
| CAHE_0542 | hypothetical protein Aasi_0894 *Amoebophilus asiaticus* 5a2 (YP_001957990) | 42 | 2e-39 | 252 | 31 | partial |
| CAHE_0547 | transposase *Wolinella succinogenes* DSM 1740 (NP_907532) | 55 | 1e-149 | 406 | 31 | full-length |
| CAHE_0548 | transposase *Rickettsia rhipicephali* str. 3-7-female6-CWPP (AFC72473) | 66 | 7e-74 | 183 | 34 | partial |
| CAHE_0549 | transposase *Rickettsia felis* (ADD74152) | 75 | 3e-129 | 135 | 33 | partial |
| CAHE_0551 | transposase *Rickettsia felis* (ADD74152) | 72 | 0 | 357 | 34 | partial |
| CAHE_0553 | chromosome segregation ATPase *Rickettsia monacensis* (YP_001967401) | 68 | 7e-139 | 304 | 33 | full-length |
| CAHE_0554 | hypothetical protein LDG_5520 *Legionella drancourtii* LLAP12 (EHL32375) | 77 | 5e-100 | 201 | 38 | full-length |
| CAHE_0577 | transposase (fragment) *Wolbachia* endosymbiont of *Culex quinquefasciatus* Pel (YP_001975160) | 73 | 8e-10 | 49 | 30 | partial |
| CAHE_0592 | transposase *Rickettsia* endosymbiont of *Ixodes scapularis* (ZP_04698353) | 87 | 6e-26 | 55 | 33 | partial |
| CAHE_0593 | transposase *Rickettsia* endosymbiont of *Ixodes scapularis* (ZP_04698353) | 70 | 1e-72 | 154 | 31 | partial |
| CAHE_0594 | chromosome segregation ATPase *Rickettsia monacensis* (YP_001967401) | 64 | 3e-135 | 312 | 34 | full-length |
| CAHE_0595 | transposase *Rickettsia* endosymbiont of *Ixodes scapularis* (ZP_04698353) | 89 | 8e-27 | 55 | 32 | partial |
| CAHE_0597 | transposase *Rickettsia* endosymbiont of *Ixodes scapularis* (ZP_04698353) | 70 | 2e-73 | 135 | 31 | partial |
| CAHE_0598 | transposase *Rickettsia* endosymbiont of *Ixodes scapularis* (ZP_04698353) | 87 | 6e-26 | 55 | 33 | partial |
| CAHE_0600 | ISPg1, transposase *Prevotella buccalis* ATCC 35310 (ZP_06285825) | 72 | 1e-16 | 92 | 33 | partial |
| CAHE_0601 | transposase IS4 family *Deferribacter desulfuricans* SSM1 (YP_003495544) | 53 | 2e-04 | 73 | 39 | partial |
| CAHE_0602 | hypothetical protein HMPREF0666_00098 *Prevotella* sp. C561 (ZP_08833922) | 42 | 3e-11 | 73 | 35 | partial |
| CAHE_0609 | hypothetical protein RPR_p14 *Rickettsia peacockii* str. Rustic (YP_002922010) | 65 | 1e-13 | 66 | 27 | partial |
| CAHE_0610 | hypothetical protein HMPREF0666_00098 *Prevotella* sp. C561 (ZP_08833922) | 42 | 3e-11 | 73 | 35 | partial |
| CAHE_0611 | transposase IS4 family *Deferribacter desulfuricans* SSM1 (YP_003495544) | 53 | 2e-04 | 73 | 39 | partial |
| CAHE_0612 | ISPg1, transposase *Prevotella buccalis* ATCC 35310 (ZP_06285825) | 72 | 1e-16 | 91 | 33 | partial |
| CAHE_0616 | transposase *Rickettsia* endosymbiont of *Ixodes scapularis* (ZP_04698516) | 77 | 1e-134 | 249 | 33 | partial |
| CAHE_0627 | transposase *Rickettsia* endosymbiont of *Ixodes scapularis* (ZP_04698353) | 75 | 4e-129 | 236 | 32 | full-length |
| CAHE_0629 | transposase *Rickettsia felis* (ADD74152) | 76 | 1e-152 | 273 | 33 | partial |
| CAHE_0630 | transposase *Rickettsia rhipicephali* str. 3-7-female6-CWPP (AFC72473) | 59 | 4e-35 | 130 | 36 | partial |
| CAHE_0631 | transposase *Rickettsia massiliae* MTU5 (YP_001499279) | 81 | 0 | 363 | 35 | full-length |
| CAHE_0682 | transposase *Amoebophilus asiaticus* (ADL32106) | 78 | 8e-27 | 84 | 33 | partial |
| CAHE_0683 | unnamed protein product *Rhizobium leguminosarum* (CAA58901) | 61 | 2e-45 | 128 | 33 | partial |
| CAHE_0684 | hypothetical protein Aasi_1273 *Amoebophilus asiaticus* 5a2 8YP_001958323) | 66 | 3e-09 | 58 | 29 | partial |
| CAHE_0686 | transposase *Wolinella succinogenes* DSM 1740 (NP_907532) | 53 | 2e-132 | 365 | 31 | partial |
| CAHE_0687 | unnamed protein product *Rickettsia felis* URRWXCal2 (YP_246337) | 75 | 0.078 | 50 | 38 | partial |
| CAHE_0688 | hypothetical protein OTT_0880 *Orientia tsutsugamushi* str. Ikeda (YP_001937572) | 53 | 4e-128 | 382 | 36 | full-length |
| CAHE_0689 | ISPg1, transposase *Prevotella buccalis* ATCC 35310 (ZP_06285825) | 66 | 8e-19 | 58 | 39 | partial |
| CAHE_0707 | unnamed protein product *Rickettsia bellii* OSU 85-389 (YP_001496109) | 71 | 1e-37 | 92 | 30 | partial |
| CAHE_0708 | transposase *Rickettsia parkeri* str. Portsmouth (AFC74898) | 73 | 2e-174 | 345 | 35 | partial |
| CAHE_0737 | transposase mutator type Spirochaeta smaragdinae DSM 11293 (YP_003803913) | 67 | 3e-55 | 131 | 33 | partial |
| CAHE_0738 | IS110 family transposase *Bacteroides fragilis* YCH46 (YP_099055) | 49 | 2e-102 | 325 | 36 | full-length |
| CAHE_0739 | IS256 family transposase, partial *Aeromonas salmonicida* subsp. *salmonicida* 01-B526 (EHI50055) | 63 | 4e-68 | 171 | 38 | partial |
| CAHE_0787 | transposase *Rickettsia* endosymbiont of *Ixodes scapularis* (ZP_04698353) | 74 | 6e-109 | 209 | 32 | partial |
| CAHE_0808 | putative transposase *Rickettsia* endosymbiont of *Ixodes scapularis* (ZP_04700133) | 61 | 3e-44 | 210 | 34 | full-length |
| CAHE_0809 | putative transposase *Rickettsia* endosymbiont of *Ixodes scapularis* (ZP_04700133) | 61 | 6e-45 | 148 | 32 | full-length |
| CAHE_0810 | transposase *Wolinella succinogenes* DSM 1740 (NP_907532) | 55 | 7e-149 | 406 | 31 | full-length |
| CAHE_0811 | transposase *Wolinella succinogenes* DSM 1740 (NP_907532) | 59 | 2e-82 | 221 | 30 | partial |
| CAHE_0812 | putative transposase *Wolbachia pipientis* wAlbB (ZP_09542015) | 49 | 4e-16 | 86 | 35 | partial |
| CAHE_0813 | IS4 family transposase *Wolbachia* endosymbiont of *Drosophila melanogaster* (NP_965935) | 63 | 2e-152 | 347 | 30 | partial |
| CAHE_0816 | transposase *Rickettsia felis* (ADD74152) | 71 | 0 | 413 | 33 | full-length |
| CAHE_0818 | resolvase domain-containing protein *Acidithiobacillus ferrivorans* SS3 (YP_004783228) | 65 | 1e-82 | 204 | 32 | full-length |
| CAHE_0819 | putative transposase *Legionella drancourtii* LLAP12 (ZP_09620192) | 47 | 3e-69 | 249 | 33 | partial |
| CAHE_0820 | putative transposase *Legionella drancourtii* LLAP12 (ZP_09620192) | 42 | 1e-93 | 383 | 34 | partial |
| CAHE_0821 | hypothetical protein LDG_7507 *Legionella drancourtii* LLAP12 (ZP_09621089) | 53 | 2e-13 | 59 | 35 | partial |
| CAHE_0822 | putative transposase *Legionella drancourtii* LLAP12 (ZP_09620192) | 56 | 9e-77 | 238 | 34 | partial |
| CAHE_0823 | IS110 family transposase *Bacteroides fragilis* YCH46 (YP_099055) | 49 | 2e-102 | 325 | 36 | full-length |
| CAHE_0824 | resolvase domain protein *Klebsiella pneumoniae* 342 | 76 | 2e-116 | 220 | 35 | full-length |
| CAHE_0825 | transposase for transposon *Escherichia coli* M605 (ZP_08348011) | 76 | 9e-17 | 52 | 40 | partial |
| CAHE_0826 | hypothetical protein pVM01_p078 *Escherichia coli* (YP_001711927) | 76 | 0 | 627 | 37 | partial |
| CAHE_0827 | IS110 family transposase *Bacteroides fragilis* YCH46 (YP_099055) | 49 | 2e-102 | 325 | 36 | full-length |
| CAHE_0828 | hypothetical protein LDG_5520 *Legionella drancourtii* LLAP12 (ZP_09619179.1) | 71 | 2e-32 | 81 | 36 | partial |
| CAHE_0830 | chromosome segregation ATPase *Rickettsia monacensis* (YP_001967401) | 64 | 5e-136 | 316 | 33 | full-length |
| CAHE_0831 | transposase *Rickettsia* endosymbiont of *Ixodes scapularis* (ZP_04698353) | 75 | 5e-126 | 236 | 32 | full-length |
| CAHE_0833 | transposase *Wolinella succinogenes* DSM 1740 (NP_907532) | 55 | 4e-149 | 406 | 31 | full-length |
| CAHE_0840 | S256 family transposase, partial *Aeromonas salmonicida* subsp. *salmonicida* 01-B526 (EHI50055) | 63 | 4e-68 | 171 | 38 | partial |
| CAHE_0841 | IS256 family transposase *Marinobacter* sp. MnI7-9 | 78 | 2e-21 | 68 | 34 | partial |
| CAHE_0842 | transposase mutator type *Spirochaeta* *smaragdinae* DSM 11293 (YP_003803913) | 67 | 3e-55 | 131 | 33 | partial |
| CAHE_0843 | transposase *Rickettsia massiliae* MTU5 (YP_001499279) | 76 | 2e-20 | 77 | 29 | partial |
| CAHE_0844 | hypothetical protein LDG_7509 *Legionella drancourtii* LLAP12 (ZP_09620471) | 68 | 5e-08 | 48 | 27 | partial |
| CAHE_0845 | transposase *Rickettsia felis* (ADD74152) | 75 | 3e-156 | 285 | 33 | partial |
| ***Cardinium* plasmid** |  |  |  |  |  |  |
| CAHE_p0001 | IS110 family transposase *Bacteroides fragilis* YCH46 (YP_099055) | 49 | 2e-102 | 325 | 36 | full-length |
| CAHE_p0002 | transposase *Rickettsia* endosymbiont of *Ixodes scapularis* (ZP_04698354) | 64 | 1e-140 | 315 | 35 | full-length |
| CAHE_p0003 | transposase *Cardinium* endosymbiont of *Ixodes scapularis* (ACZ36949) | 78 | 9e-81 | 153 | 36 | partial |
| CAHE_p0004 | integrase family protein *Candidatus* Odyssella thessalonicensis L13 (ZP_08778839) | 79 | 1e-96 | 171 | 39 | full-length |
| CAHE_p0005 | chromosome segregation ATPase *Rickettsia monacensis* (YP_001967401) | 72 | 3e-133 | 328 | 35 | partial |
| CAHE_p0006 | transposase *Rickettsia* endosymbiont of *Ixodes scapularis* (ZP_04698353) | 75 | 4e-128 | 236 | 33 | full-length |
| CAHE_p0010 | IS110 family transposase *Bacteroides fragilis* YCH46 (YP_099055) | 49 | 4e-37 | 142 | 36 | partial |
| CAHE_p0011 | unnamed protein product *Bacteroides salanitronis* DSM 18170 (YP_004258626) | 49 | 4e-48 | 171 | 36 | partial |
| CAHE_p0012 | hypothetical protein HMPREF9689_05537 *Klebsiella oxytoca* 10-5245 (EHS88024.1) | 76 | 2e-116 | 220 | 35 | full-length |
| CAHE_p0013 | hypothetical protein plu1643 *Photorhabdus luminescens* subsp. laumondii TTO1 (NP_928931) | 36 | 8e-59 | 332 | 33 | full-length |
| CAHE_p0032 | transposase *Treponema vincentii* ATCC 35580 (ZP_05622685) | 39 | 0.019 | 91 | 27 | partial |
| CAHE_p0039 | chromosome segregation ATPase *Rickettsia monacensis* (YP_001967401) | 62 | 3e-132 | 323 | 35 | full-length |
| CAHE_p0040 | IS256 family transposase, partial *Aeromonas salmonicida* subsp. salmonicida 01-B526 (EHI50055) | 65 | 8e-33 | 123 | 37 | partial |
| CAHE_p0041 | transposase *Rickettsia felis* URRWXCal2 (YP_247470) | 73 | 9e-116 | 220 | 33 | full-length |
| CAHE_p0042 | transposase *Rickettsia felis* (ADD74152) | 65 | 2e-61 | 159 | 36 | partial |
| CAHE_p0046 | transposase *Rickettsia* endosymbiont of *Ixodes scapularis* (ZP_04698353) | 74 | 1e-123 | 236 | 33 | full-length |
| CAHE_p0047 | IS110 family transposase *Bacteroides fragilis* YCH46 (YP_099055) | 50 | 4e-103 | 325 | 36 | full-length |
| CAHE_p0056 | transposase *Wolinella succinogenes* DSM 1740 (NP_907532) | 61 | 1e-74 | 226 | 30 | partial |
| CAHE_p0057 | putative transposase *Wolinella succinogenes* (CAA05034) | 51 | 2e-47 | 188 | 32 | partial |
| CAHE_p0058 | transposase (fragment) *Wolbachia* endosymbiont of *Culex quinquefasciatus* Pel (YP_001975160) | 74 | 2e-43 | 99 | 33 | partial |
| CAHE_p0061 | transposase (fragment) *Wolbachia* endosymbiont of *Culex quinquefasciatus* Pel (YP_001975160) | 74 | 5e-15 | 48 | 31 | partial |
| CAHE_p0062 | chromosome segregation ATPase *Rickettsia monacensis* (YP_001967401) | 65 | 9e-138 | 312 | 34 | full-length |
| CAHE_p0063 | transposase (fragment) *Wolbachia* endosymbiont of *Culex quinquefasciatus* Pel (YP_001975160) | 73 | 1e-45 | 114 | 32 | partial |
| CAHE_p0064 | transposase *Wolinella succinogenes* DSM 1740 (NP_907532) | 55 | 5e-149 | 406 | 31 | full-length |
| CAHE_p0065 | transposase *Rickettsia felis* (ADD74152) | 76 | 1e-160 | 285 | 33 | partial |
