# Supplementary material for: Comparative Genomics Suggests an Independent Origin of Cytoplasmic Incompatibility in Cardinium hertigii
Source: PLoS Genet. 2012 Oct 25;8(10):e1003012. doi: 10.1371/journal.pgen.1003012 (PMC3486910; doi:10.1371/journal.pgen.1003012)
Supplement: Table S3 — Cardinium hertigii proteins involved in biotin biosynthesis, glycolysis, peptidoglycan biosynthesis, and lipoate biosynthesis. (DOCX) [file pgen.1003012.s010.docx]

**Table S3:** *Cardinium hertigii* proteins involved in biotin biosynthesis, glycolysis, peptidoglycan biosynthesis, and lipoate biosynthesis.

| ***Cardinium* locus_tag** | **best blast hit (GenBank accession no.)** | **amino acid identities to best blast hit in %** | **E-value** |  | **length (amino acid)** | **GC content in %** | **putative function** |
| --- | --- | --- | --- | --- | --- | --- | --- |
| **biotin biosynthesis** |  |  |  |  |  |  |  |
| CAHE_0562 | ubiE/COQ5 methyltransferase family protein *Lawsonia intracellularis* PHE/MN1-00 (YP_594800) | 42 | 1e-61 |  | 246 | 34 | malonyl-CoA methyltransferase (bioC) |
| CAHE_0560 | serine hydroxymethyltransferase *Rickettsia* endosymbiont of *Ixodes scapularis* (ZP_04698252) | 62 | 8e-169 |  | 379 | 35 | 8-amino-7-oxononanoate synthase (bioF) |
| CAHE_0564 | adenosylmethionine-8-amino-7-oxononanoate transaminase *Rickettsia* endosymbiont of *Ixodes scapularis* (ZP_04698248) | 62 | 0 |  | 417 | 39 | 7,8-diaminopelargonic acid synthase (bioA) |
| CAHE_0563 | dethiobiotin synthase *Rickettsia* endosymbiont of *Ixodes scapularis* (ZP_04698249) | 59 | 4e-82 |  | 206 | 37 | dethiobiotin synthetase (bioD) |
| CAHE_0559 | biotin synthase *Rickettsia* endosymbiont of *Ixodes scapularis* (ZP_04698253) | 73 | 4e-173 |  | 321 | 38 | biotin synthase (bioB) |
| CAHE_0561 | conserved hypothetical protein *Rickettsia* endosymbiont of *Ixodes scapularis* (ZP_04698251) | 43 | 4e-42 |  | 221 | 33 | carboxylesterase (bioH) |
| **glycolysis** |  |  |  |  |  |  |  |
| CAHE_0062 | triosephosphate isomerase *Flavobacteriaceae bacterium* S85 (ZP_09496967) | 44 | 2e-67 |  | 259 | 40 | triosephosphate isomerase |
| CAHE_0506 | hypothetical protein Aasi_1069 *Amoebophilus asiaticus* 5a2 (YP_001958143) | 63 | 2e-148 |  | 329 | 39 | glyceraldehyde-3-phosphate dehydrogenase |
| CAHE_0097 | phosphoglycerate kinase *Gramella forsetii* KT0803 | 43 | 3e-115 |  | 397 | 38 | phosphoglycerate kinase |
| CAHE_0692 | 2,3-bisphosphoglycerate-independent phosphoglycerate mutase *Rickettsiella grylli* (ZP_02062305) | 58 | 0 |  | 518 | 40 | phosphoglycerate mutase |
| CAHE_0835 | phosphopyruvate hydratase *Rickettsiella grylli* (ZP_02061895) | 64 | 0 |  | 435 | 38 | enolase |
| **peptidoglycan biosynthesis** | |  |  |  |  |  |  |
| CAHE_0022 | UDP-N-acetylglucosamine 1-carboxyvinyltransferase *Prevotella pallens* ATCC 700821 (ZP_08674836) | 57 | 4e-168 |  | 436 | 43 | UDP-N-acetylglucosamine 1-carboxyvinyltransferase (murA) |
| CAHE_0497 | UDP-N-acetylenolpyruvoylglucosamine reductase  *Spirosoma linguale* DSM 74 (YP_003389926) | 44 | 3e-103 |  | 346 | 35 | UDP-N-acetylenolpyruvoylglucosamine reductase (murB) |
| CAHE_0642 | UDP-N-acetylmuramate--L-alanine ligase *Runella slithyformis* DSM 19594 (YP_004655336) | 43 | 6e-123 |  | 471 | 39 | UDP-N-acetylmuramate--L-alanine ligase (murC) |
| CAHE_0736 | hypothetical protein Aasi_0816 *Amoebophilus asiaticus* 5a2 (YP_001957919) | 54 | 9e-155 |  | 448 | 39 | UDP-N-acetylmuramoylalanine--D-glutamate ligase (murD) |
| CAHE_0351 | UDP-N-acetylmuramoylalanyl-D-glutamate--2,6-diaminopimelate ligase *Amoebophilus asiaticus* 5a2 (YP_001957724) | 53 | 3e-180 |  | 491 | 40 | UDP-N-acetylmuramoylalanyl-D-glutamate--2,6-diaminopimelate ligase (murE) |
| CAHE_0786 | hypothetical protein Aasi_1231 *Amoebophilus asiaticus* 5a2 (YP_00195828) | 45 | 2e-125 |  | 437 | 42 | UDP-N-acetylmuramoylalanyl-D-glutamyl-2,6-diaminopimelate/D-alanyl-D-alanyl  ligase (murF) |
| CAHE_0361 | phospho-N-acetylmuramoyl-pentapeptide-transferase  *Amoebophilus asiaticus* 5a2 (YP_001957428) | 63 | 9e-176 |  | 430 | 36 | phospho-N-acetylmuramoyl-pentapeptide-transferase (mraY) |
| CAHE_0165 | udp-N-acetylglucosamine--N-acetylmuramyL-(pentapeptide) pyrophosphoryL-undecaprenol  N-acetylglucosamine transferase *Paludibacter*  *propionicigenes* WB4 (YP_004042630) | 45 | 3e-101 |  | 369 | 38 | UDP-N-acetylglucosamine--N-acetylmuramyL-(pentapeptide) pyrophosphoryL-undecaprenol  N-acetylglucosamine transferase (murG) |
| CAHE_0508 | hypothetical protein Aasi_0981 *Amoebophilus asiaticus* 5a2 (YP_001958066) | 58 | 0 |  | 766 | 38 | peptidoglycan glycosyltransferase (pbpB) |
| CAHE_0350 | hypothetical protein Aasi_0595 *Amoebophilus asiaticus* 5a2 (YP_001957725) | 49 | 0 |  | 726 | 39 | peptidoglycan glycosyltransferase |
| **lipoate biosynthesis** |  |  |  |  |  |  |  |
| CAHE_0742 | lipoyl synthase *Cytophaga hutchinsonii* ATCC 33406 (YP_679772) | 77 | 2e-160 |  | 290 | 44 | lipoyl synthase (lipA) |
| CAHE_0318 | lipoyltransferase *Cytophaga hutchinsonii* ATCC 33406 (YP_677094) | 50 | 5e-73 |  | 236 | 37 | lipoate-protein ligase B (lipB) |
