# Supplementary material for: Comparative Genomics Suggests an Independent Origin of Cytoplasmic Incompatibility in Cardinium hertigii
Source: PLoS Genet. 2012 Oct 25;8(10):e1003012. doi: 10.1371/journal.pgen.1003012 (PMC3486910; doi:10.1371/journal.pgen.1003012)
Supplement: Table S4 — Transport proteins in the genome of Cardinium hertigii. (DOCX) [file pgen.1003012.s011.docx]

**Table S4:** Transport proteins in the genome of *Cardinium hertigii.*

| ***Cardinium* locus_tag** | **best blast hit (GenBank accession no.)** | **amino acid identities to best blast hit in %** | **E-value** | **length (amino acid)** | **Transport Classification Database**  **(TCDB) Family** | **number of transmembrane helices (HMMTOP)** |
| --- | --- | --- | --- | --- | --- | --- |
| CAHE_0018 | ADP,ATP carrier protein 1 *Simkania negevensis* Z (YP_004671674) | 48 | 3e-145 | 475 | 2.A.12  The ATP:ADP Antiporter (AAA) Family | 11 |
| CAHE_0039 | hypothetical protein Aasi_0939 *Amoebophilus asiaticus* 5a2 (YP_001958029) | 28 | 1e-41 | 482 | 2.A.21  The Solute:Sodium Symporter (SSS) Family | 13 |
| CAHE_0109 | hypothetical protein Aasi_1859 *Amoebophilus asiaticus* 5a2 (YP_003573226.1) | 47 | 6e-81 | 284 | 2.A.7  **The Drug/Metabolite Transporter (DMT) Superfamily** | 10 |
| CAHE_0117 | hypothetical protein Aasi_0563 *Amoebophilus asiaticus* 5a2 (YP_001957693.1) | 23 | 7e-11 | 691 | 1.A.3  The Ryanodine-Inositol 1,4,5-triphosphate Receptor Ca^2+^ Channel (RIR-CaC) Family | 6 |
| CAHE_0158 | ADP/ATP carrier protein *Amoebophilus asiaticus* 5a2 (YP_001957248) | 51 | 4e-154 | 500 | 2.A.12  The ATP:ADP Antiporter (AAA) Family | 12 |
| CAHE_0160 | ADP/ATP carrier protein *Amoebophilus asiaticus* 5a2 (YP_001957248) | 52 | 2e-160 | 500 | 2.A.12  The ATP:ADP Antiporter (AAA) Family | 13 |
| CAHE_0163 | hypothetical protein Aasi_0627 *Amoebophilus asiaticus* 5a2 (YP_001957754) | 57 | 2e-105 | 282 | 3.A.1  **The ATP-binding Cassette (ABC) Superfamily** | 9 |
| CAHE_0170 | hypothetical protein Aasi_0507 *Amoebophilus asiaticus* 5a2 (YP_001957641) | 52 | 1e-153 | 475 | 9.A.19  **The Mg^2+^ Transporter-E (MgtE) Family** | 6 |
| CAHE_0186 | hypothetical protein Aasi_0053 *Amoebophilus asiaticus* 5a2 (YP_001957235) | 54 | 8e-28 | 222 | 2.A.7  **The Drug/Metabolite Transporter (DMT) Superfamily** | 1 |
| CAHE_0187 | putative tansport related protein *Microscilla marina* ATCC 23134 (ZP_01687105) | 41 | 6e-25 | 135 | no hit | 1 |
| CAHE_0188 | MotA/TolQ/ExbB proton channel family protein *Algoriphagus* sp. PR1 (ZP_07721030) | 44 | 9e-49 | 205 | 1.A.30  **The H^+^- or Na^+^-translocating Bacterial Flagellar Motor/ExbBD Outer Membrane Transport Energizer (Mot-Exb) Superfamily** | 3 |
| CAHE_0195 | ABC transporter *Bacteroides fragilis* 3_1_12 (ZP_07807413) | 28 | 2e-49 | 403 | 3.A.1  **The ATP-binding Cassette (ABC) Superfamily** | 4 |
| CAHE_0196 | ABC transporter *Bacteroides fragilis* 3_1_12 (ZP_07807413) | 34 | 2e-65 | 402 | 3.A.1  **The ATP-binding Cassette (ABC) Superfamily** | 4 |
| CAHE_0197 | methyl-accepting chemotaxis sensory transducer *Anaerophaga thermohalophila* DSM 12881 (ZP_08845516.1) | 35 | 2e-60 | 365 | 3.A.1  **The ATP-binding Cassette (ABC) Superfamily** | 1 |
| CAHE_0198 | phosphonate-transporting ATPase *Muricauda ruestringensis* DSM 13258 (YP_004788342) | 58 | 7e-87 | 220 | 3.A.1  **The ATP-binding Cassette (ABC) Superfamily** | 1 |
| CAHE_0199 | outer membrane efflux protein *Pedobacter heparinus* DSM 2366 (YP_003093990) | 24 | 5e-13 | 435 | 2.A.6  **The Resistance-Nodulation-Cell Division (RND) Superfamily** | 1 |
| CAHE_0200 | permease YjgP/YjgQ family protein *Amoebophilus asiaticus* 5a2 (YP_001957213) | 44 | 7e-98 | 361 | 1.B.42  **The Outer Membrane Lipopolysaccharide Export Porin (LPS-EP) Family** | 6 |
| CAHE_0202 | ABC transporter, ATP-binding protein *Algoriphagus* sp. PR1 (ZP_07719230) | 76 | 1e-131 | 244 | 3.A.1  **The ATP-binding Cassette (ABC) Superfamily** | 1 |
| CAHE_0210 | hypothetical protein Aasi_1031 *Amoebophilus asiaticus* 5a2 (YP_001958108) | 51 | 2e-89 | 289 | 3.A.1  **The ATP-binding Cassette (ABC) Superfamily** | 7 |
| CAHE_0219 | hypothetical protein Aasi_0641 *Amoebophilus asiaticus* 5a2 (YP_001957767) | 29 | 2e-48 | 522 | 1.A.23  **The Small Conductance Mechanosensitive Ion Channel (MscS) Family** | 4 |
| CAHE_0228 | hypothetical protein Aasi_0939 *Amoebophilus asiaticus* 5a2 (YP_001958029) | 28 | 1e-109 | 1118 | 2.A.21  The Solute:Sodium Symporter (SSS) Family | 20 |
| CAHE_0236 | hypothetical protein Aasi_0694 *Amoebophilus asiaticus* 5a2 (YP_001957813) | 27 | 3e-41 | 480 | 2.A.21  The Solute:Sodium Symporter (SSS) Family | 13 |
| CAHE_0240 | hypothetical protein Aasi_0202 *Amoebophilus asiaticus* 5a2 (YP_001957376) | 64 | 1e-152 | 329 | 3.A.1  **The ATP-binding Cassette (ABC) Superfamily** | 1 |
| CAHE_0241 | hypothetical protein Aasi_0203 *Amoebophilus asiaticus* 5a2 (YP_001957377) | 76 | 1e-131 | 331 | 3.A.1  **The ATP-binding Cassette (ABC) Superfamily** | 1 |
| CAHE_0242 | hypothetical protein Aasi_0204 *Amoebophilus asiaticus* 5a2 (YP_001957378) | 56 | 3e-106 | 287 | 3.A.1  **The ATP-binding Cassette (ABC) Superfamily** | 5 |
| CAHE_0244 | hypothetical protein Aasi_0205 *Amoebophilus asiaticus* 5a2 (YP_001957379) | 54 | 1e-113 | 314 | 3.A.1  **The ATP-binding Cassette (ABC) Superfamily** | 6 |
| CAHE_0245 | hypothetical protein Aasi_1498 *Amoebophilus asiaticus* 5a2 (YP_003572991) | 44 | 2e-158 | 582 | 3.A.1  **The ATP-binding Cassette (ABC) Superfamily** | 1 |
| CAHE_0352 | hypothetical protein Aasi_0939 *Amoebophilus asiaticus* 5a2 (YP_001958029) | 26 | 1e-92 | 1180 | 2.A.21  The Solute:Sodium Symporter (SSS) Family | 18 |
| CAHE_0353 | hypothetical protein Aasi_0939 *Amoebophilus asiaticus* 5a2 (YP_001958029) | 27 | 1e-90 | 1149 | 2.A.21  The Solute:Sodium Symporter (SSS) Family | 17 |
| CAHE_0373 | permease YjgP/YjgQ family protein *Amoebophilus asiaticus* 5a2 (YP_001957197) | 35 | 3e-89 | 504 | 2.A.72  **The K^+^ Uptake Permease (KUP) Family** | 6 |
| CAHE_0386 | pnuC gene product *Flavobacterium branchiophilum* FL-15 (YP_004842679) | 25 | 4e-09 | 255 | 2.A.12  The ATP:ADP Antiporter (AAA) Family | 7 |
| CAHE_0495 | hypothetical protein Aasi_0694 *Amoebophilus asiaticus* 5a2 (YP_001957813) | 30 | 4e-56 | 501 | 2.A.21  The Solute:Sodium Symporter (SSS) Family | 13 |
| CAHE_0545 | hypothetical protein Aasi_1444 *Amoebophilus asiaticus* 5a2 (YP_001958462) | 33 | 2e-179 | 966 | 2.A.21  The Solute:Sodium Symporter (SSS) Family | 20 |
| CAHE_0546 | Na-solute symporter *Amoebophilus asiaticus* 5a2 (YP_003573149) | 49 | 2e-40 | 117 | no hit | 1 |
| CAHE_0555 | hypothetical protein Aasi_0939 *Amoebophilus asiaticus* 5a2 (YP_001958029) | 33 | 1e-48 | 388 | 2.A.21  The Solute:Sodium Symporter (SSS) Family | 9 |
| CAHE_0580 | hypothetical protein Aasi_1689 *Amoebophilus asiaticus* 5a2 (YP_003573111) | 57 | 1e-177 | 496 | 2.A.36  The Monovalent Cation:Proton Antiporter-1 (CPA1) Family | 11 |
| CAHE_0590 | ABC transporter *Dyadobacter fermentans* DSM 18053 (YP_003088332) | 59 | 7e-102 | 274 | 3.A.1  **The ATP-binding Cassette (ABC) Superfamily** | 1 |
| CAHE_0591 | hypothetical protein Aasi_1030 *Amoebophilus asiaticus* 5a2 (YP_001958107) | 52 | 4e-76 | 248 | 3.A.1  **The ATP-binding Cassette (ABC) Superfamily** | 6 |
| CAHE_0596 | metal-dependent phosphohydrolase *Amoebophilus asiaticus* 5a2 (YP_001957224) | 29 | 1e-25 | 318 | 2.A.21  The Solute:Sodium Symporter (SSS) Family | 7 |
| CAHE_0605 | lysine exporter protein LysE/YggA *Pseudomonas* sp. S9 (ZP_09710435) | 27 | 8e-23 | 219 | 2.A.76  The Resistance to Homoserine/Threonine (RhtB) Family | 6 |
| CAHE_0606 | hypothetical protein Aasi_0351 *Amoebophilus asiaticus* 5a2 (YP_001957505) | 48 | 0 | 613 | 3.A.1  **The ATP-binding Cassette (ABC) Superfamily** | 7 |
| CAHE_0633 | hypothetical protein Aasi_0939 *Amoebophilus asiaticus* 5a2 (YP_001958029) | 23 | 1e-44 | 960 | 2.A.21  The Solute:Sodium Symporter (SSS) Family | 13 |
| CAHE_0634 | metal-dependent phosphohydrolase *Amoebophilus asiaticus* 5a2 (YP_001957224) | 37 | 7e-36 | 243 | 2.A.21  The Solute:Sodium Symporter (SSS) Family | 7 |
| CAHE_0645 | unnamed protein product *Shewanella piezotolerans* WP3 (YP_002313634) | 29 | 5e-14 | 387 | 2.A.13  The C_4_-Dicarboxylate Uptake (Dcu) Family | 10 |
| CAHE_0647 | hypothetical protein Aasi_0240 *Amoebophilus asiaticus* 5a2 (YP_001957412) | 46 | 2e-121 | 437 | 2.A.13  The C_4_-Dicarboxylate Uptake (Dcu) Family | 11 |
| CAHE_0654 | hypothetical protein Aasi_0074 *Amoebophilus asiaticus* 5a2 (YP_001957252) | 38 | 4e-87 | 413 | 3.A.1  **The ATP-binding Cassette (ABC) Superfamily** | 4 |
| CAHE_0717 | hypothetical protein Aasi_0164 *Amoebophilus asiaticus* 5a2 (YP_001957338) | 46 | 2e-55 | 204 | no hit | 3 |
| CAHE_0718 | hypothetical protein Aasi_1035 *Amoebophilus asiaticus* 5a2 (YP_001958112) | 54 | 1e-85 | 220 | no hit | 1 |
| CAHE_0719 | hypothetical protein Aasi_1794 *Amoebophilus asiaticus* 5a2 (YP_003573184) | 53 | 8e-109 | 313 | 3.A.1  **The ATP-binding Cassette (ABC) Superfamily** | 1 |
| CAHE_0720 | hypothetical protein Aasi_1794 *Amoebophilus asiaticus* 5a2 (YP_003573184) | 50 | 1e-103 | 312 | 3.A.1  **The ATP-binding Cassette (ABC) Superfamily** | 1 |
| CAHE_0721 | hypothetical protein Aasi_1033 *Amoebophilus asiaticus* 5a2 (YP_001958110) | 56 | 4e-94 | 254 | 3.A.1  **The ATP-binding Cassette (ABC) Superfamily** | 1 |
| CAHE_0741 | hypothetical protein Aasi_1024 *Amoebophilus asiaticus* 5a2 (YP_001958101) | 62 | 0 | 534 | 3.A.1  **The ATP-binding Cassette (ABC) Superfamily** | 1 |
| CAHE_0754 | unnamed protein product Candidatus *Protochlamydia amoebophila* UWE25 (YP_008332) | 43 | 1e-159 | 587 | 3.A.1  **The ATP-binding Cassette (ABC) Superfamily** | 4 |
| CAHE_0759 | large conductance mechanosensitive channel protein *Nostoc punctiforme* PCC 73102 (YP_001866291) | 45 | 2e-28 | 121 | 1.A.22  **The Large Conductance Mechanosensitive Ion Channel (MscL) Family** | 2 |
| CAHE_0772 | Na-solute symporter *Amoebophilus asiaticus* 5a2 (YP_003573149) | 24 | 2e-07 | 693 | 3.A.1  **The ATP-binding Cassette (ABC) Superfamily** | 8 |
| CAHE_0775 | hypothetical protein Aasi_1444 *Amoebophilus asiaticus* 5a2 (YP_001958462) | 24 | 2e-10 | 733 | 9.B.74  no hit | 6 |
| CAHE_0788 | hypothetical protein NRI_0890 *Neorickettsia risticii* str. Illinois (YP_003082092) | 34 | 1e-32 | 255 | 3.A.1  **The ATP-binding Cassette (ABC) Superfamily** | 6 |
| CAHE_0789 | tlc2 gene product *Rickettsia bellii* RML369-C (YP_538160) | 51 | 4e-157 | 504 | 2.A.12  The ATP:ADP Antiporter (AAA) Family | 12 |
| CAHE_0796 | hypothetical protein Aasi_0939 *Amoebophilus asiaticus* 5a2 (YP_001958029) | 25 | 9e-89 | 1180 | 2.A.21  The Solute:Sodium Symporter (SSS) Family | 20 |
| CAHE_0838 | proline/betaine transporter (proP4) *Rickettsia prowazekii* str. Madrid E (NP_221046) | 30 | 2e-58 | 413 | 2.A.1  **The Major Facilitator Superfamily (MFS)** | 12 |
