# Supplementary material for: Comparative Genomics Suggests an Independent Origin of Cytoplasmic Incompatibility in Cardinium hertigii
Source: PLoS Genet. 2012 Oct 25;8(10):e1003012. doi: 10.1371/journal.pgen.1003012 (PMC3486910; doi:10.1371/journal.pgen.1003012)
Supplement: Table S5 — Comparison of the Amoebophilus asiaticus AFP-like gene cluster (as query) with the Cardinium hertigii AFP-like gene cluster and the Serratia entomophila AFP on the pADAP plasmid by blast. Blast results obtained using psi-blast are labeled with an asterisk; I, amino acid identity to best blast hit; E, E-value; n.d., not determined. (DOCX) [file pgen.1003012.s012.docx]

**Table S5.** Comparison of the *Amoebophilus asiaticus* AFP-like gene cluster (as query) with the *Cardinium hertigii* AFP-like gene cluster and the *Serratia entomophila* AFP on the pADAP plasmid by blast.

| ***Amoebophilus* AFP-like protein** | ***Cardinium* (locus_tag, aa identities to *Amoebophilus* AFP-like, E-value, PFAM domain)** | ***Serratia* (AFP_tag, aa identities to AFP, E-value, PFAM domain)** | **putative function** |
| --- | --- | --- | --- |
| Aasi_0232 | CAHE_0409 , I=65%, E=1E-236, AAA (PF00004), *Peptidase_M41* (PF01434) | Afp15, I=26%, E= 5e-16, *AAA* (PF00004) | ATPase |
| Aasi_0557 | CAHE_0037**,** I=42%, E=1e-283, *Baseplate_J* (PF04865) | Afp11, I=24%/35%, E=1e-15/2e-8, - | phage baseplate |
| Aasi_0556 | CAHE_0036, I=64%, E=3e-96, - | *Afp13, I=31%, E=0.8, *Adeno_shaft* (PF00608) | virus fibre protein |
| Aasi_1072 | CAHE_0456, I=37%, E=9e-30, - | *Afp16, I=9%, E=0.069, - | n.d. |
| Aasi_1073 | CAHE_0457, I=35%, E=5e-41, - | - | n.d. |
| Aasi_1074 | CAHE_0458, I=76%, E=4e-228, Phage_sheath_1 (PF04984) | Afp3, I=49%, E=7e-42, Phage_sheath_1 (PF04984)  Afp2, I=48%, E=3e-41, Phage_sheath_1 (PF04984)  Afp4, I=26%, E=3e-31, | phage tail sheath |
| Aasi_1075 | CAHE_0459, I=29%, E=3e-20, - | - | n.d. |
| Aasi_1076 | CAHE_0460, I=30%, E=1e-10, - | - | n.d. |
| Aasi_1077 | CAHE_0461, I=41%, E=1e-26, *Phage_T4_gp19* (PF06841) | Afp1, I=23%, E=9.9, *Phage_T4_gp19* (PF06841)  Afp5, I=22%, E=3e-21, *Phage_T4_gp19* (PF06841) | phage tail tube |
| Aasi_1078 | CAHE_0462, I=48%, E=0.011, - | - | n.d. |
| Aasi_1079 | CAHE_0463, I=35%, E=2e-36, - | Afp7, I=27%, E=3e-09, - | n.d. |
| Aasi_1080 | CAHE_0763, I=45%, E=3e-153, Phage_GPD (PF05954)/Phage_base_V (PF04717) | Afp8, I=22%, E=2e-26, Phage_GPD (PF05954)/Phage_base_V (PF04717) | VgrG |
| Aasi_1081 | CAHE_0762, I=64%, E=9e-39, PAAR_motif (PF05488) | - | n.d. |
| Aasi_1082 | CAHE_0761, I=48%, E=1e-32, GPW_gp25 (PF04965) | *Afp9, I=29%, E=5e-29, GPW_gp25 (PF04965) | lysozyme |
| Aasi_1083 | CAHE_0760, I=44%, E=5e-202, - | Afp12, I=22%, E=2e-15, - | n.d. |
| Aasi_1806 | CAHE_0118, I=24%/25%, E=3e-67/8e-35, - | Afp14, I=15%/12%, E=8e-30/1, - | n.d. |
