# Supplementary material for: Comparative Genomics Suggests an Independent Origin of Cytoplasmic Incompatibility in Cardinium hertigii
Source: PLoS Genet. 2012 Oct 25;8(10):e1003012. doi: 10.1371/journal.pgen.1003012 (PMC3486910; doi:10.1371/journal.pgen.1003012)
Supplement: Table S6 — Comparison of the Serratia entomophila AFP gene cluster (as query) with the AFP-like gene cluster of Cardinium hertigii and Amoebophilus asiaticus by blastp. I, amino acid identity to best blast hit; E, E-value; n.d., not determined. (DOCX) [file pgen.1003012.s013.docx]

**Table S6.** Comparison of the *Serratia entomophila* AFP gene cluster (as query) with the AFP-like gene cluster of *Cardinium* *hertigii* and *Amoebophilus asiaticus* by blastp.

| ***S. entomophila* AFP protein** | ***Cardinium* (locus_taq, aa identities to AFP, E-value, PFAM domain)** | ***Amoebophilus* (locus_taq, aa identities to AFP, E-value, PFAM domain)** | **putative function** |
| --- | --- | --- | --- |
| Afp1 | - | Aasi_1077, I=23%, E=0.081, *Phage_T4_gp19* (PF06841) | phage tail tube |
| Afp2 | CAHE_0458, I=45%, E=2e-40, Phage_sheath_1 (PF04984) | Aasi_1074, I=49%/27%, E=3e-42/0.21*,* Phage_sheath_1 (PF04984) | phage tail sheath |
| Afp3 | CAHE_0458, I=33%, E=1e-49, Phage_sheath_1 (PF04984) | Aasi_1074, I=50%/38%, E=2e-42/0.89, Phage_sheath_1 (PF04984) | phage tail sheath |
| Afp4 | CAHE_0458, I=36%, E=3e-26, Phage_sheath_1 (PF04984) | Aasi_1074, I=38%/23%, E=2e-26/6e-05, Phage_sheath_1 (PF04984) | phage tail sheath |
| Afp5 | CAHE_0461, I=22%, E=0.074, *Phage_T4_gp19* (PF06841) | Aasi_1077, I=25%, E=1.8, *Phage_T4_gp19* (PF06841) | phage tail tube |
| Afp6 | - | - | n.d. |
| Afp7 | CAHE_0463, I=28%, E=5e-09, - | Aasi_1079, I=27%, E=3e-12, - | n.d. |
| Afp8 | CAHE_0763, I=24%, E=2e-37, Phage_GPD (PF05954)/Phage_base_V (PF04717) | Aasi_1080, I=22%, E=8e-27, Phage_GPD (PF05954)/Phage_base_V (PF04717) | VgrG |
| Afp9 | CAHE_0761, I=29%, E=6e-13, GPW_gp25 (PF04965) | Aasi_1082, I=31%, E=4e-09, GPW_gp25 (PF04965) | lysozyme |
| Afp10 | - | - | n.d. |
| Afp11 | CAHE_0037, I=22%/31%, E=9e-19/6e-10, - | Aasi_0557, I=24%/I=35%, E=3e-18/6e-11, Baseplate_J (PF04865) | phage baseplate |
| Afp12 | CAHE_0760, I=24%/34%, E=3e-17/0.71, - | Aasi_1083, I=22%, E=4e-17, - | n.d. |
| Afp13 | - | - | virus fibre protein |
| Afp14 | - | Aasi_1806, I=24%, E=0.089, - | n.d. |
| AFP15 | CAHE_0409, I=26%, E=4e-11, - | Aasi_0232 I=27%, E=6e-09, AAA (PF00004), Peptidase_M41 (PF01434) | ATPase |
| Afp16 | - | - | n.d. |
| Afp17 | - | - | virulence factor |
| Afp18 | - | - | virulence factor |
