# Supplementary material for: Comparative Genomics Suggests an Independent Origin of Cytoplasmic Incompatibility in Cardinium hertigii
Source: PLoS Genet. 2012 Oct 25;8(10):e1003012. doi: 10.1371/journal.pgen.1003012 (PMC3486910; doi:10.1371/journal.pgen.1003012)
Supplement: Table S7 — Phenotypes of different Cardinium hertigii strains, their Encarsia wasp hosts, and presence of selected genes detected by PCR. CI (cytoplasmic incompatibility inducing), PI (parthenogensis inducing). (DOCX) [file pgen.1003012.s014.docx]

**Table S7:** Phenotypes of different *Cardinium* *hertigii* strains, their *Encarsia* wasp hosts, and presence of selected genes detected by PCR.

|  |  | ***c*Eina2** | ***c*Eper2** | ***c*Ehis1** | ***c*Eina1** |
| --- | --- | --- | --- | --- | --- |
| **phenotype** |  | CI | PI | PI | no phenotype observed |
| ***Encarsia* wasp host** |  | *E. inaron* | *E. pergandiella* | *E. hispida* | *E. inaron* |
| **Collection locality** |  | Campania, Italy | Brazil | San Diego, CA, USA | Tucson, AZ, USA |
| **reference** |  | – | [1] | [2] | [3] |
| ***Cardinium* gene** | **description** |  |  |  |  |
| CAHE_0458 | AFP-like gene | present | present | present | present |
| CAHE_0760 | AFP-like gene | present | present | present | present |
| CAHE_0763 | AFP-like gene | present | present | present | present |
| CAHE_0564 | bioA | present | present | not detectable | not detectable |
| CAHE_0677 | DEAD box RNA helicase | present | present | present | present |
| CAHE_0604 | protein of unknown function | present | present | present | present |
| CAHE_0028 | Ubiquitin specific protease | present | present | present | present |
|  |  |  |  |  |  |

**References**

1. Deshaies RJ, Joazeiro CA (2009) RING domain E3 ubiquitin ligases. Annu Rev Biochem 78: 399-434.

2. Katoh K, Toh H (2008) Recent developments in the MAFFT multiple sequence alignment program. Brief Bioinform 9: 286-298.

3. Hu M, Li P, Li M, Li W, Yao T, et al. (2002) Crystal Structure of a UBP-Family Deubiquitinating Enzyme in Isolation and in Complex with Ubiquitin Aldehyde. Cell 111: 1041-1054.
