# Supplementary material for: Comparative Genomics Suggests an Independent Origin of Cytoplasmic Incompatibility in Cardinium hertigii
Source: PLoS Genet. 2012 Oct 25;8(10):e1003012. doi: 10.1371/journal.pgen.1003012 (PMC3486910; doi:10.1371/journal.pgen.1003012)
Supplement: Table S8 — Proteins of Cardinium hertigii likely involved in host cell interaction. I, amino acid identity to best blast hit; E, E-value; n.a., not applicable; n.p., not present. (DOCX) [file pgen.1003012.s015.docx]

**Table S8:** Proteins of *Cardinium hertigii* likely involved in host cell interaction.

| ***Car dinium* locus tag** | **best blast hit (GenBank accession no., aa identities to best blast hit, E-value, PFAM domains)** | **number of TPR or AR predicted (SMART/PFAM)** | **G+C content in %** | **length (amino acids)** | **locus tags of *Wolbachia* homologs (*w*Mel, *w*Pip, *w*Ri)** |
| --- | --- | --- | --- | --- | --- |
| **TPRs** |  |  |  |  |  |
| CAHE_0312 | hypothetical protein Aasi_1962 *Amoebophilus asiaticus* (YP_003573286, I=34%, E=1e-157) | 10/5 | 39 | 1008 | n.a. |
| CAHE_0450 | hypothetical protein Aasi_0827 *Amoebophilus asiaticus* (YP_001957929, I=32%, E=2e-47) | 4/2 | 38 | 542 | n.a. |
| CAHE_0452 | hypothetical protein Aasi_0496 *Amoebophilus asiaticus* (YP_001957632, I=45%, E=4e-55) | 0/2 | 35 | 231 | n.a. |
| **ANKs** |  |  |  |  |  |
| CAHE_0040 | ankyrin *Thermofilum pendens* Hrk 5 (YP_920685, I=29%, E=0.033) | 1/3 | 33 | 364 | n.a. |
| CAHE_0095 | ankyrin 2,3/unc44 *Aedes aegypti* (XP_001649474, I=34%, E=6e-112) | 19/21 | 38 | 711 |  |
| CAHE_0205 | ankyrin repeat-containing protein *Metallosphaera yellowstonensis* MK1 (ZP_09704886, I=54%, E=6e-19) | 3/3 | 32 | 140 | n.a. |
| CAHE_0206 | ankyrin 2,3/unc44 *Aedes aegypti* (XP_001649474, I=44%, E=2e-47) | 6/8+ | 33 | 245 | n.a. |
| CAHE_0396 | ankyrin 2,3/unc44 *Aedes aegypti* (XP_001649474, I=40%, E=2e-55) | 8/7+ | 36 | 322 |  |
| CAHE_0397 | ankyrin repeat protein *Penicillium marneffei* ATCC 18224 (XP_002145964, I=47%, E=0.001) | 1/0 | 38 | 98 | n.a. |
| CAHE_0435 | ankyrin repeat-containing protein *Rickettsia bellii* OSU 85-389 (YP_001496599, I=31%, E=5e-16) | 6/3 | 35 | 393 | n.a. |
| CAHE_0488 | serine/threonine-protein kinase ripk4 *Aedes aegypti (*XP_001649473, I=36%, E=3e-19) | 4/6 | 35 | 182 | n.a. |
| CAHE_0491 | hypothetical protein Aasi_1153 *Amoebophilus asiaticus* (YP_001958218, I=43%, E=8e-10) | 2/3 | 39 | 159 | n.a. |
| CAHE_0588 | ankyrin repeat protein *Trichomonas vaginalis* G3 (XP_001314730, I=30%, E=4e-36) | 9/9 | 37 | 373 | n.a. |
| CAHE_0632 | pfs *Arthroderma otae* CBS 113480 (XP_002843051, I=38%, E=8e-33) | 6/5 | 34 | 221 | n.a. |
| CAHE_0670 | ankyrin *Sulfolobus islandicus* Y.N.15.51 (YP_002840971, I=37%, E=1e-23) | 5/6 | 39 | 221 | n.a. |
| CAHE_0680 | hypothetical protein LOC100635111 *Amphimedon queenslandica* (XP_003387964, I=31%, E=9e-111) | 27/30 | 43 | 992 | n.a. |
| CAHE_0834 | ankyrin repeat protein *Aspergillus fumigatus* A1163 (EDP48589, I=33%, E=3e-19) | 7/3 | 35 | 397 | n.a. |
| CAHE_p0007 | hypothetical protein *Trichomonas vaginalis* G3 (XP_001303050, I=40%, E=1e-08) | 2/3 | 34 | 264 | n.a. |
| CAHE_p0014 | hypothetical protein TRIATDRAFT_317156 *Trichoderma atroviride* IMI 206040 (EHK47348, I=36%, E=4e-11) | 3/3 | 33 | 324 | n.a. |
| CAHE_p0019 | ankyrin 2,3/unc44 *Aedes aegypti* (XP_001649474, I=42%, E=2e-38) | 7/9 | 28 | 298 | n.a. |
| CAHE_p0026 | ankyrin repeat protein *Trichomonas vaginalis* G3 (XP_001309767, I=28%, E=2e-36)+ RING domain (predicted with SMART) | 9/9 | 33 | 567 | n.a. |
| CAHE_p0059 | ankyrin *Sulfolobus islandicus* Y.N.15.51 (YP_002840971, I=38%, E=1e-34) | 6/7 | 29 | 262 | n.a. |
| **other putative host cell interaction proteins** | |  |  |  |  |
| CAHE_0677 | DEAD/DEAH box helicase *Micavibrio aeruginosavorus* ARL-13 (YP_004865735, I=51%, E=2e-124, *DEAD* (PF00270), *Helicase_C* (PF00271)) | n.a. | 40 | 390 | WD1236,  WPa_1078, WRi_012100 |
| CAHE_0676 | cold-shock DNA-binding domain-containing protein *Rhodopseudomonas palustris* BisA53 (YP_783219, I=56%, E=5e-21, *CSD* (PF00313)) | n.a. | 37 | 70 | WD1235, WPa_1077,  WRi_012090 |
| CAHE_0028 | similar to Ubiquitin specific peptidase 3 *Strongylocentrotus purpuratus*(XP_781718, I=29%, E= 1e-14, *UCH* (PF00443)) | n.a. |  | 406 | n.p. |
| CAHE_0010 | hypothetical protein SINV_12937 *Solenopsis invicta* (EFZ09768, I=42%, E=1e-04, *WH2* (PF02205)) | n.a. | 40 | 360 | n.a. |
| CAHE_0286 | patatin family protein *Wolbachia endosymbiont* of *Culex quinquefasciatus* JHB (ZP_03335586, I=64%, E=1e-141, *Patatin* (PF01734)) | n.a. | 31 | 316 | WD0565, WPa_1340, WRi_006880 |
| CAHE_0706 | collagen triple helix repeat-containing protein *Cellulophaga lytica* DSM 7489 (YP_004261222, I=61%, E= 4e-58, *Collagen* (PF01391)) | n.a. | 50 | 337 | n.p. |
