# Supplementary material for: Comparative Genomics Suggests an Independent Origin of Cytoplasmic Incompatibility in Cardinium hertigii
Source: PLoS Genet. 2012 Oct 25;8(10):e1003012. doi: 10.1371/journal.pgen.1003012 (PMC3486910; doi:10.1371/journal.pgen.1003012)
Supplement: Table S9 — Cardinium hertigii genes putatively involved in past horizontal gene transfer events. Nearest neighbors in phylogenetic trees are indicated (neighbor-joining trees, 2000 bootstrap replications; maximum-likelihood trees, 100 bootstrap replications; Figure S6). Genes encoding transposases, repeat proteins and Na+/proline symporters, and genes shared with Amoebophilus are not listed. AM, amoeba associated bacteria; AA, Rickettsia that are able to multiply in amoebae and arthropods; ART, arthropod associated bacteria; E, eukaryotes; X, other bacteria; n.a., not applicable. Nodes with a bootstrap higher than 75 and the same group are indicated with an asterisk. (DOCX) [file pgen.1003012.s016.docx]

**Table S9:** *Cardinium hertigii* genes putatively involved in past horizontal gene transfer events.

|  | **neighbor-joining** | | | **maximum-likelihood** | | |
| --- | --- | --- | --- | --- | --- | --- |
| ***Cardinium* locus_tag** | **category** | **bootstrap** | **nearest neighbor** | **category** | **bootstrap** | **nearest neighbor** |
| CAHE_0002 | X | 46 | *Neorickettsia sennetsu* | ART | 7 | *Bacillus thuringiensis* |
| CAHE_0010 | E | n.a. | *Nasonia vitripennis* | E | n.a. | *Entamoeba histolytica* |
| CAHE_0011 | X | 21 | *Enterobacteria* phage | AM | 10 | *Parachlamydia acanthamoebae* |
| CAHE_0018* | AM | 97 | *Simkania negevensis Z* | AM | 100 | *Simkania negevensis Z* |
| CAHE_0028 | E | 17 | *Candida glabrata* | E | 30 | *Trichomonas vaginalis* |
| CAHE_0052* | ART | 100 | *Candidatus* Midichloria mitochondrii | ART | 100 | *Candidatus* Midichloria mitochondrii |
| CAHE_0103 | X | 29 | *Hirschia baltica* | ART | 52 | *Rickettsia endosymbiont of Ixodes scapularis* |
| CAHE_0120* | ART | 99 | *Candidatus* Midichloria mitochondrii | ART | 100 | *Candidatus* Midichloria mitochondrii |
| CAHE_0149 | X | 15 | *Mycoplasma agalactiae* | X | 4 | *Mycoplasma capricolum* |
| CAHE_0211 | X | 28 | *Mycoplasma genitalium* | X | 21 | *Mycoplasma genitalium* |
| CAHE_0213 | X | 45 | *Thermodesulfovibrio yellowstonii* | X | 40 | *Nitrosococcus halophilus* |
| CAHE_0218* | E | 100 | *Thalassiosira pseudonana* | E | 100 | *Aureococcus anophagefferens* |
| CAHE_0221 | X | 87 | *Stigmatella aurantiaca* | X | 50 | *Abiotrophia defectiva* |
| CAHE_0230 | ART | n.a. | *Arsenophonus nasoniae* | ART | 100 | *Arsenophonus nasoniae* |
| CAHE_0232 | X | n.a. | *Micavibrio aeruginosavorus* | X | 66 | *Micavibrio aeruginosavorus* |
| CAHE_0239 | E | n.a. | *Thalassiosira pseudonana* | E | 100 | *Enterocytozoon bieneusi* |
| CAHE_0274 | E | 7 | *Ixodes scapularis* | E | 9 | *Daphnia pulex* |
| CAHE_0280 | X | n.a. | *Shewanella frigidimarina* | X | 12 | *Rhodobacter sphaeroides* |
| CAHE_0286 | ART | 100 | *Wolbachia* endosymbiont of *Culex quinquefasciatus* JHB | ART | 35 | *Wolbachia* endosymbiont of *Culex quinquefasciatus* Pel |
| CAHE_0287* | ART | 99 | *Photorhabdus luminescens* | ART | 94 | *Photorhabdus luminescens* |
| CAHE_0295 | n.a. | n.a. | n.a. | X | 47 | gamma proteobacterium |
| CAHE_0297 | AM | 49 | *Simkania negevensis Z* | AM | 72 | *Simkania negevensis Z* |
| CAHE_0322* | AA | 99 | *Rickettsia bellii* | AA | 84 | *Rickettsia bellii* |
| CAHE_0376 | AM | n.a. | *Waddlia chrondophila* | X | 44 | *Caldithrix abyssi* |
| CAHE_0377 | X | 43 | *Caldithrix abyssi* | X | 59 | *Caldithrix abyssi* |
| CAHE_0378* | E | 99 | *Encephalitozoon intestinalis* | E | 96 | *Encephalitozoon intestinalis* |
| CAHE_0379 | X | n.a. | *Agrobacterium radiobacter* | X | n.a. | *Clostridium bartlettii* |
| CAHE_0393 | AM | 98 | *Candidatus* Odyssella thessalonicensis | AA | 92 | *Rickettsia bellii* |
| CAHE_0432 | X | 58 | *Francisella* sp. | X | 83 | *Kangiella koreensis* |
| CAHE_0433 | X | 10 | *Paenibacillus terrae* | X | 20 | *Myxococcus xanthus* |
| CAHE_0437 | ART | 64 | *Wolbachia endosymbiont of Culex quinquefasciatus Pel* | X | 24 | *Phytophthora sojae* |
| CAHE_0442* | X | 97 | *Bartonella grahamii* | X | 92 | *Bartonella grahamii* |
| CAHE_0443* | AA | 98 | *Rickettsia bellii* | AA | 98 | *Rickettsia bellii* |
| CAHE_0444* | AA | 99 | *Rickettsia bellii* | AA | 95 | *Rickettsia bellii* |
| CAHE_0529 | ART | 51 | *Candidatus* Regiella insecticola | ART | 55 | *Candidatus* Regiella insecticola |
| CAHE_0550 | X | 42 | *Desulfomicrobium baculatum* | X | 42 | *Persephonella marina* |
| CAHE_0554* | AM | 100 | *Legionella drancourtii* | AM | 100 | *Legionella drancourtii* |
| CAHE_0559 | ART | 22 | *Candidatus* Midichloria mitochondrii | AA | 48 | *Rickettsia felis* |
| CAHE_0560* | ART | 93 | *Rickettsia endosymbiont of Ixodes scapularis* | ART | 75 | *Rickettsia endosymbiont of Ixodes scapularis* |
| CAHE_0561 | ART | 37 | *Candidatus* Midichloria mitochondrii | ART | 23 | *Candidatus* Midichloria mitochondrii |
| CAHE_0562 | X | 52 | *Lawsonia intracellularis* | X | 69 | *Neorickettsia sennetsu* |
| CAHE_0563* | ART | 94 | *Rickettsia endosymbiont of Ixodes scapularis* | ART | 92 | *Rickettsia endosymbiont of Ixodes scapularis* |
| CAHE_0564* | X | 89 | *Lawsonia intracellularis* | X | 82 | *Lawsonia intracellularis* |
| CAHE_0570* | AA | 100 | *Rickettsia felis* | AA | 100 | *Rickettsia felis* |
| CAHE_0571 | X | n.a. | *Sporolactobacillus inulinus* | X | 49 | *Listeriaceae bacterium* |
| CAHE_0603* | ART | 100 | *Orientia tsustugamushi* | ART | 100 | *Orientia tsustugamushi* |
| CAHE_0604 | ART | 82 | *Rickettsia canadensis* | ART | 58 | *Rickettsia canadensis* |
| CAHE_0605 | X | n.a. | *Serratia proteamaculans* | X | n.a. | *Serratia proteamaculans* |
| CAHE_0608* | AA | 99 | *Rickettsia prowazekii* | AA | 100 | *Rickettsia bellii* |
| CAHE_0618 | X | 19 | *Streptomyces zinciresistens* | X | 0 | *Parascardovia denticolens* |
| CAHE_0619 | X | 50 | *Picrophilus torridus* | X | 53 | *Streptococcus suis* |
| CAHE_0648* | AM | 85 | *Simkania negevensis Z* | AM | 75 | *Simkania negevensis Z* |
| CAHE_0662 | X | n.a. | *Agrobacterium vitis* | ART | n.a. | *Orientia tsustugamushi* |
| CAHE_0663 | X | 7 | *Starkeya novella* | X | 9 | *Roseomonas cervicalis* |
| CAHE_0676 | X | n.a. | *Paracoccus denitrificans* | n.a. | n.a. | n.a. |
| CAHE_0692* | X | 99 | *Rickettsia gyrilli* | X | 97 | *Rickettsia gyrilli* |
| CAHE_0706 | E | 93 | *Amphimedon queenslandica* | X | 7 | *Epulopiscium* sp. |
| CAHE_0731 | X | 31 | *Roseobacter* sp. | X | 9 | *Bradyrhizobium* sp. |
| CAHE_0740 | X | 13 | *Methylomonas methanica* | ART | 48 | *Candidatus* Midichloria mitochondrii |
| CAHE_0754 | ART | 34 | *Candidatus* Midichloria mitochondrii | AM | 47 | *Candidatus* Protochlamydia amoebophila |
| CAHE_0765 | E | n.a. | *Bombus impatiens* | E | n.a. | *Bombus impatiens* |
| CAHE_0780 | X | 49 | *Cytophaga hutchinsonii* | X | 31 | *Cytophaga hutchinsonii* |
| CAHE_0781 | E | n.a. | *Ascaris suum* | E | 97 | *Ascaris suum* |
| CAHE_0788 | X | 42 | *Neorickettsia risticii* | X | 36 | *Neorickettsia risticii* |
| CAHE_0789* | ART | 100 | *Orientia tsustugamushi* | ART | 100 | *Orientia tsustugamushi* |
| CAHE_0835* | ART | 100 | *Rickettsiella gyrilli* | ART | 100 | *Rickettsiella gyrilli* |
| CAHE_0838 | ART | 97 | *Orientia tsustugamushi* | ART | 48 | *Orientia tsustugamushi* |
| CAHE_0846 | X | 37 | *Burkholderia xenovorans* | X | 37 | *Burkholderia vietnamiensis* |
