# Supplementary material for: Comparative Genomics Suggests an Independent Origin of Cytoplasmic Incompatibility in Cardinium hertigii
Source: PLoS Genet. 2012 Oct 25;8(10):e1003012. doi: 10.1371/journal.pgen.1003012 (PMC3486910; doi:10.1371/journal.pgen.1003012)
Supplement: Table S10 — Primers used for the detection of putative host cell interaction genes in different Cardinium hertigii strains (Table S7). (DOCX) [file pgen.1003012.s017.docx]

**Table S10:** Primers used for the detection of putative host cell interaction genes in different *Cardinium hertigii* strains (Table S7).

| ***Cardinium* locus tag** | **forward primer sequence (5‘ - 3‘)** | **reverse primer sequence (5‘ - 3‘)** | **expected length of amplificate [bp]** | **annealing temperature [°C]** |
| --- | --- | --- | --- | --- |
| CAHE_0458 | TTTTTGACAATGGTGGAGCA | GGTTCTTGCACCCCAAACTA | 809 | 61 |
| CAHE_0760 | GAAGGCATTTGGTGTCGTTT | GTGATACTGCCCCTTGCATT | 403 | 58 |
| CAHE_0763 | TTGCAGGTCATTCAGCAAAG | AGCGGTTTCATCAGCCTCTA | 1020 | 61 |
| CAHE_0564 | TGCCATTAAAAGGGGCTATG | TGATGAATGGCTTGAATGGA | 954 | 47 |
| CAHE_0677 | CCCATTGGCAAGCAGTTAAT | GCAGCTTGGGGAAAATCATA | 635 | 57 |
| CAHE_0604 | TGGCTTTATTATTTGCGTCTG | GCCAAACCTTACCCATAGGAA | 402 | 54 |
| CAHE_0028 | TGCAGTGCTGCAGATTATTG | TTCATTGCGTTTTACGTAGGC | 787 | 54 |
