# Supplementary material for: Comparative Genomics Suggests an Independent Origin of Cytoplasmic Incompatibility in Cardinium hertigii
Source: PLoS Genet. 2012 Oct 25;8(10):e1003012. doi: 10.1371/journal.pgen.1003012 (PMC3486910; doi:10.1371/journal.pgen.1003012)
Supplement: Table S11 — NCBI accession numbers of proteins from the biotin biosynthesis pathway used for a concatenated data set for the calculation of a phylogenetic tree with the maximum likelihood algorithm. (DOCX) [file pgen.1003012.s018.docx]

**Table S11.** NCBI accession numbers of proteins from the biotin biosynthesis pathway used for a concatenated data set for the calculation of a phylogenetic tree with the maximum likelihood algorithm

| **organism** | **BioA** | **BioD** | **BioC** | **BioH** | **BioF** | **BioB** |
| --- | --- | --- | --- | --- | --- | --- |
| *Cardinium hertigii c*Eper1 | CAHE_0564 | CAHE_0563 | CAHE_0562 | CAHE_0561 | CAHE_0560 | CAHE_0559 |
| *Rickettsia* endosymbiont of *Ixodes scapularis* | ZP_04698248 | ZP_04698249 | ZP_04698250 | ZP_04698251 | ZP_04698252 | ZP_04698253 |
| *Lawsonia intracellularis* str. PHE/MN1-00 | YP_594802 | YP_594801 | YP_594800 | YP_594799 | YP_594798 | YP_594797 |
| *Neorickettsia risticii* str. Illinois | YP_003081804 | YP_003081804 | YP_003081802 | YP_003081801 | YP_003081800 | YP_003081798 |
| *Neorickettsia sennetsu* str. Miyayama | YP_506498 | YP_506497 | YP_506495 | YP_506494 | YP_506493 | YP_506492 |
| *Magnetospirillum magneticum* str. AMB-1 | YP_422126 | YP_422125 | YP_422124 | YP_422123 | YP_422122 | YP_422120 |
| *Achromobacter piechaudii* str. ATCC 43553 | ZP_06685978 | ZP_06685981 | ZP_06685981 | ZP_06685980 | ZP_06685979 | ZP_06689510 |
| *Bordetella pertussis Bordetella pertussis* | NP_880815 | NP_880812 | NP_880812 | NP_880813 | NP_880814 | NP_881326 |
| *Gluconacetobacter diazotrophicus* str. PAl 5 | YP_001602164 | YP_001602164 | YP_001602167 | YP_001602166 | YP_001602165 | YP_001603186 |
| *Crocosphaera watsonii* str. WH 8501 | ZP_00514990 | ZP_00515520 | ZP_00515866 | ZP_00515865 | ZP_00515864 | ZP_00514954 |
| *Nitrococcus mobilis* str. Nb-231 | ZP_01125956 | ZP_01125955 | ZP_01127180 | ZP_01128606 | ZP_01128607 | ZP_01127181 |
| *Cyanothece* sp. ATCC 51142 | YP_001804508 | YP_001804930 | YP_001805563 | YP_001805564 | YP_001805565 | YP_001805764 |
| *Zymomonas mobilis* subsp. mobilis ZM4 | YP_163653 | YP_163650 | YP_163650 | YP_163651 | YP_163652 | YP_161829 |
| *Legionella pneumophila* | YP_123751 | YP_123755 | YP_124590 | YP_123754 | YP_123753 | YP_123752 |
| *Parachlamydia acanthamoebae* str. Hall's coccus | ZP_06299038 | ZP_06299039 | ZP_06299041 | ZP_06300545 | ZP_06299042 | ZP_06300151 |
| *Escherichia coli* str. K-12 substr. MG1655 | NP_415295 | NP_415299 | NP_415298 | NP_417871 | NP_415297 | NP_415296 |
| *Kurthia* sp. 538-KA26 | BAB39453 | BAB39454 | BAB39463 | BAB39462 | BAB39461 | BAB39458 |
| *Neisseria meningitidis* str. MC58 | NP_273774 | NP_273775 | NP_273521 | NP_273326 | NP_273519 | NP_274174 |
| *Pseudomonas aeruginosa* str. PAO1 | NP_249111 | NP_249195 | NP_249194 | NP_249193 | NP_249192 | NP_249191 |
| *Vibrio cholerae* str. B33 | ZP_04400515 | ZP_04400519 | ZP_04400518 | ZP_04399326 | ZP_04400517 | ZP_04400516 |
| *Xylella fastidiosa* str. 9a5c | NP_297482 | NP_299755 | NP_299378 | NP_298645 | NP_298646 | NP_297357 |
| *Anaplasma centrale* str. Israel | YP_003328623 | YP_003328228 | YP_003328117 | - | YP_003328822 | YP_003328325 |
| *Ehrlichia ruminantium* str. Gardel | YP_196321 | YP_196818 | YP_195935 | - | YP_196094 | YP_196599 |
