# Supplementary material for: Comparative Genomics Suggests an Independent Origin of Cytoplasmic Incompatibility in Cardinium hertigii
Source: PLoS Genet. 2012 Oct 25;8(10):e1003012. doi: 10.1371/journal.pgen.1003012 (PMC3486910; doi:10.1371/journal.pgen.1003012)
Supplement: Figure S3 — Multiple sequence alignment of selected ubiquitin-specific proteases (USPs) with CAHE_0028, the USP of Cardinium hertigii. An amino acid sequence alignment of the catalytic core domains of selected USPs is shown. The alignment was done with MAFFT [45], shading of conserved amino acid residues was performed with Boxshade available at the Swiss EMBnet server (http://www.ch.embnet.org/software/BOX_form.html). Data for important amino acid residues are taken from [46]–[47]. Amino acid residues forming the catalytic triad are highlighted in red. Amino acid residues that have been shown to be involved in van der Waals contact with ubiquitin are highlighted in green. Amino acid residues that are involved in direct inter-molecular hydrogen bond interactions using their side chains and main chains are highlighted in blue. Amino acid residues are only highlighted if they were present in all aligned sequences. Regions of high sequence conservation within characterized USPs are underlined: Cys-box (215–229), QDE-box (292–305), His-box (446–468, 477–486, 512–520); numbering according to UBP7_HUMAN residues. The consensus is displayed at the bottom of each alignment block, asterisks indicate identical positions, dots indicate similar positions. Abbreviations and accession numbers: UBP2_HUMAN (human, O75604), UBP7_HUMAN (human, Q93009), UBP14_HUMAN (human, P54578), UBP4_YEAST (S. cerevisiae, CAA86791), UBP8_YEAST (S. cerevisiae, P50102), UBP15_YEAST (S. cerevisiae, P50101), Aasi_0770 (A. asiaticus, YP_001957879), Aasi_1805 (A. asiaticus, YP_003573189), USP_Cardinium (C. hertigii, CAHE_0028). (PDF) [file pgen.1003012.s003.pdf]

```
                            Cys223
                              ▼
UBP2_HUMAN   255 GLRNLGNTCFMNSILQCLSNTRELRDYCLQRLYMRDLHHGS--NAHTALVEEFAKLIQTI
UBP4_YEAST   503 GLENLGNSCYMNCIIQCILGTHELTQIFLDDSYAKHININSKLGSKGILAKYFARLVHMM
UBP8_YEAST   135 GLINMGSTCFMSSILQCLIHNPYFIRHSMSQIHSNNCKVRS---PDKCFSCALDKIVHEL
Aasi_0770     44 GIPNLKATCYMNSVLQIL-----------KAFYLPKVNEKS-----DELAKPLQALMQVI
Aasi_1805     35 GIGNLGNTCYMNSVLQIL-----------ASFYSNAFDKTK-----GPLGKTSRSLIKAI
CAHE_0028     76 GLPNIGNTCYMNAVLQII-----------AALYEDKIKSHS----------GLKNLVNKI
UBP14_HUMAN  103 GLTNLGNTCYMNATVQCIRSVPELKDA--LKRYAGALRASGEMASAQYITAALRDLFDSM
UBP7_HUMAN   212 GLKNQGATCYMNSLLQTLFFTNQ-----------------------------LRKAVYMM
UBP15_YEAST  204 GFRNQGATCYLNSLLQSYFFTKY-----------------------------FRKLVYEI
consensus    601 *. *....*......* .              .   .   .       .   .....  .

UBP2_HUMAN   313 WTSSPNDVVSPSEF--------------------KTQIQRYAP-RFVGYNQQDAQEFLR
UBP4_YEAST   563 Y----KEQVDGSKKISISPIKF-------------KLACGSVNS-LFKTASQQDCQEFCQ
UBP8_YEAST   192 YGALNTKQASSSSTSTNRQTGFIYLL----------TCAWKINQ-NLAGYSQQDAHEFWQ
Aasi_0770     88 Q----DDREAANQNEAIAVFEALH---------------HKFGW-TSDYESHEDAADLMD
Aasi_1805     79 R----GNEKVDNQEIAARAELFFKALKENEEEDEHKENIGGIGW-KPNIGAQEDASELLQ
CAHE_0028    115 N----NGHQPLKRE---EMEKFVTSL---------PENAQKMAK-EAQSGTQQDPHEFIA
UBP14_HUMAN  161 D-------KTSSSIPPIILLQFLHMAFPQFAEKG-----------EQGQYLQQDANECWI
UBP7_HUMAN   243 P----TEGDDSSKSVPLALQRVFYELQHSDKPVGTKKLTKSFGWETLDSFMQHDVQELCR
UBP15_YEAST  235 P----TEHESPNNSVPLALQRAFYQLQVSDIPLDTLELTRSFGWDTAESFTQHDVQELNR
consensus    661       .    .    . .  .   .                .      ....*...

UBP2_HUMAN   351 FLLDGLHNEVNRV---TLRPKSNPENL---DHLPDDEKGRQMWRKYLEREDSRIGDLFVG
UBP4_YEAST   605 FLLDGLHEDLNQCGSNPPLKELSQEAEARREKLSLRIASSIEWERFLTTDFSVIVDLFQG
UBP8_YEAST   241 FIINQIHQS----------------------YVLDLPNAKEVSRANNKQCECIVHTVFEG
Aasi_0770    128 KFLDWINIPLAATNTISIDPTTKEAGA---------------------------------
Aasi_1805    134 GIFDWLKLPKAKTIGTLIHPTTGNERS---------------------------------
CAHE_0028    158 NLEKELNLDRVGCGNRFLFKTTKKNKKNLYQQFNSIEDEKQERQLFVSLDEQKTTQCFLS
UBP14_HUMAN  203 QMMRVLQQKLEAI----------------EDDSVKETDSSSASAATPSKKKSLIDQFFGV
UBP7_HUMAN   299 VLLDNVENKMKGT-----------------------------------CVEGTIPKLFRG
UBP15_YEAST  291 ILMDRLENNMKGT-----------------------------------PVEGKLNEIFVG
consensus    721  ... . . .                                      .  .  .. .

UBP2_HUMAN   405 QLKSSLTCTDCGYCS-TVFDPFWDLSLPIAKRGY-PEVTLMDCMRLFTKEDVL-------
UBP4_YEAST   665 QYASRLKCKVCSHTS-TTYQPFTVLSIPIPKKNSRNNITIEDCFREFTKCENL-------
UBP8_YEAST   279 SLESSIVCPGCQNNSKTTIDPFLDLSLDIKDKKK-----LYECLDSFHKKEQL-------
Aasi_0770    155 ----------------PSDDQWFNYHVELPTERE-DLKTIQDFFNNSLVPDKVMAKLKED
Aasi_1805    161 ----------------SGKDPWSMLNVEMPQQSN--LTTMQNFVNNYLNSTGTREVKWSE
CAHE_0028    218 KMIALSREELVTDPC-VTKDPTRCSYYKGPKSSF-----------------------LKEN
UBP14_HUMAN  247 EFETTMKCTESEEEE-VTKGKENQLQLSCFINQE-----VKYLFTGL--KLRL-------
UBP7_HUMAN   324 KMVSYIQCKEVDYRS-DRREDYYDIQLSIKGKKN-----IFESFVDYVAVEQL-------
UBP15_YEAST  316 KMKSYIKCINVDYES-ARVEDFWDLQLNVKNFKN-----LQESFDNYIEMELM-------
consensus    781  . . . .      .  ...  . . .          . . .  .   . .

UBP2_HUMAN   456 DGDEKPTCCRCRGRKRCIKK--FSIQRFPKILVLHLKRFSE-------------SRIRTS
UBP4_YEAST   717 EVDEQWLCPHCEKRQPSTKQ--LTITRLPRNLIVHLKRFDN----------------LLN
UBP8_YEAST   327 -KDFNYHCGECNSTQDAIKQ--LGIHKLPSVLVLQLKRFEH-----------LLNGSNR
Aasi_0770    198 KSDRALD--RVKRLKDLDKL-------YKGMLILQLKRFATNGGTTRLDDRTYSKDFVKI
Aasi_1805    203 NDSINVDARYVPSLKDLDKL-------YGKMLVINLKRFGN----------LIPGIVTPP
CAHE_0028    255 ENNLEIQNQDPEKLQSYIQQHVMSKSKIPSILYVQLKRFTN-----------------ENT
UBP14_HUMAN  292 QEEITKQSPTLQRNALYIKS--SKISRLPAYLTIQMVRFFY-----------KEKESVNA
UBP7_HUMAN   371 DGDNKYDAGE-HGLQEAEKG--VKFLTLPPVLHLQLMRFMY-----------DPQTDQNI
UBP15_YEAST  363 NGENQYAAQD-YGLQDAQKG--VIFESFPPVLHLQLKRFEY-----------DFNYDQMV
consensus    841   .          ...  .   .   ... .*.....**
```

```
UBP2_HUMAN     501 KLTTFVNFPLRDLDLREFAS----------------------------------------
UBP4_YEAST     759 KNNDFVIYPFL-LDLTPFWANDFDGVFPPGVNDDELPIR---------------------
UBP8_YEAST     372 KLDDFIEFPTY-LNMKNYCST---------KEKDKHSEN---------------------
Aasi_0770      249 KIDKEVEEPFH-LIIKKEQTF---------------------------------------
Aasi_1805      246 KIKQEVEKPFS-LTVRHDQID---------------------------------------
CAHE_0028      299 KIETAVDGTFE-LTIKPDP-----------------------------------------
UBP14_HUMAN    339 KVLKDVKFPLM-LDMYELCTPELQEKMVSFRSKFKDLEDKKVNQQPNTSDKKSSPQKEVK
UBP7_HUMAN     417 KINDRFEFPEQ-LPLDEFLQK----------T----------------------------
UBP15_YEAST    409 KVNDKYEFPET-IDLSPFVDKDVLKKTLDSEN----------------------------
consensus      901 *.   ....   . .. .

                                                   His464           Asn/Asp481
                                                     ▼                ▼
UBP2_HUMAN     521 ----------ENTNHAVYNLYAVSNHSG-TTMGGHYTAYCRSPGTGEWHTFNDSSVTPM-
UBP4_YEAST     797 ----------GQIPPFKYELYGVACHFG-TLYGGHYTAYVKKGLKKGWLYFDDTKYKPVK
UBP8_YEAST     401 ----------GKVPDIIYELIGIVSHKG-TVNEGHYIAFCKISG-GQWFKFNDSMVSSI-
Aasi_0770      269 ----------ENEKNFYYKLVGFIRHKG-SLEEGHYKAYIKKD--GIWICYNDNDLSTV-
Aasi_1805      266 ----------GLSNNLYYELVGFINHIGEGLRRGHYIAYTKVG--KQWIEYDDSTVSPR-
CAHE_0028      317 ----------NLNETIHFDLHGFILHTG-STNGGHYIAYVKRN--EKWYEANDSSITQM-
UBP14_HUMAN    398 YEPFSFADDIGSNNCGYYDLQAVLTHQGRSSSSGHYVSWVKRK-QDEWIKFDDDKVSIV-
UBP7_HUMAN     438 ----------DPKDPANYILHAVLVHSG-DNHGGHYVVYLNPKGDGKWCKFDDDVVSRC-
UBP15_YEAST    440 ----------KDKNPYVYNLHGVLVHSG-DISTGHYYTLIKPGVEDQWYRFDDERVWRV-
consensus      961                  ..* ... * * ..  ***.....      *  ..*. .. .
```
